# Shared molecular profiles of post-laser vision correction ectasia and keratoconus with key differences in *CADPS*, *CPT1B*, *CIITA*, and *TBC1D4*

**DOI:** 10.3389/fmolb.2025.1616675

**Published:** 2025-08-06

**Authors:** Katarzyna Jaskiewicz-Rajewicz, Alicja Wysocka, Magdalena Maleszka-Kurpiel, Eliza Matuszewska-Mach, Jakub Wozniak, Rafal Ploski, Jan Matysiak, Malgorzata Rydzanicz, Marzena Gajecka

**Affiliations:** ^1^ Institute of Human Genetics, Polish Academy of Sciences, Poznan, Poland; ^2^ Optegra Eye Healthcare Clinic in Poznan, Poznan, Poland; ^3^ Chair of Ophthalmology and Optometry, Poznan University of Medical Sciences, Poznan, Poland; ^4^ Chair and Department of Inorganic and Analytical Chemistry, Poznan University of Medical Sciences, Poznan, Poland; ^5^ Chair and Department of Genetics and Pharmaceutical Microbiology, Poznan University of Medical Sciences, Poznan, Poland; ^6^ Department of Genetics and Animal Breeding, Poznan University of Life Sciences, Poznan, Poland; ^7^ Department of Medical Genetics, Medical University of Warsaw, Warsaw, Poland

**Keywords:** post-laser vision correction ectasia, post-SMILE ectasia, post-LASIK ectasia, keratoconus, corneal epithelium, transcriptomics, proteomics

## Abstract

**Introduction:**

Post-laser vision correction (post-LVC) ectasia is a serious complication that is observed in 0.033%–0.66% of corneal refractive surgeries. Similar to keratoconus (KTCN), post-LVC ectasia is classified under the category of “ectatic diseases.” We hypothesize that although the mechanistic aspects of post-LVC ectasia and KTCN are distinct, there are notable similarities in the epithelial responses, including shared molecular features.

**Methods:**

A total of 11 post-LVC ectasia, 8 mild myopia (controls), and 28 KTCN patients were included in a retrospective multiomics case–control study. The corneal epithelium (CE) samples obtained from the subjects were separated into different *topographic regions* (*TRs: central, middle,* and *peripheral*), and a total of 159 experimental samples were subjected to transcriptome (RNA-Seq) and proteome (MALDI-TOF/TOF MS/MS) profiling. The results were then verified/validated using reverse transcription quantitative polymerase chain reaction, immunofluorescence staining, and confocal microscopy in the extended sample set (n = 21).

**Results:**

The residual stromal bed, stromal ablation depth, and percent tissue altered indices were found to best predict the risk of post-LVC ectasia. From comparisons of post-LVC ectasia and KTCN, interferon-alpha and interferon-gamma hallmarks were found to be downregulated in the *central* and *middle TRs* of the CE of patients with post-LVC ectasia. Downregulation of *CADPS* gene expression was confirmed in all three *TRs* in the extended CE sample set. Cytoplasmic localizations of the CIITA and TBC1D4 proteins, which are the candidate post-LVC ectasia-specific biomarkers, were demonstrated in the CE samples.

**Discussion:**

The assessment of *CADPS, CPT1B, CIITA*, and *TBC1D4* gene expressions could enhance the risk estimation of ectasia in patients. Apart from differences in the transcription and inflammation processes, the CE of patients with post-LVC ectasia exhibits molecular features similar to KTCN.

## 1 Introduction

The earliest reports regarding serious complications in the form of ectasia development following laser vision correction (LVC) procedures were published in 1998 (Seiler et al., 1998; Seiler and Quurke, 1998). Since then, complications after these surgeries have been reported in 0.033% (Bohac et al., 2018) to 0.66% (Pallikaris et al., 2001) of patients. Such ectasia occurs mostly after laser-assisted *in situ* keratomileusis (LASIK) (Pallikaris et al., 2001); however, cases of ectasia after small incision lenticule extraction (SMILE) and photorefractive keratectomy (PRK) have also been reported (Dawson et al., 2008). Similar to keratoconus (KTCN, ICD10: H18.6), post-LVC ectasia (ICD10: H18.71) is classified under the category of “ectatic diseases” (Gomes et al., 2015). Post-LVC ectasia can develop months or years after refractive surgery. Diagnosis of this disease is commonly based on Scheimpflug imaging and optical coherence tomography evaluation. From an ophthalmological perspective, post-LVC ectasia is characterized by progressive corneal steepening and thinning either centrally or inferiorly as in KTCN, leading to severe progressive and irregular astigmatism as well as deterioration of both uncorrected and corrected visual acuity (Bohac et al., 2018; Twa et al., 2004). In contrast to the KTCN cornea, the cornea with post-LVC ectasia tends to show less-severe changes in its biomechanical properties (represented by the stiffness parameter at the first applanation), suggesting greater resistance to deformation (Zhao et al., 2020). However, corneas with post-LASIK ectasia exhibit surface symmetricity and regularity that are worse than those of KTCN corneas (Chu et al., 2018). The management of post-LVC ectasia is similar to that of KTCN and involves the use of glasses, soft contact lenses, rigid gas-permeable contact lenses, and intacs (Abad, 2008). As a treatment method for progression, the corneal cross-linking (CXL) procedure has been successfully used to strengthen the biomechanical properties of damaged corneas. In instances where all other options have been exhausted, corneal transplants are considered (Roberts and Dupps, 2014; Tong et al., 2017).

Unrecognized KTCN is a well-known risk factor for the development of corneal ectasia in patients after laser cornea refractive surgery (Randleman et al., 2003). However, primary KTCN and post-LVC ectasia should be distinguished from each other. The risk factors of post-LVC ectasia reported previously include lower age (<30 years old), family history of KTCN, topographic and tomographic abnormalities of the cornea (resembling *forme fruste* KTCN), thinner preoperative corneas (<500 µm), thinner residual stromal beds (<300 µm), excessive stromal ablation (>100 µm), high percentage of tissue altered (>40%), and high myopia (Jin et al., 2020; Randleman et al., 2008a, 2008b). The exact pathophysiology of post-LVC ectasia remains unknown. The histological abnormalities of the cornea are similar to those of primary KTCN, including thinning of the residual bed, thinning of the collagen lamellae, and decreased number of lamellae in the residual stromal bed (Dawson et al., 2008; Tong et al., 2017). Moreover, abnormal hemidesmosomes, irregularly arranged collagen fibers, and traces of wound healing (scarring) have been reported (Akhtar et al., 2014). The characteristic profile of corneal epithelium (CE) with central thinning and surrounding thickening (called *doughnut* pattern) described for KTCN epithelia (Jaskiewicz et al., 2023a) is also observable in patients with post-LVC ectasia (Rocha et al., 2013). It should be noted that some histological features, including changes in basal epithelial cells as well as anterior and posterior keratocytes, have been reported as differentiating features between post-LVC ectasia and KTCN (Alvani et al., 2020). Additionally, the presence of Bowman’s layer breaks and more severe collagen disorganization are attributed to KTCN (Santodomingo-Rubido et al., 2022).

Information regarding the molecular findings of post-LVC ectasia is scarce. There is only one report in literature that suggests inflammatory responses in corneas based on the increased density of corneal dendritic cells and elevated levels of cytokines/chemokines in the tears (Pahuja et al., 2017). Herein, for the first time in the research, we present the transcriptomic and proteomic profiles of CE. To date, there are no available records of transcriptomic (RNA sequencing) and proteomic (mass spectrometry) high-throughput data that have been evaluated together with clinical and morphological data for post-LVC ectasia. As the physiological mechanisms and molecular biomarkers of post-LVC ectasia remain unknown, we applied a multiomics approach to characterize the CE samples of patients diagnosed with post-LVC ectasia; then, we compared the obtained molecular data with the findings for patients with KTCN and control individuals undergoing PRK procedures. We hypothesize that although the mechanistic aspects of post-LVC ectasia and KTCN are distinct, there are notable similarities in the epithelial responses, including the shared molecular features.

## 2 Materials and methods

### 2.1 Ophthalmic examination and criteria for patient inclusion/exclusion

The study protocol was approved by the Bioethics Committee at Poznan University of Medical Sciences, Poznan, Poland (resolution no. 755/19, date: 19th June 2019). The possible consequences of the study were explained to the participants, and informed consent was obtained from all participants, in accordance with the guidelines of the Declaration of Helsinki. Our single-center (Optegra Eye Healthcare Clinic in Poznan, Poland) retrospective case–control study was conducted from October 2019 to May 2024.

Three subgroups of patients were included in this study, namely, post-LVC ectasia, KTCN, and control individuals (non-ectasia with mild myopia). Each individual underwent a complete ophthalmological examination, including corneal tomography and epithelial thickness mapping, as described in Supplementary Material S1.1. The inclusion criteria for the post-LVC ectasia patients were as follows: male and female patients aged over 18 years, with confirmed medical history of corneal refractive surgery, progressive inferior corneal steepening, progressive myopia and/or progressive astigmatism and/or inability to determine uncorrected visual acuity, and frequent inability to determine best-corrected visual acuity (Randleman et al., 2003). The exclusion criteria included history of corneal ectasia in the patient’s medical records, KTCN diagnosed in a family member, and abnormal topography/tomography before primary refractive surgery. The inclusion criteria for the control individuals and patients with KTCN were consistent with our previously established criteria (Czugala et al., 2012; Kabza et al., 2017), as described in Supplementary Material S1.2. The exclusion criteria for all study subgroups that were compatible with our previously established conditions (Jaskiewicz et al., 2023a) were as follows: presence of genetic disease, corneal scarring, posthydrops, and corneal thickness <400 µm (measured during the irradiation step in the CXL procedure). JASP Software was used for statistical analyses of the clinical parameters, as described in Supplementary Material S1.3. As the incidence rate of post-LVC ectasia is low, we did not determine the sample size with an *a priori* power analysis. For all significant results, the effect size metrics were verified (Cohen’s d > 0.5).

### 2.2 CXL and LVC procedures

CXL was performed in patients with post-LVC ectasia and KTCN as per the accelerated epi-off CXL protocol (12 min), while PRK was performed as a refractive error correction procedure in individuals with mild myopia (control), as described previously (Jaskiewicz et al., 2023a).

SMILE and LASIK were the primary types of LVC procedures performed in patients who were later found to have post-LVC ectasia. The SMILE procedure was performed using a femtosecond laser system to create a side incision, and a round-tipped spatula was used to dissect the lenticule from the surrounding stromal tissue. The lenticule was then carefully extracted through the same incision using microforceps. The LASIK/femto-LASIK procedure involved flap creation using either a microkeratome or femtosecond laser. After lifting the flap, stromal photoablation was carried out with an excimer laser system. Following ablation, the flap was repositioned and allowed to adhere naturally without sutures. All surgeries were conducted under topical anesthesia (Kim et al., 2019).

### 2.3 Material collection and sample preparation

The material collection and sample preparation were performed as described previously (Jaskiewicz et al., 2023a). Briefly, stamps were made toward the nose and eyebrow on the CE before excision during the CXL/PRK procedures. The obtained tissues were submerged in an RNA stabilization solution (RNAlater, Qiagen, Hilden, Germany) immediately after excision and stored at −80°C. The designation of the *topographic regions* (*TRs: central, middle*, and *peripheral*) of the CE was conducted simultaneously by the operating surgeon and researcher processing the material based on regional variations in the corneal and epithelial thicknesses assessed by corneal tomography (Pentacam, Oculus Optikgeräte GmbH) and epithelial thickness mapping (MS-39, CSO, Italy), in accordance with the methodology described in our previous study (Jaskiewicz et al., 2023a).

### 2.4 RNA and protein extraction

The total RNA, DNA, and proteins were extracted from CE samples using the RNA/DNA/Protein Purification Plus Micro Kit (Norgen Biotek, Thorold, ON, Canada), as described previously (Jaskiewicz et al., 2023a). During extraction, the DNase treatment for degradation of contaminating DNA after RNA isolation was performed using DNase I RNase-free (EURx, Gdansk, Poland). The quality of the purified RNA samples was assessed using an RNA 6000 Nano Kit (Agilent Technologies, Waldbronn, Germany), while the quantity was measured with NanoDrop (Spectrophotometer ND-1000, NanoDrop Technologies, Inc.). The quality and quantity of the purified protein samples were assessed using the Protein 230 Kit (Agilent Technologies).

### 2.5 Total RNA library preparation, sequencing, and data analyses

The total RNA libraries were prepared according to a previously established protocol (Jaskiewicz et al., 2023a) using TruSeq Stranded Total RNA Library Prep Gold (Illumina, San Diego, CA, United States), as described in Supplementary Material S1.4. A 100-bp paired-end sequencing run was performed on a NovaSeq 6000 platform (Illumina), and the CE samples were sequenced with an average coverage of 113 million read pairs per sample. Bioinformatics analyses were then performed according to a previously established protocol (Jaskiewicz et al., 2023a), as described in Supplementary Material S1.4. Briefly, the reads were trimmed using the BBDuk2 program from the BBTools suite (http://jgi.doe.gov/data-and-tools/bbtools/). Kallisto assisted by GENCODE 34 (Ensembl 100) annotations was used to estimate the expression values of the transcripts. The differential expression analysis was conducted using the *limma* package (Law et al., 2016; Ritchie et al., 2015). The genes were considered to be differentially expressed based on the following cutoffs: 0.05 for false discovery rate (FDR) and 1.5 for the absolute value of log_2_-transformed fold change (log_2_FC). To create the heatmaps, the data were normalized using library size factors and log transformation via the *scuttle* package (McCarthy et al., 2017) to ensure consistency and comparability. Following normalization, we scaled the log-transformed counts to standardize the data; this involved adjusting the data using the “scale” function in R so that each gene had a mean of zero and standard deviation of one (R Core Team, 2024). The heatmaps were then generated using the *pheatmap* package (Kolde, 2019) along with hierarchical clustering based on the Euclidean distance.

Uniform manifold approximation and projection (UMAP) embeddings were calculated using the *scran* (Lun et al., 2016) and *scater* (McCarthy et al., 2017) packages. Then, principal component analysis was performed, followed by assessment and correction of the experimental batch effects by including the next-generation sequencing (NGS) library preparation date (six levels) as a covariate. Batch correction was then conducted using the limma and fastMNN() method (Ritchie et al., 2015; Law et al., 2016). The differentially expressed genes (DEGs) were analyzed using the following comparison schemes: expression levels in the *central, middle, and peripheral TRs* of the CE were compared separately between the post-LVC ectasia and control samples (e.g., *central TR* of patients with post-LVC ectasia vs. *central TR* of controls, with similar comparisons for the *middle* and *peripheral TRs*); the comparisons were further stratified by sex (e.g., *central TR* of female patients with post-LVC ectasia vs. *central TR* of female controls). Given the large number of comparisons, we applied the Benjamini–Hochberg FDR correction (Benjamini and Hochberg, 1995) to adjust the *p*-values and control for multiple testing.

### 2.6 Verification and validation of RNA-seq data

Verification of the RNA-seq data was performed as described in Supplementary Material S1.5. Briefly, the RNA samples were reverse transcribed to cDNA using the Maxima First-Strand cDNA Synthesis Kit for RT-qPCR (Thermo Fisher Scientific Inc., Lithuania), and the expression levels of the selected genes (including three reference transcripts: *UBC*, *LDHA*, and *RPL4*) were assessed using the HOT FIREPol EvaGreen qPCR Mix Plus (Solis BioDyne OÜ, Estonia) through the CFX96 Touch Real-Time PCR Detection System (Bio-Rad Laboratories, Hercules, CA, USA). The primer sequences and annealing temperatures of the selected genes are shown in Supplementary Table S1. In addition, the results were further validated using the extended sample set of CEs derived from seven eyes of five patients with post-LVC ectasia (21 experimental samples) (Supplementary Material S1.5).

### 2.7 MALDI-TOF/TOF MS/MS protein–peptide profiling

We performed tandem matrix-assisted laser desorption/ionization time of flight/time of flight mass spectrometry (MALDI-TOF/TOF MS) analysis according to a previously established protocol (Jaskiewicz et al., 2023a; Matuszewska et al., 2021), as described in Supplementary Material S1.6. These were conducted using an UltrafleXtreme (Bruker Daltonics) mass spectrometer operated in the reflectron mode in the mass-to-charge ratio (m/z) range of 700–3,500. Then, proteomic identification was conducted using the SwissProt protein sequence database. Statistical analyses were performed using JASP Software (JASP Team, 2022), as described in Supplementary Material S1.6.

### 2.8 Immunofluorescence (IF) staining of the CE samples

The CE samples were fixed using methanol and incubated with primary (CIITA antibody: #PA5-21031, Invitrogen; TBC1D4 antibody: #TA502707S, OriGene; overnight incubation at 4°C) and secondary antibodies (Alexa Fluor® Plus 488 donkey anti-rabbit IgG: #A32790, Invitrogen; Alexa Fluor® Plus 405 donkey anti-mouse IgG: #A48257, Invitrogen), as described in Supplementary Material S1.7. The samples were analyzed under a Leica STELLARIS confocal microscope (Leica Microsystems GmbH), and the fluorescence signals for TBC1D4 (blue) and CIITA (green) were quantified using Fiji (ImageJ) (Schindelin et al., 2012). The values were normalized to the red signal from propidium iodide (PI) to control for staining and imaging variabilities.

## 3 Results

### 3.1 Characteristics of patients and biological samples

The 11 patients with post-LVC ectasia (seven male and four female; n = 11 eyes) undergoing the CXL procedure and eight control individuals (four male and four female; mild myopia; n = 14 eyes) undergoing refractive error correction were ascertained. The clinical characteristics of the patients with post-LVC ectasia and the control group are summarized in Table 1 along with information on patients with KTCN (26 male and two female; n = 28 eyes). The medical examination details for each individual are shown in Supplementary Tables S2A–S2C. The extended sample set used for study validation contained information from seven eyes of five patients with post-LVC ectasia (three female and two male), as presented in Supplementary Tables S3A, S3B.

**TABLE 1:** Clinical characteristics of examined patients with post-LVC ectasia, control individuals, and patients with KTCN, and their eyes subjected to the surgery. The p-values of Mann-Whitney test are indicated, for all significant results, the effect size metrics were verified (Cohen's d > 0.5).

Clinical Characteristics	Post-LVC ectasia (*n*=11)		Controls (*n*=8/14)^a^		KTCN (n=28)		p-value post-LVC ectasia vs controls	p-value post-LVC ectasia vs KTCN
	x ± SD	M	x ± SD	M	x ± SD	M		
Age at examination	36.73±7.71	39.0	28.25±7.01	25.0	26.71±6.42	25.0	**0.047**	**0.001**
Age at ectasia diagnosis	30.88±7.34	30.0	n/a	n/a	24.67±5.72	23.0	n/a	**0.015**
K1 [D]	41.29±3.98	41.1	43.37±1.77	43.2	46.51±4.54	45.3	0.107	**0.001**
K2 [D]	43.98±5.51	42.8	44.54±1.56	44.6	49.71±5.61	48.3	0.283	**0.002**
Kmax [D]	49.50±6.48	49.0	44.89±1.92	44.9	58.66±8.51	57.1	**0.033**	**0.001**
Anterior elevation [μm]	22.18±13.40	18.0	1.43±0.94	1.0	31.25±11.81	28.5	**<0.001**	**0.024** ^b^
Posterior elevation [μm]	44.00±26.83	37.0	2.86±3.35	2.0	66.68±25.62	58.5	**<0.001**	**0.008**
Diff BFS [µm]	26.82±22.06	19.0	3.21±0.85	3.0	45.61±21.64	40.0	**<0.001**	**0.006**
TCT [µm]	428.82±67.00	419.0	527.71±33.85	522.6	445.32±45.45	449.0	**0.005**	0.382
TET (automatic, in range 0.0-7.0mm) [μm]	44.36±4.37	43.0	45.50±2.98	45.5	41.25±4.74	45.0	0.455	0.077
average thickness of *central TR* [μm]	45.82±4.73	44.0	51.71±3.38	52.0	43.25±4.69	44.0	**0.011**	0.027
Average thickness of *middle TR* [μm]	59.36±3.44	60.0	49.07±3.52	48.5	56.32±4.65	55.0	**<0.001**	**0.029** ^b^
Average thickness of *peripheral TR* [μm]	49.46±4.03	49.0	46.93±3.22	47.5	48.89±3.68	48.0	0.210	0.626

^a^ In the study, biological samples were included from eight patients and 14 control individuals (we obtained material from both treated eyes from six individuals); the data from ophthalmological examination concern the biological samples.

^b^ This result did not meet the criterion of Cohen’s d metric.

Abbreviations: x, average; SD, standard deviation; M, median; K1, flat keratometric reading; K2, steep keratometric reading; Kmax, maximum simulated keratometry; diff BFS, difference in best fit sphere; TCT, thinnest corneal thickness; TET, thinnest epithelial thickness; TR, topographic region.

**FIGURE 1:**
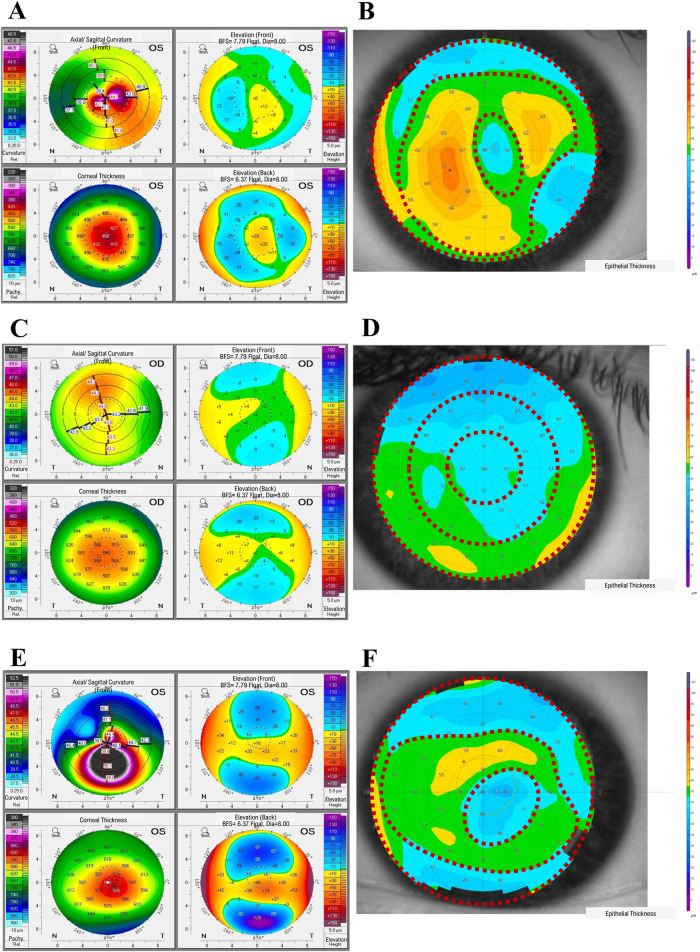
Representative results from the ophthalmological examinations of **(A, B)** a patient with post-laser vision correction (post-LVC) ectasia, **(C, D)** control individual, and **(E, F)** patient with keratoconus (KTCN), indicating the corneal *topographic regions (TRs)*. **(A, C, E)** The corneal tomography results were obtained with a Pentacam (Oculus Optikgeraete GmbH), whereas **(B, D, F)** the epithelial thickness maps were acquired using the MS-39 device (Costruzione Strumenti Oftalmici). The epithelial thickness maps show three distinct *TRs (1^st^, central; 2^nd^, middle; and 3^rd^, peripheral*). For the exact values of the parameters evaluated, please refer to Supplementary Tables S2A, S2B.

**FIGURE 2:**
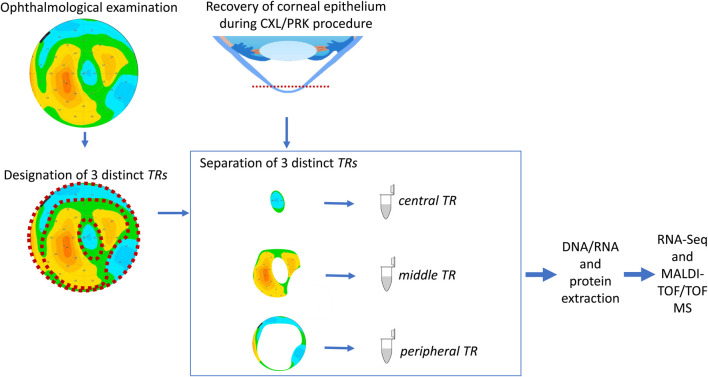
Study workflow. The procedures for designating the particular *TRs* of the corneal epithelium (CE) are compatible with our previously developed experimental model (Jaskiewicz et al., 2023a). Before cutting the CE, the individuals underwent ophthalmic examinations, and the three *TRs* were assessed based on the epithelium thickness values. After separation of the *TRs*, the DNA, RNA, and protein extractions were performed for high-throughput analyses.

In our full study group, eight patients (16 eyes) developed corneal ectasia after SMILE and six patients (12 eyes) developed post-LASIK ectasia; information regarding the type of laser correction procedure performed was unavailable for two patients. No preoperative topographic and tomographic abnormalities of the cornea were recorded for these patients. A residual stromal bed <300 µm was found in 15/22 eyes, while stromal ablation >100 µm was found in 16/24 eyes and percent tissue altered index exceeded 40% in 16/24 of the examined eyes for which sufficient clinical data were available. Preoperative high myopia was found in 6/28 eyes, while low central corneal thickness values (<500 µm) were observed in 6/28 eyes (none of them were high-myopic). We observed a characteristic *doughnut* pattern (thin cone center surrounded by thickened annulus) on the epithelial thickness map of each patient with ectasia; an example of the corneal tomography and corneal thickness mapping of an examined individual are presented in Figure 1. The *middle TRs* of the CE of patients with post-LVC ectasia were thicker than those of patients with KTCN (*p* = 0.029). Upon acknowledging these findings, we implemented a previously designed workflow (Jaskiewicz et al., 2023a) to define and separate the three *TRs* (Figure 2). From each of the *central, middle*, and *peripheral TRs* of the CE samples, we simultaneously extracted RNA and proteins, which resulted in a total of 159 experimental samples (three *TRs* from each of the 53 eyes, including 11 eyes from 11 patients with post-LVC ectasia, 14 eyes from eight controls, and 28 eyes from 28 patients with KTCN) for transcriptomic and proteomic profiling assessments. The results of the quality and quantity control of the RNA samples as well as a summary of the RNA-seq reads are presented in Supplementary Table S4. In addition to the disease status, we evaluated the sex and age differences among the post-LVC patients as biological variables (Supplementary Figures S1, S2), while the NGS library preparation date was considered a technical variable that could potentially result in batch effects (Supplementary Figure S3).

### 3.2 Post-LVC ectasia-specific gene expressions

**FIGURE 3:**
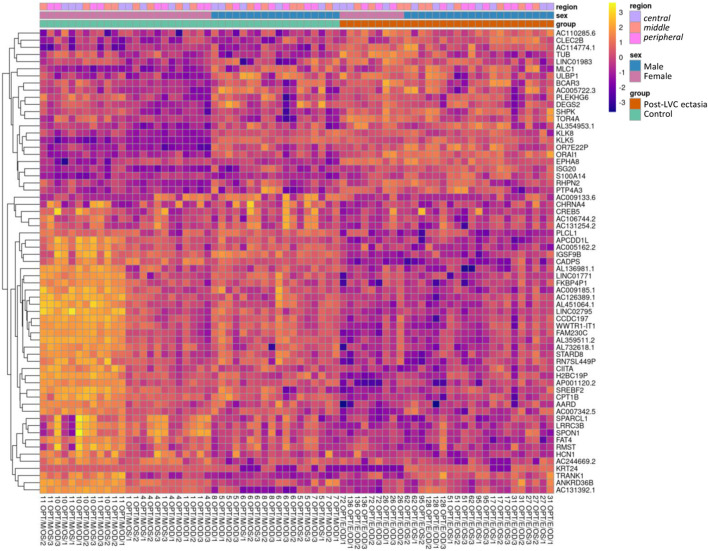
Heatmap of the RNA-seq transcriptome analysis for 65 selected genes from the differential expression analysis for post-LVC ectasia. The genes (in rows, *n* = 65) were hierarchically clustered based on the Euclidean distance. The colors correspond to data that were normalized using library size factors, followed by log transformation and scaling (each gene has a mean of zero and standard deviation of one), where the dark violet to bright yellow color gradient denotes low to high expressions. The genes presented here correspond to the most differentially expressed genes in post-LVC ectasia (Supplementary Table S7) including our gene of interest (*CIITA*). The samples (in columns, *n* = 72) correspond to the list from Supplementary Table S4 (nos. 1–72). Above the heatmap are the control samples shown in green squares and post-LVC ectasia samples shown in red squares, while the sexes of the patients are indicated in blue color for male and pink color for female patients; the *central TR* is annotated using mauve color, *middle TR* corresponds to peach color, and *peripheral TR* is shown in fuchsia color.

Upon comparing the particular *TRs* of the CE samples, the differential analysis showed significant DEGs between post-LVC ectasia and controls, including a total of 1,296 DEGs comprising 677 upregulated (486 protein-coding) and 619 downregulated (240 protein-coding) genes (Supplementary Tables S5, S6). From each comparison, based on the absolute log_2_FC values, we selected the top 15 DEGs to generate a heatmap (Figure 3; Supplementary Figure S4; Supplementary Table S7) by recognizing that both disease and sex impacted the gene expression profiles in post-LVC ectasia. The hallmark gene set analysis revealed some features like epithelial-to-mesenchymal transitions and apical junctions only in male patients with post-LVC ectasia (Supplementary Figure S5; Supplementary Table S8), whereas the pathway enrichment analysis showed translation, eukaryotic translation initiation, and eukaryotic translation elongation as the most upregulated pathways in a non-sex-dependent manner (Supplementary Table S9).

### 3.3 Post-LVC ectasia mimicking KTCN

**FIGURE 4:**
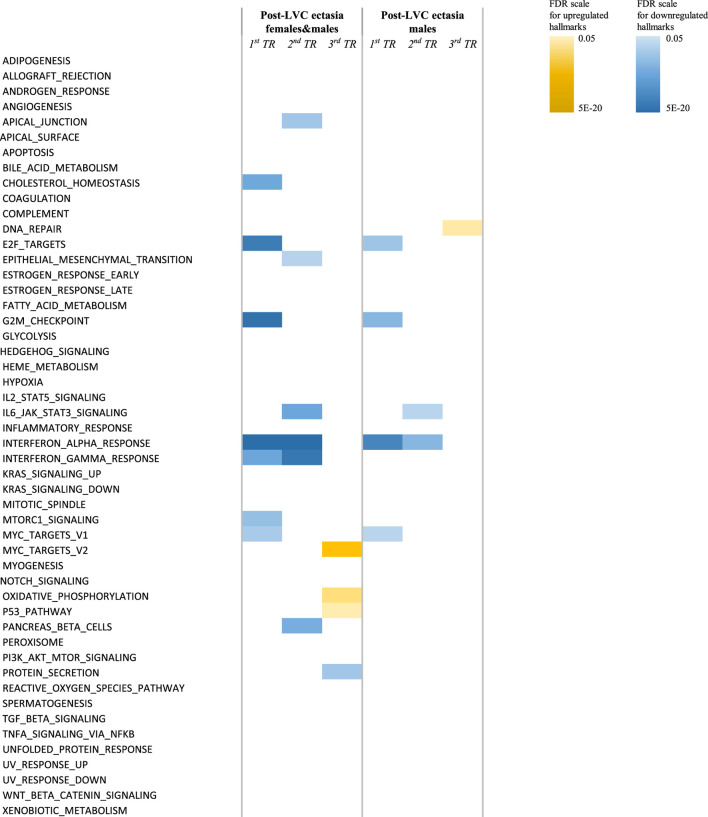
Hallmark pathways differentially enriched in the *TRs* of the CE of patients with post-LVC ectasia compared to those of patients with KTCN. Enrichment analysis was performed under different settings depending on the sex of the individuals. The color scales represent the false discovery rate (FDR) values for each upregulated and downregulated hallmark. For detailed information on the values, please see Supplementary Table S11.

No protein-coding genes were revealed in the differential analysis between post-LVC ectasia and KTCN. However, other classes/biotypes of transcripts, miRNAs (MIR3132), lncRNAs (AC120498.2), snRNAs (RNU1-142P), and processed pseudogenes (AL161713.1, AC004386.2, MRPS16P2, and TECRP1) were recognized (Supplementary Table S10). Based on these DEGs as well as the most common DEGs from the comparison of post-LVC ectasia vs. controls (Supplementary Table S7), the heatmap shown in Supplementary Figure S6 was generated. The differences between post-LVC ectasia and KTCN were recognized in the hallmark gene set analysis, where the interferon-alpha and interferon-gamma responses were downregulated in the *central* and *middle TRs* of the CE of patients with post-LVC ectasia compared to KTCN (Figure 4; Supplementary Table S11). Pathway enrichment analysis showed that interferon signaling was also downregulated in these *TRs* of the CE (Supplementary Table S12). Moreover, the pathways for keratinization and antigen processing cross-presentation were downregulated in the *middle TR* of the CE while the DNA methylation pathway was upregulated in the *peripheral TR* of the CE between patients with post-LVC ectasia and KTCN (Supplementary Table S12).

### 3.4 Proteome profiling

**FIGURE 5:**
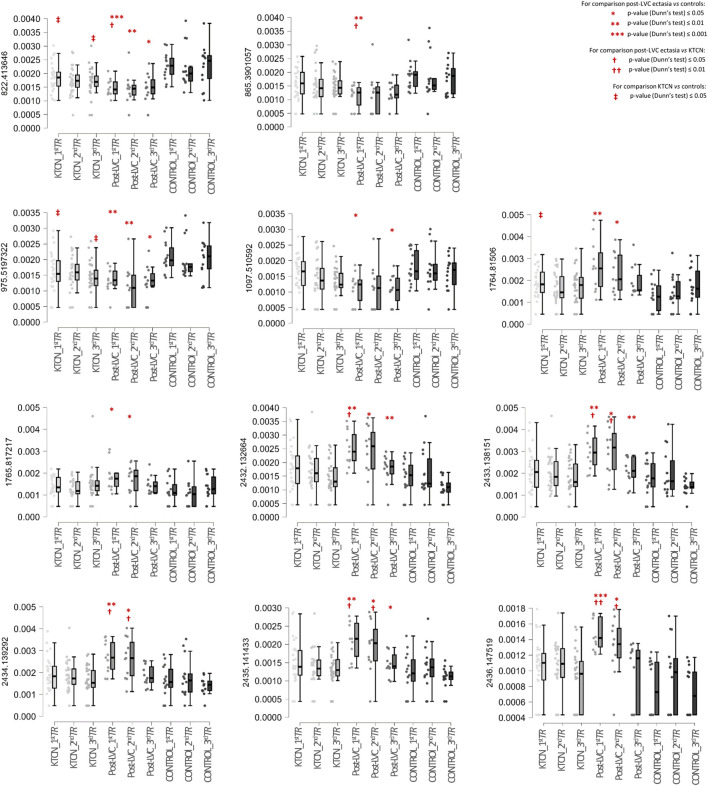
Box plots of selected protein peaks from the proteome assessment could be used to distinguish particular *TRs* of the CE in a multigroup analysis including patients with post-LVC ectasia, patients with KTCN, and controls. The protein peaks were identified as fragments of the proteins TBC1D4 (m/z peaks at 2432.132664, 2433.138151, 2434.139292, 2435.141433, and 2436.147519), DOCK8 (m/z peak at 1097.510592), ZFP109 (m/z peak at 865.3901057), KRT12 (m/peak at 822.413646), ABCA13 (m/z peak at 975.5197322), GAPDH (m/z peak at 1764.81506), SDC1 (m/z peak at 1765.817217), and ACTB (m/z peak at 3185.612433). On the left side of each plot, the m/z value for that particular protein peak is presented. The *x*-axis shows the subgroups of samples in the following order: *central TR* of KTCN patients (KTCN_*1* ^*st*^ *TR*), *middle TR* of KTCN patients (KTCN_*2* ^*nd*^ *TR*), *peripheral TR* of KTCN patients (KTCN_*3* ^*rd*^ *TR*), *central TR* of post-LVC ectasia patients (post-LVC_*1* ^*st*^ *TR*), *middle TR* of post-LVC ectasia patients (post-LVC_*2* ^*nd*^ *TR*), *peripheral TR* of post-LVC ectasia patients (post-LVC_*3* ^*rd*^ *TR*), *central TR* of control individuals (control_*1* ^*st*^ *TR*), *middle TR* of control individuals (control_*2* ^*nd*^ *TR*), and *peripheral TR* of control individuals (control_*3* ^*rd*^ *TR*). The *y*-axis shows the intensities of the protein peaks. The statistically significant results (based on Dunn’s *post hoc* test) are marked. For detailed information on the fragment sequences, protein names, *p*-values from the Kruskal–Wallis test, and *p*-values from Dunn’s *post hoc* test, please see Supplementary Table S16.

In total, 1,484 different peaks (protein/peptide fragments) were detected in all the CE samples during proteome profiling. We identified 47 unique protein fragments as discriminative for post-LVC ectasia (compared to controls; *p*-value ≤ 0.05; Supplementary Tables S13, S14), including CWC25, DOCK8, GOLGA4, KRT12, USP31, WDR62, ZFP106, ABCA13, FAT3, and ACTB, which were depleted in specific *TRs* of the CE irrespective of sex, whereas TBC1D4 and ACTB were enriched in a non-sex-dependent manner. Comparing post-LVC ectasia and KTCN showed that the fragments of eight proteins (TBC1D4, ACTB, GNAB, GAPDH, SDC1, FLRT1, GAPVD1, and KRT5) were enriched in post-LVC ectasia in particular *TRs* of the CE irrespective of sex (*p*-value ≤ 0.05; Supplementary Table S15). In the multigroup analysis (post-LVC ectasia vs. controls vs. KTCN), fragments from 20, 9, and 27 proteins were found to be differentiated in the *central, middle*, and *peripheral TRs* of the CE, respectively (*p*-value ≤ 0.05; Table 2; Supplementary Table S16). Next, in the *post hoc* analysis for the *central TR*, five different fragments of the TBC1D4 protein (with m/z peaks at 2432.132664, 2433.138151, 2434.139292, 2435.141433, and 2436.147519), one fragment of DOCK8 (with m/z peak at 1097.510592), and one fragment of ZFP109 (with m/z peak at 865.3901057) were revealed to simultaneously differentiate post-LVC ectasia from both the control and KTCN groups. Correspondingly, for the *middle TR*, one fragment each of KRT12 (with m/z peak at 822.413646), ABCA13 (with m/z peak at 975.5197322), GAPDH (with m/z peak at 1764.81506), SDC1 (with m/z peak at 1765.817217), and ACTB (with m/z peak at 3185.612433) as well as five fragments of the TBC1D4 protein (with the same peaks as those for the *central TR*) were identified (Figure 5).

**TABLE 2:** Proteins found to be discriminative for particular *TRs* of CE of patients with post-LVC ectasia in comparison to corresponding *TRs* of CE of patients with KTCN. Analysis was performed in three settings depending on the sex of the individuals included. All m/z values, fragment sequence, protein name, and p-values of the Mann-Whitney test are presented in Supplementary Table S16.

Directon of change	Post-LVC ectasia females&males vs KTCN females&males			Post-LVC ectasia males vs KTCN males		
	*1* ^*st*^ *TR*	*2* ^*nd*^ *TR*	*3* ^*rd*^ *TR*	*1* ^*st*^ *TR*	*2* ^*nd*^ *TR*	*3* ^*rd*^ *TR*
UP	TBC1D4^a^	ACTB^a^	ACTB	PMS2CL	ACTB^a^	FLRT1^a^
	—	GANAB^a^	FAM102B	TBC1D4^a^	ENO1	GAPVD1^a^
	—	GAPDH^a^	FLRT1^a^	—	GANAB^a^	KRT5^a^
	—	KRT76	GAPVD1^a^	—	GAPDH*	MRGBP
	—	SDC1^a^	KRT5^a^	—	NUCB1	—
	—	TBC1D4	KRT76	—	SDC1*	—
	—	—	—	—	TBC1D4	—
DOWN	DOCK8	ABCA13	LRRC20	—	—	—
	KRT12	FGF8	—	—	—	—
	ZFP106	KRT12	—	—	—	—

^a^ Indicates proteins revealed to be distinguishable for particular TRs of the CE irrespective of sex in each comparison.

Abbreviations: UP, upregulated proteins; DOWN, downregulated proteins; TR, topographic region of the corneal epithelium, 1^st^TR, central topographic region, 2^nd^TR, middle topographic region, 3^rd^TR, peripheral topographic region.

### 3.5 Validation of the study results

**FIGURE 6:**
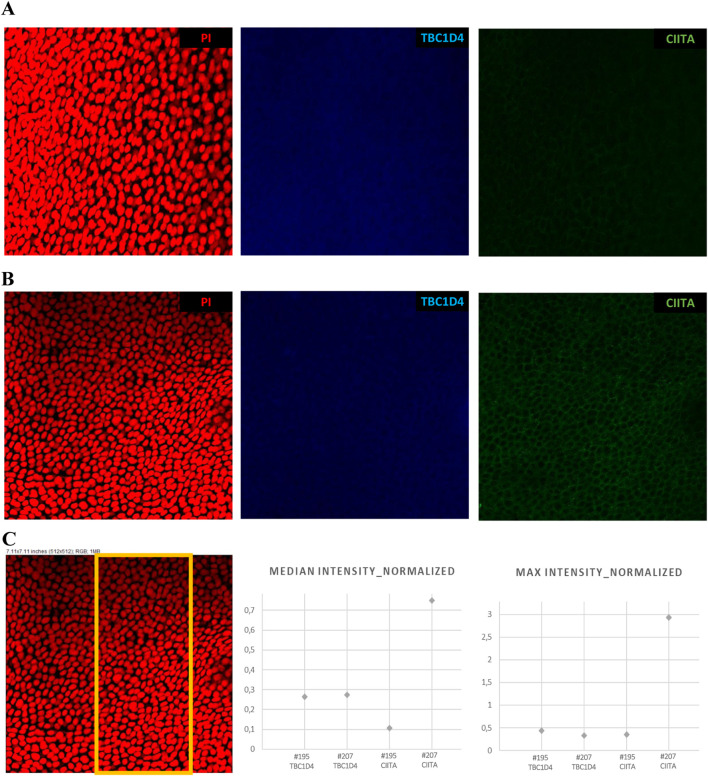
Spatial expression profiles of CIITA and TBC1D4 proteins in the CE samples of patients with post-LVC ectasia ((**A**) #195 OPT/E/OD) and KTCN ((**B**) #207 OPT/KTCN/OS) using 3D confocal imaging. **(C)** Representative region of interest selected for quantitative analysis, with the corresponding plots showing the median and maximum fluorescence intensities of TBC1D4 (blue) and CIITA (green), normalized to the nuclear signal obtained from propidium iodide (PI, red) staining. The cytoplasmic localization of CIITA and cytoplasmic (also likely nuclear) localization of TBC1D4 are visible. The Z-stack images were acquired with a Leica STELLARIS confocal microscope using the HC PL APO CS2 20×/0.75 DRY objective, digital zoom = 2.0, and “max” intensity option of LAS X 4.6.1 software. All microscope settings are described in the metadata file shared in Mendeley Data Repository (DOI: 10.17632/p656wtzjv8.1).

We performed RT-qPCR experiments to verify the RNA-seq data and assessed the expressions of *CADPS*, *CPT1B*, *S100A14*, *KLK5*, *LDHA*, *RLP4*, and *UBC* genes in each of the 72 originally collected CE samples (Supplementary Table S1 shows the primer sequences and annealing temperatures; Supplementary Table S17 contains the obtained Ct values). A high positive correlation (Pearson’s r = 0.901, *p* = 0.006) was found between the RNA-seq and RT-qPCR data, as presented in Supplementary Figure S7 and Supplementary Table S18. To further confirm the study outcomes, five additional patients with post-LVC ectasia (total of seven eyes) who underwent the CXL procedure were recruited and examined, as shown in Supplementary Tables S3A, S3B. The downregulated expressions of two selected post-LVC ectasia-specific genes, *CADPS* and *CPT1B*, were positively validated in this extended sample set of patients in all three *TRs* (*p* < 0.001 for *central TR*, *p* = 0.07 for *middle TR*, and *p* < 0.001 for *peripheral TR*) and *middle TR* (*p* < 0.001), respectively (Supplementary Figure S8; Supplementary Table S19). Next, IF staining was performed to further assess the spatial expression of *CIITA*, which was downregulated at the RNA and protein levels in the CE samples of patients with post-LVC ectasia in this study. Furthermore, TBC1D4 protein that was enriched in all three *TRs* of the CE samples of patients with post-LVC ectasia in comparison to both controls and patients with KTCN was chosen to validate the study results. The cytoplasmic locations of CIITA and TBC1D4 in the CE were also revealed (Figure 6; Supplementary Table S20).

## 4 Discussion

Corneal ectasia encompasses both primary occurring and surgically induced progressive corneal thinning and protrusion (Garcia-Ferrer et al., 2019); therefore, it includes post-LVC ectasia, KTCN, and other conditions like pellucid marginal degeneration, keratoglobus, and wound ectasia after penetrating keratoplasty. Given the growing number of refractive surgeries performed in recent times (Ambrósio, 2019), it has become necessary to enhance ectasia risk assessments. Simultaneously, we investigated the molecular features as the pathophysiology of post-LVC ectasia remains unknown. In our study, there were no pronounced sex-based differences among patients with post-LVC ectasia; the study group comprised seven female and nine male patients, in contrast to the group of 28 patients with KTCN where only two women were examined. Some reports have suggested a slightly higher incidence of post-LVC ectasia in men (Randleman et al., 2008b; Moshirfar et al., 2021); however, a small sample size and selection criteria could have biased this conclusion. On the other hand, higher prevalence of KTCN in male patients has been repeatedly reported in medium- and large-scale studies (Jaskiewicz et al., 2023b; Fink et al., 2005; Wagner et al., 2007). Although the male sex is considered a risk factor for KTCN, there is no identified/published molecular basis for this phenomenon. Nevertheless, even as this aspect is unclear, the lack of male predominance and older age in our study are important distinguishing demographic factors for post-LVC ectasia in relation to KTCN. The statistically significant difference in age at examination between post-LVC ectasia and KTCN patients is attributed to the naturally late diagnosis of post-LVC ectasia as an effect of primary refractive surgery, as also reported by Rocha et al. (2013).

Importantly, we have not confirmed that the rate of ectasia after LASIK is higher than that after SMILE or PRK (Moshirfar et al., 2021). In our study, the eight patients with post-LVC ectasia had undergone the SMILE procedure in the past. We speculate that the previously reported relatively low rates of post-SMILE ectasia and low numbers of published cases are merely an effect of the novelty of this procedure; therefore, the findings may change in the near future. In two patients from our study group, ectasia symptoms manifested 3 months after the primary surgery, suggesting room for improvement in the qualification procedure for refractive surgery. On the other hand, more than 10 years had elapsed between the primary surgery and development of ectasia in four patients, emphasizing the need for continuous regular ophthalmological examinations after surgery. We did not confirm thinner preoperative central corneal thickness (<500 µm) and higher preoperative refractive error as a single risk factor of post-LVC ectasia, but the values of excessive stromal ablation (>100 µm), thinner residual stromal bed (<300 µm), and higher percent tissue altered index (>40%, calculated based on the central corneal thickness, flap thickness, and ablation depth) exceeded the reported borderline values (Randleman et al., 2003; Jin et al., 2020; Randleman et al., 2008a, 2008b) in approximately two-thirds of the examined eyes, for which sufficient clinical data were available.

Our study offers an insight into the post-LVC ectasia phenotype, where the CE shows multiple morphological and molecular alternations. Abnormalities of the CE in post-LASIK and post-PRK ectatic corneas, including hypoplasia (attributable to *central TR* in our study), occasional foci of the epithelial hyperplasia (present in the *middle TR* in our study), and abnormal basement membrane structures, suggesting ongoing remodeling have been reported previously (Dawson et al., 2008; Rocha et al., 2013; Maguen et al., 2007). In this study, we found central thinning with surrounding thickening in CE samples derived from patients with post-LVC ectasia, indicating a donut pattern of the same kind noted in KTCN (Jaskiewicz et al., 2023a; Reinstein et al., 2009). Previously, Rocha et al. (2013) found similar epithelial thickness profiles in corneas with post-LVC ectasia and KTCN; however, their analysis only concerned the central corneal epithelia (central 6-mm diameter). In the present study, this increased average thickness of the *middle TR* was observed in patients with post-LVC ectasia compared to both KTCN and controls. Khamar et al. (2019) used the results of epithelial imaging, anterior corneal surface, and Bowman’s layer, along with the outcomes of previous studies and suggested that there were higher degrees of epithelial and stromal changes in post-LVC ectasia eyes than KTCN, which is in line with our results regarding the thinnest corneal thickness and CE thickness in the *middle TR*.

To acknowledge the findings regarding the thickness profiles of the CE in patients with post-LVC ectasia, we evaluated the three areas of the cornea (*central, middle*, and *peripheral TRs*) described in our previous KTCN studies (Jaskiewicz et al., 2023a, 2023c) using high-throughput molecular techniques. We found that both disease status and sex impacted the gene expression profile in post-LVC ectasia. In the pathway enrichment analysis comparing post-LVC ectasia with controls, we found translation, eukaryotic translation initiation, and eukaryotic translation elongation as the most upregulated pathways. Aside from these alterations, upregulated levels of ACTB and GAPDH were found in proteome profiling. ACTB is involved in transcription and its regulation (Hu et al., 2004; Miralles and Visa, 2006), whereas GAPDH is involved in glycolysis, transcription, RNA transport, DNA replication, and apoptosis (Colell et al., 2009; Tristan et al., 2011), so the obtained results may imply enhanced activation of proliferation in the CE of patients with post-LVC ectasia. However, an enriched hallmark of apoptosis was also found in the studied samples; therefore, we may hypothesize apoptosis-induced proliferation in the CE of patients with post-LVC ectasia.

Including patients with KTCN in the study proved their added value for characterizing the molecular profile of post-LVC ectasia. To date, different approaches have been used to study CE samples and KTCN corneas, including high-throughput technologies and various omics levels (Jaskiewicz et al., 2023a; Kabza et al., 2017; Kabza et al., 2019; Khaled et al., 2018; Nowak-Malczewska et al., 2024; Shinde et al., 2019), whereas studies on post-LVC ectasia have been scarce. The differential analysis between post-LVC ectasia and KTCN revealed only seven genes, none of which encode proteins. In this study, we did not explore non-protein-coding transcripts in depth. Further assessment of the obtained experimental data showed more prominent, although limited, differences between post-LVC ectasia and KTCN in the hallmark gene set analysis. The interferon-alpha and interferon-gamma response hallmarks were found to be downregulated in the *central* and *middle TRs* of the CE of patients with post-LVC ectasia compared with KTCN but upregulated with respect to the controls. This result is of particular interest because we are aware of the inflammatory background in KTCN based on the pro-inflammatory transcriptomic and proteomic profiles of corneal and tear samples identified in recent years (Chaerkady et al., 2013; Gijs et al., 2023; Jun et al., 2011; Shetty et al., 2015). Moreover, the genetic aspects regarding immune system functioning have been demonstrated in KTCN research. First, we identified a KTCN-specific variant in the *IL1RN* gene (c.214 + 242C>T) in 2013 that encodes a protein modulating the effects of IL-1 (Nowak et al., 2013); presently, whole-genome sequencing (WGS) data allowed us to provide additional insights into this aspect in 2023, as we identified non-coding and coding variants in genes contributing to molecular pathways like antigen presentation and interferon-alpha/beta signaling (Jaskiewicz et al., 2023c).

To verify and validate the study outcomes, additional patients with post-LVC ectasia were recruited in this study, and we further confirmed that the expression of *CADPS* was the most dysregulated in the *central TR*. CADPS protein is involved in the exocytosis of vesicles filled with neurotransmitters and neuropeptides, so it is highly expressed in the nervous and endocrine systems (Cisternas et al., 2003; Speidel et al., 2003). Thus far, there are no published data concerning the cornea and this gene; however, it has been reported to promote metastasis in colorectal cancer via its influence on cell migration (Wu et al., 2019). Moreover, *CADPS* was observed to directly interact with *SDCBP* (Rolland et al., 2014), which is involved in cell adhesion as a link between the proteoglycan/matrix receptor syndecan-1 and cytoskeleton (Stepp et al., 2010; Stepp et al., 2002). Furthermore, *SDCBP*-null mice showed abnormalities in the wound-healing process for damaged mouse corneas (Stepp et al., 2010; Stepp et al., 2002). The findings regarding changes in the cytoskeleton formation and wound healing process were published in the KTCN studies (Jaskiewicz et al., 2023a; Kabza et al., 2017).

The reduced expression of *CPT1B* in the *middle TR* of patients with post-LVC ectasia was confirmed in the extended sample set. This enzyme is associated with mitochondrial oxidation of long-chain fatty acids (LCFAs) since it catalyzes the transfer of the acyl group of the LCFA–CoA conjugate onto carnitine (Bonnefont et al., 2004), which was found to protect against corneal stress activation (Flanagan et al., 2010) and reported to be upregulated in KTCN-derived keratocytes after conditioning with transforming growth factor-β (Sarker-Nag et al., 2016). Importantly, we did not observe any differences between post-LVC ectasia and KTCN with respect to the oxidative stress markers and metalloproteinases, which is in line with previously published results (Maguen et al., 2007; Arnal et al., 2011).

Furthermore, validation of the results involved determination of the spatial expression pattern of CIITA, which was downregulated in the *peripheral TR* of the CE samples of patients with post-LVC ectasia at both the RNA and protein levels in this study. CIITA is a regulator of the major histocompatibility complex (MHC) class II gene expression, whose depletion was previously reported in a relapse of acute myeloid leukemia (Toffalori et al., 2019). MHC class-II-positive Langerhans cells were observed in the limbus and periphery of the CE but not in the apex of normal mouse corneas, whereas the expression of MHC class II was also present in the apex part during inflammation resembling graft rejection (Hamrah et al., 2002). Alterations in *CIITA* expression have not been reported in post-LVC ectasia research; however, its role as a controller of adaptive and intrinsic immunities could be pivotal in recovery after refractive surgery.

The TBC1D4 protein (based on five different protein fragments) was significantly upregulated in the *central* and *middle TRs* of the CE samples of patients with post-LVC ectasia, as revealed by the multigroup analysis of proteomic data. In our previous study, the TBC1D4 protein was found to be elevated in the *peripheral TR* of patients with KTCN (Jaskiewicz et al., 2023a), which is in line with the lack of significant changes in this *TR* when comparing post-LVC ectasia with KTCN in the current study. Importantly, IF staining of additional CE samples confirmed the enriched expression of TBC1D4 in post-LVC ectasia. One of the well-known functions of this protein is its role in glucose homeostasis, where it mediates GLUT4 levels at the plasma membrane in response to insulin (Ha et al., 2019); however, unlike most tissues, the CE is characterized by insulin-independent glucose uptake (Chadt and Al-Hasani, 2020). Importantly, overexpression of the phospho-site mutants of TBC1D1 and TBC1D4 was reported to reduce cell surface expression of GLUT1 in non-insulin target cells (Henriques et al., 2020). Therefore, TBC1D4 could play a role in determining the subcellular distributions of GLUTs between different membrane compartments and hence influence the remodeling and wound healing of CE in post-LVC ectasia.

There are some important limitations to this study. Because it is impossible to obtain CE samples from healthy individuals without refractive errors, our control group constituted of individuals with mild myopia. However, to provide reliable results, the control individuals undergoing the PRK procedure underwent detailed clinical examinations before surgery as well as regular ophthalmological examinations for at least 3 years after the procedure. We note that the small sample size may have partially biased our findings regarding the ectasia rate after SMILE/LASIK as well as the associated ectasia risk factors. Moreover, owing to the limited sample size, we were unable to further investigate differences within the post-LVC ectasia study group, including the post-LASIK and post-SMILE subgroups. As the presented results entail epithelial transcriptomic and proteomic features, they may not fully reflect the mechanistic differences between post-LVC ectasia and KTCN. Most of the molecular findings presented herein reflect the donut pattern phenotype in the CEs of both post-LVC ectasia and KTCN patients. Nonetheless, we propose that subtle molecular features in the CE could precede the full clinical manifestation of ectasia (which involves both stromal weakening and epithelial remodeling) and that further investigations may be warranted.

In this first extensive multiomics study on post-LVC ectasia, the incidence of ectasia after SMILE and LASIK procedures showed no rate differences, whereas the residual stromal bed, stromal ablation depth, and percent tissue altered indices accurately predicted the risk of ectasia. Although some features of the transcription and inflammation processes could be used to differentiate patients with post-LVC ectasia from KTCN, similarities in the clinical as well as molecular backgrounds were observed more frequently. Currently, the safety of refractive surgeries is based on extended preoperative clinical examination, but further molecular studies involving potential biomarkers (*CADPS*, *CPT1B*, *CIITA*, and *TBC1D4*) assessed in the ectasia-free fellow eye (e.g., in the form of impression cytology) are expected to help improve the scoring of preoperative risk factors and identify patients at high risk of developing ectasia.

## Data Availability

The data presented in the study are deposited in the Mendeley Data Repository at https://data.mendeley.com/datasets/p656wtzjv8/1.

## Abbreviations

CE, corneal epithelium; CXL, corneal cross-linking; DEG, differentially expressed gene; KTCN, keratoconus; LASIK, laser *in situ* keratomileusis; LVC, laser vision correction; MALDI-TOF/TOF MS/MS, tandem matrix-assisted laser desorption/ionization time of flight/time of flight mass spectrometry; PRK, photorefractive keratectomy; SMILE, small incision lenticule extraction; TR, topographic region.
