# Supplementary material for: Shared molecular profiles of post-laser vision correction ectasia and keratoconus with key differences in CADPS, CPT1B, CIITA, and TBC1D4
Source: Front Mol Biosci. 2025 Aug 6;12:1616675. doi: 10.3389/fmolb.2025.1616675 (PMC12364684; doi:10.3389/fmolb.2025.1616675)
Supplement: Supplementary file 1 [file DataSheet1.pdf]

frontiers

Supplementary Material

Shared molecular profiles of post-laser vision correction ectasia and keratoconus with key differences in *CADPS, CPT1B, CIITA*, and *TBC1D4*

Katarzyna Jaskiewicz-Rajewicz[1*], Alicja Wysocka[1*], Magdalena Maleszka-Kurpiel[2,3], Eliza Matuszewska-Mach[4], Jakub Wozniak[5,6], Rafal Ploski[7], Jan Matysiak[4], Malgorzata Rydzanicz[7], Marzena Gajecka[1, 5]

[*] Equally contributed

[1] Institute of Human Genetics, Polish Academy of Sciences, Poznan, Poland

[2] Optegra Eye Health Care Clinic in Poznan, Poznan, Poland

[3] Chair of Ophthalmology and Optometry, Poznan University of Medical Sciences, Poznan, Poland

[4] Chair and Department of Inorganic and Analytical Chemistry, Poznan University of Medical Sciences, Poznan, Poland

[5] Chair and Department of Genetics and Pharmaceutical Microbiology, Poznan University of Medical Sciences, Poznan, Poland

[6] Department of Genetics and Animal Breeding, Poznan University of Life Sciences, Poznan, Poland

[7] Department of Medical Genetics, Medical University of Warsaw, Warsaw, Poland

1. Supplementary Data

1.1. Ophthalmic Examination

The ophthalmic examination embraced the assessments of both uncorrected (UCVA) and best corrected visual acuity (BCVA), intraocular pressure (IOP), corneal tomography with rotating Scheimpflug camera WaveLight® Oculyzer™ II (Alcon, Texas, US) or Pentacam (Oculus Optikgeraete GmbH; Wetzlar, Germany), epithelial thickness mapping (Spectral-Domain Optical Coherence Tomography [SD-OCT] device, Zeiss Cirrus 5000, Carl Zeiss Meditec, Dublin, California, USA, or MS-39, Costruzione Strumenti Oftalmici, Florence, Italy) slit-lamp, and dilated funduscopic examination. Patients were required to refrain from wearing contact lenses before examination (4 weeks, 2 weeks, and 1 week, for gas permeable rigid, toric, and soft lenses, respectively).

Also, a questionnaire comprising the behavioral, environmental, and socioeconomic aspects was completed by each participant, as previously described(1).

1.2. Inclusion criteria for control and KTCN individuals

The control individuals' inclusion criteria (males and females, non-ectasia patients aged over 18 years) comprised the mild myopia phenotype with no clinical signs of corneal ectasia in both eyes, examined in both the corneal tomography map and the corneal epithelium thickness map, without other corneal and eye abnormalities. A presence of topographic and tomographic abnormalities found in the cornea during the follow-up (lasting at least 3 years) after the PRK procedure was the exclusion criterion.

The inclusion criteria for individuals with keratoconus (KTCN). Males and females, patients aged over 18 years with progressive KTCN, defined by a consistent change in at least two of the following parameters: steepening of the anterior corneal surface, steepening of the posterior corneal surface, thinning and/or an increase in the rate of corneal thickness change

from the periphery to the thinnest point were the inclusion criteria, which are consistent with previously established inclusion criteria in our KTCN research(2,3).

1.3. Statistical Analyses of Clinical Parameters

The JASP Software(4) was used in statistical analyses of clinical parameters. The normality of continuous data was assessed by the Shapiro-Wilk test. The t-test was applied for continuous variables with a normal distribution. If the normality assumption was not satisfied, the Mann–Whitney was conducted. For all performed statistical tests p-values <0.05 were considered as statistically significant. As the rate of post-laser vision correction (post-LVC) ectasia is low, no sample size with an a priori power analysis was performed. For all significant results, the effect size metrics were verified (Cohen's d > 0.5).

1.4. Total RNA Library Preparation and Sequencing, and RNA-Seq Data Analyses

Total RNA libraries were prepared according to a previously established protocol(1,3), using TruSeq Stranded Total RNA Library Prep Gold (Illumina, San Diego, CA, USA) in accordance with the manufacturer's protocol. The subsequent steps of cytoplasmic and mitochondrial rRNA depletion, fragmentation of total RNA, cDNA first-strand synthesis using reverse transcriptase (SuperScript II Reverse Transcriptase, Thermo Fisher Scientific, Lithuania), and the second cDNA strand synthesis (DNA Polymerase I and RNase H, Illumina) were performed. Next, end repair, 3′ end adenylation, indexed adapter ligation (TruSeq RNA UD Indexes, Illumina), and cycles of PCR amplification were completed. The generated libraries were qualified using the Agilent HS DNA Kit (Agilent Technologies, Santa Clara, CA, USA), then quantified using the Qubit dsDNA High Sensitivity Kit (Life Technologies, Eugene, OR, USA).

Bioinformatic analyses were executed according to a previously established protocol(1). Briefly, reads were trimmed using the BBDuk2 program from the BBTools suite (http://jgi.doe.gov/data-and-tools/bbtools/) to remove Illumina adapters, poor-quality regions (mean Phred quality, <5), and reads matching human rRNA sequences. Kallisto assisted by GENCODE 34 (Ensembl 100) annotations were used to estimate the transcripts' expression values. The differential expression analysis was executed using the limma package(5,6). Genes were considered to be differentially expressed based on the following cutoffs: 0.05 for false discovery rate (FDR) and 1.5 for the absolute value of log2 transformed fold change (log2FC). Molecular Signatures Database (MsigDB) Hallmark Gene Set Collection(7) and Reactome(8,9) pathway enrichment analysis was performed using CAMERA method implemented in the limma package(10). Pathways/hallmarks were considered to be differentially represented based on the cutoff of the 0.05 FDR.

To create the heatmaps, the data was normalized using library size factors and log transformation with the scuttle package(11), to ensure consistency and comparability. Following normalization, we scaled the log-transformed counts to standardize the data, which involved adjusting the data so that each gene had a mean of zero and a standard deviation of one, using the scale function in R(12). The heatmaps were then generated using the pheatmap package(13), with hierarchical clustering performed using Euclidean distance. All analyses were conducted in the R statistical environment version 4.4.0(12).

1.5. Verification and validation of RNA-Seq data

To verify the RNA-Seq data the RNA samples used in the original RNA-Seq assessment, were reverse transcribed to cDNA with the Maxima First Strand cDNA Synthesis Kit for RT-qPCR, with dsDNase (Thermo Fisher Scientific Inc., Lithuania), according to the manufacturer’s procedure. Expression levels of selected genes (including three reference

transcripts, *UBC*, *LDHA*, and *RPL4)* were assessed using the HOT FIREPol EvaGreen qPCR Mix Plus (Solis BioDyne OÜ, Estonia) according to the manufacturer's protocol, using the CFX96 Touch Real-Time PCR Detection System (Bio-Rad Laboratories, Hercules, CA, USA). The reference genes were selected based on previous studies (14) and expression data from our KTCN research(1,3,15). Each reaction was performed in triplicates, and if one of the replicates deviated by ≥0.5 Ct it was removed and two replicates were included in the following calculations. The primer sequences and annealing temperatures are shown in Table S1. Relative quantification of the gene expression was normalized to the level of the *UBC*, *LDHA*, and *RPL4* transcripts with the comparative CT method. The log2 transformed FC values of gene expression levels between post-LVC ectasia, KTCN and control samples were calculated for the RT-qPCR and RNA-Seq data. Next, Pearson correlation coefficient between these values was obtained using the JASP Software(4).

Additionally, to validate the study results on RNA level, the additional CE samples, were collected and processed according to the study workflow (Figure 2), and next RT-qPCR reactions were performed. The Mann-Whitney U test was executed to verify the differences in expression levels of particular genes in post-LVC ectasia, compared to controls (based on delta Ct values), and box-plots were generated to visualize the results(4).

1.6. MALDI-TOF/TOF MS/MS Protein-Peptide Profiling

A tandem matrix-assisted laser desorption/ionization-time of flight/time of flight mass spectrometry (MALDI-TOF/TOF MS) analysis was performed according to previously established protocol(1,16). All samples were subjected to the in-solution tryptic overnight digestion, according to the modified Pierce Kit protocol (Thermo Scientific). Next, samples were desalted, purified, and concentrated using ZipTip C18 solid-phase extraction (SPE) micropipette tips (Millipore, Burlington, MA, USA) under the manufacturer's protocol. The

obtained SPE eluates were mixed with 0.7 mg/mL acetonitrile (ACN) / water solution of α-Cyano-4-hydroxycinnamic acid (HCCA) matrix and subjected to MS analysis.

During MALDI-TOF/TOF MS/MS proteomic analysis each experimental sample was manually spotted into the MTP AnchorChip 800 µm target plate (Bruker Daltonics, Bremen, Germany) in three technical repetitions. In further profiling, the means of three technical replicates were analyzed. Next, peaks (different protein/peptide fragments) with missing values in more than 30% of all samples were removed from analysis and the remaining missing values were filled by half of the minimum value. In the final statistical analyses, performed in JASP Software(4), 405 out of 1,484 peaks were included. This data did not meet the assumption of normality (verified using the Shapiro-Wilk test), therefore for two-group and more than two-group analyses, Mann–Whitney and Kruskal-Wallis tests were conducted, respectively. As a post-hoc test, Dunn's test with correction for multiple testing using Holm-Bonferroni method was performed. For all performed statistical tests p-values < 0.05 were considered as statistically significant (discriminative peaks). Only discriminative peaks remained for the subsequent identification using SwissProt protein sequence database. Not all discriminative peaks were unambiguously identified. To visualize the results box plots were generated(4).

1.7. Immunofluorescence (IF) staining of CE samples

The CE samples were rinsed 3x with PBS (pH 7.4) and then fixed for 10 minutes in ice-cold methanol at -20°C. After incubation samples were rinsed with PBS (3x) followed by adding a blocking buffer containing 5% BSA, 0,3% Triton, and 10% normal donkey serum (ab7475, Abcam) in PBS (pH 7.4) for 1 hour at room temperature. Subsequently, the samples were incubated overnight at 4°C with primary CIITA antibody (PA5-21031, Invitrogen) and primary TBC1D4 antibody (TA502707S, OriGene) at concentrations 1:200 and 1:25, respectively. Next, the tissues were rinsed with PBST (3x) and incubated for 2 hours at room

temperature in the dark with secondary antibodies Alexa Fluor® Plus 488 donkey anti-rabbit IgG (A32790, Invitrogen) and Alexa Fluor® Plus 405 donkey anti-mouse IgG (A48257, Invitrogen) at concentrations 1:1000 and 1:100 respectively. After incubation, the samples were rinsed with PBST, and counterstained with propidium iodide (Sigma Aldrich) (5µg/ml in blocking buffer) in the dark for 10 min at room temperature followed by rinsing with PBST and H_2O.

Samples prepared as abovementioned were analyzed under the Leica STELLARIS Confocal Microscope (Leica Microsystems GmbH).

2. Supplementary Tables

Table S1. The primer sequences and annealing temperatures used in RT-qPCR for RNA-Seq data verification and validation.

Gene	Primer Sequence	Product Size	Annealing Temperature
LDHA	F: AGGCTACACATCCTGGGCTA<br>R: TTCAAACGGGCCTCTTCCTC	216 bp	59°C
RPL4	F: ATCCAAAGAGCCCTTCGAGC<br>R: GAGCTTGTGATTCCTGGCCT	156 bp	65°C
UBC	F: CCACTCTGCACTTGGTCCTG<br>R: TTGGGAATGCAACAACTTTAT	109 bp	59°C
S100A14	F: ACAGTGTCGGTCAGCCAAC<br>R: TTCTCTTCCAGGCCACAGTT	195 bp	59°C
CADPS	F: CCGAATGGATAAGCCTCAAA<br>R: TCCACAGTGTAGCCATCCAA	192 bp	59°C
CPT1B	F: GCCAGGCCTTCTTTAGCTCT<br>R: CTGGCCATTCTTGAAGGAAA	212 bp	55°C
KLK5	F: AAGGCCCAACCAGCTCTACT<br>R: AACATCTGCTGCCCAGATTC	144 bp	59°C

Table S2A. The detailed clinical data of examined post-LVC ectasia, control, and KTCN individuals.
Clinical data concerning both eyes is presented, and samples subjected to RNA-Seq and/or MALDI-TOF/TOF MS/MS assessment are indicated.

Patient ID	Diagnosis	Sex	Examination age	Age at diagnosis	Examined eye	K1 [D]	K2 [D]	Kmax [D]	Anterior Elevation [µm]	Posterior Elevation [µm]	TCT [µm]	AL [mm]	IOP [mmHg]	Samples subjected to RNA-seq	Samples subjected to MALDI MS
1 OPT/M	CONTROL	F	33	n/a	OS	45.4	46.3	46.7	1	-1	507	22.98	14	yes	yes
					OD	45.4	46.5	47	2	-2	501	23.02	13	yes	yes
10 OPT/M	CONTROL	F	42	n/a	OS	45.5	46.3	46.7	1	5	533	22.89	15	yes	yes
					OD	45.2	46.3	46.8	1	5	527	22.9	15	yes	yes
11 OPT/M	CONTROL	F	23	n/a	OS	43	44.5	44.8	3	7	594	24.56	15	yes	yes
					OD	42.7	44.6	45	2	10	592	24.96	13	yes	yes
4 OPT/M	CONTROL	F	30	n/a	OS	44.4	45.3	45.7	1	4	516	23.77	16	yes	yes
					OD	44	44.8	45.2	0	-1	515	24.19	15	yes	yes
5 OPT/M	CONTROL	M	24	n/a	OS	43.3	44.2	44.4	2	2	531	23.31	nd	yes	yes
					OD	42.9	43.9	44.3	2	2	539	23.68	nd	yes	yes
6 OPT/M	CONTROL	M	30	n/a	OS	41.5	43.1	43.4	0	1	476	25.08	15	yes	yes
					OD	41.3	42.8	43.1	1	1	489	25.09	11	yes	yes
7 OPT/M	CONTROL	M	23	n/a	OS	38.2	38.9	40.1	1	6	504	26.26	13	no	no
					OD	39.6	40.9	41	3	5	550	25.56	16	yes	yes
8 OPT/M	CONTROL	M	21	n/a	OS	43.2	44	44.3	1	4	519	23.53	10	no	no
					OD	43	44	44.3	1	2	518	23.53	9	yes	yes
128 OPT/E	post-LVC ectasia	M	39	38[x]	OS	36.6	36.8	42.6	15	8	438	27.02	18	no	no
					OD	41.4	43.4	49	25	60	397	26.68	18	yes	yes
136 OPT/E	post-LVC ectasia	F	28	26	OS	41.0	43.3	46.9	12	35	404	24.65	13	no	no
					OD	39.2	40.8	42.3	14	20	419	24.76	11	yes	yes
146 OPT/E	post-LVC ectasia	F	42	41	OS	42.4	46.9	52.6	46	78	511	24.06	14	no	yes
					OD	40.3	43.1	50.4	27	55	558	24.03	14	no	no
17 OPT/E	post-LVC ectasia	M	45	44[x]	OS	50.3	57.8	65.4	48	103	361	27.73	9	yes	yes
					OD	40.9	46	56.4	37	59	397	27.76	10	no	no
26 OPT/E	post-LVC ectasia	F	43	43[x]	OS	44.2	44.9	45.6	4	8	566	22.49	12	no	no
					OD	45.9	48.4	53.4	13	37	539	21.27	12	yes	yes
27 OPT/E	post-LVC ectasia	M	46	45[x]	OS	36.9	39.2	46	22	34	501	26.88	11.2	yes	yes
					OD	38.5	39.1	43.1	4	5	516	26.62	10.9	no	no
31 OPT/E	post-LVC ectasia	M	32	32[x]	OS	38.2	39.5	44.6	2	7	376	24.94	11.4	no	no
					OD	39.9	40.6	48	12	16	353	24.81	13.1	yes	yes
51 OPT/E	post-LVC ectasia	M	22	21	OS	41.1	42.8	49.8	18	28	359	24.9	16	yes	yes
					OD	40.7	41	46.4	15	8	375	24.78	17	no	no
62 OPT/E	post-LVC ectasia	M	38	37	OS	39.2	41.8	45.4	10	46	379	nd	16.7	yes	yes
					OD	nd	nd	44.9	15	10	413	nd	16.7	no	no

72 OPT/E	post-LVC ectasia	F	30	29	OS	36.0	37.2	42.0	8	15	435	25.63	12	no	no
					OD	36.3	38.2	41.9	11	21	425	26.16	15	yes	yes
95 OPT/E	post-LVC ectasia	M	39	31	OS	41.6	43.9	50.7	25	41	473	nd	9	yes	yes
					OD	40.5	42.8	55.7	74	71	436	nd	11	no	no
11 OPT/E	KTCN	M	25	25	OS	42.1	44.7	49.7	24	61	432	23.77	11	yes	yes
					OD	42.4	42.8	45.2	1	25	367	23.76	12	no	no
140 OPT/E	KTCN	M	27	27	OS	43.4	47.4	54.9	25	45	453	24.88	11.8	no	no
					OD	44	48.8	57.3	31	55	449	24.97	10.8	yes	yes
143 OPT/E	KTCN	M	26	22	OS	43.6	47.9	53.8	22	46	470	23.9	18	no	yes
					OD	43.3	43.3	45.5	8	21	490	23.99	16	no	no
151 OPT/E	KTCN	M	23	23	OS	43.4	47.1	58.8	40	70	456	26.93	10	no	yes
					OD	41.6	43.8	54	30	57	471	26.67	11	no	no
154 OPT/E	KTCN	M	27	27	OS	41.4	42.3	43.4	6	12	469	24.8	19	no	no
					OD	42.1	44.1	48.3	14	37	452	24.91	18	no	yes
16 OPT/E	KTCN	M	28	28	OS	41.9	44.1	49.1	16	50	532	24.69	14	no	no
					OD	41.3	44	50.9	20	56	530	24.92	11	yes	yes
161 OPT/E	KTCN	M	22	22	OS	53.6	53.9	70.3	53	94	449	24.71	11	no	yes
					OD	44.8	45.4	50	14	29	503	24.75	13	no	no
166 OPT/E	KTCN	M	23	23	OS	53.6	59.8	71.4	43	81	411	23.4	7	no	yes
					OD	44	47	51.4	15	43	470	23.91	11	no	no
19 OPT/E	KTCN	M	20	19	OS	48.9	51.4	67	33	49	396	24.53	11	yes	yes
					OD	41.8	43.2	44.4	3	3	473	24.11	16	no	no
22 OPT/E	KTCN	M	25	23	OS	42.4	46.5	52.4	19	42	484	24.15	11	no	no
					OD	43.1	47.2	52.5	18	40	480	24.34	11	yes	yes
23 OPT/E	KTCN	M	31	30	OS	44.9	46.6	51.8	20	56	469	23.82	12	yes	yes
					OD	43.1	44	44.4	3	10	504	23.83	16	no	no
24 OPT/E	KTCN	M	39	nd	OS	50.2	56.3	63.7	34	55	419	22.51	9	yes	yes
					OD	43.1	44	44.4	3	10	436	22.54	10	no	no
25 OPT/E	KTCN	M	44	40	OS	54.9	58.5	69.3	45	89	398	25.92	9	yes	yes
					OD	42.3	43	44.2	6	11	508	24.76	16	no	no
29 OPT/E	KTCN	F	34	30	OS	44.2	44.9	45.6	4	8	344	nd	9	no	no
					OD	46.7	48.5	56.8	24	52	442	nd	6	yes	yes
3 OPT/E	KTCN	M	28	28	OS	43.5	44.7	52.1	24	52	449	26.17	11	yes	yes
					OD	42.1	43.2	48.2	15	32	469	26.21	12	no	no
32 OPT/E	KTCN	M	42	41	OS	47.7	51.1	53.3	32	65	407	24.1	8	yes	yes
					OD	44.2	46.8	51.3	26	57	442	24.2	10	no	no
33 OPT/E	KTCN	M	32	17	OS	42.1	42.4	42.8	3	4	596	24.54	17	no	no
					OD	46.8	48.5	57.5	27	80	497	24.67	11	yes	yes
34 OPT/E	KTCN	F	23	22	OS	45.8	46.6	50.7	22	54	503	23.02	nd	yes	yes
					OD	41.7	42.6	43.4	6	16	524	23.02	nd	no	no
35 OPT/E	KTCN	M	22	21	OS	42.1	43.2	43.7	3	5	533	24.38	14	no	no

					OD	48.5	51.6	62	26	74	453	24.53	14	yes	yes
43 OPT/E	KTCN	M	27	26	OS	43.2	44.8	48.3	17	39	373	nd	9	no	no
					OD	45.6	47.2	52.3	19	49	417	nd	9	no	yes
44 OPT/E	KTCN	M	25	25	OS	52.9	53.5	70	41	91	440	23.09	10	no	yes
					OD	48.8	49.5	58.9	23	51	460	22.9	11	no	no
64 OPT/E	KTCN	M	23	21	OS	50.9	52.8	61.3	48	83	364	24.08	16	yes	yes
					OD	43.1	44.5	54.4	38	70	408	23.99	17	no	no
67 OPT/E	KTCN	M	21	21	OS	45.2	49.2	61.7	44	73	430	23.18	16	no	no
					OD	44	48.1	60.4	50	90	420	23.33	17	yes	yes
7 OPT/E	KTCN	M	21	21	OS	41.7	45.4	53.4	20	37	502	24.06	11	yes	yes
					OD	50.9	54.6	73.6	63	115	438	24.21	16	no	no
75 OPT/E	KTCN	M	22	22	OS	43.9	50.8	59.7	33	75	501	23.83	17	yes	yes
					OD	42.3	44.8	48.9	12	38	535	23.6	16	no	no
80 OPT/E	KTCN	M	26	25	OS	40.7	41.8	51.4	32	71	485	24.09	12	no	no
					OD	41.7	44.9	52.2	30	66	471	24.14	11	yes	yes
82 OPT/E	KTCN	M	24	19	OS	43.2	45.4	50.4	18	39	534	23.7	19	no	no
					OD	56.2	66.9	84.1	58	164	319	24.1	19	yes	yes
9 OPT/E	KTCN	M	18	18	OS	40.8	41	52.1	24	46	473	25.58	9	yes	yes
					OD	41	41.6	50.3	22	37	478	25.75	10	no	no

[x] - The age at the time of diagnosis by the clinician was given, although according to the patient, the ectasia symptoms appeared earlier,

* Abbreviations in table: M – male, F – female, K1 – flat keratometric readings, K2 – steep keratometric readings, Kmax – maximum simulated keratometry, OD – right eye, OS – left eye, nd – data not available, n/a – not applicable, TCT – thinnest corneal thickness, AL - Axial length, IOP – intraocular pressure, KTCN – keratoconus, post-LVC ectasia – post-laser vision correction ectasia, MALDI-MS – tandem matrix-assisted laser desorption/ionization-time of flight/time of flight mass spectrometry.

Table S2B. The detailed clinical data regarding the thickness of CE of examined individuals.
Presented clinical data embraces average CE thickness in ranges 0.0-2.0, 2.0-5.0, 5.0-7.0, and 7.0-9.0mm from automatic assessment of examined post-LVC ectasia, control, and KTCN individuals, together with the average thickness of *central/ middle/ peripheral TR* of CE in manual assessment, and description of cone location.

Patient ID	Diagnosis	Sex	Examined eye	TET (in range 0.0-7.0mm) [µm]	Average thickness in 0.0-2.0mm range [µm]	Average thickness, 2.0-5.0mm range [µm]	Average thickness in 5.0-7.0mm range [µm]	Average thickness in 7.0-9.0mm range [µm]	Cone location (central, superior/inferior, nasal/temporal)	Average thickness of 1st*TR* [µm]	Average thickness of 2^{tn}*TR* [µm]	Average thickness of 3rd*TR* [µm]
1 OPT/M	CONTROL	F	OS	44	52	49	47	47	central, central	52	48	47
			OD	45	48	47	46	45	central, central	48	47	45
10 OPT/M	CONTROL	F	OS	47	54	51	49	48	central, temporal	54	50	48
			OD	46	55	52	51	48	central, temporal	55	51	48
11 OPT/M	CONTROL	F	OS	49	55	54	52	49	central, central	55	53	50
			OD	48	54	53	52	44	central, central	54	54	50
4 OPT/M	CONTROL	F	OS	41	46	44	44	44	central, central	46	44	44
			OD	41	48	45	44	45	central, central	48	45	45
5 OPT/M	CONTROL	M	OS	46	52	51	49	49	central, temporal	52	50	49
			OD	46	52	50	49	48	central, central	51	49	48
6 OPT/M	CONTROL	M	OS	44	49	47	45	46	central, temporal	49	46	45
			OD	45	49	48	46	46	central, central	49	46	45
7 OPT/M	CONTROL	M	OS	46	59	57	50	45	central, central	58	52	43
			OD	43	53	49	45	43	central, central	53	48	40
8 OPT/M	CONTROL	M	OS	51	57	55	53	52	central, central	57	53	51
			OD	52	58	56	54	52	central, central	58	56	53
128 OPT/E	post-LVC ectasia	M	OS	50	64	65	56	50	central, central	63	68	51
			OD	36	42	63	57	51	central, central	40	65	50
136 OPT/E	post-LVC ectasia	F	OS	48	52	64	60	56	central, central	50	65	54
			OD	53	55	61	58	54	central, central	53	60	53
146 OPT/E	post-LVC ectasia	F	OS	43	61	53	53	49	central, central	45	61	49
			OD	49	61	57	52	49	central, inferior	51	61	49
17 OPT/E	post-LVC ectasia	M	OS	42	42	54	52	47	central, central	42	58	45
			OD	40	46	52	49	46	central, temporal	42	57	46
26 OPT/E	post-LVC ectasia	F	OS	43	51	49	47	50	central, central	49	53	48
			OD	43	44	47	53	49	inferior, temporal	44	56	48
27 OPT/E	post-LVC ectasia	M	OS	42	63	56	48	45	inferior, temporal	42	61	48
			OD	42	57	48	39	nd	central, central	54	59	49
31 OPT/E	post-LVC ectasia	M	OS	44	46	45	39	nd	central, central	47	52	46
			OD	46	50	51	49	44	central, central	44	55	42
51 OPT/E	post-LVC ectasia	M	OS	47	58	57	56	52	central, central	48	64	57

			OD	50	61	57	54	50	central, central	61	56	52
62 OPT/E	post-LVC ectasia	M	OS	42	45	56	53	49	central, central	43	57	49
			OD	43	52	54	50	48	central, central	47	55	50
72 OPT/E	post-LVC ectasia	F	OS	50	59	57	55	53	central, central	59	57	54
			OD	48	57	58	54	51	central, central	55	61	53
95 OPT/E	post-LVC ectasia	M	OS	46	53	54	51	49	central, central	48	55	50
			OD	43	53	50	47	47	inferior, temporal	44	57	48
11 OPT/E	KTCN	M	OS	39	54	49	46	45	inferior, temporal	40	53	46
			OD	45	55	50	46	46	inferior, temporal	45	53	46
140 OPT/E	KTCN	M	OS	44	45	53	53	50	central, central	45	56	50
			OD	42	44	53	53	50	central, central	44	55	50
143 OPT/E	KTCN	M	OS	44	53	57	57	56	central, central	46	61	56
			OD	50	61	58	58	57	central, central	54	60	56
151 OPT/E	KTCN	M	OS	45	51	61	56	53	central, central	50	63	51
			OD	46	55	56	54	53	central, central	50	60	51
154 OPT/E	KTCN	M	OS	50	56	54	52	51	central, central	52	56	51
			OD	50	55	57	54	51	central, central	51	60	50
16 OPT/E	KTCN	M	OS	44	46	49	48	48	inferior, central	44	50	47
			OD	43	45	48	49	48	inferior, central	43	50	48
161 OPT/E	KTCN	M	OS	49	58	60	58	54	central, central	51	65	53
			OD	45	59	52	54	51	central, central	49	61	51
166 OPT/E	KTCN	M	OS	40	45	53	56	53	central, central	45	59	52
			OD	47	52	55	55	52	central, central	49	57	52
19 OPT/E	KTCN	M	OS	34	35	43	49	47	central, temporal	36	52	45
			OD	40	46	44	43	46	central, temporal	42	45	43
22 OPT/E	KTCN	M	OS	46	53	51	50	49	inferior, temporal	46	53	48
			OD	46	50	51	51	48	inferior, temporal	46	53	48
23 OPT/E	KTCN	M	OS	45	54	55	55	53	inferior, temporal	46	62	45
			OD	54	63	62	61	57	central, temporal	57	64	56
24 OPT/E	KTCN	M	OS	37	39	46	51	50	central, temporal	38	52	46
			OD	40	42	47	50	50	central, central	46	52	43
25 OPT/E	KTCN	M	OS	36	36	43	52	49	central, central	38	52	46
			OD	41	53	49	44	44	inferior, temporal	46	52	43
29 OPT/E	KTCN	F	OS	34	34	44	51	50	central, central	35	54	46
			OD	36	36	48	52	51	central, temporal	37	57	44
3 OPT/E	KTCN	M	OS	40	47	46	51	48	central, temporal	41	52	43
			OD	43	50	48	46	42	central, temporal	43	52	45
32 OPT/E	KTCN	M	OS	37	43	46	46	48	central, temporal	40	52	46
			OD	37	43	46	46	48	inferior, central	39	52	46
33 OPT/E	KTCN	M	OS	46	52	51	49	48	inferior, temporal	52	50	48
			OD	35	35	47	51	46	central, temporal	36	52	46

34 OPT/E	KTCN	F	OS	45	45	51	53	53	central, temporal	46	54	48
			OD	48	49	50	50	51	inferior, temporal	48	53	48
35 OPT/E	KTCN	M	OS	45	51	49	47	46	central, central	50	49	47
			OD	37	37	48	51	48	central, central	37	53	45
43 OPT/E	KTCN	M	OS	50	54	63	61	57	inferior, temporal	50	66	58
			OD	49	50	55	56	57	inferior, temporal	49	55	57
44 OPT/E	KTCN	M	OS	38	40	51	50	49	central, central	39	54	50
			OD	45	47	50	52	50	central, temporal	46	56	51
64 OPT/E	KTCN	M	OS	37	40	51	56	54	central, central	39	60	55
			OD	42	47	58	55	54	central, central	43	61	55
67 OPT/E	KTCN	M	OS	38	44	50	49	50	central, central	42	52	50
			OD	35	42	50	49	49	central, central	43	55	50
7 OPT/E	KTCN	M	OS	49	55	53	50	49	inferior, temporal	49	58	48
			OD	36	36	48	53	48	central, temporal	38	54	47
75 OPT/E	KTCN	M	OS	41	45	53	52	50	central, central	44	58	51
			OD	45	54	56	52	50	inferior, central	48	60	51
80 OPT/E	KTCN	M	OS	46	61	64	61	54	inferior, temporal	49	69	54
			OD	42	59	62	61	56	inferior, temporal	45	68	53
82 OPT/E	KTCN	M	OS	36	53	57	54	49	central, central	50	60	50
			OD	39	52	55	54	50	inferior, temporal	48	60	50
9 OPT/E	KTCN	M	OS	43	54	48	46	47	inferior, nasal	44	52	47
			OD	44	53	49	48	47	inferior, central	45	53	46

* Abbreviations in table: CE – corneal epithelium, M – male, F – female, OD – right eye, OS – left eye, TET – thinnest epithelial thickness, KTCN – keratoconus, post-LVC ectasia – post-laser vision correction ectasia, *TR – topographic region*, *1^{st}TR – central topographic region*, *2^{nd}TR – middle topographic region*, *3^{rd}TR – peripheral topographic region*.

Table S2C. The detailed clinical data regarding the refractive surgical procedure of patients with post-LVC ectasia.
The information regarding refractive surgical procedure such as method, date, flap thickness, ablation depth, optical zone, residual stromal bed (RSB), percentage of tissue altered (PTA), preoperative central corneal thickness (CCT), and preoperative refractive error is presented.

Patient ID	Diagnosis	Date of refractive surgical procedure	Age of refractive surgical procedure	Refractive surgical procedure	Date (month, year) of ectasia diagnosis	Time (months/years) from the refractive surgical procedure after which ectasia was diagnosed	Examined eye	Preoperative refractive error (Dsph/ Dcyl/ ax)	Preoperative CCT [µm]	Flap thickness [µm]	Ablation depth [µm]	Optical zone [mm]	RSB [µm]	PTA [%]
128 OPT/E	post-LVC ectasia	18.03.2008	24	LASIK	2.2022	14 years[x]	OS	-7.0/-1.0/175	534	120	108.1	6.5	302	42.72
							OD	-6.75/-0.75/3	528	120	113.63	7	294	44.25
136 OPT/E	post-LVC ectasia	26.02.2020	26	SMILE	5.2020	3 months	OS	-3.75/-1.5/10	519	135	104	6.7	280	46.05
							OD	-4.0/-1.0/35	515	130	107	6.7	278	46.02
146 OPT/E	post-LVC ectasia	x.x.2009	28	LASIK	1.2023	13 years	OS	-3.5/-0.5/25	566	140	59.92	6.5	356	35.32
							OD	-3.0/-0.25/112	559	140	49.08	6.5	365	33.82
17 OPT/E	post-LVC ectasia	11.03.2008	32	LASIK	1.2020	12 years[x]	OS	-7.75/-0.75/160	527	140	121.01	6.05	265	49.53
							OD	-8.0/-0.75/20	535	140	124.24	6.05	270	49.39
26 OPT/E	post-LVC ectasia	x.2013	36	nd	3.2020	7 years[x]	OS	nd	nd	nd	nd	nd	nd	nd
							OD	nd	nd	nd	nd	nd	nd	nd
27 OPT/E	post-LVC ectasia	23.04.2008	34	LASIK	x.2019	11 years[x]	OS	-5.25/-0.75/90	580	140	87.78	6.5	352	39.27
							OD	-5.75/-0.25/60	580	140	84.53	6.5	355	38.71
31 OPT/E	post-LVC ectasia	10.01.2019	31	LASIK	4.2020	1 year[x]	OS	-4.0/-3.25/15	495	100	nd	nd	nd	nd
							OD	-3.5/-4.75/15	492	100	nd	nd	nd	nd
51 OPT/E	post-LVC ectasia	24.08.2018	20	SMILE	1.2019	5 months	OS	-3.5/-1.25/180	469	nd	nd	nd	nd	nd
							OD	-3.5/-1.25/5	474	nd	nd	nd	nd	nd
62 OPT/E	post-LVC ectasia	27.12.2018	35	SMILE	12.2020	2 years	OS	-5.25/-2.25/18	521	130	124	6.4	257	48.75
							OD	-5.75/-2.25/5	511	130	118	6.4	260	48.53
72 OPT/E	post-LVC ectasia	18.12.2019	28	SMILE	3.2021	1 year	OS	-5.5/-0.5/180	537	135	130	7	272	49.25
							OD	-5.5/-0.75/175	536	135	134	7	276	50.19
95 OPT/E	post-LVC ectasia	26.10.2010	28	LASIK	x.2013	3 years	OS	-4.5/0/0	514	120	67.09	6.5	343	36.40
							OD	-5.0/-0.25/10	507	120	81.07	6.5	301	39.66

[x] - The time from the refractive surgical procedure to ectasia diagnosis by the clinician is given, although according to the patient, the ectasia symptoms appeared earlier,

* Abbreviations in table: post-LVC ectasia – post-laser vision correction ectasia, OD – right eye, OS – left eye, CCT – central corneal thickness, RSB – residual stromal bed, SMILE – Small Incision Lenticule Extraction, LASIK – Laser-Assisted in situ Keratomileusis, PTA – percentage of tissue altered.

Table S3A. The detailed clinical data of additional examined post-LVC ectasia individuals.

Clinical data concerning both eyes is presented, and samples subjected to RT-qPCR experiments are indicated. Presented clinical data embraces average CE thickness in ranges 0.0-2.0, 2.0-5.0, 5.0-7.0, and 7.0-9.0mm from automatic assessment of examined post-LVC ectasia, control, and KTCN individuals, together with the average thickness of *central/ middle/ peripheral TRs* of CE in manual assessment, and description of cone location.

Patient ID	Sex	Examination age	Age at diagnosis	Examined eye	K1 [D]	K2 [D]	Kmax [D]	Anterior Elevation [µm]	Posterior Elevation [µm]	TCT [µm]	AL [mm]	IOP [mmHg]	TET (in range 0.0-7.0mm) [µm]	Average thickness in 0.0-2.0mm range [µm]	Average thickness in 2.0-5.0mm range [µm]	Average thickness in 5.0-7.0mm range [µm]	Average thickness in 7.0-9.0mm range [µm]	Cone location (central, superior/ inferior, nasal/ temporal)	Average thickness of 1st *TR* [µm]	Average thickness of 2nd *TR* [µm]	Average thickness of 3rd *TR* [µm]	samples subjected to RTqPCR
176 OPT/E	F	35	34	OS	42.0	42.3	43.3	2	6	456	nd	13.9	53	55	59	56	53	central, central	54	59	52	no
				OD	43.1	43.8	46.6	9	20	436	nd	13.6	44	50	58	57	53	central, central	47	59	53	yes
178 OPT/E	M	45	45 [x]	OS	41.6	42.3	43.6	8	15	468	24.25	13	46	56	55	51	49	central, central	52	56	49	no
				OD	41.9	44.9	47.0	14	36	451	24.23	11	45	53	54	51	50	central, central	50	55	49	yes
187 OPT/E	M	40	36	OS	37.6	41.4	48.7	15	26	418	nd	15	52	59	60	56	52	central, central	52	60	52	yes
				OD	39.7	41.0	45.5	10	14	453	nd	15	51	57	59	59	55	central, central	52	61	54	yes
188 OPT/E	F	26	24	OS	39.4	40.8	42.2	4	9	418	nd	13.8	51	56	59	57	54	central, central	55	60	52	yes
				OD	39.6	41.7	44.1	13	15	409	nd	14	52	57	61	57	54	central, central	55	61	52	yes
195 OPT/E	F	31	30	OS	37.0	39.5	42.6	10	21	434	nd	nd	51	54	57	54	50	central, central	52	58	50	yes
				OD	37.8	38.5	42.0	11	31	414	nd	nd	49	51	57	53	47	central, central	49	61	47	yes

[x] - The age at the time of diagnosis by the clinician was given, although according to the patient, the ectasia symptoms appeared earlier,

* Abbreviations in table: M – male, F – female, K1 – flat keratometric readings, K2 – steep keratometric readings, Kmax – maximum simulated keratometry, OD – right eye, OS – left eye, nd – data not available, n/a – not applicable, TCT – thinnest corneal thickness, AL - Axial length, IOP – intraocular pressure, post-LVC ectasia – post-laser vision correction ectasia, RTqPCR – quantitative reverse transcription polymerase chain reaction, *TR – topographic region*, *1stTR – central topographic region*, *2ndTR – middle topographic region*, *3rdTR – peripheral topographic region*.

Table S3B. The detailed clinical data regarding the refractive surgical procedure of additional examined post-LVC ectasia individuals. The information regarding refractive surgical procedure such as method, date, flap thickness, ablation depth, optical zone, residual stromal bed (RSB), percentage of tissue altered (PTA), preoperative central corneal thickness (CCT), and preoperative refractive error is presented.

Patient ID	Diagnosis	Date of refractive surgical procedure	Age of refractive surgical procedure	Refractive surgical procedure	Date (month, year) of ectasia diagnosis	Time (months/years) from the refractive surgical procedure after which ectasia was diagnosed	Examined eye	Preoperative refractive error (Dsph/ Dcyl/ ax)	Preoperative CCT [µm]	Flap thickness [µm]	Ablation depth [µm]	Optical zone [mm]	RSB [µm]	PTA [%]
176 OPT/E	post-LVC ectasia	18.03.2022	34	SMILE	6.2022	3 months	OS	-1.75/-0.75/95	499	135	66	7	298	40.28
							OD	-1.75/-1.0/105	499	135	71	7	290	41.28
178 OPT/E	post-LVC ectasia	x.x.2018	40	nd	5.2023	5 years[x]	OS	-3.25/-0.75/100	523	140	nd	nd	nd	26.77
							OD	-3.0/-0.5/70	528	140	nd	nd	nd	26.52
187 OPT/E	post-LVC ectasia	19.12.2019	35	SMILE	12.2020	1 year	OS	-6.75/0/0	515	135	120	6.4	266	49.51
							OD	-2.5/-0.75/112	509	135	81	7	291	42.44
188 OPT/E	post-LVC ectasia	19.10.2022	24	SMILE	1.2023	3 months	OS	-4.75/-0.25/160	517	130	118	7	265	47.97
							OD	-5.25/-1.0/15	512	130	120	6.6	256	48.83
195 OPT/E	post-LVC ectasia	x.x.2019	26	SMILE	4.2024	5 years	OS	nd	nd	nd	nd	nd	nd	nd
							OD	nd	nd	nd	nd	nd	nd	nd

[x] - The time from the refractive surgical procedure to ectasia diagnosis by the clinician is given, although according to the patient, the ectasia symptoms appeared earlier,

* Abbreviations in table: post-LVC ectasia – post-lser vision correction ectasia, OD – right eye, OS – left eye, CCT – central corneal thickness, RSB – residual stromal bed, SMILE – Small Incision Lenticule Extraction, LASIK – Laser-Assisted in situ Keratomileusis, PTA – percentage of tissue altered.

Table S4. The results of the quality and quantity control of RNA samples.
The results of quality (Nanodrop ND-1000) and quantity of the purified RNA (RNA 6000 Nano Kit, Agilent Technologies) and the number of RNAseq read before and after quality control (including rRNA depletion) for each analyzed sample. After rRNA depletion, we obtained at least 48.6 mln reads (in a range of 48.6 - 168.8 mln) per sample.

No.	Sample ID	Patient ID	*TR* of CE	Diagnosis	DNase I treatment	260/280 ratio	RNA concentration [ng/ul]	RIN values	Total RNA input [ng]	Total reads	No rRNA trimmed_reads
1.	1 OPT/M/OS/1	1 OPT/M	1st	CONTROL	yes	2.0	153.0	8.2	810.9	101882519	80309283
2.	1 OPT/M/OS/2	1 OPT/M	2nd	CONTROL	yes	2.0	208.0	7.9	811.2	71644110	67959339
3.	1 OPT/M/OS/3	1 OPT/M	3rd	CONTROL	yes	2.1	570.0	7.9	798.0	74897811	71539319
4.	1 OPT/M/OD/1	1 OPT/M	1st	CONTROL	yes	2.1	134.0	8.0	804.0	172934970	168824960
5.	1 OPT/M/OD/2	1 OPT/M	2nd	CONTROL	yes	2.1	324.0	8.2	810.0	83307794	81000364
6.	1 OPT/M/OD/3	1 OPT/M	3rd	CONTROL	yes	2.1	359.0	8.2	825.7	177917811	167701580
7.	10 OPT/M/OS/1	10 OPT/M	1st	CONTROL	yes	1.9	62.0	5.5	496.0	118285021	113555832
8.	10 OPT/M/OS/2	10 OPT/M	2nd	CONTROL	yes	1.9	113.0	4.0	802.3	78537489	76323055
9.	10 OPT/M/OS/3	10 OPT/M	3rd	CONTROL	yes	2.0	271.0	4.1	813.0	67140794	64008692
10.	10 OPT/M/OD/1	10 OPT/M	1st	CONTROL	yes	1.9	25.0	5.4	225.0	79401052	59983381
11.	10 OPT/M/OD/2	10 OPT/M	2nd	CONTROL	yes	2.0	86.0	4.3	799.8	82830681	79022897
12.	10 OPT/M/OD/3	10 OPT/M	3rd	CONTROL	yes	2.0	390.0	4.9	819.0	130834175	125362985
13.	11 OPT/M/OS/1	11 OPT/M	1st	CONTROL	yes	1.9	54.0	5.4	540.0	80223595	77225574
14.	11 OPT/M/OS/2	11 OPT/M	2nd	CONTROL	yes	2.0	78.0	5.0	780.0	122518566	116706801
15.	11 OPT/M/OS/3	11 OPT/M	3rd	CONTROL	yes	2.0	151.0	5.0	800.3	107381958	104078028
16.	11 OPT/M/OD/1	11 OPT/M	1st	CONTROL	yes	1.8	32.0	4.6	320.0	70908921	67817965
17.	11 OPT/M/OD/2	11 OPT/M	2nd	CONTROL	yes	2.0	72.0	3.2	720.0	73189089	69983192
18.	11 OPT/M/OD/3	11 OPT/M	3rd	CONTROL	yes	2.1	144.0	4.1	806.4	75887656	65000013
19.	4 OPT/M/OS/1	4 OPT/M	1st	CONTROL	yes	2.1	115.0	8.7	805.0	78531685	72616233
20.	4 OPT/M/OS/2	4 OPT/M	2nd	CONTROL	yes	2.1	232.0	9.2	812.0	88312300	70509627
21.	4 OPT/M/OS/3	4 OPT/M	3rd	CONTROL	yes	2.0	617.0	8.2	802.1	67410671	64690536
22.	4 OPT/M/OD/1	4 OPT/M	1st	CONTROL	yes	2.1	64.0	6.6	640.0	145402612	135147738
23.	4 OPT/M/OD/2	4 OPT/M	2nd	CONTROL	yes	2.1	238.0	6.9	809.2	141267531	135765833
24.	4 OPT/M/OD/3	4 OPT/M	3rd	CONTROL	yes	2.1	643.0	8.5	835.9	122707897	116871083
25.	5 OPT/M/OS/1	5 OPT/M	1st	CONTROL	yes	2.1	136.0	7.1	816.0	113219818	108983944
26.	5 OPT/M/OS/2	5 OPT/M	2nd	CONTROL	yes	2.1	143.0	7.1	800.8	111430762	107650394
27.	5 OPT/M/OS/3	5 OPT/M	3rd	CONTROL	yes	2.1	338.0	7.7	811.2	105965206	103294222
28.	5 OPT/M/OD/1	5 OPT/M	1st	CONTROL	yes	1.9	58.0	8.5	580.0	66603591	61319599
29.	5 OPT/M/OD/2	5 OPT/M	2nd	CONTROL	yes	2.1	247.0	8.3	815.1	76271406	63426651
30.	5 OPT/M/OD/3	5 OPT/M	3rd	CONTROL	yes	2.1	701.0	8.4	771.1	66452129	61856751
31.	6 OPT/M/OS/1	6 OPT/M	1st	CONTROL	yes	2.1	67.0	6.9	670.0	90711882	62282334

32.	6 OPT/M/OS/2	6 OPT/M	2nd	CONTROL	yes	2.1	189.0	7.2	793.8	65243160	62725328
33.	6 OPT/M/OS/3	6 OPT/M	3rd	CONTROL	yes	2.1	124.0	6.5	806.0	91362069	72891558
34.	6 OPT/M/OD/1	6 OPT/M	1st	CONTROL	yes	2.0	165.0	8.8	792.0	166892756	152226542
35.	6 OPT/M/OD/2	6 OPT/M	2nd	CONTROL	yes	2.0	337.0	8.4	808.8	121383060	119608259
36.	6 OPT/M/OD/3	6 OPT/M	3rd	CONTROL	yes	2.0	507.0	8.4	811.2	221452408	117868501
37.	7 OPT/M/OD/1	7 OPT/M	1st	CONTROL	yes	2.1	162.0	8.5	810.0	190902805	151631532
38.	7 OPT/M/OD/2	7 OPT/M	2nd	CONTROL	yes	2.1	256.0	7.7	819.2	114248793	108682018
39.	7 OPT/M/OD/3	7 OPT/M	3rd	CONTROL	yes	2.1	313.0	7.5	813.8	104184319	102354882
40.	8 OPT/M/OD/1	8 OPT/M	1st	CONTROL	yes	2.0	150.0	6.7	810.0	119207787	113051496
41.	8 OPT/M/OD/2	8 OPT/M	2nd	CONTROL	yes	2.0	258.0	7.6	799.8	76354523	73161232
42.	8 OPT/M/OD/3	8 OPT/M	3rd	CONTROL	yes	2.0	576.0	7.7	806.4	81863750	78692686
43.	128 OPT/E/OD/1	128 OPT/E	1st	post-LVC ectasia	yes	2.0	111.0	9.1	799.2	66546476	63831049
44.	128 OPT/E/OD/2	128 OPT/E	2nd	post-LVC ectasia	yes	1.8	18.0	9.4	180.0	103879920	88343765
45.	128 OPT/E/OD/3	128 OPT/E	3rd	post-LVC ectasia	yes	2.0	122.0	8.5	805.2	67849667	65940518
46.	136 OPT/E/OD/1	136 OPT/E	1st	post-LVC ectasia	yes	2.1	20.0	9.6	200.0	103938896	99168241
47.	136 OPT/E/OD/2	136 OPT/E	2nd	post-LVC ectasia	yes	2.0	165.0	9.1	808.5	96343834	90520500
48.	136 OPT/E/OD/3	136 OPT/E	3rd	post-LVC ectasia	yes	2.1	285.0	8.8	798.0	64165334	62519460
49.	17 OPT/E/OS/1	17 OPT/E	1st	post-LVC ectasia	yes	2.0	111.0	6.6	799.2	134246892	113346807
50.	17 OPT/E/OS/2	17 OPT/E	2nd	post-LVC ectasia	yes	2.1	189.0	7.3	793.8	139245796	134621734
51.	17 OPT/E/OS/3	17 OPT/E	3rd	post-LVC ectasia	yes	2.1	227.0	7.7	794.5	160045844	152476488
52.	26 OPT/E/OD/1	26 OPT/E	1st	post-LVC ectasia	yes	2.0	71.0	7.8	710.0	138063241	133166278
53.	26 OPT/E/OD/2	26 OPT/E	2nd	post-LVC ectasia	yes	2.1	80.0	8.0	800.0	164496209	159990844
54.	26 OPT/E/OD/3	26 OPT/E	3rd	post-LVC ectasia	yes	2.1	234.0	8.5	795.6	131840660	125812894
55.	27 OPT/E/OS/1	27 OPT/E	1st	post-LVC ectasia	yes	2.0	120.0	8.1	804.0	155655569	151534344
56.	27 OPT/E/OS/2	27 OPT/E	2nd	post-LVC ectasia	yes	2.1	167.0	8.0	801.6	149854822	145173718
57.	27 OPT/E/OS/3	27 OPT/E	3rd	post-LVC ectasia	yes	2.1	444.0	8.5	799.2	142381094	134948232
58.	31 OPT/E/OD/1	31 OPT/E	1st	post-LVC ectasia	yes	1.9	49.0	6.9	490.0	136160507	129168490
59.	31 OPT/E/OD/2	31 OPT/E	2nd	post-LVC ectasia	yes	2.0	91.0	7.9	800.8	134286726	129556956
60.	31 OPT/E/OD/3	31 OPT/E	3rd	post-LVC ectasia	yes	2.8	174.0	7.6	800.4	127959441	119611275
61.	51 OPT/E/OS/1	51 OPT/E	1st	post-LVC ectasia	yes	2.0	61.0	8.4	610.0	69933748	68338745
62.	51 OPT/E/OS/2	51 OPT/E	2nd	post-LVC ectasia	yes	2.0	208.0	8.7	811.2	64134245	61362460
63.	51 OPT/E/OS/3	51 OPT/E	3rd	post-LVC ectasia	yes	2.0	319.0	8.5	797.5	62261650	60177140
64.	62 OPT/E/OS/1	62 OPT/E	1st	post-LVC ectasia	yes	1.9	25.0	9.3	250.0	95280224	90921123
65.	62 OPT/E/OS/2	62 OPT/E	2nd	post-LVC ectasia	yes	2.0	29.0	8.6	290.0	75869655	72115849
66.	62 OPT/E/OS/3	62 OPT/E	3rd	post-LVC ectasia	yes	2.0	129.0	8.8	799.8	74477717	63008546
67.	72 OPT/E/OD/1	72 OPT/E	1st	post-LVC ectasia	yes	1.9	27.0	9.3	270.0	64651730	60646699
68.	72 OPT/E/OD/2	72 OPT/E	2nd	post-LVC ectasia	yes	2.0	119.0	9.3	797.3	65751328	62255965
69.	72 OPT/E/OD/3	72 OPT/E	3rd	post-LVC ectasia	yes	2.1	234.0	8.7	795.6	79645979	75097404
70.	95 OPT/E/OS/1	95 OPT/E	1st	post-LVC ectasia	yes	2.0	76.0	9.4	760.0	116182658	108046937
71.	95 OPT/E/OS/2	95 OPT/E	2nd	post-LVC ectasia	yes	2.0	24.0	8.5	240.0	100354803	95169756
72.	95 OPT/E/OS/3	95 OPT/E	3rd	post-LVC ectasia	yes	2.0	148.0	8.9	799.2	146486958	74927371

73.	11 OPT/E/OS/1	11 OPT/E	1st	KTCN	yes	2.0	93.0	7.3	799.8	107752136	104263773
74.	11 OPT/E/OS/2	11 OPT/E	2nd	KTCN	yes	2.1	99.0	7.1	801.9	135313909	129992563
75.	11 OPT/E/OS/3	11 OPT/E	3rd	KTCN	yes	2.1	552.0	7.8	828.0	109561740	105392029
76.	140 OPT/E/OD/1	140 OPT/E	1st	KTCN	yes	2.0	63.0	8.0	630.0	72278183	67280623
77.	140 OPT/E/OD/2	140 OPT/E	2nd	KTCN	yes	2.0	115.0	8.3	805.0	76735915	73152043
78.	140 OPT/E/OD/3	140 OPT/E	3rd	KTCN	yes	2.0	408.0	7.7	816.0	72710293	69060332
79.	16 OPT/E/OD/1	16 OPT/E	1st	KTCN	yes	2.0	102.0	8.4	795.6	161399094	157362841
80.	16 OPT/E/OD/2	16 OPT/E	2nd	KTCN	yes	2.1	241.0	8.3	795.3	111090866	107139191
81.	16 OPT/E/OD/3	16 OPT/E	3rd	KTCN	yes	2.1	413.0	8.2	784.7	145595476	141735446
82.	19 OPT/E/OS/1	19 OPT/E	1st	KTCN	yes	2.1	102.0	8.0	795.6	120807143	116604903
83.	19 OPT/E/OS/2	19 OPT/E	2nd	KTCN	yes	2.1	245.0	8.0	808.5	91785473	88314617
84.	19 OPT/E/OS/3	19 OPT/E	3rd	KTCN	yes	2.1	410.0	8.1	820.0	163942509	159966932
85.	22 OPT/E/OD/1	22 OPT/E	1st	KTCN	yes	2.1	43.0	9.4	430.0	119076146	108579045
86.	22 OPT/E/OD/2	22 OPT/E	2nd	KTCN	yes	2.1	96.0	8.7	796.8	135849671	129125558
87.	22 OPT/E/OD/3	22 OPT/E	3rd	KTCN	yes	2.1	267.0	8.4	801.0	129924062	128153726
88.	23 OPT/E/OS/1	23 OPT/E	1st	KTCN	yes	2.1	107.0	6.0	802.5	133209708	124549819
89.	23 OPT/E/OS/2	23 OPT/E	2nd	KTCN	yes	2.2	190.0	7.6	798.0	143470684	117858697
90.	23 OPT/E/OS/3	23 OPT/E	3rd	KTCN	yes	2.1	310.0	7.5	806.0	154154835	128119536
91.	24 OPT/E/OS/1	24 OPT/E	1st	KTCN	yes	2.1	53.0	8.6	530.0	105221435	101642719
92.	24 OPT/E/OS/2	24 OPT/E	2nd	KTCN	yes	2.1	104.0	8.7	800.8	165460161	159234820
93.	24 OPT/E/OS/3	24 OPT/E	3rd	KTCN	yes	2.1	210.0	8.9	798.0	112938965	108828385
94.	25 OPT/E/OS/1	25 OPT/E	1st	KTCN	yes	2.1	105.0	8.3	798.0	106723783	102092614
95.	25 OPT/E/OS/2	25 OPT/E	2nd	KTCN	yes	2.1	148.0	8.3	799.2	155082523	149379800
96.	25 OPT/E/OS/3	25 OPT/E	3rd	KTCN	yes	2.1	216.0	8.4	799.2	151030395	133196145
97.	29 OPT/E/OD/1	29 OPT/E	1st	KTCN	yes	2.1	179.0	8.4	805.5	165862217	159175765
98.	29 OPT/E/OD/2	29 OPT/E	2nd	KTCN	yes	2.1	242.0	8.4	798.6	129730801	123386448
99.	29 OPT/E/OD/3	29 OPT/E	3rd	KTCN	yes	2.1	374.0	8.4	785.4	123871520	120197464
100.	3 OPT/E/OS/1	3 OPT/E	1st	KTCN	yes	2.2	232.0	6.2	812.0	135957661	130613500
101.	3 OPT/E/OS/2	3 OPT/E	2nd	KTCN	yes	2.2	167.0	4.6	801.6	119848911	111107700
102.	3 OPT/E/OS/3	3 OPT/E	3rd	KTCN	yes	2.2	308.0	7.1	800.8	131219310	108786108
103.	32 OPT/E/OS/1	32 OPT/E	1st	KTCN	yes	2.1	97.0	8.4	795.4	129466985	125407374
104.	32 OPT/E/OS/2	32 OPT/E	2nd	KTCN	yes	2.1	581.0	8.1	813.4	163469075	157827889
105.	32 OPT/E/OS/3	32 OPT/E	3rd	KTCN	yes	2.1	200.0	7.3	800.0	184640247	151208331
106.	33 OPT/E/OD/1	33 OPT/E	1st	KTCN	yes	2.0	97.0	7.9	795.4	123422648	118201384
107.	33 OPT/E/OD/2	33 OPT/E	2nd	KTCN	yes	2.1	289.0	7.8	809.2	110812075	102996005
108.	33 OPT/E/OD/3	33 OPT/E	3rd	KTCN	yes	2.1	400.0	7.8	800.0	132119298	126755659
109.	34 OPT/E/OS/1	34 OPT/E	1st	KTCN	yes	2.1	125.0	8.3	800.0	151407777	116310016
110.	34 OPT/E/OS/2	34 OPT/E	2nd	KTCN	yes	2.1	123.0	8.0	799.5	138603388	132309767
111.	34 OPT/E/OS/3	34 OPT/E	3rd	KTCN	yes	2.1	323.0	8.4	807.5	111661360	108676316
112.	35 OPT/E/OD/1	35 OPT/E	1st	KTCN	yes	2.1	64.0	8.0	640.0	144664234	130659935
113.	35 OPT/E/OD/2	35 OPT/E	2nd	KTCN	yes	2.1	152.0	7.9	805.6	106291087	103063963

114.	35 OPT/E/OD/3	35 OPT/E	3rd	KTCN	yes	2.1	499.0	7.9	798.4	105540385	103730254
115.	64 OPT/E/OS/1	64 OPT/E	1st	KTCN	yes	2.0	53.0	8.5	530.0	139022888	136368954
116.	64 OPT/E/OS/2	64 OPT/E	2nd	KTCN	yes	2.0	120.0	8.7	804.0	158385386	155675331
117.	64 OPT/E/OS/3	64 OPT/E	3rd	KTCN	yes	2.1	301.0	8.6	812.7	64309504	61992139
118.	67 OPT/E/OD/1	67 OPT/E	1st	KTCN	yes	1.9	37.0	9.5	370.0	67313180	65232976
119.	67 OPT/E/OD/2	67 OPT/E	2nd	KTCN	yes	2.0	69.0	9.1	690.0	154180946	125134295
120.	67 OPT/E/OD/3	67 OPT/E	3rd	KTCN	yes	2.1	224.0	8.7	806.4	55789707	61944160
121.	7 OPT/E/OS/1	7 OPT/E	1st	KTCN	no	2.1	58.0	6.3	290.0	137752045	125242097
122.	7 OPT/E/OS/2	7 OPT/E	2nd	KTCN	no	2.4	37.0	5.0	148.0	119032787	113674999
123.	7 OPT/E/OS/3	7 OPT/E	3rd	KTCN	no	2.1	344.0	7.1	791.2	121655860	117974057
124.	75 OPT/E/OS/1	75 OPT/E	1st	KTCN	yes	2.0	73.0	9.1	730.0	74075202	68224680
125.	75 OPT/E/OS/2	75 OPT/E	2nd	KTCN	yes	2.0	69.0	9.1	690.0	83963145	79249196
126.	75 OPT/E/OS/3	75 OPT/E	3rd	KTCN	yes	2.0	130.0	8.9	806.0	85125789	80744063
127.	80 OPT/E/OD/1	80 OPT/E	1st	KTCN	yes	2.0	134.0	9.1	804.0	88668215	64772911
128.	80 OPT/E/OD/2	80 OPT/E	2nd	KTCN	yes	2.0	169.0	9.1	760.5	89022829	48573601
129.	80 OPT/E/OD/3	80 OPT/E	3rd	KTCN	yes	2.1	312.0	8.9	811.2	87859349	83740139
130.	82 OPT/E/OD/1	82 OPT/E	1st	KTCN	yes	2.0	79.0	8.8	790.0	67315303	63491484
131.	82 OPT/E/OD/2	82 OPT/E	2nd	KTCN	yes	2.0	121.0	8.9	798.6	67967216	61037953
132.	82 OPT/E/OD/3	82 OPT/E	3rd	KTCN	yes	2.0	66.0	9.0	660.0	168597319	161125195
133.	9 OPT/E/OS/1	9 OPT/E	1st	KTCN	yes	2.1	111.0	6.5	799.2	148030333	143077513
134.	9 OPT/E/OS/2	9 OPT/E	2nd	KTCN	yes	2.1	98.0	6.8	803.6	132649024	111679824
135.	9 OPT/E/OS/3	9 OPT/E	3rd	KTCN	yes	2.1	312.0	6.3	811.2	149679722	142956889

Abbreviations: CE – corneal epithelium, *TR – topographic region*, RIN – RNA Integrity Number, post-LVC ectasia – post-laser vision correction ectasia, KTCN – keratoconus.

Table S5. Summary of differentially expressed genes (DEGs).

The table presents the total number of upregulated and downregulated genes identified in each comparison, stratified by subgroup, sex, and *topographic region* (*TR*) of the corneal epithelium (CE).

	Comparison	*central TR*	*middle TR*	*peripheral TR*
DOWNREGULATED GENS	post-LVC males and females *vs* controls males and females	103 (98 protein-coding)	4 (4 protein-coding)	501 (411 protein-coding)
	post-LVC males *vs* controls males	2 (0 protein-coding)	0 (0 protein-coding)	0 (0 protein-coding)
	post-LVC females *vs* controls females	3 (2 protein-coding)	1 (1 protein-coding)	5 (3 protein-coding)
	post-LVC males and females *vs* KTCN males and females	0 (0 protein-coding)	0 (0 protein-coding)	7 (0 protein-coding)
	post-LVC males *vs* KTCN males	0 (0 protein-coding)	0 (0 protein-coding)	0 (0 protein-coding)
	post-LVC females *vs* KTCN females	-	-	-
UPREGULATED GENES	post-LVC males and females *vs* controls, males and females	60 (59 protein-coding)	4 (2 protein-coding)	580 (197 protein-coding)
	post-LVC males *vs* controls males	2 (2 protein-coding)	0 (0 protein-coding)	0 (0 protein-coding)
	post-LVC females *vs* controls females	11 (8 protein-coding)	2 (1 protein-coding)	18 (15 protein-coding)
	post-LVC males and females *vs* KTCN males and females	0 (0 protein-coding)	0 (0 protein-coding)	0 (0 protein-coding)
	post-LVC males *vs* KTCN males	0 (0 protein-coding)	0 (0 protein-coding)	0 (0 protein-coding)
	post-LVC females *vs* KTCN females	-	-	-

Abbreviations: KTCN – keratoconus; post-LVC ectasia – post-laser vision correction ectasia; *TR – central topographic region*,

Table S6. Protein-coding differentially expressed genes (DEGs) in post-LVC ectasia.
The DEGs in the particular *TR* of the CE of patients with post-LVC ectasia compared to adequate *TR* of the CE of control individuals. The presented results concern only protein-coding genes. The compared *topographic regions* with the sex of included patients, Ensembl gene identifier ('Gene_id'), gene name, log2FC, and adjusted p-value are indicated. DEGs for all types of classes/biotypes of transcripts are shared in Mendeley Data Repository (doi: 10.17632/p656wtzjv8.1).

Comparison	Gene_id	Gene_name	Log2FC	Adjusted p-value
post-LVC ectasia *vs* CONTROL_*1stTR*_female	ENSG00000167916	*KRT24*	-5.0783	<0.0001
post-LVC ectasia *vs* CONTROL_*1stTR*_female	ENSG00000100427	*MLC1*	4.7202	0.0363
post-LVC ectasia *vs* CONTROL_*1stTR*_female	ENSG00000167754	*KLK5*	2.9742	0.0012
post-LVC ectasia *vs* CONTROL_*1stTR*_female	ENSG00000129455	*KLK8*	2.4042	0.0012
post-LVC ectasia *vs* CONTROL_*1stTR*_female	ENSG00000184489	*PTP4A3*	1.7622	0.0027
post-LVC ectasia *vs* CONTROL_*1stTR*_female	ENSG00000166402	*TUB*	1.5526	0.0483
post-LVC ectasia *vs* CONTROL_*1stTR*_female	ENSG00000198113	*TOR4A*	1.4066	0.0094
post-LVC ectasia *vs* CONTROL_*1stTR*_female	ENSG00000111981	*ULBP1*	1.3201	0.0483
post-LVC ectasia *vs* CONTROL_*1stTR*_female	ENSG00000168350	*DEGS2*	1.1646	0.0204
post-LVC ectasia *vs* CONTROL_*1stTR*_female	ENSG00000198911	*SREBF2*	-0.7592	0.0070
post-LVC ectasia *vs* CONTROL_*1stTR*_female, male	ENSG00000163618	*CADPS*	-2.3578	0.0044
post-LVC ectasia *vs* CONTROL_*1stTR*_female, male	ENSG00000205002	*AARD*	-1.6866	0.0323
post-LVC ectasia *vs* CONTROL_*1stTR*_female, male	ENSG00000175699	*CCDC197*	-1.6848	0.0474
post-LVC ectasia *vs* CONTROL_*1stTR*_female, male	ENSG00000080854	*IGSF9B*	-1.4161	0.0323
post-LVC ectasia *vs* CONTROL_*1stTR*_female, male	ENSG00000130052	*STARD8*	-1.3741	0.0442
post-LVC ectasia *vs* CONTROL_*1stTR*_female, male	ENSG00000164330	*EBF1*	-1.3183	0.0442
post-LVC ectasia *vs* CONTROL_*1stTR*_female, male	ENSG00000072080	*SPP2*	-1.3182	0.0467
post-LVC ectasia *vs* CONTROL_*1stTR*_female, male	ENSG00000142611	*PRDM16*	-1.3175	0.0474
post-LVC ectasia *vs* CONTROL_*1stTR*_female, male	ENSG00000091972	*CD200*	-1.2002	0.0453
post-LVC ectasia *vs* CONTROL_*1stTR*_female, male	ENSG00000197168	*NEK5*	-1.1373	0.0442
post-LVC ectasia *vs* CONTROL_*1stTR*_female, male	ENSG00000100276	*RASL10A*	-1.1320	0.0353
post-LVC ectasia *vs* CONTROL_*1stTR*_female, male	ENSG00000152049	*KCNE4*	-1.0930	0.0483
post-LVC ectasia *vs* CONTROL_*1stTR*_female, male	ENSG00000013297	*CLDN11*	-1.0896	0.0429
post-LVC ectasia *vs* CONTROL_*1stTR*_female, male	ENSG00000047457	*CP*	-1.0625	0.0362
post-LVC ectasia *vs* CONTROL_*1stTR*_female, male	ENSG00000180616	*SSTR2*	-1.0431	0.0442
post-LVC ectasia *vs* CONTROL_*1stTR*_female, male	ENSG00000055732	*MCOLN3*	-0.9909	0.0255
post-LVC ectasia *vs* CONTROL_*1stTR*_female, male	ENSG00000164776	*PHKG1*	-0.9233	0.0179
post-LVC ectasia *vs* CONTROL_*1stTR*_female, male	ENSG00000206538	*VGLL3*	-0.8963	0.0474
post-LVC ectasia *vs* CONTROL_*1stTR*_female, male	ENSG00000127152	*BCL11B*	-0.8952	0.0442
post-LVC ectasia *vs* CONTROL_*1stTR*_female, male	ENSG00000143127	*ITGA10*	-0.8917	0.0365
post-LVC ectasia *vs* CONTROL_*1stTR*_female, male	ENSG00000138758	*SEPTIN11*	-0.8592	0.0365
post-LVC ectasia *vs* CONTROL_*1stTR*_female, male	ENSG00000275395	*FCGBP*	-0.8478	0.0483
post-LVC ectasia *vs* CONTROL_*1stTR*_female, male	ENSG00000137261	*KIAA0319*	-0.8271	0.0429
post-LVC ectasia *vs* CONTROL_*1stTR*_female, male	ENSG00000188641	*DPYD*	-0.8259	0.0315
post-LVC ectasia *vs* CONTROL_*1stTR*_female, male	ENSG00000137573	*SULF1*	-0.8080	0.0453
post-LVC ectasia *vs* CONTROL_*1stTR*_female, male	ENSG00000166257	*SCN3B*	-0.8050	0.0255
post-LVC ectasia *vs* CONTROL_*1stTR*_female, male	ENSG00000161405	*IKZF3*	-0.8012	0.0433
post-LVC ectasia *vs* CONTROL_*1stTR*_female, male	ENSG00000005187	*ACSM3*	-0.7736	0.0429
post-LVC ectasia *vs* CONTROL_*1stTR*_female, male	ENSG00000133958	*UNC79*	-0.7662	0.0413
post-LVC ectasia *vs* CONTROL_*1stTR*_female, male	ENSG00000286112	*AL441992.3*	-0.7236	0.0480
post-LVC ectasia *vs* CONTROL_*1stTR*_female, male	ENSG00000157388	*CACNA1D*	-0.7081	0.0442
post-LVC ectasia *vs* CONTROL_*1stTR*_female, male	ENSG00000121440	*PDZRN3*	-0.6992	0.0349
post-LVC ectasia *vs* CONTROL_*1stTR*_female, male	ENSG00000206530	*CFAP44*	-0.6847	0.0442
post-LVC ectasia *vs* CONTROL_*1stTR*_female, male	ENSG00000177096	*PHETA2*	0.5915	0.0327
post-LVC ectasia *vs* CONTROL_*1stTR*_female, male	ENSG00000170043	*TRAPPC1*	0.6016	0.0442
post-LVC ectasia *vs* CONTROL_*1stTR*_female, male	ENSG00000172936	*MYD88*	0.6022	0.0453
post-LVC ectasia *vs* CONTROL_*1stTR*_female, male	ENSG00000100348	*TXN2*	0.6090	0.0489

post-LVC ectasia *vs* CONTROL_*1stTR*_female, male	ENSG00000116711	*PLA2G4A*	0.6202	0.0348
post-LVC ectasia *vs* CONTROL_*1stTR*_female, male	ENSG00000117308	*GALE*	0.6219	0.0323
post-LVC ectasia *vs* CONTROL_*1stTR*_female, male	ENSG00000181218	*H2AW*	0.6445	0.0451
post-LVC ectasia *vs* CONTROL_*1stTR*_female, male	ENSG00000143653	*SCCPDH*	0.6596	0.0304
post-LVC ectasia *vs* CONTROL_*1stTR*_female, male	ENSG00000139890	*REM2*	0.6926	0.0429
post-LVC ectasia *vs* CONTROL_*1stTR*_female, male	ENSG00000175600	*SUGCT*	0.6985	0.0414
post-LVC ectasia *vs* CONTROL_*1stTR*_female, male	ENSG00000100823	*APEX1*	0.7127	0.0439
post-LVC ectasia *vs* CONTROL_*1stTR*_female, male	ENSG00000143162	*CREG1*	0.7132	0.0327
post-LVC ectasia *vs* CONTROL_*1stTR*_female, male	ENSG00000205220	*PSMB10*	0.7434	0.0474
post-LVC ectasia *vs* CONTROL_*1stTR*_female, male	ENSG00000101400	*SNTA1*	0.7454	0.0255
post-LVC ectasia *vs* CONTROL_*1stTR*_female, male	ENSG00000167601	*AXL*	0.7460	0.0453
post-LVC ectasia *vs* CONTROL_*1stTR*_female, male	ENSG00000005243	*COPZ2*	0.7483	0.0323
post-LVC ectasia *vs* CONTROL_*1stTR*_female, male	ENSG00000106992	*AK1*	0.7538	0.0323
post-LVC ectasia *vs* CONTROL_*1stTR*_female, male	ENSG00000112667	*DNPH1*	0.7636	0.0442
post-LVC ectasia *vs* CONTROL_*1stTR*_female, male	ENSG00000243056	*EIF4EBP3*	0.7693	0.0474
post-LVC ectasia *vs* CONTROL_*1stTR*_female, male	ENSG00000109861	*CTSC*	0.7804	0.0323
post-LVC ectasia *vs* CONTROL_*1stTR*_female, male	ENSG00000110492	*MDK*	0.7819	0.0413
post-LVC ectasia *vs* CONTROL_*1stTR*_female, male	ENSG00000177697	*CD151*	0.7910	0.0488
post-LVC ectasia *vs* CONTROL_*1stTR*_female, male	ENSG00000189334	*S100A14*	0.8008	0.0179
post-LVC ectasia *vs* CONTROL_*1stTR*_female, male	ENSG00000144485	*HES6*	0.8016	0.0474
post-LVC ectasia *vs* CONTROL_*1stTR*_female, male	ENSG00000147100	*SLC16A2*	0.8174	0.0442
post-LVC ectasia *vs* CONTROL_*1stTR*_female, male	ENSG00000051523	*CYBA*	0.8225	0.0439
post-LVC ectasia *vs* CONTROL_*1stTR*_female, male	ENSG00000105327	*BBC3*	0.8368	0.0323
post-LVC ectasia *vs* CONTROL_*1stTR*_female, male	ENSG00000127399	*LRRC61*	0.8463	0.0456
post-LVC ectasia *vs* CONTROL_*1stTR*_female, male	ENSG00000147804	*SLC39A4*	0.8540	0.0429
post-LVC ectasia *vs* CONTROL_*1stTR*_female, male	ENSG00000231500	*RPS18*	0.8861	0.0372
post-LVC ectasia *vs* CONTROL_*1stTR*_female, male	ENSG00000110852	*CLEC2B*	0.8910	0.0315
post-LVC ectasia *vs* CONTROL_*1stTR*_female, male	ENSG00000276045	*ORAI1*	0.8959	0.0149
post-LVC ectasia *vs* CONTROL_*1stTR*_female, male	ENSG00000102359	*SRPX2*	0.9002	0.0413
post-LVC ectasia *vs* CONTROL_*1stTR*_female, male	ENSG00000123143	*PKN1*	0.9023	0.0315
post-LVC ectasia *vs* CONTROL_*1stTR*_female, male	ENSG00000003137	*CYP26B1*	0.9043	0.0442
post-LVC ectasia *vs* CONTROL_*1stTR*_female, male	ENSG00000198113	*TOR4A*	0.9353	0.0323
post-LVC ectasia *vs* CONTROL_*1stTR*_female, male	ENSG00000133124	*IRS4*	0.9482	0.0323
post-LVC ectasia *vs* CONTROL_*1stTR*_female, male	ENSG00000173825	*TIGD3*	0.9580	0.0429
post-LVC ectasia *vs* CONTROL_*1stTR*_female, male	ENSG00000235169	*SMIM1*	0.9757	0.0315
post-LVC ectasia *vs* CONTROL_*1stTR*_female, male	ENSG00000102575	*ACP5*	0.9954	0.0365
post-LVC ectasia *vs* CONTROL_*1stTR*_female, male	ENSG00000073756	*PTGS2*	1.0541	0.0474
post-LVC ectasia *vs* CONTROL_*1stTR*_female, male	ENSG00000147465	*STAR*	1.0575	0.0453
post-LVC ectasia *vs* CONTROL_*1stTR*_female, male	ENSG00000160932	*LY6E*	1.0620	0.0315
post-LVC ectasia *vs* CONTROL_*1stTR*_female, male	ENSG00000240891	*PLCXD2*	1.0801	0.0323
post-LVC ectasia *vs* CONTROL_*1stTR*_female, male	ENSG00000165716	*DIPK1B*	1.4687	0.0489
post-LVC ectasia *vs* CONTROL_*1stTR*_female, male	ENSG00000070886	*EPHA8*	1.8424	0.0179
post-LVC ectasia *vs* CONTROL_*1stTR*_female, male	ENSG00000163431	*LMOD1*	2.2471	0.0179
post-LVC ectasia *vs* CONTROL_*1stTR*_male	ENSG00000189334	*S100A14*	0.8446	0.0384
post-LVC ectasia *vs* CONTROL_*1stTR*_male	ENSG00000168016	*TRANK1*	0.8483	0.0273
post-LVC ectasia *vs* CONTROL_*2ndTR*_female	ENSG00000167916	*KRT24*	-5.3154	<0.0001
post-LVC ectasia *vs* CONTROL_*2ndTR*_female	ENSG00000167754	*KLK5*	2.7653	0.0069
post-LVC ectasia *vs* CONTROL_*2ndTR*_female, male	ENSG00000163618	*CADPS*	-2.0253	0.0212
post-LVC ectasia *vs* CONTROL_*2ndTR*_female, male	ENSG00000080854	*IGSF9B*	-1.6538	0.0212
post-LVC ectasia *vs* CONTROL_*2ndTR*_female, male	ENSG00000198768	*APCDD1L*	-0.9637	0.0212
post-LVC ectasia *vs* CONTROL_*2ndTR*_female, male	ENSG00000205560	*CPT1B*	-0.8508	0.0212
post-LVC ectasia *vs* CONTROL_*2ndTR*_female, male	ENSG00000110852	*CLEC2B*	0.9819	0.0212
post-LVC ectasia *vs* CONTROL_*2ndTR*_female, male	ENSG00000070886	*EPHA8*	1.9033	0.0212
post-LVC ectasia *vs* CONTROL_*3rdTR*_female	ENSG00000167916	*KRT24*	-5.4892	<0.0001
post-LVC ectasia *vs* CONTROL_*3rdTR*_female	ENSG00000196912	*ANKRD36B*	-0.9258	0.0322
post-LVC ectasia *vs* CONTROL_*3rdTR*_female	ENSG00000198911	*SREBF2*	-0.6292	0.0375
post-LVC ectasia *vs* CONTROL_*3rdTR*_female	ENSG00000137936	*BCAR3*	0.7569	0.0460
post-LVC ectasia *vs* CONTROL_*3rdTR*_female	ENSG00000197417	*SHPK*	0.8199	0.0232
post-LVC ectasia *vs* CONTROL_*3rdTR*_female	ENSG00000008323	*PLEKHG6*	0.8683	0.0232

post-LVC ectasia *vs* CONTROL_*3rdTR*_female	ENSG00000131941	*RHPN2*	0.8902	0.0234
post-LVC ectasia *vs* CONTROL_*3rdTR*_female	ENSG00000172183	*ISG20*	0.9446	0.0232
post-LVC ectasia *vs* CONTROL_*3rdTR*_female	ENSG00000111981	*ULBP1*	1.3070	0.0459
post-LVC ectasia *vs* CONTROL_*3rdTR*_female	ENSG00000168350	*DEGS2*	1.3533	0.0067
post-LVC ectasia *vs* CONTROL_*3rdTR*_female	ENSG00000276045	*ORAI1*	1.4113	0.0350
post-LVC ectasia *vs* CONTROL_*3rdTR*_female	ENSG00000184489	*PTP4A3*	1.4682	0.0314
post-LVC ectasia *vs* CONTROL_*3rdTR*_female	ENSG00000177697	*CD151*	1.5303	0.0365
post-LVC ectasia *vs* CONTROL_*3rdTR*_female	ENSG00000175600	*SUGCT*	1.5356	0.0067
post-LVC ectasia *vs* CONTROL_*3rdTR*_female	ENSG00000187624	*C17orf97*	1.5820	0.0033
post-LVC ectasia *vs* CONTROL_*3rdTR*_female	ENSG00000072041	*SLC6A15*	1.6516	0.0375
post-LVC ectasia *vs* CONTROL_*3rdTR*_female	ENSG00000129455	*KLK8*	2.3111	0.0067
post-LVC ectasia *vs* CONTROL_*3rdTR*_female	ENSG00000167754	*KLK5*	3.0519	0.0015
post-LVC ectasia *vs* CONTROL_*3rdTR*_female, male	ENSG00000280893	*AC009133.6*	-3.7780	0.0445
post-LVC ectasia *vs* CONTROL_*3rdTR*_female, male	ENSG00000101204	*CHRNA4*	-3.5820	0.0403
post-LVC ectasia *vs* CONTROL_*3rdTR*_female, male	ENSG00000179796	*LRRC3B*	-3.4805	0.0174
post-LVC ectasia *vs* CONTROL_*3rdTR*_female, male	ENSG00000152583	*SPARCL1*	-3.3063	0.0307
post-LVC ectasia *vs* CONTROL_*3rdTR*_female, male	ENSG00000262655	*SPON1*	-2.7605	0.0454
post-LVC ectasia *vs* CONTROL_*3rdTR*_female, male	ENSG00000164588	*HCN1*	-2.6711	0.0342
post-LVC ectasia *vs* CONTROL_*3rdTR*_female, male	ENSG00000115896	*PLCL1*	-2.6178	0.0296
post-LVC ectasia *vs* CONTROL_*3rdTR*_female, male	ENSG00000196159	*FAT4*	-2.2417	0.0174
post-LVC ectasia *vs* CONTROL_*3rdTR*_female, male	ENSG00000146592	*CREB5*	-2.1030	0.0457
post-LVC ectasia *vs* CONTROL_*3rdTR*_female, male	ENSG00000136928	*GABBR2*	-2.0249	0.0457
post-LVC ectasia *vs* CONTROL_*3rdTR*_female, male	ENSG00000163618	*CADPS*	-2.0206	0.0077
post-LVC ectasia *vs* CONTROL_*3rdTR*_female, male	ENSG00000108231	*LGI1*	-1.9893	0.0342
post-LVC ectasia *vs* CONTROL_*3rdTR*_female, male	ENSG00000105492	*SIGLEC6*	-1.9375	0.0174
post-LVC ectasia *vs* CONTROL_*3rdTR*_female, male	ENSG00000174640	*SLCO2A1*	-1.9155	0.0457
post-LVC ectasia *vs* CONTROL_*3rdTR*_female, male	ENSG00000184347	*SLIT3*	-1.8909	0.0342
post-LVC ectasia *vs* CONTROL_*3rdTR*_female, male	ENSG00000144596	*GRIP2*	-1.8735	0.0293
post-LVC ectasia *vs* CONTROL_*3rdTR*_female, male	ENSG00000137265	*IRF4*	-1.8392	0.0428
post-LVC ectasia *vs* CONTROL_*3rdTR*_female, male	ENSG00000123119	*NECAB1*	-1.7806	0.0353
post-LVC ectasia *vs* CONTROL_*3rdTR*_female, male	ENSG00000158874	*APOA2*	-1.7733	0.0417
post-LVC ectasia *vs* CONTROL_*3rdTR*_female, male	ENSG00000165762	*OR4K2*	-1.7634	0.0254
post-LVC ectasia *vs* CONTROL_*3rdTR*_female, male	ENSG00000112936	*C7*	-1.7445	0.0492
post-LVC ectasia *vs* CONTROL_*3rdTR*_female, male	ENSG00000072657	*TRHDE*	-1.7397	0.0482
post-LVC ectasia *vs* CONTROL_*3rdTR*_female, male	ENSG00000176842	*IRX5*	-1.7041	0.0455
post-LVC ectasia *vs* CONTROL_*3rdTR*_female, male	ENSG00000108825	*PTGES3L-AARSD1*	-1.6688	0.0492
post-LVC ectasia *vs* CONTROL_*3rdTR*_female, male	ENSG00000004846	*ABCB5*	-1.6498	0.0174
post-LVC ectasia *vs* CONTROL_*3rdTR*_female, male	ENSG00000154080	*CHST9*	-1.6454	0.0434
post-LVC ectasia *vs* CONTROL_*3rdTR*_female, male	ENSG00000204930	*FAM221B*	-1.6406	0.0284
post-LVC ectasia *vs* CONTROL_*3rdTR*_female, male	ENSG00000161509	*GRIN2C*	-1.6351	0.0482
post-LVC ectasia *vs* CONTROL_*3rdTR*_female, male	ENSG00000116833	*NR5A2*	-1.6039	0.0384
post-LVC ectasia *vs* CONTROL_*3rdTR*_female, male	ENSG00000149575	*SCN2B*	-1.5988	0.0296
post-LVC ectasia *vs* CONTROL_*3rdTR*_female, male	ENSG00000116675	*DNAJC6*	-1.5845	0.0407
post-LVC ectasia *vs* CONTROL_*3rdTR*_female, male	ENSG00000172986	*GXYLT2*	-1.5181	0.0334
post-LVC ectasia *vs* CONTROL_*3rdTR*_female, male	ENSG00000154310	*TNIK*	-1.5074	0.0290
post-LVC ectasia *vs* CONTROL_*3rdTR*_female, male	ENSG00000120215	*MLANA*	-1.5067	0.0428
post-LVC ectasia *vs* CONTROL_*3rdTR*_female, male	ENSG00000261341	*AC010325.1*	-1.5042	0.0307
post-LVC ectasia *vs* CONTROL_*3rdTR*_female, male	ENSG00000147138	*GPR174*	-1.4776	0.0286
post-LVC ectasia *vs* CONTROL_*3rdTR*_female, male	ENSG00000139915	*MDGA2*	-1.4681	0.0469
post-LVC ectasia *vs* CONTROL_*3rdTR*_female, male	ENSG00000171657	*GPR82*	-1.4651	0.0350
post-LVC ectasia *vs* CONTROL_*3rdTR*_female, male	ENSG00000180432	*CYP8B1*	-1.4573	0.0386
post-LVC ectasia *vs* CONTROL_*3rdTR*_female, male	ENSG00000132840	*BHMT2*	-1.4569	0.0323
post-LVC ectasia *vs* CONTROL_*3rdTR*_female, male	ENSG00000185985	*SLITRK2*	-1.4546	0.0307
post-LVC ectasia *vs* CONTROL_*3rdTR*_female, male	ENSG00000099617	*EFNA2*	-1.4384	0.0397
post-LVC ectasia *vs* CONTROL_*3rdTR*_female, male	ENSG00000196834	*POTEI*	-1.4367	0.0286
post-LVC ectasia *vs* CONTROL_*3rdTR*_female, male	ENSG00000119698	*PPP4R4*	-1.4321	0.0397
post-LVC ectasia *vs* CONTROL_*3rdTR*_female, male	ENSG00000140873	*ADAMTS18*	-1.4288	0.0428
post-LVC ectasia *vs* CONTROL_*3rdTR*_female, male	ENSG00000100433	*KCNK10*	-1.4158	0.0351
post-LVC ectasia *vs* CONTROL_*3rdTR*_female, male	ENSG00000164796	*CSMD3*	-1.4121	0.0302

post-LVC ectasia *vs* CONTROL_*3rdTR*_female, male	ENSG00000123243	*ITIH5*	-1.4119	0.0286
post-LVC ectasia *vs* CONTROL_*3rdTR*_female, male	ENSG00000169291	*SHE*	-1.3885	0.0414
post-LVC ectasia *vs* CONTROL_*3rdTR*_female, male	ENSG00000007171	*NOS2*	-1.3862	0.0286
post-LVC ectasia *vs* CONTROL_*3rdTR*_female, male	ENSG00000242221	*PSG2*	-1.3847	0.0416
post-LVC ectasia *vs* CONTROL_*3rdTR*_female, male	ENSG00000081138	*CDH7*	-1.3833	0.0174
post-LVC ectasia *vs* CONTROL_*3rdTR*_female, male	ENSG00000283288	*SMIM33*	-1.3786	0.0419
post-LVC ectasia *vs* CONTROL_*3rdTR*_female, male	ENSG00000186868	*MAPT*	-1.3775	0.0430
post-LVC ectasia *vs* CONTROL_*3rdTR*_female, male	ENSG00000080854	*IGSF9B*	-1.3640	0.0296
post-LVC ectasia *vs* CONTROL_*3rdTR*_female, male	ENSG00000144642	*RBMS3*	-1.3474	0.0286
post-LVC ectasia *vs* CONTROL_*3rdTR*_female, male	ENSG00000141622	*RNF165*	-1.3390	0.0457
post-LVC ectasia *vs* CONTROL_*3rdTR*_female, male	ENSG00000015592	*STMN4*	-1.3321	0.0419
post-LVC ectasia *vs* CONTROL_*3rdTR*_female, male	ENSG00000105141	*CASP14*	-1.3213	0.0386
post-LVC ectasia *vs* CONTROL_*3rdTR*_female, male	ENSG00000168646	*AXIN2*	-1.3211	0.0468
post-LVC ectasia *vs* CONTROL_*3rdTR*_female, male	ENSG00000071991	*CDH19*	-1.3173	0.0419
post-LVC ectasia *vs* CONTROL_*3rdTR*_female, male	ENSG00000172551	*MUCL1*	-1.3144	0.0488
post-LVC ectasia *vs* CONTROL_*3rdTR*_female, male	ENSG00000182613	*OR2V2*	-1.3138	0.0368
post-LVC ectasia *vs* CONTROL_*3rdTR*_female, male	ENSG00000134061	*CD180*	-1.3114	0.0400
post-LVC ectasia *vs* CONTROL_*3rdTR*_female, male	ENSG00000157542	*KCNJ6*	-1.2836	0.0357
post-LVC ectasia *vs* CONTROL_*3rdTR*_female, male	ENSG00000153993	*SEMA3D*	-1.2752	0.0387
post-LVC ectasia *vs* CONTROL_*3rdTR*_female, male	ENSG00000130035	*GALNT8*	-1.2654	0.0364
post-LVC ectasia *vs* CONTROL_*3rdTR*_female, male	ENSG00000221970	*OR2A1*	-1.2589	0.0412
post-LVC ectasia *vs* CONTROL_*3rdTR*_female, male	ENSG00000074047	*GLI2*	-1.2523	0.0457
post-LVC ectasia *vs* CONTROL_*3rdTR*_female, male	ENSG00000176049	*JAKMIP2*	-1.2468	0.0469
post-LVC ectasia *vs* CONTROL_*3rdTR*_female, male	ENSG00000205922	*ONECUT3*	-1.2438	0.0408
post-LVC ectasia *vs* CONTROL_*3rdTR*_female, male	ENSG00000112769	*LAMA4*	-1.2412	0.0463
post-LVC ectasia *vs* CONTROL_*3rdTR*_female, male	ENSG00000187955	*COL14A1*	-1.2409	0.0380
post-LVC ectasia *vs* CONTROL_*3rdTR*_female, male	ENSG00000136929	*HEMGN*	-1.2375	0.0457
post-LVC ectasia *vs* CONTROL_*3rdTR*_female, male	ENSG00000107242	*PIP5K1B*	-1.2287	0.0254
post-LVC ectasia *vs* CONTROL_*3rdTR*_female, male	ENSG00000165478	*HEPACAM*	-1.2245	0.0469
post-LVC ectasia *vs* CONTROL_*3rdTR*_female, male	ENSG00000049540	*ELN*	-1.2196	0.0350
post-LVC ectasia *vs* CONTROL_*3rdTR*_female, male	ENSG00000106278	*PTPRZ1*	-1.2191	0.0338
post-LVC ectasia *vs* CONTROL_*3rdTR*_female, male	ENSG00000141469	*SLC14A1*	-1.2121	0.0305
post-LVC ectasia *vs* CONTROL_*3rdTR*_female, male	ENSG00000151090	*THRB*	-1.1991	0.0428
post-LVC ectasia *vs* CONTROL_*3rdTR*_female, male	ENSG00000154330	*PGM5*	-1.1956	0.0286
post-LVC ectasia *vs* CONTROL_*3rdTR*_female, male	ENSG00000086991	*NOX4*	-1.1953	0.0174
post-LVC ectasia *vs* CONTROL_*3rdTR*_female, male	ENSG00000259417	*CTXND1*	-1.1922	0.0243
post-LVC ectasia *vs* CONTROL_*3rdTR*_female, male	ENSG00000151650	*VENTX*	-1.1780	0.0327
post-LVC ectasia *vs* CONTROL_*3rdTR*_female, male	ENSG00000077782	*FGFR1*	-1.1778	0.0404
post-LVC ectasia *vs* CONTROL_*3rdTR*_female, male	ENSG00000173626	*TRAPPC3L*	-1.1654	0.0484
post-LVC ectasia *vs* CONTROL_*3rdTR*_female, male	ENSG00000196628	*TCF4*	-1.1597	0.0457
post-LVC ectasia *vs* CONTROL_*3rdTR*_female, male	ENSG00000037280	*FLT4*	-1.1593	0.0379
post-LVC ectasia *vs* CONTROL_*3rdTR*_female, male	ENSG00000183484	*GPR132*	-1.1584	0.0326
post-LVC ectasia *vs* CONTROL_*3rdTR*_female, male	ENSG00000133067	*LGR6*	-1.1564	0.0445
post-LVC ectasia *vs* CONTROL_*3rdTR*_female, male	ENSG00000147724	*FAM135B*	-1.1537	0.0470
post-LVC ectasia *vs* CONTROL_*3rdTR*_female, male	ENSG00000135903	*PAX3*	-1.1514	0.0307
post-LVC ectasia *vs* CONTROL_*3rdTR*_female, male	ENSG00000127533	*F2RL3*	-1.1416	0.0296
post-LVC ectasia *vs* CONTROL_*3rdTR*_female, male	ENSG00000047662	*FAM184B*	-1.1411	0.0479
post-LVC ectasia *vs* CONTROL_*3rdTR*_female, male	ENSG00000183317	*EPHA10*	-1.1402	0.0457
post-LVC ectasia *vs* CONTROL_*3rdTR*_female, male	ENSG00000124772	*CPNE5*	-1.1288	0.0350
post-LVC ectasia *vs* CONTROL_*3rdTR*_female, male	ENSG00000196381	*ZNF781*	-1.1228	0.0372
post-LVC ectasia *vs* CONTROL_*3rdTR*_female, male	ENSG00000162782	*TDRD5*	-1.1141	0.0286
post-LVC ectasia *vs* CONTROL_*3rdTR*_female, male	ENSG00000167208	*SNX20*	-1.1135	0.0349
post-LVC ectasia *vs* CONTROL_*3rdTR*_female, male	ENSG00000143512	*HHIPL2*	-1.1104	0.0293
post-LVC ectasia *vs* CONTROL_*3rdTR*_female, male	ENSG00000126262	*FFAR2*	-1.1038	0.0473
post-LVC ectasia *vs* CONTROL_*3rdTR*_female, male	ENSG00000163380	*LMOD3*	-1.1018	0.0386
post-LVC ectasia *vs* CONTROL_*3rdTR*_female, male	ENSG00000171016	*PYGO1*	-1.1005	0.0342
post-LVC ectasia *vs* CONTROL_*3rdTR*_female, male	ENSG00000110777	*POU2AF1*	-1.0966	0.0473
post-LVC ectasia *vs* CONTROL_*3rdTR*_female, male	ENSG00000154099	*DNAAF1*	-1.0959	0.0387
post-LVC ectasia *vs* CONTROL_*3rdTR*_female, male	ENSG00000144285	*SCN1A*	-1.0920	0.0386

post-LVC ectasia *vs* CONTROL_*3rdTR*_female, male	ENSG00000164287	*CDC20B*	-1.0829	0.0476
post-LVC ectasia *vs* CONTROL_*3rdTR*_female, male	ENSG00000127325	*BEST3*	-1.0819	0.0400
post-LVC ectasia *vs* CONTROL_*3rdTR*_female, male	ENSG00000113263	*ITK*	-1.0780	0.0412
post-LVC ectasia *vs* CONTROL_*3rdTR*_female, male	ENSG00000273540	*AGBL1*	-1.0662	0.0443
post-LVC ectasia *vs* CONTROL_*3rdTR*_female, male	ENSG00000172578	*KLHL6*	-1.0648	0.0342
post-LVC ectasia *vs* CONTROL_*3rdTR*_female, male	ENSG00000165409	*TSHR*	-1.0610	0.0441
post-LVC ectasia *vs* CONTROL_*3rdTR*_female, male	ENSG00000154162	*CDH12*	-1.0594	0.0457
post-LVC ectasia *vs* CONTROL_*3rdTR*_female, male	ENSG00000115165	*CYTIP*	-1.0584	0.0399
post-LVC ectasia *vs* CONTROL_*3rdTR*_female, male	ENSG00000140795	*MYLK3*	-1.0454	0.0386
post-LVC ectasia *vs* CONTROL_*3rdTR*_female, male	ENSG00000130635	*COL5A1*	-1.0453	0.0481
post-LVC ectasia *vs* CONTROL_*3rdTR*_female, male	ENSG00000128606	*LRRC17*	-1.0437	0.0457
post-LVC ectasia *vs* CONTROL_*3rdTR*_female, male	ENSG00000061337	*LZTS1*	-1.0422	0.0457
post-LVC ectasia *vs* CONTROL_*3rdTR*_female, male	ENSG00000126217	*MCF2L*	-1.0399	0.0344
post-LVC ectasia *vs* CONTROL_*3rdTR*_female, male	ENSG00000184226	*PCDH9*	-1.0367	0.0491
post-LVC ectasia *vs* CONTROL_*3rdTR*_female, male	ENSG00000184261	*KCNK12*	-1.0348	0.0428
post-LVC ectasia *vs* CONTROL_*3rdTR*_female, male	ENSG00000172519	*OR10H5*	-1.0340	0.0350
post-LVC ectasia *vs* CONTROL_*3rdTR*_female, male	ENSG00000164106	*SCRG1*	-1.0255	0.0428
post-LVC ectasia *vs* CONTROL_*3rdTR*_female, male	ENSG00000275395	*FCGBP*	-1.0232	0.0284
post-LVC ectasia *vs* CONTROL_*3rdTR*_female, male	ENSG00000183206	*POTEC*	-1.0217	0.0383
post-LVC ectasia *vs* CONTROL_*3rdTR*_female, male	ENSG00000117020	*AKT3*	-1.0212	0.0419
post-LVC ectasia *vs* CONTROL_*3rdTR*_female, male	ENSG00000114349	*GNAT1*	-1.0211	0.0468
post-LVC ectasia *vs* CONTROL_*3rdTR*_female, male	ENSG00000165995	*CACNB2*	-1.0152	0.0463
post-LVC ectasia *vs* CONTROL_*3rdTR*_female, male	ENSG00000111666	*CHPT1*	-0.9997	0.0364
post-LVC ectasia *vs* CONTROL_*3rdTR*_female, male	ENSG00000165300	*SLITRK5*	-0.9906	0.0387
post-LVC ectasia *vs* CONTROL_*3rdTR*_female, male	ENSG00000158258	*CLSTN2*	-0.9841	0.0415
post-LVC ectasia *vs* CONTROL_*3rdTR*_female, male	ENSG00000198768	*APCDD1L*	-0.9829	0.0069
post-LVC ectasia *vs* CONTROL_*3rdTR*_female, male	ENSG00000174667	*OR7D4*	-0.9793	0.0469
post-LVC ectasia *vs* CONTROL_*3rdTR*_female, male	ENSG00000181690	*PLAG1*	-0.9718	0.0174
post-LVC ectasia *vs* CONTROL_*3rdTR*_female, male	ENSG00000221836	*OR2A5*	-0.9704	0.0457
post-LVC ectasia *vs* CONTROL_*3rdTR*_female, male	ENSG00000006118	*TMEM132A*	-0.9591	0.0313
post-LVC ectasia *vs* CONTROL_*3rdTR*_female, male	ENSG00000222036	*POTEM*	-0.9587	0.0286
post-LVC ectasia *vs* CONTROL_*3rdTR*_female, male	ENSG00000233024	*NPIPA9*	-0.9559	0.0007
post-LVC ectasia *vs* CONTROL_*3rdTR*_female, male	ENSG00000181718	*OR5T2*	-0.9396	0.0370
post-LVC ectasia *vs* CONTROL_*3rdTR*_female, male	ENSG00000036530	*CYP46A1*	-0.9395	0.0441
post-LVC ectasia *vs* CONTROL_*3rdTR*_female, male	ENSG00000198535	*C2CD4A*	-0.9356	0.0386
post-LVC ectasia *vs* CONTROL_*3rdTR*_female, male	ENSG00000134138	*MEIS2*	-0.9322	0.0205
post-LVC ectasia *vs* CONTROL_*3rdTR*_female, male	ENSG00000251287	*ALG1L2*	-0.9246	0.0489
post-LVC ectasia *vs* CONTROL_*3rdTR*_female, male	ENSG00000122188	*LAX1*	-0.9174	0.0326
post-LVC ectasia *vs* CONTROL_*3rdTR*_female, male	ENSG00000124092	*CTCFL*	-0.9169	0.0428
post-LVC ectasia *vs* CONTROL_*3rdTR*_female, male	ENSG00000175344	*CHRNA7*	-0.9105	0.0478
post-LVC ectasia *vs* CONTROL_*3rdTR*_female, male	ENSG00000173281	*PPP1R3B*	-0.9049	0.0441
post-LVC ectasia *vs* CONTROL_*3rdTR*_female, male	ENSG00000112183	*RBM24*	-0.8978	0.0334
post-LVC ectasia *vs* CONTROL_*3rdTR*_female, male	ENSG00000167100	*SAMD14*	-0.8929	0.0463
post-LVC ectasia *vs* CONTROL_*3rdTR*_female, male	ENSG00000172602	*RND1*	-0.8896	0.0412
post-LVC ectasia *vs* CONTROL_*3rdTR*_female, male	ENSG00000172324	*OR5A2*	-0.8725	0.0419
post-LVC ectasia *vs* CONTROL_*3rdTR*_female, male	ENSG00000101347	*SAMHD1*	-0.8688	0.0444
post-LVC ectasia *vs* CONTROL_*3rdTR*_female, male	ENSG00000206077	*ZDHHC11B*	-0.8662	0.0492
post-LVC ectasia *vs* CONTROL_*3rdTR*_female, male	ENSG00000180616	*SSTR2*	-0.8621	0.0471
post-LVC ectasia *vs* CONTROL_*3rdTR*_female, male	ENSG00000100336	*APOL4*	-0.8523	0.0479
post-LVC ectasia *vs* CONTROL_*3rdTR*_female, male	ENSG00000005981	*ASB4*	-0.8517	0.0412
post-LVC ectasia *vs* CONTROL_*3rdTR*_female, male	ENSG00000104888	*SLC17A7*	-0.8453	0.0457
post-LVC ectasia *vs* CONTROL_*3rdTR*_female, male	ENSG00000155511	*GRIA1*	-0.8422	0.0473
post-LVC ectasia *vs* CONTROL_*3rdTR*_female, male	ENSG00000187730	*GABRD*	-0.8409	0.0350
post-LVC ectasia *vs* CONTROL_*3rdTR*_female, male	ENSG00000165521	*EML5*	-0.8405	0.0375
post-LVC ectasia *vs* CONTROL_*3rdTR*_female, male	ENSG00000185046	*ANKS1B*	-0.8390	0.0342
post-LVC ectasia *vs* CONTROL_*3rdTR*_female, male	ENSG00000130413	*STK33*	-0.8382	0.0457
post-LVC ectasia *vs* CONTROL_*3rdTR*_female, male	ENSG00000198467	*TPM2*	-0.8259	0.0430
post-LVC ectasia *vs* CONTROL_*3rdTR*_female, male	ENSG00000154928	*EPHB1*	-0.8227	0.0384
post-LVC ectasia *vs* CONTROL_*3rdTR*_female, male	ENSG00000121207	*LRAT*	-0.8199	0.0457

post-LVC ectasia *vs* CONTROL_*3rdTR*_female, male	ENSG00000142408	*CACNG8*	-0.8183	0.0457
post-LVC ectasia *vs* CONTROL_*3rdTR*_female, male	ENSG00000186648	*CARMIL3*	-0.8102	0.0341
post-LVC ectasia *vs* CONTROL_*3rdTR*_female, male	ENSG00000166105	*GLB1L3*	-0.8086	0.0417
post-LVC ectasia *vs* CONTROL_*3rdTR*_female, male	ENSG00000058091	*CDK14*	-0.8072	0.0205
post-LVC ectasia *vs* CONTROL_*3rdTR*_female, male	ENSG00000103175	*WFDC1*	-0.8062	0.0463
post-LVC ectasia *vs* CONTROL_*3rdTR*_female, male	ENSG00000137261	*KIAA0319*	-0.8023	0.0331
post-LVC ectasia *vs* CONTROL_*3rdTR*_female, male	ENSG00000075884	*ARHGAP15*	-0.7712	0.0493
post-LVC ectasia *vs* CONTROL_*3rdTR*_female, male	ENSG00000197816	*CCDC180*	-0.7695	0.0428
post-LVC ectasia *vs* CONTROL_*3rdTR*_female, male	ENSG00000162739	*SLAMF6*	-0.7664	0.0491
post-LVC ectasia *vs* CONTROL_*3rdTR*_female, male	ENSG00000158560	*DYNC1I1*	-0.7598	0.0471
post-LVC ectasia *vs* CONTROL_*3rdTR*_female, male	ENSG00000141837	*CACNA1A*	-0.7564	0.0428
post-LVC ectasia *vs* CONTROL_*3rdTR*_female, male	ENSG00000179583	*CIITA*	-0.7448	0.0286
post-LVC ectasia *vs* CONTROL_*3rdTR*_female, male	ENSG00000137573	*SULF1*	-0.7373	0.0350
post-LVC ectasia *vs* CONTROL_*3rdTR*_female, male	ENSG00000121440	*PDZRN3*	-0.7355	0.0205
post-LVC ectasia *vs* CONTROL_*3rdTR*_female, male	ENSG00000198932	*GPRASP1*	-0.7319	0.0419
post-LVC ectasia *vs* CONTROL_*3rdTR*_female, male	ENSG00000132535	*DLG4*	-0.7253	0.0342
post-LVC ectasia *vs* CONTROL_*3rdTR*_female, male	ENSG00000183196	*CHST6*	-0.7212	0.0334
post-LVC ectasia *vs* CONTROL_*3rdTR*_female, male	ENSG00000205560	*CPT1B*	-0.7200	0.0286
post-LVC ectasia *vs* CONTROL_*3rdTR*_female, male	ENSG00000171385	*KCND3*	-0.7184	0.0267
post-LVC ectasia *vs* CONTROL_*3rdTR*_female, male	ENSG00000105479	*CCDC114*	-0.7134	0.0457
post-LVC ectasia *vs* CONTROL_*3rdTR*_female, male	ENSG00000204681	*GABBR1*	-0.6970	0.0409
post-LVC ectasia *vs* CONTROL_*3rdTR*_female, male	ENSG00000198185	*ZNF334*	-0.6902	0.0434
post-LVC ectasia *vs* CONTROL_*3rdTR*_female, male	ENSG00000005187	*ACSM3*	-0.6755	0.0401
post-LVC ectasia *vs* CONTROL_*3rdTR*_female, male	ENSG00000100077	*GRK3*	-0.6533	0.0342
post-LVC ectasia *vs* CONTROL_*3rdTR*_female, male	ENSG00000153291	*SLC25A27*	-0.6437	0.0366
post-LVC ectasia *vs* CONTROL_*3rdTR*_female, male	ENSG00000166147	*FBN1*	-0.6402	0.0491
post-LVC ectasia *vs* CONTROL_*3rdTR*_female, male	ENSG00000204311	*PJVK*	-0.6281	0.0422
post-LVC ectasia *vs* CONTROL_*3rdTR*_female, male	ENSG00000163638	*ADAMTS9*	-0.6242	0.0488
post-LVC ectasia *vs* CONTROL_*3rdTR*_female, male	ENSG00000198198	*SZT2*	-0.6089	0.0444
post-LVC ectasia *vs* CONTROL_*3rdTR*_female, male	ENSG00000206530	*CFAP44*	-0.6055	0.0412
post-LVC ectasia *vs* CONTROL_*3rdTR*_female, male	ENSG00000243156	*MICAL3*	-0.6037	0.0357
post-LVC ectasia *vs* CONTROL_*3rdTR*_female, male	ENSG00000157388	*CACNA1D*	-0.5853	0.0453
post-LVC ectasia *vs* CONTROL_*3rdTR*_female, male	ENSG00000245848	*CEBPA*	0.5860	0.0454
post-LVC ectasia *vs* CONTROL_*3rdTR*_female, male	ENSG00000181610	*MRPS23*	0.5864	0.0478
post-LVC ectasia *vs* CONTROL_*3rdTR*_female, male	ENSG00000142546	*NOSIP*	0.5865	0.0498
post-LVC ectasia *vs* CONTROL_*3rdTR*_female, male	ENSG00000116711	*PLA2G4A*	0.5866	0.0286
post-LVC ectasia *vs* CONTROL_*3rdTR*_female, male	ENSG00000099385	*BCL7C*	0.5866	0.0469
post-LVC ectasia *vs* CONTROL_*3rdTR*_female, male	ENSG00000167578	*RAB4B*	0.5875	0.0491
post-LVC ectasia *vs* CONTROL_*3rdTR*_female, male	ENSG00000130193	*THEM6*	0.5877	0.0457
post-LVC ectasia *vs* CONTROL_*3rdTR*_female, male	ENSG00000114779	*ABHD14B*	0.5886	0.0364
post-LVC ectasia *vs* CONTROL_*3rdTR*_female, male	ENSG00000184897	*H1-10*	0.5896	0.0401
post-LVC ectasia *vs* CONTROL_*3rdTR*_female, male	ENSG00000151651	*ADAM8*	0.5904	0.0412
post-LVC ectasia *vs* CONTROL_*3rdTR*_female, male	ENSG00000177096	*PHETA2*	0.5912	0.0243
post-LVC ectasia *vs* CONTROL_*3rdTR*_female, male	ENSG00000171310	*CHST11*	0.5913	0.0453
post-LVC ectasia *vs* CONTROL_*3rdTR*_female, male	ENSG00000177156	*TALDO1*	0.5919	0.0334
post-LVC ectasia *vs* CONTROL_*3rdTR*_female, male	ENSG00000168275	*COA6*	0.5925	0.0481
post-LVC ectasia *vs* CONTROL_*3rdTR*_female, male	ENSG00000105364	*MRPL4*	0.5932	0.0386
post-LVC ectasia *vs* CONTROL_*3rdTR*_female, male	ENSG00000101843	*PSMD10*	0.5936	0.0428
post-LVC ectasia *vs* CONTROL_*3rdTR*_female, male	ENSG00000101182	*PSMA7*	0.5943	0.0492
post-LVC ectasia *vs* CONTROL_*3rdTR*_female, male	ENSG00000184840	*TMED9*	0.5954	0.0419
post-LVC ectasia *vs* CONTROL_*3rdTR*_female, male	ENSG00000111669	*TPI1*	0.5958	0.0478
post-LVC ectasia *vs* CONTROL_*3rdTR*_female, male	ENSG00000129255	*MPDU1*	0.5962	0.0419
post-LVC ectasia *vs* CONTROL_*3rdTR*_female, male	ENSG00000114021	*NIT2*	0.5971	0.0286
post-LVC ectasia *vs* CONTROL_*3rdTR*_female, male	ENSG00000169964	*TMEM42*	0.5973	0.0412
post-LVC ectasia *vs* CONTROL_*3rdTR*_female, male	ENSG00000108479	*GALK1*	0.5987	0.0479
post-LVC ectasia *vs* CONTROL_*3rdTR*_female, male	ENSG00000126254	*RBM42*	0.6002	0.0422
post-LVC ectasia *vs* CONTROL_*3rdTR*_female, male	ENSG00000246705	*H2AJ*	0.6010	0.0482
post-LVC ectasia *vs* CONTROL_*3rdTR*_female, male	ENSG00000105472	*CLEC11A*	0.6012	0.0488
post-LVC ectasia *vs* CONTROL_*3rdTR*_female, male	ENSG00000171004	*HS6ST2*	0.6015	0.0413

post-LVC ectasia *vs* CONTROL_*3rdTR*_female, male	ENSG00000176485	*PLAAT3*	0.6021	0.0400
post-LVC ectasia *vs* CONTROL_*3rdTR*_female, male	ENSG00000187049	*TMEM216*	0.6023	0.0350
post-LVC ectasia *vs* CONTROL_*3rdTR*_female, male	ENSG00000178078	*STAP2*	0.6024	0.0284
post-LVC ectasia *vs* CONTROL_*3rdTR*_female, male	ENSG00000173530	*TNFRSF10D*	0.6030	0.0372
post-LVC ectasia *vs* CONTROL_*3rdTR*_female, male	ENSG00000114383	*TUSC2*	0.6037	0.0401
post-LVC ectasia *vs* CONTROL_*3rdTR*_female, male	ENSG00000244038	*DDOST*	0.6049	0.0375
post-LVC ectasia *vs* CONTROL_*3rdTR*_female, male	ENSG00000175573	*C11orf68*	0.6051	0.0478
post-LVC ectasia *vs* CONTROL_*3rdTR*_female, male	ENSG00000130055	*GDPD2*	0.6058	0.0457
post-LVC ectasia *vs* CONTROL_*3rdTR*_female, male	ENSG00000165410	*CFL2*	0.6062	0.0488
post-LVC ectasia *vs* CONTROL_*3rdTR*_female, male	ENSG00000105518	*TMEM205*	0.6084	0.0419
post-LVC ectasia *vs* CONTROL_*3rdTR*_female, male	ENSG00000104883	*PEX11G*	0.6085	0.0344
post-LVC ectasia *vs* CONTROL_*3rdTR*_female, male	ENSG00000105355	*PLIN3*	0.6087	0.0419
post-LVC ectasia *vs* CONTROL_*3rdTR*_female, male	ENSG00000215915	*ATAD3C*	0.6091	0.0326
post-LVC ectasia *vs* CONTROL_*3rdTR*_female, male	ENSG00000179933	*C14orf119*	0.6103	0.0422
post-LVC ectasia *vs* CONTROL_*3rdTR*_female, male	ENSG00000117592	*PRDX6*	0.6106	0.0397
post-LVC ectasia *vs* CONTROL_*3rdTR*_female, male	ENSG00000183172	*SMDT1*	0.6120	0.0428
post-LVC ectasia *vs* CONTROL_*3rdTR*_female, male	ENSG00000048162	*NOP16*	0.6124	0.0286
post-LVC ectasia *vs* CONTROL_*3rdTR*_female, male	ENSG00000184584	*STING1*	0.6127	0.0471
post-LVC ectasia *vs* CONTROL_*3rdTR*_female, male	ENSG00000154342	*WNT3A*	0.6128	0.0488
post-LVC ectasia *vs* CONTROL_*3rdTR*_female, male	ENSG00000106367	*AP1S1*	0.6130	0.0364
post-LVC ectasia *vs* CONTROL_*3rdTR*_female, male	ENSG00000116691	*MIIP*	0.6135	0.0364
post-LVC ectasia *vs* CONTROL_*3rdTR*_female, male	ENSG00000102178	*UBL4A*	0.6139	0.0404
post-LVC ectasia *vs* CONTROL_*3rdTR*_female, male	ENSG00000224051	*CPTP*	0.6143	0.0386
post-LVC ectasia *vs* CONTROL_*3rdTR*_female, male	ENSG00000005022	*SLC25A5*	0.6153	0.0400
post-LVC ectasia *vs* CONTROL_*3rdTR*_female, male	ENSG00000109089	*CDR2L*	0.6161	0.0457
post-LVC ectasia *vs* CONTROL_*3rdTR*_female, male	ENSG00000125734	*GPR108*	0.6162	0.0420
post-LVC ectasia *vs* CONTROL_*3rdTR*_female, male	ENSG00000166394	*CYB5R2*	0.6165	0.0406
post-LVC ectasia *vs* CONTROL_*3rdTR*_female, male	ENSG00000203722	*RAET1G*	0.6179	0.0457
post-LVC ectasia *vs* CONTROL_*3rdTR*_female, male	ENSG00000198951	*NAGA*	0.6182	0.0286
post-LVC ectasia *vs* CONTROL_*3rdTR*_female, male	ENSG00000066379	*ZNRD1*	0.6183	0.0469
post-LVC ectasia *vs* CONTROL_*3rdTR*_female, male	ENSG00000234465	*PINLYP*	0.6186	0.0478
post-LVC ectasia *vs* CONTROL_*3rdTR*_female, male	ENSG00000161179	*YDJC*	0.6187	0.0356
post-LVC ectasia *vs* CONTROL_*3rdTR*_female, male	ENSG00000184162	*NR2C2AP*	0.6192	0.0419
post-LVC ectasia *vs* CONTROL_*3rdTR*_female, male	ENSG00000169026	*SLC49A3*	0.6201	0.0457
post-LVC ectasia *vs* CONTROL_*3rdTR*_female, male	ENSG00000157593	*SLC35B2*	0.6202	0.0398
post-LVC ectasia *vs* CONTROL_*3rdTR*_female, male	ENSG00000185043	*CIB1*	0.6213	0.0347
post-LVC ectasia *vs* CONTROL_*3rdTR*_female, male	ENSG00000160813	*PPP1R35*	0.6215	0.0421
post-LVC ectasia *vs* CONTROL_*3rdTR*_female, male	ENSG00000130545	*CRB3*	0.6219	0.0293
post-LVC ectasia *vs* CONTROL_*3rdTR*_female, male	ENSG00000112494	*UNC93A*	0.6223	0.0334
post-LVC ectasia *vs* CONTROL_*3rdTR*_female, male	ENSG00000030110	*BAK1*	0.6236	0.0412
post-LVC ectasia *vs* CONTROL_*3rdTR*_female, male	ENSG00000179091	*CYC1*	0.6254	0.0468
post-LVC ectasia *vs* CONTROL_*3rdTR*_female, male	ENSG00000171135	*JAGN1*	0.6255	0.0415
post-LVC ectasia *vs* CONTROL_*3rdTR*_female, male	ENSG00000197837	*H4-16*	0.6257	0.0422
post-LVC ectasia *vs* CONTROL_*3rdTR*_female, male	ENSG00000105701	*FKBP8*	0.6263	0.0457
post-LVC ectasia *vs* CONTROL_*3rdTR*_female, male	ENSG00000181026	*AEN*	0.6266	0.0438
post-LVC ectasia *vs* CONTROL_*3rdTR*_female, male	ENSG00000182544	*MFSD5*	0.6271	0.0490
post-LVC ectasia *vs* CONTROL_*3rdTR*_female, male	ENSG00000022567	*SLC45A4*	0.6286	0.0053
post-LVC ectasia *vs* CONTROL_*3rdTR*_female, male	ENSG00000197417	*SHPK*	0.6291	0.0007
post-LVC ectasia *vs* CONTROL_*3rdTR*_female, male	ENSG00000119333	*WDR34*	0.6300	0.0349
post-LVC ectasia *vs* CONTROL_*3rdTR*_female, male	ENSG00000169692	*AGPAT2*	0.6303	0.0419
post-LVC ectasia *vs* CONTROL_*3rdTR*_female, male	ENSG00000187051	*RPS19BP1*	0.6318	0.0428
post-LVC ectasia *vs* CONTROL_*3rdTR*_female, male	ENSG00000277972	*CISD3*	0.6320	0.0417
post-LVC ectasia *vs* CONTROL_*3rdTR*_female, male	ENSG00000103811	*CTSH*	0.6324	0.0468
post-LVC ectasia *vs* CONTROL_*3rdTR*_female, male	ENSG00000134955	*SLC37A2*	0.6325	0.0457
post-LVC ectasia *vs* CONTROL_*3rdTR*_female, male	ENSG00000132004	*FBXW9*	0.6336	0.0350
post-LVC ectasia *vs* CONTROL_*3rdTR*_female, male	ENSG00000221821	*C6orf226*	0.6338	0.0491
post-LVC ectasia *vs* CONTROL_*3rdTR*_female, male	ENSG00000128272	*ATF4*	0.6344	0.0494
post-LVC ectasia *vs* CONTROL_*3rdTR*_female, male	ENSG00000104341	*LAPTM4B*	0.6347	0.0434
post-LVC ectasia *vs* CONTROL_*3rdTR*_female, male	ENSG00000123892	*RAB38*	0.6350	0.0342

post-LVC ectasia *vs* CONTROL_*3rdTR*_female, male	ENSG00000130522	*JUND*	0.6350	0.0386
post-LVC ectasia *vs* CONTROL_*3rdTR*_female, male	ENSG00000170619	*COMMD5*	0.6355	0.0351
post-LVC ectasia *vs* CONTROL_*3rdTR*_female, male	ENSG00000159377	*PSMB4*	0.6357	0.0490
post-LVC ectasia *vs* CONTROL_*3rdTR*_female, male	ENSG00000109861	*CTSC*	0.6359	0.0350
post-LVC ectasia *vs* CONTROL_*3rdTR*_female, male	ENSG00000131015	*ULBP2*	0.6373	0.0428
post-LVC ectasia *vs* CONTROL_*3rdTR*_female, male	ENSG00000174917	*MICOS13*	0.6374	0.0468
post-LVC ectasia *vs* CONTROL_*3rdTR*_female, male	ENSG00000174903	*RAB1B*	0.6378	0.0307
post-LVC ectasia *vs* CONTROL_*3rdTR*_female, male	ENSG00000143612	*C1orf43*	0.6380	0.0350
post-LVC ectasia *vs* CONTROL_*3rdTR*_female, male	ENSG00000257949	*TEN1*	0.6380	0.0457
post-LVC ectasia *vs* CONTROL_*3rdTR*_female, male	ENSG00000204237	*OXLD1*	0.6384	0.0362
post-LVC ectasia *vs* CONTROL_*3rdTR*_female, male	ENSG00000127445	*PIN1*	0.6388	0.0459
post-LVC ectasia *vs* CONTROL_*3rdTR*_female, male	ENSG00000117448	*AKR1A1*	0.6395	0.0320
post-LVC ectasia *vs* CONTROL_*3rdTR*_female, male	ENSG00000131408	*NR1H2*	0.6400	0.0364
post-LVC ectasia *vs* CONTROL_*3rdTR*_female, male	ENSG00000167565	*SERTAD3*	0.6400	0.0401
post-LVC ectasia *vs* CONTROL_*3rdTR*_female, male	ENSG00000073849	*ST6GAL1*	0.6406	0.0351
post-LVC ectasia *vs* CONTROL_*3rdTR*_female, male	ENSG00000103502	*CDIPT*	0.6419	0.0364
post-LVC ectasia *vs* CONTROL_*3rdTR*_female, male	ENSG00000204272	*NBDY*	0.6422	0.0421
post-LVC ectasia *vs* CONTROL_*3rdTR*_female, male	ENSG00000006534	*ALDH3B1*	0.6428	0.0323
post-LVC ectasia *vs* CONTROL_*3rdTR*_female, male	ENSG00000118640	*VAMP8*	0.6434	0.0492
post-LVC ectasia *vs* CONTROL_*3rdTR*_female, male	ENSG00000167601	*AXL*	0.6436	0.0386
post-LVC ectasia *vs* CONTROL_*3rdTR*_female, male	ENSG00000178363	*CALML3*	0.6438	0.0493
post-LVC ectasia *vs* CONTROL_*3rdTR*_female, male	ENSG00000221955	*SLC12A8*	0.6443	0.0419
post-LVC ectasia *vs* CONTROL_*3rdTR*_female, male	ENSG00000117691	*NENF*	0.6447	0.0482
post-LVC ectasia *vs* CONTROL_*3rdTR*_female, male	ENSG00000008324	*SS18L2*	0.6456	0.0464
post-LVC ectasia *vs* CONTROL_*3rdTR*_female, male	ENSG00000154102	*C16orf74*	0.6466	0.0344
post-LVC ectasia *vs* CONTROL_*3rdTR*_female, male	ENSG00000217555	*CKLF*	0.6469	0.0457
post-LVC ectasia *vs* CONTROL_*3rdTR*_female, male	ENSG00000114631	*PODXL2*	0.6471	0.0350
post-LVC ectasia *vs* CONTROL_*3rdTR*_female, male	ENSG00000179085	*DPM3*	0.6479	0.0492
post-LVC ectasia *vs* CONTROL_*3rdTR*_female, male	ENSG00000132207	*SLX1A*	0.6484	0.0419
post-LVC ectasia *vs* CONTROL_*3rdTR*_female, male	ENSG00000052344	*PRSS8*	0.6493	0.0276
post-LVC ectasia *vs* CONTROL_*3rdTR*_female, male	ENSG00000164967	*RPP25L*	0.6496	0.0457
post-LVC ectasia *vs* CONTROL_*3rdTR*_female, male	ENSG00000124767	*GLO1*	0.6505	0.0400
post-LVC ectasia *vs* CONTROL_*3rdTR*_female, male	ENSG00000182154	*MRPL41*	0.6508	0.0455
post-LVC ectasia *vs* CONTROL_*3rdTR*_female, male	ENSG00000148290	*SURF1*	0.6515	0.0454
post-LVC ectasia *vs* CONTROL_*3rdTR*_female, male	ENSG00000189334	*S100A14*	0.6517	0.0286
post-LVC ectasia *vs* CONTROL_*3rdTR*_female, male	ENSG00000169972	*PUSL1*	0.6535	0.0379
post-LVC ectasia *vs* CONTROL_*3rdTR*_female, male	ENSG00000137404	*NRM*	0.6542	0.0478
post-LVC ectasia *vs* CONTROL_*3rdTR*_female, male	ENSG00000123143	*PKN1*	0.6544	0.0457
post-LVC ectasia *vs* CONTROL_*3rdTR*_female, male	ENSG00000131475	*VPS25*	0.6544	0.0419
post-LVC ectasia *vs* CONTROL_*3rdTR*_female, male	ENSG00000105281	*SLC1A5*	0.6550	0.0284
post-LVC ectasia *vs* CONTROL_*3rdTR*_female, male	ENSG00000153048	*CARHSP1*	0.6553	0.0430
post-LVC ectasia *vs* CONTROL_*3rdTR*_female, male	ENSG00000172382	*PRSS27*	0.6556	0.0442
post-LVC ectasia *vs* CONTROL_*3rdTR*_female, male	ENSG00000205795	*CYS1*	0.6574	0.0481
post-LVC ectasia *vs* CONTROL_*3rdTR*_female, male	ENSG00000115286	*NDUFS7*	0.6575	0.0353
post-LVC ectasia *vs* CONTROL_*3rdTR*_female, male	ENSG00000163479	*SSR2*	0.6576	0.0421
post-LVC ectasia *vs* CONTROL_*3rdTR*_female, male	ENSG00000173207	*CKS1B*	0.6587	0.0479
post-LVC ectasia *vs* CONTROL_*3rdTR*_female, male	ENSG00000177868	*SVBP*	0.6602	0.0457
post-LVC ectasia *vs* CONTROL_*3rdTR*_female, male	ENSG00000168374	*ARF4*	0.6608	0.0286
post-LVC ectasia *vs* CONTROL_*3rdTR*_female, male	ENSG00000217930	*PAM16*	0.6615	0.0302
post-LVC ectasia *vs* CONTROL_*3rdTR*_female, male	ENSG00000172586	*CHCHD1*	0.6616	0.0426
post-LVC ectasia *vs* CONTROL_*3rdTR*_female, male	ENSG00000105755	*ETHE1*	0.6620	0.0481
post-LVC ectasia *vs* CONTROL_*3rdTR*_female, male	ENSG00000196072	*BLOC1S2*	0.6622	0.0457
post-LVC ectasia *vs* CONTROL_*3rdTR*_female, male	ENSG00000188643	*S100A16*	0.6644	0.0407
post-LVC ectasia *vs* CONTROL_*3rdTR*_female, male	ENSG00000161653	*NAGS*	0.6645	0.0394
post-LVC ectasia *vs* CONTROL_*3rdTR*_female, male	ENSG00000162543	*UBXN10*	0.6675	0.0205
post-LVC ectasia *vs* CONTROL_*3rdTR*_female, male	ENSG00000125037	*EMC3*	0.6684	0.0422
post-LVC ectasia *vs* CONTROL_*3rdTR*_female, male	ENSG00000100387	*RBX1*	0.6684	0.0323
post-LVC ectasia *vs* CONTROL_*3rdTR*_female, male	ENSG00000256812	*CAPNS2*	0.6693	0.0350
post-LVC ectasia *vs* CONTROL_*3rdTR*_female, male	ENSG00000171314	*PGAM1*	0.6700	0.0485

post-LVC ectasia *vs* CONTROL_*3rdTR*_female, male	ENSG00000166289	*PLEKHF1*	0.6705	0.0350
post-LVC ectasia *vs* CONTROL_*3rdTR*_female, male	ENSG00000175756	*AURKAIP1*	0.6714	0.0430
post-LVC ectasia *vs* CONTROL_*3rdTR*_female, male	ENSG00000169750	*RAC3*	0.6720	0.0412
post-LVC ectasia *vs* CONTROL_*3rdTR*_female, male	ENSG00000137547	*MRPL15*	0.6725	0.0360
post-LVC ectasia *vs* CONTROL_*3rdTR*_female, male	ENSG00000095906	*NUBP2*	0.6737	0.0417
post-LVC ectasia *vs* CONTROL_*3rdTR*_female, male	ENSG00000180992	*MRPL14*	0.6737	0.0386
post-LVC ectasia *vs* CONTROL_*3rdTR*_female, male	ENSG00000107438	*PDLIM1*	0.6759	0.0380
post-LVC ectasia *vs* CONTROL_*3rdTR*_female, male	ENSG00000189143	*CLDN4*	0.6767	0.0364
post-LVC ectasia *vs* CONTROL_*3rdTR*_female, male	ENSG00000112667	*DNPH1*	0.6795	0.0364
post-LVC ectasia *vs* CONTROL_*3rdTR*_female, male	ENSG00000103024	*NME3*	0.6832	0.0440
post-LVC ectasia *vs* CONTROL_*3rdTR*_female, male	ENSG00000177854	*TMEM187*	0.6835	0.0371
post-LVC ectasia *vs* CONTROL_*3rdTR*_female, male	ENSG00000137133	*HINT2*	0.6841	0.0457
post-LVC ectasia *vs* CONTROL_*3rdTR*_female, male	ENSG00000141741	*MIEN1*	0.6856	0.0492
post-LVC ectasia *vs* CONTROL_*3rdTR*_female, male	ENSG00000167165	*UGT1A6*	0.6859	0.0346
post-LVC ectasia *vs* CONTROL_*3rdTR*_female, male	ENSG00000106305	*AIMP2*	0.6862	0.0413
post-LVC ectasia *vs* CONTROL_*3rdTR*_female, male	ENSG00000166126	*AMN*	0.6866	0.0205
post-LVC ectasia *vs* CONTROL_*3rdTR*_female, male	ENSG00000171443	*ZNF524*	0.6871	0.0334
post-LVC ectasia *vs* CONTROL_*3rdTR*_female, male	ENSG00000181817	*LSM10*	0.6888	0.0398
post-LVC ectasia *vs* CONTROL_*3rdTR*_female, male	ENSG00000103152	*MPG*	0.6914	0.0353
post-LVC ectasia *vs* CONTROL_*3rdTR*_female, male	ENSG00000168894	*RNF181*	0.6929	0.0469
post-LVC ectasia *vs* CONTROL_*3rdTR*_female, male	ENSG00000166183	*ASPG*	0.6942	0.0334
post-LVC ectasia *vs* CONTROL_*3rdTR*_female, male	ENSG00000213977	*TAX1BP3*	0.6945	0.0368
post-LVC ectasia *vs* CONTROL_*3rdTR*_female, male	ENSG00000167118	*URM1*	0.6945	0.0366
post-LVC ectasia *vs* CONTROL_*3rdTR*_female, male	ENSG00000198680	*TUSC1*	0.6948	0.0397
post-LVC ectasia *vs* CONTROL_*3rdTR*_female, male	ENSG00000150394	*CDH8*	0.6956	0.0461
post-LVC ectasia *vs* CONTROL_*3rdTR*_female, male	ENSG00000134056	*MRPS36*	0.6962	0.0324
post-LVC ectasia *vs* CONTROL_*3rdTR*_female, male	ENSG00000011009	*LYPLA2*	0.6980	0.0303
post-LVC ectasia *vs* CONTROL_*3rdTR*_female, male	ENSG00000137818	*RPLP1*	0.6989	0.0428
post-LVC ectasia *vs* CONTROL_*3rdTR*_female, male	ENSG00000115255	*REEP6*	0.6992	0.0342
post-LVC ectasia *vs* CONTROL_*3rdTR*_female, male	ENSG00000116209	*TMEM59*	0.6993	0.0408
post-LVC ectasia *vs* CONTROL_*3rdTR*_female, male	ENSG00000205542	*TMSB4X*	0.7001	0.0478
post-LVC ectasia *vs* CONTROL_*3rdTR*_female, male	ENSG00000077348	*EXOSC5*	0.7002	0.0432
post-LVC ectasia *vs* CONTROL_*3rdTR*_female, male	ENSG00000198931	*APRT*	0.7007	0.0420
post-LVC ectasia *vs* CONTROL_*3rdTR*_female, male	ENSG00000177556	*ATOX1*	0.7011	0.0404
post-LVC ectasia *vs* CONTROL_*3rdTR*_female, male	ENSG00000102934	*PLLP*	0.7013	0.0399
post-LVC ectasia *vs* CONTROL_*3rdTR*_female, male	ENSG00000172927	*MYEOV*	0.7022	0.0488
post-LVC ectasia *vs* CONTROL_*3rdTR*_female, male	ENSG00000168701	*TMEM208*	0.7030	0.0307
post-LVC ectasia *vs* CONTROL_*3rdTR*_female, male	ENSG00000171425	*ZNF581*	0.7034	0.0350
post-LVC ectasia *vs* CONTROL_*3rdTR*_female, male	ENSG00000197019	*SERTAD1*	0.7034	0.0404
post-LVC ectasia *vs* CONTROL_*3rdTR*_female, male	ENSG00000244187	*TMEM141*	0.7047	0.0364
post-LVC ectasia *vs* CONTROL_*3rdTR*_female, male	ENSG00000224877	*NDUFAF8*	0.7080	0.0350
post-LVC ectasia *vs* CONTROL_*3rdTR*_female, male	ENSG00000065911	*MTHFD2*	0.7086	0.0441
post-LVC ectasia *vs* CONTROL_*3rdTR*_female, male	ENSG00000165672	*PRDX3*	0.7096	0.0483
post-LVC ectasia *vs* CONTROL_*3rdTR*_female, male	ENSG00000135390	*ATP5MC2*	0.7096	0.0420
post-LVC ectasia *vs* CONTROL_*3rdTR*_female, male	ENSG00000130332	*LSM7*	0.7106	0.0323
post-LVC ectasia *vs* CONTROL_*3rdTR*_female, male	ENSG00000166595	*CIAO2B*	0.7109	0.0473
post-LVC ectasia *vs* CONTROL_*3rdTR*_female, male	ENSG00000196502	*SULT1A1*	0.7125	0.0364
post-LVC ectasia *vs* CONTROL_*3rdTR*_female, male	ENSG00000117472	*TSPAN1*	0.7134	0.0364
post-LVC ectasia *vs* CONTROL_*3rdTR*_female, male	ENSG00000141933	*TPGS1*	0.7134	0.0323
post-LVC ectasia *vs* CONTROL_*3rdTR*_female, male	ENSG00000174276	*ZNHIT2*	0.7134	0.0459
post-LVC ectasia *vs* CONTROL_*3rdTR*_female, male	ENSG00000284194	*SCO2*	0.7139	0.0364
post-LVC ectasia *vs* CONTROL_*3rdTR*_female, male	ENSG00000101400	*SNTA1*	0.7148	0.0216
post-LVC ectasia *vs* CONTROL_*3rdTR*_female, male	ENSG00000117318	*ID3*	0.7156	0.0472
post-LVC ectasia *vs* CONTROL_*3rdTR*_female, male	ENSG00000167397	*VKORC1*	0.7176	0.0492
post-LVC ectasia *vs* CONTROL_*3rdTR*_female, male	ENSG00000101460	*MAP1LC3A*	0.7205	0.0318
post-LVC ectasia *vs* CONTROL_*3rdTR*_female, male	ENSG00000099795	*NDUFB7*	0.7212	0.0443
post-LVC ectasia *vs* CONTROL_*3rdTR*_female, male	ENSG00000119986	*AVPI1*	0.7264	0.0428
post-LVC ectasia *vs* CONTROL_*3rdTR*_female, male	ENSG00000147100	*SLC16A2*	0.7275	0.0420
post-LVC ectasia *vs* CONTROL_*3rdTR*_female, male	ENSG00000155380	*SLC16A1*	0.7275	0.0464

post-LVC ectasia *vs* CONTROL_*3rdTR*_female, male	ENSG00000183628	*DGCR6*	0.7286	0.0342
post-LVC ectasia *vs* CONTROL_*3rdTR*_female, male	ENSG00000116670	*MAD2L2*	0.7288	0.0364
post-LVC ectasia *vs* CONTROL_*3rdTR*_female, male	ENSG00000126768	*TIMM17B*	0.7288	0.0379
post-LVC ectasia *vs* CONTROL_*3rdTR*_female, male	ENSG00000110852	*CLEC2B*	0.7289	0.0334
post-LVC ectasia *vs* CONTROL_*3rdTR*_female, male	ENSG00000116663	*FBXO6*	0.7289	0.0174
post-LVC ectasia *vs* CONTROL_*3rdTR*_female, male	ENSG00000105258	*POLR2I*	0.7291	0.0473
post-LVC ectasia *vs* CONTROL_*3rdTR*_female, male	ENSG00000125968	*ID1*	0.7306	0.0426
post-LVC ectasia *vs* CONTROL_*3rdTR*_female, male	ENSG00000149806	*FAU*	0.7310	0.0489
post-LVC ectasia *vs* CONTROL_*3rdTR*_female, male	ENSG00000117362	*APH1A*	0.7318	0.0307
post-LVC ectasia *vs* CONTROL_*3rdTR*_female, male	ENSG00000102243	*VGLL1*	0.7324	0.0400
post-LVC ectasia *vs* CONTROL_*3rdTR*_female, male	ENSG00000211450	*SELENOH*	0.7325	0.0364
post-LVC ectasia *vs* CONTROL_*3rdTR*_female, male	ENSG00000196218	*RYR1*	0.7332	0.0284
post-LVC ectasia *vs* CONTROL_*3rdTR*_female, male	ENSG00000213689	*TREX1*	0.7351	0.0354
post-LVC ectasia *vs* CONTROL_*3rdTR*_female, male	ENSG00000185306	*C12orf56*	0.7365	0.0485
post-LVC ectasia *vs* CONTROL_*3rdTR*_female, male	ENSG00000165233	*CARD19*	0.7367	0.0296
post-LVC ectasia *vs* CONTROL_*3rdTR*_female, male	ENSG00000186010	*NDUFA13*	0.7372	0.0457
post-LVC ectasia *vs* CONTROL_*3rdTR*_female, male	ENSG00000172183	*ISG20*	0.7410	0.0018
post-LVC ectasia *vs* CONTROL_*3rdTR*_female, male	ENSG00000146072	*TNFRSF21*	0.7418	0.0151
post-LVC ectasia *vs* CONTROL_*3rdTR*_female, male	ENSG00000204444	*APOM*	0.7427	0.0481
post-LVC ectasia *vs* CONTROL_*3rdTR*_female, male	ENSG00000075415	*SLC25A3*	0.7449	0.0333
post-LVC ectasia *vs* CONTROL_*3rdTR*_female, male	ENSG00000178449	*COX14*	0.7451	0.0403
post-LVC ectasia *vs* CONTROL_*3rdTR*_female, male	ENSG00000165264	*NDUFB6*	0.7459	0.0479
post-LVC ectasia *vs* CONTROL_*3rdTR*_female, male	ENSG00000167074	*TEF*	0.7462	0.0406
post-LVC ectasia *vs* CONTROL_*3rdTR*_female, male	ENSG00000243056	*EIF4EBP3*	0.7463	0.0319
post-LVC ectasia *vs* CONTROL_*3rdTR*_female, male	ENSG00000232388	*SMIM26*	0.7475	0.0371
post-LVC ectasia *vs* CONTROL_*3rdTR*_female, male	ENSG00000196976	*LAGE3*	0.7495	0.0488
post-LVC ectasia *vs* CONTROL_*3rdTR*_female, male	ENSG00000101187	*SLCO4A1*	0.7503	0.0397
post-LVC ectasia *vs* CONTROL_*3rdTR*_female, male	ENSG00000197353	*LYPD2*	0.7505	0.0493
post-LVC ectasia *vs* CONTROL_*3rdTR*_female, male	ENSG00000141526	*SLC16A3*	0.7508	0.0419
post-LVC ectasia *vs* CONTROL_*3rdTR*_female, male	ENSG00000177105	*RHOG*	0.7516	0.0386
post-LVC ectasia *vs* CONTROL_*3rdTR*_female, male	ENSG00000173171	*MTX1*	0.7523	0.0368
post-LVC ectasia *vs* CONTROL_*3rdTR*_female, male	ENSG00000205220	*PSMB10*	0.7533	0.0321
post-LVC ectasia *vs* CONTROL_*3rdTR*_female, male	ENSG00000170889	*RPS9*	0.7534	0.0441
post-LVC ectasia *vs* CONTROL_*3rdTR*_female, male	ENSG00000164251	*F2RL1*	0.7545	0.0386
post-LVC ectasia *vs* CONTROL_*3rdTR*_female, male	ENSG00000002330	*BAD*	0.7549	0.0364
post-LVC ectasia *vs* CONTROL_*3rdTR*_female, male	ENSG00000163710	*PCOLCE2*	0.7558	0.0415
post-LVC ectasia *vs* CONTROL_*3rdTR*_female, male	ENSG00000198755	*RPL10A*	0.7593	0.0461
post-LVC ectasia *vs* CONTROL_*3rdTR*_female, male	ENSG00000105677	*TMEM147*	0.7597	0.0430
post-LVC ectasia *vs* CONTROL_*3rdTR*_female, male	ENSG00000204323	*SMIM5*	0.7605	0.0216
post-LVC ectasia *vs* CONTROL_*3rdTR*_female, male	ENSG00000242372	*EIF6*	0.7622	0.0286
post-LVC ectasia *vs* CONTROL_*3rdTR*_female, male	ENSG00000104964	*TLE5*	0.7628	0.0334
post-LVC ectasia *vs* CONTROL_*3rdTR*_female, male	ENSG00000173267	*SNCG*	0.7631	0.0334
post-LVC ectasia *vs* CONTROL_*3rdTR*_female, male	ENSG00000108961	*RANGRF*	0.7651	0.0386
post-LVC ectasia *vs* CONTROL_*3rdTR*_female, male	ENSG00000176101	*SSNA1*	0.7677	0.0400
post-LVC ectasia *vs* CONTROL_*3rdTR*_female, male	ENSG00000105519	*CAPS*	0.7701	0.0342
post-LVC ectasia *vs* CONTROL_*3rdTR*_female, male	ENSG00000106399	*RPA3*	0.7710	0.0357
post-LVC ectasia *vs* CONTROL_*3rdTR*_female, male	ENSG00000117450	*PRDX1*	0.7712	0.0302
post-LVC ectasia *vs* CONTROL_*3rdTR*_female, male	ENSG00000129562	*DAD1*	0.7760	0.0412
post-LVC ectasia *vs* CONTROL_*3rdTR*_female, male	ENSG00000175602	*CCDC85B*	0.7765	0.0465
post-LVC ectasia *vs* CONTROL_*3rdTR*_female, male	ENSG00000137709	*POU2F3*	0.7767	0.0286
post-LVC ectasia *vs* CONTROL_*3rdTR*_female, male	ENSG00000229833	*PET100*	0.7777	0.0419
post-LVC ectasia *vs* CONTROL_*3rdTR*_female, male	ENSG00000179958	*DCTPP1*	0.7783	0.0464
post-LVC ectasia *vs* CONTROL_*3rdTR*_female, male	ENSG00000161677	*JOSD2*	0.7792	0.0485
post-LVC ectasia *vs* CONTROL_*3rdTR*_female, male	ENSG00000106153	*CHCHD2*	0.7808	0.0492
post-LVC ectasia *vs* CONTROL_*3rdTR*_female, male	ENSG00000110700	*RPS13*	0.7824	0.0426
post-LVC ectasia *vs* CONTROL_*3rdTR*_female, male	ENSG00000167515	*TRAPPC2L*	0.7873	0.0286
post-LVC ectasia *vs* CONTROL_*3rdTR*_female, male	ENSG00000228300	*FAM174C*	0.7884	0.0492
post-LVC ectasia *vs* CONTROL_*3rdTR*_female, male	ENSG00000198113	*TOR4A*	0.7892	0.0364
post-LVC ectasia *vs* CONTROL_*3rdTR*_female, male	ENSG00000072818	*ACAP1*	0.7893	0.0428

post-LVC ectasia *vs* CONTROL_*3rdTR*_female, male	ENSG00000146938	*NLGN4X*	0.7916	0.0403
post-LVC ectasia *vs* CONTROL_*3rdTR*_female, male	ENSG00000107738	*VSIR*	0.7923	0.0428
post-LVC ectasia *vs* CONTROL_*3rdTR*_female, male	ENSG00000100290	*BIK*	0.7926	0.0290
post-LVC ectasia *vs* CONTROL_*3rdTR*_female, male	ENSG00000170296	*GABARAP*	0.7936	0.0386
post-LVC ectasia *vs* CONTROL_*3rdTR*_female, male	ENSG00000181218	*H2AW*	0.7943	0.0174
post-LVC ectasia *vs* CONTROL_*3rdTR*_female, male	ENSG00000143162	*CREG1*	0.8004	0.0151
post-LVC ectasia *vs* CONTROL_*3rdTR*_female, male	ENSG00000125775	*SDCBP2*	0.8005	0.0183
post-LVC ectasia *vs* CONTROL_*3rdTR*_female, male	ENSG00000153551	*CMTM7*	0.8006	0.0428
post-LVC ectasia *vs* CONTROL_*3rdTR*_female, male	ENSG00000277791	*PSMB3*	0.8016	0.0412
post-LVC ectasia *vs* CONTROL_*3rdTR*_female, male	ENSG00000219200	*RNASEK*	0.8024	0.0293
post-LVC ectasia *vs* CONTROL_*3rdTR*_female, male	ENSG00000126267	*COX6B1*	0.8027	0.0419
post-LVC ectasia *vs* CONTROL_*3rdTR*_female, male	ENSG00000258315	*C17orf49*	0.8033	0.0403
post-LVC ectasia *vs* CONTROL_*3rdTR*_female, male	ENSG00000196154	*S100A4*	0.8034	0.0426
post-LVC ectasia *vs* CONTROL_*3rdTR*_female, male	ENSG00000136514	*RTP4*	0.8037	0.0254
post-LVC ectasia *vs* CONTROL_*3rdTR*_female, male	ENSG00000168273	*SMIM4*	0.8052	0.0174
post-LVC ectasia *vs* CONTROL_*3rdTR*_female, male	ENSG00000105583	*WDR83OS*	0.8069	0.0434
post-LVC ectasia *vs* CONTROL_*3rdTR*_female, male	ENSG00000205155	*PSENEN*	0.8073	0.0342
post-LVC ectasia *vs* CONTROL_*3rdTR*_female, male	ENSG00000127666	*TICAM1*	0.8096	0.0174
post-LVC ectasia *vs* CONTROL_*3rdTR*_female, male	ENSG00000142606	*MMEL1*	0.8096	0.0307
post-LVC ectasia *vs* CONTROL_*3rdTR*_female, male	ENSG00000137038	*DMAC1*	0.8106	0.0473
post-LVC ectasia *vs* CONTROL_*3rdTR*_female, male	ENSG00000187624	*C17orf97*	0.8111	0.0400
post-LVC ectasia *vs* CONTROL_*3rdTR*_female, male	ENSG00000183034	*OTOP2*	0.8112	0.0419
post-LVC ectasia *vs* CONTROL_*3rdTR*_female, male	ENSG00000125971	*DYNLRB1*	0.8114	0.0417
post-LVC ectasia *vs* CONTROL_*3rdTR*_female, male	ENSG00000169976	*SF3B5*	0.8125	0.0419
post-LVC ectasia *vs* CONTROL_*3rdTR*_female, male	ENSG00000127399	*LRRC61*	0.8140	0.0380
post-LVC ectasia *vs* CONTROL_*3rdTR*_female, male	ENSG00000131188	*PRR7*	0.8149	0.0406
post-LVC ectasia *vs* CONTROL_*3rdTR*_female, male	ENSG00000128626	*MRPS12*	0.8164	0.0342
post-LVC ectasia *vs* CONTROL_*3rdTR*_female, male	ENSG00000125835	*SNRPB*	0.8182	0.0400
post-LVC ectasia *vs* CONTROL_*3rdTR*_female, male	ENSG00000167644	*C19orf33*	0.8186	0.0428
post-LVC ectasia *vs* CONTROL_*3rdTR*_female, male	ENSG00000111678	*C12orf57*	0.8189	0.0478
post-LVC ectasia *vs* CONTROL_*3rdTR*_female, male	ENSG00000167315	*ACAA2*	0.8207	0.0341
post-LVC ectasia *vs* CONTROL_*3rdTR*_female, male	ENSG00000105669	*COPE*	0.8223	0.0386
post-LVC ectasia *vs* CONTROL_*3rdTR*_female, male	ENSG00000125611	*CHCHD5*	0.8256	0.0457
post-LVC ectasia *vs* CONTROL_*3rdTR*_female, male	ENSG00000157064	*NMNAT2*	0.8281	0.0286
post-LVC ectasia *vs* CONTROL_*3rdTR*_female, male	ENSG00000177697	*CD151*	0.8283	0.0296
post-LVC ectasia *vs* CONTROL_*3rdTR*_female, male	ENSG00000149557	*FEZ1*	0.8297	0.0386
post-LVC ectasia *vs* CONTROL_*3rdTR*_female, male	ENSG00000178980	*SELENOW*	0.8310	0.0419
post-LVC ectasia *vs* CONTROL_*3rdTR*_female, male	ENSG00000105327	*BBC3*	0.8322	0.0243
post-LVC ectasia *vs* CONTROL_*3rdTR*_female, male	ENSG00000105372	*RPS19*	0.8349	0.0492
post-LVC ectasia *vs* CONTROL_*3rdTR*_female, male	ENSG00000105011	*ASF1B*	0.8353	0.0443
post-LVC ectasia *vs* CONTROL_*3rdTR*_female, male	ENSG00000152082	*MZT2B*	0.8364	0.0364
post-LVC ectasia *vs* CONTROL_*3rdTR*_female, male	ENSG00000051523	*CYBA*	0.8367	0.0286
post-LVC ectasia *vs* CONTROL_*3rdTR*_female, male	ENSG00000179673	*RPRML*	0.8420	0.0364
post-LVC ectasia *vs* CONTROL_*3rdTR*_female, male	ENSG00000131495	*NDUFA2*	0.8429	0.0397
post-LVC ectasia *vs* CONTROL_*3rdTR*_female, male	ENSG00000063241	*ISOC2*	0.8438	0.0481
post-LVC ectasia *vs* CONTROL_*3rdTR*_female, male	ENSG00000214063	*TSPAN4*	0.8454	0.0342
post-LVC ectasia *vs* CONTROL_*3rdTR*_female, male	ENSG00000003137	*CYP26B1*	0.8461	0.0341
post-LVC ectasia *vs* CONTROL_*3rdTR*_female, male	ENSG00000085662	*AKR1B1*	0.8463	0.0350
post-LVC ectasia *vs* CONTROL_*3rdTR*_female, male	ENSG00000175592	*FOSL1*	0.8465	0.0481
post-LVC ectasia *vs* CONTROL_*3rdTR*_female, male	ENSG00000205544	*TMEM256*	0.8469	0.0372
post-LVC ectasia *vs* CONTROL_*3rdTR*_female, male	ENSG00000186603	*HPDL*	0.8471	0.0353
post-LVC ectasia *vs* CONTROL_*3rdTR*_female, male	ENSG00000134825	*TMEM258*	0.8490	0.0397
post-LVC ectasia *vs* CONTROL_*3rdTR*_female, male	ENSG00000140264	*SERF2*	0.8493	0.0401
post-LVC ectasia *vs* CONTROL_*3rdTR*_female, male	ENSG00000087086	*FTL*	0.8513	0.0463
post-LVC ectasia *vs* CONTROL_*3rdTR*_female, male	ENSG00000198258	*UBL5*	0.8523	0.0430
post-LVC ectasia *vs* CONTROL_*3rdTR*_female, male	ENSG00000128185	*DGCR6L*	0.8531	0.0419
post-LVC ectasia *vs* CONTROL_*3rdTR*_female, male	ENSG00000225663	*MCRIP1*	0.8536	0.0342
post-LVC ectasia *vs* CONTROL_*3rdTR*_female, male	ENSG00000197696	*NMB*	0.8553	0.0457
post-LVC ectasia *vs* CONTROL_*3rdTR*_female, male	ENSG00000148834	*GSTO1*	0.8558	0.0492

post-LVC ectasia *vs* CONTROL_*3rdTR*_female, male	ENSG00000146066	*HIGD2A*	0.8581	0.0356
post-LVC ectasia *vs* CONTROL_*3rdTR*_female, male	ENSG00000165215	*CLDN3*	0.8602	0.0445
post-LVC ectasia *vs* CONTROL_*3rdTR*_female, male	ENSG00000034510	*TMSB10*	0.8617	0.0494
post-LVC ectasia *vs* CONTROL_*3rdTR*_female, male	ENSG00000198937	*CCDC167*	0.8657	0.0428
post-LVC ectasia *vs* CONTROL_*3rdTR*_female, male	ENSG00000100129	*EIF3L*	0.8661	0.0371
post-LVC ectasia *vs* CONTROL_*3rdTR*_female, male	ENSG00000117410	*ATP6V0B*	0.8680	0.0366
post-LVC ectasia *vs* CONTROL_*3rdTR*_female, male	ENSG00000147403	*RPL10*	0.8688	0.0400
post-LVC ectasia *vs* CONTROL_*3rdTR*_female, male	ENSG00000162244	*RPL29*	0.8718	0.0419
post-LVC ectasia *vs* CONTROL_*3rdTR*_female, male	ENSG00000137880	*GCHFR*	0.8747	0.0307
post-LVC ectasia *vs* CONTROL_*3rdTR*_female, male	ENSG00000197903	*H2BC12*	0.8814	0.0488
post-LVC ectasia *vs* CONTROL_*3rdTR*_female, male	ENSG00000281991	*TMEM265*	0.8825	0.0457
post-LVC ectasia *vs* CONTROL_*3rdTR*_female, male	ENSG00000216490	*IFI30*	0.8846	0.0345
post-LVC ectasia *vs* CONTROL_*3rdTR*_female, male	ENSG00000005243	*COPZ2*	0.8877	0.0069
post-LVC ectasia *vs* CONTROL_*3rdTR*_female, male	ENSG00000196890	*H2BU1*	0.8882	0.0174
post-LVC ectasia *vs* CONTROL_*3rdTR*_female, male	ENSG00000267855	*NDUFA7*	0.8902	0.0342
post-LVC ectasia *vs* CONTROL_*3rdTR*_female, male	ENSG00000130748	*TMEM160*	0.8939	0.0419
post-LVC ectasia *vs* CONTROL_*3rdTR*_female, male	ENSG00000198794	*SCAMP5*	0.8964	0.0350
post-LVC ectasia *vs* CONTROL_*3rdTR*_female, male	ENSG00000103363	*ELOB*	0.9011	0.0408
post-LVC ectasia *vs* CONTROL_*3rdTR*_female, male	ENSG00000198242	*RPL23A*	0.9013	0.0481
post-LVC ectasia *vs* CONTROL_*3rdTR*_female, male	ENSG00000204889	*KRT40*	0.9013	0.0492
post-LVC ectasia *vs* CONTROL_*3rdTR*_female, male	ENSG00000161281	*COX7A1*	0.9013	0.0476
post-LVC ectasia *vs* CONTROL_*3rdTR*_female, male	ENSG00000125652	*ALKBH7*	0.9050	0.0386
post-LVC ectasia *vs* CONTROL_*3rdTR*_female, male	ENSG00000147465	*STAR*	0.9147	0.0422
post-LVC ectasia *vs* CONTROL_*3rdTR*_female, male	ENSG00000243749	*TMEM35B*	0.9156	0.0353
post-LVC ectasia *vs* CONTROL_*3rdTR*_female, male	ENSG00000182117	*NOP10*	0.9168	0.0374
post-LVC ectasia *vs* CONTROL_*3rdTR*_female, male	ENSG00000164587	*RPS14*	0.9245	0.0488
post-LVC ectasia *vs* CONTROL_*3rdTR*_female, male	ENSG00000284797	*AC008397.1*	0.9257	0.0401
post-LVC ectasia *vs* CONTROL_*3rdTR*_female, male	ENSG00000142684	*ZNF593*	0.9266	0.0296
post-LVC ectasia *vs* CONTROL_*3rdTR*_female, male	ENSG00000105640	*RPL18A*	0.9285	0.0426
post-LVC ectasia *vs* CONTROL_*3rdTR*_female, male	ENSG00000072041	*SLC6A15*	0.9315	0.0307
post-LVC ectasia *vs* CONTROL_*3rdTR*_female, male	ENSG00000221983	*UBA52*	0.9332	0.0416
post-LVC ectasia *vs* CONTROL_*3rdTR*_female, male	ENSG00000176340	*COX8A*	0.9415	0.0386
post-LVC ectasia *vs* CONTROL_*3rdTR*_female, male	ENSG00000083845	*RPS5*	0.9450	0.0421
post-LVC ectasia *vs* CONTROL_*3rdTR*_female, male	ENSG00000111640	*GAPDH*	0.9459	0.0457
post-LVC ectasia *vs* CONTROL_*3rdTR*_female, male	ENSG00000147804	*SLC39A4*	0.9466	0.0204
post-LVC ectasia *vs* CONTROL_*3rdTR*_female, male	ENSG00000267314	*AC104532.1*	0.9470	0.0342
post-LVC ectasia *vs* CONTROL_*3rdTR*_female, male	ENSG00000197191	*CYSRT1*	0.9476	0.0350
post-LVC ectasia *vs* CONTROL_*3rdTR*_female, male	ENSG00000274618	*H4C6*	0.9515	0.0384
post-LVC ectasia *vs* CONTROL_*3rdTR*_female, male	ENSG00000010030	*ETV7*	0.9577	0.0286
post-LVC ectasia *vs* CONTROL_*3rdTR*_female, male	ENSG00000100234	*TIMP3*	0.9577	0.0491
post-LVC ectasia *vs* CONTROL_*3rdTR*_female, male	ENSG00000267795	*SMIM22*	0.9603	0.0351
post-LVC ectasia *vs* CONTROL_*3rdTR*_female, male	ENSG00000127540	*UQCR11*	0.9689	0.0407
post-LVC ectasia *vs* CONTROL_*3rdTR*_female, male	ENSG00000089356	*FXYD3*	0.9717	0.0307
post-LVC ectasia *vs* CONTROL_*3rdTR*_female, male	ENSG00000231500	*RPS18*	0.9718	0.0174
post-LVC ectasia *vs* CONTROL_*3rdTR*_female, male	ENSG00000163923	*RPL39L*	0.9728	0.0364
post-LVC ectasia *vs* CONTROL_*3rdTR*_female, male	ENSG00000100300	*TSPO*	0.9728	0.0284
post-LVC ectasia *vs* CONTROL_*3rdTR*_female, male	ENSG00000184990	*SIVA1*	0.9743	0.0296
post-LVC ectasia *vs* CONTROL_*3rdTR*_female, male	ENSG00000175701	*MTLN*	0.9793	0.0211
post-LVC ectasia *vs* CONTROL_*3rdTR*_female, male	ENSG00000254806	*SYS1-DBNDD2*	0.9929	0.0380
post-LVC ectasia *vs* CONTROL_*3rdTR*_female, male	ENSG00000163682	*RPL9*	0.9966	0.0356
post-LVC ectasia *vs* CONTROL_*3rdTR*_female, male	ENSG00000171159	*C9orf16*	0.9982	0.0286
post-LVC ectasia *vs* CONTROL_*3rdTR*_female, male	ENSG00000010327	*STAB1*	1.0001	0.0497
post-LVC ectasia *vs* CONTROL_*3rdTR*_female, male	ENSG00000276045	*ORAI1*	1.0013	0.0021
post-LVC ectasia *vs* CONTROL_*3rdTR*_female, male	ENSG00000240972	*MIF*	1.0097	0.0324
post-LVC ectasia *vs* CONTROL_*3rdTR*_female, male	ENSG00000145879	*SPINK7*	1.0125	0.0412
post-LVC ectasia *vs* CONTROL_*3rdTR*_female, male	ENSG00000170276	*HSPB2*	1.0145	0.0364
post-LVC ectasia *vs* CONTROL_*3rdTR*_female, male	ENSG00000148671	*ADIRF*	1.0231	0.0323
post-LVC ectasia *vs* CONTROL_*3rdTR*_female, male	ENSG00000233493	*TMEM238*	1.0392	0.0174
post-LVC ectasia *vs* CONTROL_*3rdTR*_female, male	ENSG00000175600	*SUGCT*	1.0493	0.0011

post-LVC ectasia *vs* CONTROL_*3rdTR*_female, male	ENSG00000235169	*SMIM1*	1.0532	0.0174
post-LVC ectasia *vs* CONTROL_*3rdTR*_female, male	ENSG00000131686	*CA6*	1.0621	0.0488
post-LVC ectasia *vs* CONTROL_*3rdTR*_female, male	ENSG00000213741	*RPS29*	1.0682	0.0468
post-LVC ectasia *vs* CONTROL_*3rdTR*_female, male	ENSG00000205364	*MT1M*	1.0806	0.0426
post-LVC ectasia *vs* CONTROL_*3rdTR*_female, male	ENSG00000275714	*H3C1*	1.0918	0.0454
post-LVC ectasia *vs* CONTROL_*3rdTR*_female, male	ENSG00000160932	*LY6E*	1.0981	0.0174
post-LVC ectasia *vs* CONTROL_*3rdTR*_female, male	ENSG00000126264	*HCST*	1.1290	0.0466
post-LVC ectasia *vs* CONTROL_*3rdTR*_female, male	ENSG00000187608	*ISG15*	1.1437	0.0284
post-LVC ectasia *vs* CONTROL_*3rdTR*_female, male	ENSG00000273542	*H4C12*	1.1461	0.0342
post-LVC ectasia *vs* CONTROL_*3rdTR*_female, male	ENSG00000184845	*DRD1*	1.1657	0.0351
post-LVC ectasia *vs* CONTROL_*3rdTR*_female, male	ENSG00000131771	*PPP1R1B*	1.1782	0.0296
post-LVC ectasia *vs* CONTROL_*3rdTR*_female, male	ENSG00000167653	*PSCA*	1.1796	0.0477
post-LVC ectasia *vs* CONTROL_*3rdTR*_female, male	ENSG00000142694	*EVA1B*	1.1837	0.0387
post-LVC ectasia *vs* CONTROL_*3rdTR*_female, male	ENSG00000278828	*H3C10*	1.2014	0.0342
post-LVC ectasia *vs* CONTROL_*3rdTR*_female, male	ENSG00000106366	*SERPINE1*	1.2413	0.0347
post-LVC ectasia *vs* CONTROL_*3rdTR*_female, male	ENSG00000183625	*CCR3*	1.2733	0.0243
post-LVC ectasia *vs* CONTROL_*3rdTR*_female, male	ENSG00000139971	*ARMH4*	1.2845	0.0497
post-LVC ectasia *vs* CONTROL_*3rdTR*_female, male	ENSG00000259112	*NDUFC2-KCTD14*	1.3085	0.0286
post-LVC ectasia *vs* CONTROL_*3rdTR*_female, male	ENSG00000133124	*IRS4*	1.3179	0.0034
post-LVC ectasia *vs* CONTROL_*3rdTR*_female, male	ENSG00000188730	*VWC2*	1.3552	0.0457
post-LVC ectasia *vs* CONTROL_*3rdTR*_female, male	ENSG00000178934	*LGALS7B*	1.3637	0.0491
post-LVC ectasia *vs* CONTROL_*3rdTR*_female, male	ENSG00000132205	*EMILIN2*	1.4175	0.0478
post-LVC ectasia *vs* CONTROL_*3rdTR*_female, male	ENSG00000136688	*IL36G*	1.4219	0.0397
post-LVC ectasia *vs* CONTROL_*3rdTR*_female, male	ENSG00000258644	*SYNJ2BP-COX16*	1.4769	0.0230
post-LVC ectasia *vs* CONTROL_*3rdTR*_female, male	ENSG00000070886	*EPHA8*	1.6586	0.0203
post-LVC ectasia *vs* CONTROL_*3rdTR*_female, male	ENSG00000167754	*KLK5*	1.8456	0.0434
post-LVC ectasia *vs* CONTROL_*3rdTR*_female, male	ENSG00000163431	*LMOD1*	2.0385	0.0302
post-LVC ectasia *vs* CONTROL_*3rdTR*_female, male	ENSG00000181104	*F2R*	2.0858	0.0284
post-LVC ectasia *vs* CONTROL_*3rdTR*_female, male	ENSG00000286075	*AC009412.1*	2.6275	0.0421
post-LVC ectasia *vs* CONTROL_*3rdTR*_female, male	ENSG00000256514	*AP003419.1*	3.3318	0.0350

Abbreviations: post-LVC ectasia – post-laser vision correction ectasia; *1stTR – central topographic region*, *2ndTR – middle topographic region*, *3rdTR – peripheral topographic*; log2FC - log2 transformed fold change.

Table S7. Top differentially expressed genes (DEG) in post-LVC ectasia.

A list of the top 15 DEGs revealed from each particular *TRs* of the CE of patients with post-LVC ectasia (in comparison to control individuals). Selection was based on absolute log2FC values.

Comparison	Gene_id	Gene_name	Log2FC	Adjusted p-value
post-LVC ectasia *vs* CONTROL_*1stTR*_female	ENSG00000167916	*KRT24*	-5.07832	<0.0001
post-LVC ectasia *vs* CONTROL_*1stTR*_female	ENSG00000100427	*MLC1*	4.72020	0.03632
post-LVC ectasia *vs* CONTROL_*1stTR*_female	ENSG00000167754	*KLK5*	2.97415	0.00118
post-LVC ectasia *vs* CONTROL_*1stTR*_female	ENSG00000129455	*KLK8*	2.40420	0.00118
post-LVC ectasia *vs* CONTROL_*1stTR*_female	ENSG00000184489	*PTP4A3*	1.76216	0.00266
post-LVC ectasia *vs* CONTROL_*1stTR*_female	ENSG00000223783	*LINC01983*	1.69786	0.02043
post-LVC ectasia *vs* CONTROL_*1stTR*_female	ENSG00000262714	*AC007342.5*	-1.67492	0.02905
post-LVC ectasia *vs* CONTROL_*1stTR*_female	ENSG00000166402	*TUB*	1.55257	0.04835
post-LVC ectasia *vs* CONTROL_*1stTR*_female	ENSG00000198113	*TOR4A*	1.40664	0.00944
post-LVC ectasia *vs* CONTROL_*1stTR*_female	ENSG00000111981	*ULBP1*	1.32008	0.04835
post-LVC ectasia *vs* CONTROL_*1stTR*_female	ENSG00000179799	*OR7E22P*	1.20644	0.00701
post-LVC ectasia *vs* CONTROL_*1stTR*_female	ENSG00000168350	*DEGS2*	1.16461	0.02043
post-LVC ectasia *vs* CONTROL_*1stTR*_female	ENSG00000262681	*AC005722.3*	0.96904	0.02043
post-LVC ectasia *vs* CONTROL_*1stTR*_female	ENSG00000198911	*SREBF2*	-0.75924	0.00701
post-LVC ectasia *vs* CONTROL_*1stTR*_female, male	ENSG00000163618	*CADPS*	-2.35783	0.00444
post-LVC ectasia *vs* CONTROL_*1stTR*_female, male	ENSG00000251463	*FKBP4P1*	-1.84254	0.03653
post-LVC ectasia *vs* CONTROL_*1stTR*_female, male	ENSG00000272568	*AC005162.2*	-1.78332	0.03152
post-LVC ectasia *vs* CONTROL_*1stTR*_female, male	ENSG00000279516	*FAM230C*	-1.74190	0.03269
post-LVC ectasia *vs* CONTROL_*1stTR*_female, male	ENSG00000242889	*RN7SL449P*	-1.72208	0.04128
post-LVC ectasia *vs* CONTROL_*1stTR*_female, male	ENSG00000205002	*AARD*	-1.68664	0.03230
post-LVC ectasia *vs* CONTROL_*1stTR*_female, male	ENSG00000175699	*CCDC197*	-1.68480	0.04740
post-LVC ectasia *vs* CONTROL_*1stTR*_female, male	ENSG00000240808	*AC126389.1*	-1.62399	0.04740
post-LVC ectasia *vs* CONTROL_*1stTR*_female, male	ENSG00000280317	*AL732618.1*	-1.58369	0.04740
post-LVC ectasia *vs* CONTROL_*1stTR*_female, male	ENSG00000225675	*LINC01771*	-1.56171	0.04512
post-LVC ectasia *vs* CONTROL_*1stTR*_female, male	ENSG00000286571	*AL359511.2*	-1.47760	0.03624
post-LVC ectasia *vs* CONTROL_*1stTR*_female, male	ENSG00000080854	*IGSF9B*	-1.41611	0.03230
post-LVC ectasia *vs* CONTROL_*1stTR*_female, male	ENSG00000130052	*STARD8*	-1.37409	0.04415
post-LVC ectasia *vs* CONTROL_*1stTR*_female, male	ENSG00000272137	*AL451064.1*	-1.37078	0.04895
post-LVC ectasia *vs* CONTROL_*1stTR*_female, male	ENSG00000241985	*WWTR1-IT1*	-1.35411	0.04415
post-LVC ectasia *vs* CONTROL_*1stTR*_male	ENSG00000253653	*AC009185.1*	-1.41380	0.04429
post-LVC ectasia *vs* CONTROL_*1stTR*_male	ENSG00000285210	*LINC02795*	-0.88566	0.03855
post-LVC ectasia *vs* CONTROL_*1stTR*_male	ENSG00000189334	*S100A14*	0.84455	0.03842
post-LVC ectasia *vs* CONTROL_*1stTR*_male	ENSG00000168016	*TRANK1*	0.84834	0.02728
post-LVC ectasia *vs* CONTROL_*2ndTR*_female	ENSG00000167916	*KRT24*	-5.31540	<0.0001
post-LVC ectasia *vs* CONTROL_*2ndTR*_female	ENSG00000179799	*OR7E22P*	1.56400	0.00014
post-LVC ectasia *vs* CONTROL_*2ndTR*_female	ENSG00000167754	*KLK5*	2.76526	0.00687
post-LVC ectasia *vs* CONTROL_*2ndTR*_female, male	ENSG00000163618	*CADPS*	-2.02534	0.02115
post-LVC ectasia *vs* CONTROL_*2ndTR*_female, male	ENSG00000080854	*IGSF9B*	-1.65384	0.02115
post-LVC ectasia *vs* CONTROL_*2ndTR*_female, male	ENSG00000198768	*APCDD1L*	-0.96375	0.02115
post-LVC ectasia *vs* CONTROL_*2ndTR*_female, male	ENSG00000205560	*CPT1B*	-0.85079	0.02115
post-LVC ectasia *vs* CONTROL_*2ndTR*_female, male	ENSG00000110852	*CLEC2B*	0.98191	0.02115
post-LVC ectasia *vs* CONTROL_*2ndTR*_female, male	ENSG00000279692	*AC110285.6*	1.60878	0.02115
post-LVC ectasia *vs* CONTROL_*2ndTR*_female, male	ENSG00000251501	*AC114774.1*	1.65102	0.02115
post-LVC ectasia *vs* CONTROL_*2ndTR*_female, male	ENSG00000070886	*EPHA8*	1.90326	0.02115
post-LVC ectasia *vs* CONTROL_*3rdTR*_female	ENSG00000167916	*KRT24*	-5.48918	<0.0001
post-LVC ectasia *vs* CONTROL_*3rdTR*_female	ENSG00000198237	*AC131392.1*	-1.07225	0.02345
post-LVC ectasia *vs* CONTROL_*3rdTR*_female	ENSG00000196912	*ANKRD36B*	-0.92579	0.03217
post-LVC ectasia *vs* CONTROL_*3rdTR*_female	ENSG00000220323	*H2BC19P*	-0.87854	0.03750
post-LVC ectasia *vs* CONTROL_*3rdTR*_female	ENSG00000198911	*SREBF2*	-0.62916	0.03750
post-LVC ectasia *vs* CONTROL_*3rdTR*_female	ENSG00000137936	*BCAR3*	0.75690	0.04595
post-LVC ectasia *vs* CONTROL_*3rdTR*_female	ENSG00000197417	*SHPK*	0.81995	0.02320
post-LVC ectasia *vs* CONTROL_*3rdTR*_female	ENSG00000008323	*PLEKHG6*	0.86833	0.02320

post-LVC ectasia *vs* CONTROL_*3rdTR*_female	ENSG00000131941	*RHPN2*	0.89020	0.02345
post-LVC ectasia *vs* CONTROL_*3rdTR*_female	ENSG00000172183	*ISG20*	0.94460	0.02320
post-LVC ectasia *vs* CONTROL_*3rdTR*_female	ENSG00000287929	*AL354953.1*	1.03472	0.02450
post-LVC ectasia *vs* CONTROL_*3rdTR*_female	ENSG00000111981	*ULBP1*	1.30696	0.04590
post-LVC ectasia *vs* CONTROL_*3rdTR*_female	ENSG00000168350	*DEGS2*	1.35333	0.00667
post-LVC ectasia *vs* CONTROL_*3rdTR*_female	ENSG00000276045	*ORAI1*	1.41133	0.03499
post-LVC ectasia *vs* CONTROL_*3rdTR*_female	ENSG00000184489	*PTP4A3*	1.46824	0.03144
post-LVC ectasia *vs* CONTROL_*3rdTR*_female, male	ENSG00000280893	*AC009133.6*	-3.77795	0.04448
post-LVC ectasia *vs* CONTROL_*3rdTR*_female, male	ENSG00000101204	*CHRNA4*	-3.58198	0.04029
post-LVC ectasia *vs* CONTROL_*3rdTR*_female, male	ENSG00000179796	*LRRC3B*	-3.48046	0.01741
post-LVC ectasia *vs* CONTROL_*3rdTR*_female, male	ENSG00000152583	*SPARCL1*	-3.30632	0.03069
post-LVC ectasia *vs* CONTROL_*3rdTR*_female, male	ENSG00000251487	*AC106744.2*	-2.82601	0.02861
post-LVC ectasia *vs* CONTROL_*3rdTR*_female, male	ENSG00000262655	*SPON1*	-2.76046	0.04538
post-LVC ectasia *vs* CONTROL_*3rdTR*_female, male	ENSG00000164588	*HCN1*	-2.67106	0.03422
post-LVC ectasia *vs* CONTROL_*3rdTR*_female, male	ENSG00000115896	*PLCL1*	-2.61781	0.02958
post-LVC ectasia *vs* CONTROL_*3rdTR*_female, male	ENSG00000267143	*AP001120.2*	-2.58015	0.02861
post-LVC ectasia *vs* CONTROL_*3rdTR*_female, male	ENSG00000274642	*AC244669.2*	-2.34063	0.04166
post-LVC ectasia *vs* CONTROL_*3rdTR*_female, male	ENSG00000253604	*AC131254.2*	-2.29217	0.03859
post-LVC ectasia *vs* CONTROL_*3rdTR*_female, male	ENSG00000196159	*FAT4*	-2.24165	0.01741
post-LVC ectasia *vs* CONTROL_*3rdTR*_female, male	ENSG00000226668	*AL136981.1*	-2.21497	0.01832
post-LVC ectasia *vs* CONTROL_*3rdTR*_female, male	ENSG00000146592	*CREB5*	-2.10295	0.04571
post-LVC ectasia *vs* CONTROL_*3rdTR*_female, male	ENSG00000255794	*RMST*	-2.08134	0.02678
post-LVC ectasia *vs* CONTROL_*3rdTR*_female, male	ENSG00000179583	*CIITA*	-0.7448	0.0286

Abbreviations: post-LVC ectasia – post-laser vision correction ectasia; *1^{st}TR – central topographic region*, *2^{nd}TR – middle topographic region*, *3^{rd}TR – peripheral topographic*; log2FC - log2 transformed fold change.

Table S8. The hallmark pathways differentially enriched in post-LVC ectasia.

The hallmark pathways differentially enriched in particular *TRs* of the CE of patients with post-LVC ectasia, in comparison to corresponding *TRs* of the CE of control individuals. Enrichment analysis was performed in three settings depending on the sex of the individuals included. Empty cells mean a lack of changes.

Hallmark	Comparison	post-LVC ectasia females&males			post-LVC ectasia females			post-LVC ectasia males		
		1st TR	*2nd TR*	*3rd TR*	*1st TR*	*2nd TR*	*3rd TR*	*1st TR*	*2nd TR*	*3rd TR*
ADIPOGENESIS	directon of change	UP	UP	UP	UP	UP	UP	UP	UP	UP
	FDR value	8.94E-11	1.14E-08	5.88E-09	1.02E-09	5.49E-08	3.26E-09	3.16E-08	0.0002	0.0004
ALLOGRAFT REJECTION	directon of change	UP	UP	UP				UP	UP	
	FDR value	0.0072	0.0309	0.0413				0.0005	0.0080	
ANDROGEN RESPONSE	directon of change	UP	UP	UP	UP	UP	UP	UP	UP	
	FDR value	9.14E-05	0.0003	0.0029	0.0004	0.0008	0.0044	0.0005	0.0134	
ANGIOGENESIS	directon of change	UP	UP	UP	UP			UP	UP	
	FDR value	0.0030	0.0193	0.0353	0.0319			0.0009	0.0308	
APICAL JUNCTION	directon of change	UP						UP		
	FDR value	0.0290						0.0073		
APICAL SURFACE	directon of change									
	FDR value									
APOPTOSIS	directon of change	UP	UP	UP	UP	UP	UP	UP	UP	UP
	FDR value	8.17E-09	1.64E-07	7.65E-07	3.13E-07	1.85E-06	1.42E-06	7.28E-07	0.0013	0.0063
BILE ACID METABOLISM	directon of change	UP	UP	UP	UP	UP	UP		UP	
	FDR value	0.0217	0.0144	0.0144	0.0166	0.0374	0.0080		0.0301	
CHOLESTEROL HOMEOSTASIS	directon of change	UP	UP	UP					UP	UP
	FDR value	0.0024	0.0462	0.0152				0.0014		0.0266
COAGULATION	directon of change	UP	UP	UP	UP	UP	UP	UP		
	FDR value	1.34E-05	0.0007	0.0008	0.0008	0.0027	0.0015	0.0001		
COMPLEMENT	directon of change	UP	UP	UP	UP	UP	UP	UP	UP	UP
	FDR value	0.0004	0.0007	0.0008	0.0056	0.0034	0.0197	0.0002	0.0067	0.0017
DNA REPAIR	directon of change	UP	UP	UP	UP	UP	UP	UP	UP	UP
	FDR value	1.09E-10	1.45E-09	5.89E-10	5.5E-10	1.25E-08	1.02E-09	1.13E-07	0.0004	0.0003
E2F TARGETS	directon of change	UP	UP	UP	UP	UP	UP	UP	UP	UP
	FDR value	2.36E-10	8.43E-12	4.76E-07	5.83E-07	4.11E-10	0.000166	1.02E-10	1.62E-07	1.87E-05
EPITHELIAL MESENCHYMAL TRANSITION	directon of change	UP	UP					UP		
	FDR value	0.0009	0.0214					6.78E-05		
ESTROGEN RESPONSE EARLY	directon of change	UP	UP	UP	UP	UP	UP	UP		UP
	FDR value	0.0007	0.0184	0.0075	0.0017	0.0119	0.0039	0.0006		0.0492
ESTROGEN RESPONSE LATE	directon of change	UP	UP	UP	UP	UP	UP	UP	UP	UP
	FDR value	1.23E-07	9.26E-06	7.26E-06	1.42E-06	2.94E-06	1.54E-06	8.46E-07	0.0033	0.0039
FATTY ACID METABOLISM	directon of change	UP	UP	UP	UP	UP	UP	UP	UP	UP
	FDR value	5.31E-08	8.21E-07	1.59E-06	1.15E-06	2.3E-06	1.04E-06	5.46E-07	0.0007	0.0024
G2M CHECKPOINT	directon of change	UP	UP	UP	UP	UP	UP	UP	UP	UP
	FDR value	4.94E-06	3.59E-06	0.0023	0.0003	3.94E-06	0.0450	1.4E-06	0.0011	0.0012
GLYCOLYSIS	directon of change	UP	UP	UP	UP	UP	UP	UP	UP	UP
	FDR value	2.86E-09	7.9E-08	2.48E-07	1.18E-07	1.17E-06	1.07E-06	1.21E-07	5.43E-05	0.0002
HEDGEHOG SIGNALING	directon of change									
	FDR value									
HEME METABOLISM	directon of change	UP	UP	UP	UP	UP	UP	UP	UP	UP
	FDR value	1.53E-05	0.0002	0.001232	1.72E-05	6.98E-05	0.0003	0.0002	0.0214	
HYPOXIA	directon of change	UP	UP	UP	UP	UP	UP	UP	UP	UP
	FDR value	9.26E-06	0.0001	0.0002	1.79E-05	0.0003	4.94E-05	8.65E-05	0.0077	0.0177
IL2 STAT5 SIGNALING	directon of change	UP	UP	UP	UP	UP	UP	UP	UP	UP
	FDR value	0.0004	0.0037	0.0015	0.0008	0.0017	0.0011	0.0007	0.0216	0.0355
IL6 JAK STAT3 SIGNALING	directon of change	UP		UP	UP		UP	UP		
	FDR value	0.0041		0.0462	0.0111		0.0421	0.0077		
INFLAMMATORY RESPONSE	directon of change	UP	UP	UP	UP	UP	UP	UP		
	FDR value	0.0072	0.0229	0.0153	0.0327	0.0170	0.0091	0.0032		
INTERFERON ALPHA RESPONSE	directon of change	UP	UP	UP	UP	UP	UP	UP	UP	UP
	FDR value	4.46E-07	1.59E-06	2.24E-06	0.0017	0.0197	0.0005	2.42E-11	2.34E-09	1.61E-06
INTERFERON GAMMA RESPONSE	directon of change	UP	UP	UP	UP	UP	UP	UP	UP	UP
	FDR value	1.1E-07	2.33E-06	1E-05	0.0003	0.0070	0.0014	2.42E-11	7.5E-09	1.39E-05

KRAS SIGNALING UP	directon of change<br>FDR value	UP<br>0.0023	UP<br>0.0045		UP<br>0.0029	UP<br>0.0019	UP<br>0.0347	UP<br>0.0027		
KRAS SIGNALING DOWN	directon of change<br>FDR value									
MITOTIC SPINDLE	directon of change<br>FDR value							UP<br>0.0152	UP<br>0.0383	UP<br>0.0312
MTORC1 SIGNALING	directon of change<br>FDR value	UP<br>2.18E-12	UP<br>2.18E-11	UP<br>6.71E-09	UP<br>6.53E-09	UP<br>1.02E-09	UP<br>3.78E-07	UP<br>1.6E-11	UP<br>1.68E-06	UP<br>2.4E-05
MYC TARGETS V1	directon of change<br>FDR value	UP<br>5.54E-20	UP<br>4.66E-21	UP<br>5.3E-15	UP<br>5.66E-18	UP<br>4.71E-20	UP<br>2.6E-14	UP<br>5.27E-13	UP<br>3.21E-07	UP<br>0.0002
MYC TARGETS V2	directon of change<br>FDR value	UP<br>1.06E-09	UP<br>1.39E-07	UP<br>2.79E-08	UP<br>5.08E-10	UP<br>5.5E-10	UP<br>6.11E-09	UP<br>7.38E-07		UP<br>0.0018
MYOGENESIS	directon of change<br>FDR value	UP<br>0.0211					UP<br>0.0365	UP<br>0.0243		
NOTCH SIGNALING	directon of change<br>FDR value									
OXIDATIVE PHOSPHORYLATION	directon of change<br>FDR value	UP<br>4.66E-21	UP<br>2.29E-19	UP<br>4.66E-21	UP<br>1.91E-21	UP<br>1.64E-19	UP<br>1.91E-21	UP<br>1.09E-10	UP<br>0.0001	UP<br>5.46E-07
P53 PATHWAY	directon of change<br>FDR value	UP<br>3.42E-10	UP<br>3.79E-07	UP<br>1.42E-09	UP<br>4.12E-09	UP<br>1.18E-07	UP<br>1.69E-10	UP<br>1.76E-08	UP<br>0.0029	UP<br>0.0002
PANCREAS BETA CELLS	directon of change<br>FDR value									DOWN<br>0.0472
PEROXISOME	directon of change<br>FDR value	UP<br>3.25E-06	UP<br>9.25E-05	UP<br>4.34E-05	UP<br>1.67E-05	UP<br>7.79E-05	UP<br>3.03E-05	UP<br>1.27E-05	UP<br>0.0060	UP<br>0.0084
PI3K AKT MTOR SIGNALING	directon of change<br>FDR value	UP<br>8.99E-06	UP<br>0.0005	UP<br>0.0002	UP<br>4.91E-05	UP<br>0.0006	UP<br>0.0004	UP<br>4.08E-05	UP<br>0.0385	UP<br>0.0067
PROTEIN SECRETION	directon of change<br>FDR value	UP<br>1.87E-08	UP<br>1.05E-10	UP<br>8.9E-07	UP<br>3E-09	UP<br>2.35E-09	UP<br>4.64E-06	UP<br>5.45E-05	UP<br>2.4E-05	UP<br>0.001469
REACTIVE OXYGEN SPECIES PATHWAY	directon of change<br>FDR value	UP<br>2.31E-06	UP<br>0.0001	UP<br>4.62E-06	UP<br>4.66E-06	UP<br>0.0001	UP<br>1.3E-05	UP<br>0.0004		UP<br>0.0071
SPERMATOGENESIS	directon of change<br>FDR value		UP<br>0.0398							
TGF BETA SIGNALING	directon of change<br>FDR value	UP<br>0.0014	UP<br>0.0005	UP<br>0.0026	UP<br>0.0004	UP<br>0.0003	UP<br>0.0029	UP<br>0.0205	UP<br>0.0355	UP<br>0.0216
TNFA SIGNALING VIA NFKB	directon of change<br>FDR value	UP<br>2.48E-07	UP<br>4.1E-06	UP<br>2.04E-06	UP<br>2.7E-07	UP<br>1.63E-06	UP<br>7.22E-07	UP<br>3.62E-05	UP<br>0.0167	UP<br>0.0063
UNFOLDED PROTEIN RESPONSE	directon of change<br>FDR value	UP<br>1.38E-09	UP<br>8.11E-09	UP<br>1.02E-06	UP<br>2.88E-09	UP<br>2.32E-08	UP<br>5.08E-07	UP<br>3.21E-07	UP<br>0.0003	UP<br>0.0067
UV RESPONSE UP	directon of change<br>FDR value	UP<br>4.88E-10	UP<br>2.18E-07	UP<br>1.23E-07	UP<br>3.83E-08	UP<br>5.65E-07	UP<br>5.52E-08	UP<br>1.13E-08	UP<br>0.0030	UP<br>0.0055
UV RESPONSE DOWN	directon of change<br>FDR value									
WNT BETA CATENIN SIGNALING	directon of change<br>FDR value	UP<br>0.0462						UP<br>0.0183		
XENOBIOTIC METABOLISM	directon of change<br>FDR value	UP<br>2.04E-06	UP<br>0.0002	UP<br>4.26E-05	UP<br>4.99E-05	UP<br>0.0008	UP<br>4.95E-05	UP<br>4E-06	UP<br>0.0055	UP<br>0.0158

Abbreviations: post-LVC ectasia – post-laser vision correction ectasia; *1st TR – central topographic region*, *2nd TR – middle topographic region*, *3rd TR – peripheral topographic*

Table S9. The REACTOME pathways differentially enriched in post-LVC ectasia.

The REACTOME pathways differentially enriched in particular *TRs* of the CE of patients with post-LVC ectasia, in comparison to corresponding *TRs* of the CE of control individuals. Enrichment analysis was performed in three settings depending on the sex of the individuals included and for each topographic region separately. Top 10 differentiating pathways (based on FDR value) for each comparison are presented and all results are shared in Mendeley Data Repository (doi: 10.17632/p656wtzjv8.1).

	Pathway Name	Directon of change	P-value	FDR
post-LVC ectasia *vs* CONTROL *1st TR*_female, male	TRANSLATION	Up	1.72E-26	4.16E-23
	SRP_DEPENDENT_COTRANSLATIONAL_PROTEIN_TARGETING_TO_MEMBRANE	Up	7.26E-22	4.40E-19
	EUKARYOTIC_TRANSLATION_INITIATION	Up	2.60E-21	1.05E-18
	RRNA_PROCESSING	Up	5.18E-21	1.79E-18
	EUKARYOTIC_TRANSLATION_ELONGATION	Up	1.26E-20	3.59E-18
	REGULATION_OF_EXPRESSION_OF_SLITS_AND_ROBOS	Up	2.71E-20	6.92E-18
	RESPONSE_OF_EIF2AK4_GCN2_TO_AMINO_ACID_DEFICIENCY	Up	8.71E-20	2.01E-17
	DNA_REPLICATION_PRE_INITIATION	Up	4.09E-19	8.62E-17
	MITOCHONDRIAL_TRANSLATION	Up	1.12E-18	1.94E-16
	RUNX1_REGULATES_TRANSCRIPTION_OF_GENES_INVOLVED_IN_DIFFERENTIATION_OF_HSCS	Up	1.21E-18	2.01E-16
post-LVC ectasia *vs* CONTROL *2nd TR*_female, male	TRANSLATION	Up	2.84E-27	1.38E-23
	SRP_DEPENDENT_COTRANSLATIONAL_PROTEIN_TARGETING_TO_MEMBRANE	Up	2.41E-24	2.92E-21
	EUKARYOTIC_TRANSLATION_INITIATION	Up	1.98E-23	1.82E-20
	EUKARYOTIC_TRANSLATION_ELONGATION	Up	2.26E-23	1.82E-20
	RESPONSE_OF_EIF2AK4_GCN2_TO_AMINO_ACID_DEFICIENCY	Up	9.53E-22	5.13E-19
	RRNA_PROCESSING	Up	1.70E-21	8.23E-19
	REGULATION_OF_EXPRESSION_OF_SLITS_AND_ROBOS	Up	3.23E-21	1.20E-18
	INFLUENZA_INFECTION	Up	2.11E-20	5.68E-18
	MITOCHONDRIAL_TRANSLATION	Up	7.77E-19	1.45E-16
	SELENOAMINO_ACID_METABOLISM	Up	9.43E-19	1.69E-16
post-LVC ectasia *vs* CONTROL *3rd TR*_female, male	TRANSLATION	Up	3.49E-25	5.63E-22
	SRP_DEPENDENT_COTRANSLATIONAL_PROTEIN_TARGETING_TO_MEMBRANE	Up	7.75E-23	5.37E-20
	EUKARYOTIC_TRANSLATION_ELONGATION	Up	2.55E-21	1.05E-18
	EUKARYOTIC_TRANSLATION_INITIATION	Up	7.21E-21	2.33E-18
	RESPONSE_OF_EIF2AK4_GCN2_TO_AMINO_ACID_DEFICIENCY	Up	8.21E-21	2.49E-18
	RRNA_PROCESSING	Up	7.24E-20	1.75E-17
	DNA_REPLICATION_PRE_INITIATION	Up	3.31E-19	7.29E-17
	REGULATION_OF_EXPRESSION_OF_SLITS_AND_ROBOS	Up	6.78E-19	1.37E-16
	INFLUENZA_INFECTION	Up	7.39E-19	1.43E-16
	MITOCHONDRIAL_TRANSLATION	Up	1.67E-18	2.61E-16

post-LVC ectasia *vs* CONTROL *1st TR*_female	TRANSLATION	Up	4.36E-26	2.11E-22
	RRNA_PROCESSING	Up	1.48E-21	1.43E-18
	MITOCHONDRIAL_TRANSLATION	Up	1.32E-19	7.97E-17
	EUKARYOTIC_TRANSLATION_INITIATION	Up	3.68E-19	1.52E-16
	SRP_DEPENDENT_COTRANSLATIONAL_PROTEIN_TARGETING_TO_MEMBRANE	Up	4.32E-19	1.61E-16
	EUKARYOTIC_TRANSLATION_ELONGATION	Up	1.78E-18	5.39E-16
	RESPONSE_OF_EIF2AK4_GCN2_TO_AMINO_ACID_DEFICIENCY	Up	9.06E-18	1.91E-15
	REGULATION_OF_EXPRESSION_OF_SLITS_AND_ROBOS	Up	1.05E-17	2.11E-15
	INFLUENZA_INFECTION	Up	1.53E-17	2.85E-15
	METABOLISM_OF_RNA	Up	1.92E-17	3.44E-15
post-LVC ectasia *vs* CONTROL *2nd TR*_female	TRANSLATION	Up	9.28E-26	2.25E-22
	RRNA_PROCESSING	Up	1.26E-21	1.43E-18
	SRP_DEPENDENT_COTRANSLATIONAL_PROTEIN_TARGETING_TO_MEMBRANE	Up	2.63E-20	2.11E-17
	EUKARYOTIC_TRANSLATION_INITIATION	Up	3.04E-20	2.11E-17
	EUKARYOTIC_TRANSLATION_ELONGATION	Up	3.76E-19	1.52E-16
	REGULATION_OF_EXPRESSION_OF_SLITS_AND_ROBOS	Up	2.49E-18	7.11E-16
	MITOCHONDRIAL_TRANSLATION	Up	2.89E-18	7.79E-16
	RESPONSE_OF_EIF2AK4_GCN2_TO_AMINO_ACID_DEFICIENCY	Up	4.78E-18	1.10E-15
	INFLUENZA_INFECTION	Up	6.96E-18	1.53E-15
	METABOLISM_OF_RNA	Up	1.15E-17	2.22E-15
post-LVC ectasia *vs* CONTROL *3rd TR*_female	TRANSLATION	Up	9.90E-25	1.60E-21
	RRNA_PROCESSING	Up	2.93E-19	1.44E-16
	MITOCHONDRIAL_TRANSLATION	Up	2.97E-19	1.44E-16
	SRP_DEPENDENT_COTRANSLATIONAL_PROTEIN_TARGETING_TO_MEMBRANE	Up	8.22E-19	2.85E-16
	EUKARYOTIC_TRANSLATION_INITIATION	Up	1.59E-18	5.12E-16
	EUKARYOTIC_TRANSLATION_ELONGATION	Up	3.31E-18	8.43E-16
	RESPONSE_OF_EIF2AK4_GCN2_TO_AMINO_ACID_DEFICIENCY	Up	4.55E-18	1.10E-15
	REGULATION_OF_EXPRESSION_OF_SLITS_AND_ROBOS	Up	2.20E-16	3.68E-14
	THE_CITRIC_ACID_TCA_CYCLE_AND_RESPIRATORY_ELECTRON_TRANSPORT	Up	8.80E-16	1.42E-13
	RESPIRATORY_ELECTRON_TRANSPORT	Up	1.90E-15	2.63E-13
post-LVC ectasia *vs* CONTROL *1st TR*_male	DNA_REPLICATION_PRE_INITIATION	Up	4.55E-20	2.20E-16
	DNA_REPLICATION	Up	1.05E-18	2.54E-15
	SRP_DEPENDENT_COTRANSLATIONAL_PROTEIN_TARGETING_TO_MEMBRANE	Up	3.37E-18	4.32E-15
	RUNX1_REGULATES_TRANSCRIPTION_OF_GENES_INVOLVED_IN_DIFFERENTIATION_OF_HSCS	Up	3.57E-18	4.32E-15
	DNA_METHYLATION	Up	6.03E-18	5.42E-15
	SIRT1_NEGATIVELY_REGULATES_RRNA_EXPRESSION	Up	6.72E-18	5.42E-15
	EUKARYOTIC_TRANSLATION_INITIATION	Up	9.11E-18	6.30E-15
	TRANSLATION	Up	1.25E-17	7.55E-15

	EUKARYOTIC_TRANSLATION_ELONGATION	Up	2.53E-17	1.23E-14
	CONDENSATION_OF_PROPHASE_CHROMOSOMES	Up	4.04E-17	1.78E-14
post-LVC ectasia *vs* CONTROL *2nd TR*_male	EUKARYOTIC_TRANSLATION_ELONGATION	Up	1.76E-13	1.64E-11
	SRP_DEPENDENT_COTRANSLATIONAL_PROTEIN_TARGETING_TO_MEMBRANE	Up	1.90E-13	1.70E-11
	RESPONSE_OF_EIF2AK4_GCN2_TO_AMINO_ACID_DEFICIENCY	Up	2.59E-12	1.68E-10
	EUKARYOTIC_TRANSLATION_INITIATION	Up	4.68E-12	2.70E-10
	DNA_REPLICATION	Up	3.75E-11	1.57E-09
	INFLUENZA_INFECTION	Up	4.46E-11	1.83E-09
	DNA_REPLICATION_PRE_INITIATION	Up	1.25E-10	4.63E-09
	TRANSLATION	Up	2.09E-10	7.46E-09
	NONSENSE_MEDIATED_DECAY_NMD	Up	2.79E-10	9.72E-09
	REGULATION_OF_EXPRESSION_OF_SLITS_AND_ROBOS	Up	3.55E-10	1.18E-08
post-LVC ectasia *vs* CONTROL *3rd TR*_male	ASSEMBLY_OF_THE_ORC_COMPLEX_AT_THE_ORIGIN_OF_REPLICATION	Up	1.90E-17	1.02E-14
	SIRT1_NEGATIVELY_REGULATES_RRNA_EXPRESSION	Up	8.02E-17	2.77E-14
	ACTIVATED_PKN1_STIMULATES_TRANSCRIPTION_OF_AR_ANDROGEN_RECEPTOR_REGULATED_GENES_KLK2_AND_KLK3	Up	3.32E-16	8.94E-14
	MEIOTIC_RECOMBINATION	Up	3.51E-16	8.94E-14
	DNA_REPLICATION_PRE_INITIATION	Up	4.99E-16	1.21E-13
	DNA_METHYLATION	Up	7.03E-16	1.49E-13
	CONDENSATION_OF_PROPHASE_CHROMOSOMES	Up	3.24E-15	5.41E-13
	TRANSCRIPTIONAL_REGULATION_BY_SMALL_RNAS	Up	1.08E-14	1.59E-12
	DNA_REPLICATION	Up	2.23E-14	2.95E-12
	PRC2_METHYLATES_HISTONES_AND_DNA	Up	2.25E-14	2.95E-12

Abbreviations: post-LVC ectasia – post-laser vision correction ectasia; *1stTR – central topographic region*, *2ndTR – middle topographic region*, *3rdTR – peripheral topographic*; log2FC - log2 transformed fold change.

Table S10. Differentially expressed genes (DEGs) between post-LVC ectasia and KTCN. The DEGs in the particular *TRs* of the CE of patients with post-LVC ectasia compared to adequate the *TRs* of the CE of patients with KTCN. The compared *topographic regions* with the sex of included patients, Ensembl gene identifier ('Gene_id'), gene name, log2FC, and adjusted p-value are indicated.

Comparison	Gene_id	Gene_name	Log2FC	Adjusted p-value
post-LVC ectasia *vs* KTCN_*3rd TR*_female, male	ENSG00000265252	*MIR3132*	-1.2645	0.04878
post-LVC ectasia *vs* KTCN_*3rd TR*_female, male	ENSG00000260182	*AC120498.2*	-1.4655	0.04878
post-LVC ectasia *vs* KTCN_*3rd TR*_female, male	ENSG00000258549	*AL161713.1*	-1.1511	0.04878
post-LVC ectasia *vs* KTCN_*3rd TR*_female, male	ENSG00000226515	*AC004386.2*	-1.1691	0.04878
post-LVC ectasia *vs* KTCN_*3rd TR*_female, male	ENSG00000252491	*RNU1-142P*	-1.1560	0.04878
post-LVC ectasia *vs* KTCN_*3rd TR*_female, male	ENSG00000232925	*MRPS16P2*	-1.1811	0.04878
post-LVC ectasia *vs* KTCN_*3rd TR*_female, male	ENSG00000235043	*TECRP1*	-1.2370	0.04878

Abbreviations: post-LVC ectasia – post-laser vision correction ectasia; KTCN- keratoconus; *3rdTR – peripheral topographic*; log2FC - log2 transformed fold change

Table S11. The hallmark pathways differentially enriched between post-LVC ectasia and KTCN.

The hallmark pathways differentially enriched in particular *TRs* of the CE of patients with post-LVC ectasia, in comparison to corresponding *TRs* of the CE of patients with KTCN. Enrichment analysis was performed in two settings depending on the sex of the individuals included. Empty cells mean a lack of changes.

Hallmark	Comparison	Post-LVC ectasia females&males			Post-LVC ectasia males		
		1st TR	*2nd TR*	*3rd TR*	*1st TR*	*2nd TR*	*3rd TR*
ADIPOGENESIS	directon of change<br>FDR value						
ALLOGRAFT REJECTION	directon of change<br>FDR value						
ANDROGEN RESPONSE	directon of change<br>FDR value						
ANGIOGENESIS	directon of change<br>FDR value						
APICAL JUNCTION	directon of change<br>FDR value		DOWN<br>0.0269				
APICAL SURFACE	directon of change<br>FDR value						
APOPTOSIS	directon of change<br>FDR value						
BILE ACID METABOLISM	directon of change<br>FDR value						
CHOLESTEROL HOMEOSTASIS	directon of change<br>FDR value	0.0019					
COAGULATION	directon of change<br>FDR value						
COMPLEMENT	directon of change<br>FDR value						
DNA REPAIR	directon of change<br>FDR value						UP<br>0.0408
E2F TARGETS	directon of change<br>FDR value	DOWN<br>4.05e-05			DOWN<br>0.0250		
EPITHELIAL MESENCHYMAL TRANSITION	directon of change<br>FDR value		DOWN<br>0.0369				
ESTROGEN RESPONSE EARLY	directon of change<br>FDR value						
ESTROGEN RESPONSE LATE	directon of change<br>FDR value			DOWN<br>0.0269			
FATTY ACID METABOLISM	directon of change<br>FDR value						
G2M CHECKPOINT	directon of change<br>FDR value	DOWN<br>1.45e-05			DOWN<br>0.01354		
GLYCOLYSIS	directon of change<br>FDR value						
HEDGEHOG SIGNALING	directon of change<br>FDR value						
HEME METABOLISM	directon of change<br>FDR value						
HYPOXIA	directon of change<br>FDR value						
IL2 STAT5 SIGNALING	directon of change<br>FDR value						
IL6 JAK STAT3 SIGNALING	directon of change<br>FDR value		DOWN<br>0.0010			DOWN<br>0.0383	
INFLAMMATORY RESPONSE	directon of change<br>FDR value						
INTERFERON ALPHA RESPONSE	directon of change<br>FDR value	DOWN<br>5.28e-10	DOWN<br>5.467e-09		DOWN<br>6.42e-05	DOWN<br>0.0136	
INTERFERON GAMMA RESPONSE	directon of change<br>FDR value	DOWN<br>0.0003	DOWN<br>2.73e-05				
	directon of change						

KRAS SIGNALING UP	FDR value		
KRAS SIGNALING DOWN	directon of change<br>FDR value		
MITOTIC SPINDLE	directon of change<br>FDR value		
MTORC1 SIGNALING	directon of change<br>FDR value	DOWN<br>0.0213	
MYC TARGETS V1	directon of change<br>FDR value	DOWN<br>0.0305	DOWN<br>0.0383
MYC TARGETS V2	directon of change<br>FDR value	UP<br>0.0019	
MYOGENESIS	directon of change<br>FDR value		
NOTCH SIGNALING	directon of change<br>FDR value		
OXIDATIVE PHOSPHORYLATION	directon of change<br>FDR value	UP<br>0.0305	
P53 PATHWAY	directon of change<br>FDR value	UP<br>0.0433	
PANCREAS BETA CELLS	directon of change<br>FDR value	DOWN<br>0.0063	
PEROXISOME	directon of change<br>FDR value		
PI3K AKT MTOR SIGNALING	directon of change<br>FDR value		
PROTEIN SECRETION	directon of change<br>FDR value	DOWN<br>0.0269	
REACTIVE OXYGEN SPECIES PATHWAY	directon of change<br>FDR value		
SPERMATOGENESIS	directon of change<br>FDR value		
TGF BETA SIGNALING	directon of change<br>FDR value		
TNFA SIGNALING VIA NFKB	directon of change<br>FDR value		
UNFOLDED PROTEIN RESPONSE	directon of change<br>FDR value		
UV RESPONSE UP	directon of change<br>FDR value		
UV RESPONSE DOWN	directon of change<br>FDR value		
WNT BETA CATENIN SIGNALING	directon of change<br>FDR value		
XENOBIOTIC METABOLISM	directon of change<br>FDR value		

Abbreviations: post-LVC ectasia – post-laser vision correction ectasia; KTCN- keratoconus; *1stTR – central topographic 1*

Table S12. The REACTOME pathways differentially enriched between post-LVC ectasia and KTCN.

The REACTOME pathways differentially enriched in particular *TRs* of the CE of patients with post-LVC ectasia, in comparison to corresponding *TRs* of the CE of patients with KTCN. Enrichment analysis was performed in two settings depending on the sex of the individuals included and for each topographic region separately. Top 10 differentiating pathways (based on FDR value) for each comparison are presented and all results are shared in Mendeley Data Repository (doi: 10.17632/p656wtzjv8.1).

	Pathway Name	Directon of change	P-value	FDR
post-LVC ectasia *vs* KTCN 1st *TR*_female, male	INTERFERON_ALPHA_BETA_SIGNALING	Down	2.0E-10	6.2E-08
	CHOLESTEROL_BIOSYNTHESIS	Down	3.3E-10	8.4E-08
	RESOLUTION_OF_SISTER_CHROMATID_COHESION	Down	8.1E-08	1.0E-05
	CELL_CYCLE_CHECKPOINTS	Down	9.0E-08	1.1E-05
	INTERFERON_SIGNALING	Down	1.2E-07	1.4E-05
	MITOTIC_METAPHASE_AND_ANAPHASE	Down	4.4E-07	4.1E-05
	DNA_METHYLATION	Down	4.6E-07	4.1E-05
	SEPARATION_OF_SISTER_CHROMATIDS	Down	5.2E-07	4.5E-05
	RHO_GTPASES_ACTIVATE_FORMINS	Down	6.1E-07	5.1E-05
	MITOTIC_SPINDLE_CHECKPOINT	Down	8.2E-07	6.7E-05
post-LVC ectasia *vs* KTCN 2nd *TR*_female, male	INTERFERON_ALPHA_BETA_SIGNALING	Down	3.5E-09	6.7E-07
	FORMATION_OF_THE_CORNIFIED_ENVELOPE	Down	7.7E-08	9.9E-06
	INTERFERON_SIGNALING	Down	2.7E-07	2.7E-05
	INTERFERON_GAMMA_SIGNALING	Down	2.7E-07	2.7E-05
	KERATINIZATION	Down	3.6E-07	3.5E-05
	THE_ROLE_OF_GTSE1_IN_G2_M_PROGRESSION_AFTER_G2_CHECKPOINT	Down	1.9E-05	1.0E-03
	ANTIGEN_PROCESSING_CROSS_PRESENTATION	Down	1.2E-04	4.8E-03
	RESPIRATORY_ELECTRON_TRANSPORT_ATP_SYNTHESIS_BY_CHEMIOSMOTIC_COUPLING_AND_HEAT_PRODUCTION_BY_ UNCOUPLING_PROTEINS	Down	1.4E-04	5.5E-03
	INWARDLY_RECTIFYING_K_CHANNELS	Down	1.5E-04	5.8E-03
	CROSS_PRESENTATION_OF_SOLUBLE_EXOGENOUS_ANTIGENS_ENDOSOMES	Down	1.5E-04	5.8E-03
post-LVC ectasia *vs* KTCN 3rd *TR*_female, male	EUKARYOTIC_TRANSLATION_ELONGATION	Up	1.6E-17	7.6E-14
	RESPONSE_OF_EIF2AK4_GCN2_TO_AMINO_ACID_DEFICIENCY	Up	1.1E-14	2.6E-11
	SRP_DEPENDENT_COTRANSLATIONAL_PROTEIN_TARGETING_TO_MEMBRANE	Up	1.9E-14	3.0E-11
	DNA_METHYLATION	Up	5.9E-14	7.1E-11
	ACTIVATED_PKN1_STIMULATES_TRANSCRIPTION_OF_AR_ANDROGEN_RECEPTOR_REGULATED_GENES_KLK2_AND_KLK3	Up	1.8E-13	1.7E-10
	SIRT1_NEGATIVELY_REGULATES_RRNA_EXPRESSION	Up	9.1E-13	7.3E-10
	ASSEMBLY_OF_THE_ORC_COMPLEX_AT_THE_ORIGIN_OF_REPLICATION	Up	1.9E-12	1.3E-09
	SELENOAMINO_ACID_METABOLISM	Up	2.3E-12	1.4E-09
	EUKARYOTIC_TRANSLATION_INITIATION	Up	4.0E-12	2.1E-09
	MEIOTIC_RECOMBINATION	Up	1.3E-11	6.5E-09

post-LVC ectasia *vs* KTCN *1st TR*_male	INTERFERON_ALPHA_BETA_SIGNALING	Down	1.0E-05	8.2E-04
	ANTIVIRAL_MECHANISM_BY_IFN_STIMULATED_GENES	Down	3.3E-05	2.3E-03
	MITOTIC_METAPHASE_AND_ANAPHASE	Down	9.3E-05	6.1E-03
	INTERFERON_SIGNALING	Down	1.0E-04	6.5E-03
	APC_C_MEDIATED_DEGRADATION_OF_CELL_CYCLE_PROTEINS	Down	1.1E-04	7.0E-03
	CHOLESTEROL_BIOSYNTHESIS	Down	1.4E-04	8.3E-03
	SEPARATION_OF_SISTER_CHROMATIDS	Down	1.9E-04	1.1E-02
	THE_ROLE_OF_GTSE1_IN_G2_M_PROGRESSION_AFTER_G2_CHECKPOINT	Down	2.2E-04	1.2E-02
	APC_C_CDH1_MEDIATED_DEGRADATION_OF_CDC20_AND_OTHER_APC_C_CDH1_TARGETED_PROTEINS_IN_LATE_MITOSIS_ EARLY_G1	Down	3.0E-04	1.5E-02
	RESOLUTION_OF_SISTER_CHROMATID_COHESION	Down	3.8E-04	1.9E-02
post-LVC ectasia *vs* KTCN *2nd TR*_male	INWARDLY_RECTIFYING_K_CHANNELS	Down	3.6E-05	2.5E-03
	THE_ROLE_OF_GTSE1_IN_G2_M_PROGRESSION_AFTER_G2_CHECKPOINT	Down	8.8E-05	5.8E-03
	FORMATION_OF_THE_CORNIFIED_ENVELOPE	Down	1.1E-04	7.0E-03
	RESPIRATORY_ELECTRON_TRANSPORT_ATP_SYNTHESIS_BY_CHEMIOSMOTIC_COUPLING_AND_HEAT_PRODUCTION_BY_ UNCOUPLING_PROTEINS	Down	1.9E-04	1.1E-02
	FORMATION_OF_ATP_BY_CHEMIOSMOTIC_COUPLING	Down	2.1E-04	1.1E-02
	CRISTAE_FORMATION	Down	2.2E-04	1.2E-02
	KERATINIZATION	Down	2.6E-04	1.3E-02
	INTERFERON_SIGNALING	Down	4.5E-04	2.2E-02
	PROSTACYCLIN_SIGNALLING_THROUGH_PROSTACYCLIN_RECEPTOR	Down	4.8E-04	2.2E-02
	THE_CITRIC_ACID_TCA_CYCLE_AND_RESPIRATORY_ELECTRON_TRANSPORT	Down	4.9E-04	2.2E-02
post-LVC ectasia *vs* KTCN *3rd TR*_male	DNA_METHYLATION	Up	1.9E-16	9.3E-13
	ACTIVATED_PKN1_STIMULATES_TRANSCRIPTION_OF_AR_ANDROGEN_RECEPTOR_REGULATED_GENES_KLK2_AND_KLK3	Up	4.2E-16	1.0E-12
	ASSEMBLY_OF_THE_ORC_COMPLEX_AT_THE_ORIGIN_OF_REPLICATION	Up	3.1E-15	5.0E-12
	SIRT1_NEGATIVELY_REGULATES_RRNA_EXPRESSION	Up	6.3E-15	7.7E-12
	MEIOTIC_RECOMBINATION	Up	7.9E-15	7.7E-12
	CONDENSATION_OF_PROPHASE_CHROMOSOMES	Up	2.3E-13	1.9E-10
	PRC2_METHYLATES_HISTONES_AND_DNA	Up	4.4E-13	3.0E-10
	HDACS_DEACETYLATE_HISTONES	Up	1.0E-12	6.2E-10
	HCMV_LATE_EVENTS	Up	5.0E-12	2.7E-09
	EUKARYOTIC_TRANSLATION_ELONGATION	Up	9.1E-12	4.4E-09

Abbreviations: post-LVC ectasia – post-laser vision correction ectasia; KTCN – keratoconus; *1st TR – central topographic region*, *2nd TR – middle topographic region*, *3rd TR – peripheral topographic*; log2FC - log2 transformed fold change.

Table S13. Proteins found to be discriminative in *TRs* of CE in patients with post-LVC ectasia in comparison to corresponding CE *TRs* of control individuals. Analysis was performed in three settings depending on the sex of the individuals included. All m/z values (corresponding to individual protein fragments), fragment sequence, protein name, and p-values of the Mann-Whitney test are presented in Table S14.

	Post-LVC ectasia females&males			Post-LVC ectasia females			Post-LVC ectasia males		
	1stTR	*2ndTR*	*3rdTR*	*1stTR*	*2ndTR*	*3rdTR*	*1stTR*	*2ndTR*	*3rdTR*
UP	ALDH3A1<br>ENO1<br>GAPDH<br>KRT76<br>PPIA†<br>SDC1<br>TBC1D4*	ACTB<br>ENO1<br>GAPDH<br>PPIA†<br>SDC1<br>TBC1D4†	ACTB*<br>ENO1*<br>FAM102B<br>TBC1D4*†	ANK2<br>ENO1<br>GANAB<br>GAPDH<br>PPIA<br>PTPLB<br>SDC1<br>TBC1D4*	ACTB<br>ALDH3A1<br>ENO1<br>GAPDH<br>SDC1<br>TBC1D4	ACTB*<br>ENO1*<br>TBC1D4*†	KRT76<br>TBC1D4*	-	ACTB*†<br>ENO1*†<br>FAM102B<br>FLRT1<br>GAPVD1<br>KRT3†<br>KRT5<br>KRT76<br>MRGBP<br>TBC1D4*
DOWN	5 SV<br>ABCA13*<br>BTN2A2*<br>CIITA<br>CLEC4F<br>CLU<br>CWC25*<br>DOCK8*<br>FAT3<br>FFAR3<br>FGF8<br>GOLGA4*<br>HSPA1A<br>ISL2<br>KRT12*<br>LRRC20<br>LTBP4<br>MLL<br>PPIA†<br>STAB1<br>SUMO2<br>TMEM217<br>TRRAP<br>USP31*<br>WDR62*<br>ZFP106*	ABCA13*<br>BTN2A2<br>CIITA<br>CLEC4F<br>CLU<br>DOCK8<br>FAT3*<br>FFAR3<br>FGF8<br>GOLGA4<br>HSPB1<br>KRT12*<br>KRT3<br>KRT7<br>LTBP4<br>MLL<br>PPIA†<br>STAB1<br>SUMO2<br>TBC1D4†<br>TRRAP<br>USP31<br>WDR62*<br>ZFP106	5 SV<br>ABCA13<br>ANXA2<br>BTN2A2<br>CIITA<br>CLEC4F<br>CLU<br>CWC25<br>DOCK8<br>FAM186B<br>FAT3<br>GAL3ST4<br>GAPVD1<br>GOLGA4<br>HSPB1<br>INSRR<br>ISL2<br>KRT12*<br>KRT7<br>LRRC27<br>NUCB1<br>PPIA<br>SLC25A23<br>SUMO2<br>TBC1D4†<br>TMEM217<br>TSEN54<br>USP31<br>WDR62<br>ZFP106	5 SV<br>ABCA13*<br>BTN2A2*<br>CIITA<br>CLU<br>CWC25*<br>DOCK8*<br>FGF8<br>GOLGA4*<br>ISL2<br>KRT12<br>KRT3<br>LRRC20<br>MLL<br>TSEN54<br>USP31*<br>WDR62*<br>ZFP106*	ABCA13*<br>FAT3*<br>KRT12*<br>KRT5<br>LRRC20<br>MLL<br>PPIA<br>TRRAP<br>WDR62*	FAT3<br>FGF8<br>KRT12*<br>TBC1D4†<br>USP31	ABCA13*<br>ANXA2<br>BTN2A2*<br>C15orf52<br>CWC25*<br>DOCK8*<br>FAT3<br>GOLGA4*<br>KRT12*<br>LTBP4<br>PTPN1<br>RMI1<br>STAB1<br>SUMO2<br>TMEM217<br>USP31*<br>WDR62*<br>ZFP106*	ABCA13*<br>ANXA2<br>BTN2A2<br>C15orf52<br>CIITA<br>CLEC4F<br>FAM186B<br>FAR2<br>FAT3*<br>GAL3ST4<br>GOLGA4<br>HSPB1<br>KRT12*<br>KRT3<br>KRT7<br>LRRC27<br>LTBP4<br>SHROOM2<br>SUMO2<br>TMEM217<br>WDR62*	ABCA13<br>ACTB†<br>ANXA2<br>BTN2A2<br>CIITA<br>CLEC4F<br>CLU<br>DOCK8<br>ENO1†<br>FABP5<br>FAM186B<br>GAL3ST4<br>GAPVD1<br>GOLGA4<br>HSPB1<br>INSRR<br>ISL2<br>KRT12*<br>KRT3†<br>KRT7<br>LTBP4<br>NUCB1<br>PTPLB<br>RMI1<br>SLC25A23<br>SUMO2<br>TMEM217<br>TSEN54<br>ZFP106

Abbreviations and symbols in Table: UP stands for upregulated proteins, DOWN stands for downregulated proteins, post-LVC ectasia – post-laser vision correction ectasia, *TR – topographic region* of corneal epithelium, *1stTR – central topographic region*, *2ndTR – middle topographic region*, *3rdTR – peripheral topographic region.*

* indicates proteins revealed as discriminating for particular *TRs* of CE, irrespectively of sex/in each comparison

† different protein fragments were revealed as discriminating for particular *TRs* of CE as up- and downregulated (see Table S14 for details): for PPIA in *TR1* (females&males), the m/z peak of 1989.983248 was 'down-', but the m/z peak of 1990.973795 was 'up-'; for TBC1D4 in *TR2* (females&males), the m/z peak of 2432.132664 was 'down-', but m/z peaks of 2433.138151, 2434.139292, 2435.141433, and 2436.147519 were 'up-'; for PPIA in *TR2* (females&males), the m/z peak of 11989.983248 was 'down-', but the m/z peak of 1990.973795 was 'up-'; for TBC1D4 in *TR3* (females&males), the m/z peak of 2435.141433 was 'down-', but the m/z peaks of 2432.132664, 2433.138151, and 2434.139292 were 'up-'; for TBC1D4 in *TR3* (females), the m/z peak of 2433.138151 was 'down-', but the m/z peaks of 2432.132664 and 2435.141433 were 'up-'; for ACTB in *TR3* (males), the m/z peaks of 1516.737438 and 1517.730027 were 'down-', but the m/z peaks of 3184.6149 and 3185.612433 were 'up-'; for ENO1 in *TR3* (males), the m/z peaks of 806.4419971 and 807.4377956 were 'down-

‘, but the m/z peak of 2176.06074 was ‘up-’; for KRT3 in *TR3* (males), the m/z peaks of 1263.689918, 1264.692268, and 1265.692258 were ‘down-’, but the m/z peaks of 3056.429785 and 3058.437485 were ‘up-’

Table S14. The details on discriminative proteins in post-LVC ectasia. Identified proteins classified as discriminative for particular *TRs* of the CE of patients with post-LVC ectasia in comparison to corresponding *TRs* of the CE of control individuals. Analysis was performed in three settings depending on the sex of the individuals included and presented together with m/z values, fragment sequence, protein name, p-values of Mann-Whitney test, and direction of change.

			post-LVC ectasia females&males						post-LVC ectasia females						post-LVC ectasia males					
			1st TR		*2nd TR*		*3rd TR*		*1st TR*		*2nd TR*		*3rd TR*		*1st TR*		*2nd TR*		*3rd TR*	
m/z values	Sequence	Protein name	p-value	change	p-value	change	p-value	change	p-value	change	p-value	change	p-value	change	p-value	change	p-value	change	p-value	change
704.3989859	x	x	0.477		0.891		0.848		0.734		0.932		0.074		0.101		0.234		0.014	down
761.391548	x	x	0.536		0.373		0.851		0.028	down	0.048	down	0.57		0.366		0.445		0.181	
766.3869393	x	x	0.427		0.978		0.847		1		0.932		0.428		0.101		0.445		0.035	down
777.3853583	x	x	0.009		0.019	down	0.042	down	0.014	down	0.261	down	0.234	down	0.035	down	0.005	down	0.027	down
795.4004836	x	x	0.298		0.183		0.183		0.395		0.57		0.283		0.181		0.181		0.035	down
805.4287908	R.GVVMLNR.V	Tyrosine-protein phosphatase non-receptor type 1	0.088		0.599		0.802		0.333		0.333		0.928		0.035	down	0.617		0.283	
806.4419971	K.YNQLLR.I	Alpha-enolase	0.536		0.427		0.727		0.933		0.799		0.461		0.073		0.101		0.005	down
807.4377956	K.YNQLLR.I	Alpha-enolase	0.511		0.547		0.584		1		0.496		0.734		0.051		0.295		0.005	down
810.4064156	x	x	0.0001	down	0.03	down	0.344		0.008	down	0.008	down	0.808		0.022	down	0.628		0.181	
821.4095891	R.LAAEDFR.M	Keratin, type I cytoskeletal 12	0.0001	down	0.005	down	0.038	down	0.004	down	0.008	down	0.283		0.022	down	0.295		0.022	down
822.413646	R.LAAEDFR.M	Keratin, type I cytoskeletal 12	0.0001	down	0.001	down	0.015	down	0.004	down	0.016	down	0.154		0.014	down	0.073		0.022	down
823.433282	x	x	0.565		0.137		0.546		0.669		0.025	down	0.604		0.073		1		0.628	
843.4859953	K.LAAMPALR.S	Leucine-rich repeat-containing protein 20	0.029	down	0.311		0.075		0.016	down	0.146		0.173		0.534		0.534		0.138	
844.4803004	K.LAAMPALR.S	Leucine-rich repeat-containing protein 20	0.117		0.162		0.066		0.2		0.05	down	0.073		0.283		0.836		0.224	
865.3901057	K.DGFNNTR.K	Zinc finger protein 106 homolog	0.002	down	0.03	down	0.018	down	0.008	down	0.167		0.154		0.008	down	0.073		0.035	down
884.4333238	x	x	0.035	down	0.015	down	0.107		0.008	down	0.074		0.283		0.534		0.101		0.138	
896.4314275	R.SSSHSPPR.H	Pre-mRNA-splicing factor CWC25 homolog	0.0001	down	0.155		0.013	down	0.008	down	0.173		0.106		0.038	down	0.534		0.074	
915.4823985	M.NVTSIALR.A	RecQ-mediated genome instability protein 1	0.107		0.051		0.311		0.368		0.109		1		0.022	down	0.181		0.008	down
916.4830142	M.NVTSIALR.A	RecQ-mediated genome instability protein 1	0.106		0.179		0.115		0.347		0.799		0.73		0.035	down	0.174		0.012	down
944.5210882	K.RGNIHPPR.D	Galactose-3-	0.234		0.128		0.046	down	0.365		0.333		0.202		0.101		0.038	down	0.073	
945.5224892	K.RGNIHPPR.D	Galactose-3-	0.366		0.106		0.021	down	0.202		0.214		0.283		0.295		0.101		0.008	down
946.5231131	K.RGNIHPPR.D	Galactose-3-	0.338		0.083		0.014	down	0.461		0.347		0.307		0.534		0.038	down	0.008	down
961.4794575	K.NVDTNQDR.L	Nucleobindin-1	0.427		0.458		0.019	down	0.496		0.252		0.538		0.445		0.534		0.008	down
971.4818511	x	x	0.054		0.059		0.721		0.147		0.008	down	1		0.073		0.836		0.445	
973.5207475	K.DKILESIR.Q	ATP-binding cassette sub-family A member 13	0.084		0.074		0.025	down	0.148		0.167		0.283		0.074		0.008	down	0.001	down
975.5197322	K.DKILESIR.Q	ATP-binding cassette sub-family A member 13	0.0001	down	0.0001	down	0.017	down	0.004	down	0.013	down	0.214		0.014	down	0.035	down	0.022	down

976.473973	x	x	0.02 down	0.02 down	0.046 down	0.008 down	0.049 down	0.683	0.138	0.174	0.012 down
1005.46904	x	x	0.003 down	0.007 down	0.1	0.022 down	0.073	0.57	0.035 down	0.038 down	0.101
1007.506637	R.SYDEAILR.L	Butyrophilin subfamily 2 member A2	0.075	0.051	0.007 down	0.214	0.283	0.154	0.366	0.005 down	0.003 down
1008.504567	R.SYDEAILR.L	Butyrophilin subfamily 2 member A2	0.013 down	0.021 down	0.029 down	0.048 down	0.109	0.283	0.022 down	0.014 down	0.001 down
1009.508715	R.SYDEAILR.L	Butyrophilin subfamily 2 member A2	0.026 down	0.026 down	0.01 down	0.148	0.368	0.173	0.008 down	0.027 down	0.003 down
1016.529016	R.REAPYGAPR.F	Latent-transforming growth factor beta-binding protein 4	0.049 down	0.046 down	0.051	0.234	0.214	0.154	0.014 down	0.038 down	0.014 down
1043.540342	M.ATVQQLEGR.W	Fatty acid-binding protein, epidermal	0.636	0.561	0.16	1	0.468	0.545	0.352	0.181	0.038 down
1045.560265	R.VLPDMVSLR.V	MHC class II transactivator	0.051	0.046 down	0.038 down	0.048 down	0.106	0.154	0.138	0.035 down	0.008 down
1046.553857	R.VLPDMVSLR.V	MHC class II transactivator	0.031 down	0.059	0.04 down	0.106	0.106	0.234	0.073	0.051	0.005 down
1059.598382	R.VLDELTLTR.T	Keratin, type I cytoskeletal 12	0.003 down	0.003 down	0.018 down	0.028 down	0.048 down	0.073 down	0.014 down	0.008 down	0.014 down
1060.586979	R.VLDELTLTR.T	Keratin, type I cytoskeletal 12	0.003 down	0.006 down	0.025 down	0.028 down	0.05 down	0.154 down	0.014 down	0.014 down	0.014 down
1061.581164	R.VLDELTLTR.T	Keratin, type I cytoskeletal 12	0.004 down	0.007 down	0.017 down	0.021 down	0.106	0.033 down	0.014 down	0.008 down	0.053
1067.561762	x	x	0.046 down	0.081	0.049 down	0.05 down	0.252	0.142	0.101	0.074	0.052
1075.572561	R.RPHFFFPK.S	Clusterin	0.007 down	0.032 down	0.002 down	0.014 down	0.134	0.109	0.1	0.099	0.005 down
1097.510592	K.DLSKDQHGR.N	Dedicator of cytokinesis protein 8	0.008 down	0.034 down	0.021 down	0.014 down	0.167	0.146	0.005 down	0.073	0.005 down
1104.585083	R.QGLSMRQIR.F	Small ubiquitin-related modifier 2	0.008 down	0.046 down	0.033 down	0.074	0.154	0.109	0.005 down	0.022 down	0.002 down
1108.556254	x	x	0.04 down	0.583	0.011 down	0.173	0.266	0.049 down	0.073	0.945	0.052
1109.55798	x	x	0.309	0.561	0.009 down	0.437	0.468	0.61	0.534	0.445	0.003 down
1111.563379	R.QDIAFAYQR.R	Annexin A2	0.058	0.166	0.038 down	0.154	0.283	0.214	0.022 down	0.138	0.035 down
1112.56154	R.QDIAFAYQR.R	Annexin A2	0.075	0.062	0.025 down	0.395	0.441	0.109	0.014 down	0.022 down	0.035 down
1133.560061	x	x	0.035 down	0.041 down	0.005 down	0.049 down	0.167	0.048 down	0.181	0.074	0.008 down
1139.551285	x	x	0.394	0.701	0.081	0.545	0.669	0.333	0.295	0.181	0.027 down
1141.525265	K.EELDQQNKR.F	Golgin subfamily A member 4	0.004 down	0.023 down	0.009 down	0.008 down	0.074	0.106	0.005 down	0.073	0.005 down
1142.535865	K.EELDQQNKR.F	Golgin subfamily A member 4	0.023 down	0.039 down	0.065	0.033 down	0.342	0.342	0.008 down	0.022 down	0.008 down
1152.574192	K.IREWYETR.G	Keratin, type I cytoskeletal 12	0.529	0.344	0.017 down	0.683	0.461	0.154	1	0.295	0.035 down
1153.579693	K.IREWYETR.G	Keratin, type I cytoskeletal 12	0.427	0.31	0.007 down	0.368	0.437	0.106	0.836	0.073	0.012 down
1154.577639	K.IREWYETR.G	Keratin, type I cytoskeletal 12	0.286	0.198	0.016 down	0.368	0.441	0.146	0.534	0.073	0.008 down
1163.612106	R.LFDQAFGLPR.L	Heat shock protein beta-1	0.956	0.324	0.004 down	1	0.496	0.214	1	0.181	0.005 down
1164.612131	R.LFDQAFGLPR.L	Heat shock protein beta-1	0.956	0.46	0.01 down	0.933	0.932	0.395	0.731	0.138	0.001 down
1165.606688	R.LFDQAFGLPR.L	Heat shock protein beta-1	0.373	0.12	0.002 down	0.683	0.283	0.073	0.445	0.022 down	0.005 down
1166.60228	R.LFDQAFGLPR.L	Heat shock protein beta-1	0.125	0.018 down	0.003 down	0.214	0.154	0.106	0.366	0.008 down	0.005 down
1171.605614	K.APGSKGSCPLR.K	C-type lectin domain family 4 member F	0.033 down	0.03 down	0.01 down	0.073	0.104	0.084	0.181	0.008 down	0.008 down
1172.594636	K.APGSKGSCPLR.K	C-type lectin domain family 4 member F	0.066	0.244	0.085	0.073	0.368	0.461	0.295	0.101	0.035 down
1173.601438	x	x	0.134	0.317	0.075	0.283	0.283	0.347	0.181	0.295	0.005 down
1194.582589	x	x	0.008 down	0.051	0.033 down	0.048 down	0.109	0.395	0.008 down	0.101	0.005 down
1195.585252	x	x	0.013 down	0.085	0.033 down	0.109	0.148	0.368	0.014 down	0.101	0.008 down

1196.586373	x	x	0.045 down	0.105	0.039 down	0.441	0.167	0.2	0.038 down	0.138	0.018 down
1221.639817	x	x	0.03 down	0.006 down	0.08	0.195	0.057	0.333	0.014 down	0.027 down	0.037 down
1263.689918	K.LALDVEIATYR.K	Keratin, type II cytoskeletal 3	0.183	0.12	0.051	0.683	0.683	0.368	0.051	0.022 down	0.035 down
1264.692268	K.LALDVEIATYR.K	Keratin, type II cytoskeletal 3	0.202	0.085	0.051	0.808	0.368	0.283	0.051	0.035 down	0.035 down
1265.692258	K.LALDVEIATYR.K	Keratin, type II cytoskeletal 3	0.183	0.044 down	0.051	0.461 down	0.154	0.368	0.181	0.035 down	0.035 down
1266.653945	R.AADVEPSSPKPK.R	Putative NPIP-like protein L	0.033 down	0.166	0.058	0.016 down	0.214	0.109	0.366	0.945	0.295
1267.652091	R.AADVEPSSPKPK.R	Putative NPIP-like protein L	0.018 down	0.134	0.044 down	0.016 down	0.109	0.073	0.181	0.945	0.234
1268.649403	R.AADVEPSSPKPK.R	Putative NPIP-like protein L	0.052	0.166	0.084	0.033 down	0.073	0.089	0.366	0.945	0.445
1274.578974	R.QLLPGDEFSLR.E	Insulin gene enhancer protein ISL-2	0.013 down	0.075	0.021 down	0.048 down	0.154	0.214	0.073	0.138	0.014 down
1275.581293	R.QLLPGDEFSLR.E	Insulin gene enhancer protein ISL-2	0.018 down	0.052	0.025 down	0.048 down	0.104	0.173	0.073	0.138	0.014 down
1276.580218	R.QLLPGDEFSLR.E	Insulin gene enhancer protein ISL-2	0.013 down	0.073	0.009 down	0.049 down	0.252	0.073	0.073	0.073	0.018 down
1310.666466	K.EMAWKVNMYR.G	Transformation/transcription domain-associated protein	0.039 down	0.034 down	0.154	0.104	0.049 down	0.2	0.074	0.283	0.445
1321.694627	K.NRSQGNIISYK.R	Transmembrane protein 217	0.027 down	0.057	0.131	0.084	0.134	0.266	0.027 down	0.138	0.352
1322.655187	K.NRSQGNIISYK.R	Transmembrane protein 217	0.095	0.119	0.018 down	0.173	0.202	0.154	0.234	0.022 down	0.014 down
1323.650852	K.NRSQGNIISYK.R	Transmembrane protein 217	0.125	0.089	0.013 down	0.173	0.2	0.12	0.352	0.014 down	0.018 down
1327.704029	K.TPSKDSLDPDPR.C	WD repeat-containing protein 62	0.008 down	0.013 down	0.055	0.074	0.049 down	0.173	0.014 down	0.073	0.101
1328.696177	K.TPSKDSLDPDPR.C	WD repeat-containing protein 62	0.007 down	0.028 down	0.066	0.073	0.146	0.214	0.027 down	0.073	0.138
1329.691889	K.TPSKDSLDPDPR.C	WD repeat-containing protein 62	0.008 down	0.011 down	0.045 down	0.049 down	0.05 down	0.106	0.074	0.038 down	0.074
1345.630926	R.DQLRGHPDIPR.L	Protein FAM186B	0.198	0.051	0.021 down	0.202	0.226	0.234	0.051	0.014 down	0.005 down
1346.630597	R.DQLRGHPDIPR.L	Protein FAM186B	0.239	0.054	0.015 down	0.395	0.226	0.167	0.138	0.018 down	0.008 down
1355.775775	x	x	0.03 down	0.158	0.492	0.073	0.167	0.342	0.035 down	0.429	0.836
1392.749705	R.LVWILGRGGSHR.R	Free fatty acid receptor 3	0.033 down	0.038 down	0.095	0.109	0.214	0.214	0.138	0.051	0.234
1393.740482	K.YLLKEDMAGIPK.A	Fatty acyl-CoA reductase 2	0.12	0.095	0.051	0.214	0.461	0.154	0.295	0.035 down	0.138
1410.692159	R.RVTALVPSEAAVR.Q	Stabilin-1	0.044 down	0.018 down	0.095	0.214	0.109	0.214	0.035 down	0.051	0.138
1411.695885	R.RVTALVPSEAAVR.Q	Stabilin-1	0.033 down	0.04 down	0.095	0.109	0.148	0.283	0.051	0.101	0.138
1412.694588	R.RVTALVPSEAAVR.Q	Stabilin-1	0.093	0.023 down	0.062	0.252	0.106	0.084	0.051	0.073	0.138
1418.750943	K.VDALNDEINFLR.T	Keratin, type II cytoskeletal 7	0.244	0.011 down	0.12	0.461	0.154	0.368	0.051	0.008 down	0.181
1419.752533	K.VDALNDEINFLR.T	Keratin, type II cytoskeletal 7	0.317	0.018 down	0.095	0.683	0.283	0.368	0.051	0.008 down	0.138
1420.751756	K.VDALNDEINFLR.T	Keratin, type II cytoskeletal 7	0.222	0.013 down	0.051	0.368	0.283	0.154	0.051	0.008 down	0.101
1421.737393	K.VDALNDEINFLR.T	Keratin, type II cytoskeletal 7	0.262	0.066	0.048 down	0.269	0.214	0.496	0.073	0.008 down	0.027 down
1450.740819	R.QFIMPVVSAISSR.I	Histone-lysine N-methyltransferase MLL	0.015 down	0.149	0.149	0.048 down	0.154	0.283	0.051	0.445	0.295
1451.735498	R.QFIMPVVSAISSR.I	Histone-lysine N-methyltransferase MLL	0.013 down	0.066	0.107	0.074	0.048 down	0.283	0.051	0.366	0.138
1452.73821	R.QFIMPVVSAISSR.I	Histone-lysine N-methyltransferase MLL	0.018 down	0.034 down	0.058	0.084	0.049 down	0.2	0.051	0.283	0.133
1469.726911	R.NQGIEGSPGGRVTR.S	Uncharacterized protein C15orf52	0.249	0.257	0.656	0.73	0.93	0.468	0.035 down	0.018 down	0.52
1472.715506	K.SSQLDSGVPSSPGGR.Q	Ubiquitin carboxyl-terminal hydrolase 31	0.012 down	0.029 down	0.023 down	0.038 down	0.104	0.033 down	0.014 down	0.131	0.073

1516.737438	K.IWHHTFYNELR.V	Actin, cytoplasmic 1	0.647	0.851	0.12	0.808	0.808	0.808	0.945	0.234	0.022 down
1517.730027	K.IWHHTFYNELR.V	Actin, cytoplasmic 1	0.979	0.565	0.12	1	0.549	0.683	1	0.138	0.022 down
1540.778881	K.VVIGMDVAASEFFR.S	Alpha-enolase	0.033 up	0.044 up	0.267 up	0.028 up	0.016 up	0.57	0.628	0.836	0.366
1547.742935	M.EPEPEPAAVEVPAGR.V	tRNA-splicing endonuclease subunit Sen54	0.681	0.363	0.043 down	0.214 down	0.428	0.342	0.731	0.234	0.012 down
1548.794825	R.AGREPQGVSQQHVR.E	Fibroblast growth factor 8	0.046 down	0.1 down	0.066	0.033 down	0.104	0.033 down	0.138	0.534	0.534
1549.800607	R.AGREPQGVSQQHVR.E	Fibroblast growth factor 8	0.055	0.049 down	0.188	0.057	0.084	0.073	0.138	0.281	0.945
1557.775442	x	x	0.956	0.28	0.178	0.73	0.587	0.437	0.234	0.035 down	0.073
1558.773424	x	x	0.803	0.333	0.295	0.428	0.587	0.792	0.133	0.035 down	0.073
1578.761179	x	x	0.758	0.846	0.104	0.479	0.65	0.666	0.366	0.1	0.008 down
1620.816992	x	x	0.132	0.477	0.066	0.347	1	0.683	0.295	0.366	0.005 down
1646.809377	-.MAAVAATAAAKGNGGGGGR.A	3-hydroxyacyl-CoA dehydratase 2	0.213	0.335	0.161	0.028 up	0.187	0.795	0.617	0.731	0.005 down
1701.825419	x	x	0.641	0.238	0.039 down	1	0.798	0.347	0.445	0.051	0.038 down
1702.834224	x	x	0.603	0.238	0.046 down	0.865	0.437	0.368	0.534	0.051	0.038 down
1763.82384	K.LISWYDNEFGYSNR.V	Glyceraldehyde-3-phosphate dehydrogenase	0.006 up	0.052	0.979	0.02 up	0.074	0.461	0.295	0.628	0.445
1764.81506	K.LISWYDNEFGYSNR.V	Glyceraldehyde-3-phosphate dehydrogenase	0.003 up	0.027 up	0.529	0.02 up	0.014 up	0.269	0.138	0.628	1
1765.817217	K.EGEAVVLPEVEPGLTAR.E	Syndecan-1	0.023 up	0.018 up	0.681	0.049 up	0.006 up	0.444	0.366	0.534	0.836
1795.844542	x	x	0.622	0.565	0.014 down	1	0.307	0.195	0.731	0.731	0.074
1796.852034	x	x	0.528	0.721	0.043 down	0.552	0.669	0.261	0.943	0.731	0.099
1859.901	x	x	0.008 up	0.142	0.023 up	0.02 up	0.195	0.049 up	0.295	0.518	0.628
1860.904692	x	x	0.003 up	0.005 up	0.014 up	0.021 up	0.022 up	0.104	0.181	0.153	0.101
1861.916282	x	x	0.05 up	0.14	0.003 up	0.187	0.468	0.02 up	0.295	0.317	0.035 down
1862.962705	x	x	0.202	0.603	0.134	1	0.865	0.016 down	0.022 up	0.234	0.366
1863.96948	x	x	0.311	0.681	0.025 down	1	0.933	0.016 down	0.073	0.617	1
1864.92974	x	x	0.188	0.978	0.066	0.671	1	0.05 down	0.153	1	0.836
1873.947553	R.REPWLLPSQHNDIIR.D	Neutral alpha-glucosidase AB	0.244	0.809	0.572	0.016 up	0.933	0.57	1	0.731	0.836
1874.948694	R.REPWLLPSQHNDIIR.D	Neutral alpha-glucosidase AB	0.183	0.727	0.687	0.028 up	0.933	0.683	0.731	0.836	0.731
1875.946715	R.REPWLLPSQHNDIIR.D	Neutral alpha-glucosidase AB	0.183	0.936	0.403	0.016 up	1	0.808	0.945	0.731	0.945
1876.938663	R.REPWLLPSQHNDIIR.D	Neutral alpha-glucosidase AB	0.052	0.493	0.298	0.016 up	0.734	0.808	0.534	0.534	0.628
1891.969282	R.LGGNSSDFEIQEDKVPR.E	Insulin receptor-related protein	0.267	0.609	0.044 down	0.154	0.933	0.683	0.628	0.295	0.051
1892.968198	R.LGGNSSDFEIQEDKVPR.E	Insulin receptor-related protein	0.291	0.536	0.038 down	0.073	0.933	0.57	0.836	0.295	0.051
1893.964563	R.LGGNSSDFEIQEDKVPR.E	Insulin receptor-related protein	0.46	0.501	0.046 down	0.154	0.933	0.61	1	0.234	0.035 down
1902.909032	x	x	0.012 down	0.026 down	0.107	0.2	0.368	0.048 down	0.008 down	0.008 down	0.731
1903.909237	x	x	0.072	0.027 down	0.262	0.277	0.266	0.074	0.035 down	0.037 down	0.836
1909.96725	K.QDLIKMTAILTTDVSDK.A	Ankyrin-2	0.134	0.893	0.12	0.016 up	0.808	0.683	0.534	0.366	0.101
1919.905447	x	x	0.035 up	0.741	0.68	0.146	0.66	0.545	0.234	0.366	0.945
1988.006537	x	x	0.003 down	0.021 down	0.033 down	0.033 up	0.048 down	0.073	0.101	0.366	0.138
1989.006468	x	x	0.001 down	0.025 down	0.033 down	0.008 up	0.073	0.214	0.051	0.445	0.035 down

1989.983248	M.VNPTVFFDIAVDGEPLGR.V	Peptidyl-prolyl cis-trans isomerase A	0.002 down	0.005 down	0.029 down	0.022 up	0.016 down	0.073	0.138	0.138	0.051
1990.973795	M.VNPTVFFDIAVDGEPLGR.V	Peptidyl-prolyl cis-trans isomerase A	0.003 up	0.033 up	0.095	0.014 up	0.109	0.214	0.181	0.234	0.366
2010.098693	K.QLVAGAVAGAVSRTGTAPLDR.L	Calcium-binding mitochondrial carrier protein SCaMC-3	0.848	0.179	0.02 down	0.683	0.261	0.669	0.943	0.138	0.005 down
2011.10915	K.QLVAGAVAGAVSRTGTAPLDR.L	Calcium-binding mitochondrial carrier protein SCaMC-3	0.603	0.285	0.009 down	0.799	0.342	0.347	0.836	0.138	0.005 down
2012.10652	K.QLVAGAVAGAVSRTGTAPLDR.L	Calcium-binding mitochondrial carrier protein SCaMC-3	0.848	0.142	0.011 down	0.683	0.187	0.347	0.83	0.133	0.005 down
2014.083031	x	x	0.311	0.125	0.027 down	0.395	0.173	0.368	0.534	0.224	0.014 down
2030.029306	R.LLQQLAMTGSEEGDPRTK.S	GTPase-activating protein and VPS9 domain-containing protein 1	0.477	0.809	0.12	0.234	0.808	0.933	0.731	0.731	0.008 up
2031.030984	R.LLQQLAMTGSEEGDPRTK.S	GTPase-activating protein and VPS9 domain-containing protein 1	0.642	0.767	0.038 down	0.444	1	1	0.945	0.628	0.002 up
2033.050567	R.LLQQLAMTGSEEGDPRTK.S	GTPase-activating protein and VPS9 domain-containing protein 1	0.344	0.183	0.015 down	0.461	0.808	0.368	0.366	0.051	0.022 down
2034.043454	R.LLQQLAMTGSEEGDPRTK.S	GTPase-activating protein and VPS9 domain-containing protein 1	0.338	0.059	0.033 down	0.496	0.368	0.283	0.295	0.052	0.051
2047.053947	R.QNLEPLFEQYINNLRR.Q	Keratin, type II cytoskeletal 5	0.344	0.434	0.149	0.683	0.214	1	0.445	0.836	0.014 up
2048.062733	R.QNLEPLFEQYINNLRR.Q	Keratin, type II cytoskeletal 5	0.727	0.166	0.501	0.808	0.048 down	0.57	0.945	0.445	0.035 up
2055.044382	K.EKASFLPPVEKPDLSELR.K	Leucine-rich repeat-containing protein 27	0.145	0.078	0.032 down	0.669	0.428	0.146	0.053	0.008 down	0.101
2105.104326	R.LIQEQEQELVGALAADLHK.N	Aldehyde dehydrogenase, dimeric NADP-preferring	0.04 up	0.115	0.934	0.148	0.07	0.66	0.181	1	0.668
2106.105774	R.LIQEQEQELVGALAADLHK.N	Aldehyde dehydrogenase, dimeric NADP-preferring	0.095	0.054	0.978	0.187	0.006 up	0.932	0.281	1	0.731
2176.06074	K.AGYTDKVVIGMDVAASEFFR.S	Alpha-enolase	0.805	1	0.032 up	0.252	0.808	1	0.731	0.617	0.003 up
2264.105079	x	x	0.04 down	0.394	0.68	0.552	0.549	0.798	0.101	0.721	0.174
2265.108064	x	x	0.007 up	0.366	0.935	0.142	0.671	0.61	0.035 up	0.628	1
2266.106452	x	x	0.014 up	0.023 up	0.239	0.106	0.021 up	0.368	0.131	0.317	0.668
2296.1347	x	x	0.008 up	0.161	0.16	0.109	0.444	0.552	0.035 up	0.222	0.128
2297.138205	x	x	0.034 up	0.891	0.207	0.2	0.57	0.808	0.037 up	0.567	0.026 up
2298.139694	x	x	0.129	0.219	0.113	0.437	0.863	0.669	0.103	0.244	0.036 up
2306.144385	R.TLQSNGIIMYTRANPCIILK.I	Protocadherin Fat 3	0.058	0.001 down	0.025 down	0.683	0.016 down	0.154	0.101	0.022 down	0.138
2307.145093	R.TLQSNGIIMYTRANPCIILK.I	Protocadherin Fat 3	0.051	0.0001 down	0.029 down	0.808	0.016 down	0.154 down	0.101	0.022 down	0.138
2308.144862	R.TLQSNGIIMYTRANPCIILK.I	Protocadherin Fat 3	0.051	0.0001 down	0.029 down	0.808	0.016 down	0.154 down	0.101	0.022 down	0.138

2309.144491	R.TLQSNGIIMYTRANPCI ILK.I	Protocadherin Fat 3	0.058		0.0001	down	0.018	down	0.808		0.028	down	0.154	down	0.073		0.022	down	0.138	
2310.144162	R.TLQSNGIIMYTRANPCI ILK.I	Protocadherin Fat 3	0.018	down	0.0001	down	0.008	down	0.368		0.048	down	0.154	down	0.035	down	0.005	down	0.101	
2311.146427	R.TLQSNGIIMYTRANPCI ILK.I	Protocadherin Fat 3	0.207		0.002	down	0.024	down	0.734		0.05	down	0.266	down	0.038	down	0.005	down	0.073	
2354.14968	x	x	0.019	up	0.11		0.243		0.252		0.147		0.441		0.053		0.615		0.941	
2375.179976	K.APNPPTFSELSHCRGAPE LPR.E	Protein Shroom2	0.565		0.131		0.286		1		0.549		0.269		0.295		0.003	down	1	
2376.160903	K.APNPPTFSELSHCRGAPE LPR.E	Protein Shroom2	0.891		0.336		0.848		0.437		0.604		0.669		0.445		0.001	down	0.295	
2380.168494	x	x	0.155		0.04	down	0.049	down	0.283		0.148		0.283		0.181		0.073		0.101	
2381.175688	x	x	0.147		0.021	down	0.023	down	0.307		0.2		0.073		0.181		0.052		0.1	
2392.169511	x	x	0.158		0.074		0.007	up	0.526		0.3		0.552		0.534		0.295		0.001	up
2393.201159	x	x	0.029	up	0.056		0.098		0.347		0.195		0.669		0.026	up	0.131		0.051	
2394.193067	x	x	0.03	up	0.265		0.226		0.365		0.666		0.792		0.035	up	0.313		0.103	
2403.23436	K.LYLQDNAISHIPYNTLAK MR.E	Leucine-rich repeat transmembrane protein FLRT1	0.848		0.848		0.084		0.261		0.671		0.865		0.628		0.945		0.005	up
2404.25209	K.LYLQDNAISHIPYNTLAK MR.E	Leucine-rich repeat transmembrane protein FLRT1	0.639		0.763		0.125		0.195		0.932		0.683		0.721		0.731		0.005	up
2432.132664	R.KQYRPDMMSLQIQMYQL SR.L	TBC1 domain family member 4	0.002	up	0.037	down	0.002	up	0.073		0.109		0.016	up	0.035	up	0.366		0.032	up
2433.138151	R.KQYRPDMMSLQIQMYQL SR.L	TBC1 domain family member 4	0.002	up	0.021	up	0.004	up	0.106		0.109		0.008	down	0.022	up	0.234		0.101	
2434.139292	R.KQYRPDMMSLQIQMYQL SR.L	TBC1 domain family member 4	0.003	up	0.025	up	0.018	up	0.05	up	0.109		0.109		0.035	up	0.181		0.101	
2435.141433	R.KQYRPDMMSLQIQMYQL SR.L	TBC1 domain family member 4	0.002	up	0.023	up	0.015	down	0.049	up	0.05	up	0.016	up	0.008	up	0.181		0.234	
2436.147519	R.KQYRPDMMSLQIQMYQL SR.L	TBC1 domain family member 4	0.0001	up	0.038	up	0.081		0.007	up	0.057		0.094		0.008	up	0.445		0.293	
2448.217379	x	x	0.02	up	0.869		0.395		0.02	up	0.792		0.933		0.445		0.534		0.429	
2530.200277	K.EDVDPHNGADDVFSSSG SL GKASEK.S	MRG-binding protein	0.193		0.51		0.321		0.298		0.444		0.666		0.234		1		0.045	up
2541.33711	R.QDNSILKVLISMQLMSGD P CFK.T	Protein FAM102B	0.355		0.455		0.032	up	0.195		0.395		0.792		0.826		1		0.011	up
2542.315803	R.QDNSILKVLISMQLMSGD P CFK.T	Protein FAM102B	0.611		0.801		0.219		0.538		0.489		0.795		0.826		0.826		0.036	up
2570.087675	x	x	0.218		0.979		0.195		0.444		0.808		0.932		0.534		0.731		0.026	up
2588.064237	X	x	0.373		0.021	down	0.297		0.808		0.126		0.552		0.295		0.138		0.474	
2589.073853	X	x	0.295		0.014	down	1		0.333		0.146		0.792		0.445		0.038	down	0.942	
2769.335539	X	x	0.051		0.034	up	0.045	up	0.266		0.266		0.669		0.091		0.047	up	nd	up

2770.33891	x	x	0.058		0.062		0.041	up	0.368		0.444		0.461		0.052		0.086		0.011	up
2771.336868	x	x	0.009	up	0.052		0.026	up	0.148		0.202		0.214		0.025	up	0.138		nd	up
2772.332818	x	x	0.003	up	0.009	up	0.036	up	0.069		0.069		0.269		0.037	up	0.052		nd	up
2773.33583	x	x	0.001	up	0.004	up	0.075		0.028	up	0.037	up	0.333		0.017	up	nd		nd	up
2774.329917	x	x	0.015	up	0.198		0.366		0.106		0.269		0.808		0.074		0.445		0.131	
2786.358962	K.QTQIFTTYSDNQPGVLIQ VY EGER.A	Heat shock 70 kDa protein 1A/1B	0.044	down	0.584		0.702		0.109		1		0.461		0.101		0.628		0.567	
2798.338657	x	x	0.077		0.129		0.032	up	0.269		0.66		0.214		0.074		0.084		0.023	up
2799.340626	x	x	0.115		0.228		0.039	up	0.106		0.66		0.269		0.27		0.169		0.011	up
2800.348426	x	x	0.123		0.188		0.018	up	0.104		0.604		0.2		0.367		0.123		nd	up
2801.351193	x	x	0.141		0.118		0.025	up	0.266		0.333		0.261		0.313		0.103		nd	up
2802.350883	x	x	0.157		0.165		0.048	up	0.437		0.538		0.545			up	0.103		nd	up
2830.345107	x	x	0.656		0.385		0.468		1		0.441		0.93		0.346		0.016	up	0.074	
2831.332201	x	x	0.3		0.123		0.246		0.669		0.932		1		0.202		0.024	up	0.016	up
2996.420953	x	x	0.012	up	0.152		0.075		0.049	up	0.444		0.368		0.051		0.192		nd	up
2997.43113	x	x	0.008	up	0.285		0.039	up	0.033	up	0.552		0.154		0.052		0.445		0.02	up
2998.442617	x	x	0.02	up	0.066		0.161		0.074		0.148		0.214		0.073		0.534		0.128	
3012.551991	R.HIADLAGNSEVILPVPAF NVINGGSHAGNK.L	Alpha-enolase	0.035	up	0.088		0.17		0.073		0.147		0.261		0.445		0.628		0.607	
3013.549824	R.HIADLAGNSEVILPVPAF NVINGGSHAGNK.L	Alpha-enolase	0.059		0.083		0.07		0.148		0.064		0.047	up	0.366		0.534		0.452	
3054.420508	x	x	0.261		0.935		0.239		0.799		0.552		0.808		0.283		0.445		0.035	up
3056.429785	R.TLYDAELSQMQSHISDT SVVLSMDNNR.S	Keratin, type II cytoskeletal 3	0.434		0.936		0.163		0.57		0.57		0.933		0.628		0.366		0.022	up
3058.437485	R.TLYDAELSQMQSHISDT SVVLSMDNNR.S	Keratin, type II cytoskeletal 3	0.602		0.805		0.162		0.932		0.552		0.933		0.352		0.366		0.012	up
3102.399455	R.TLYEMELSQMQSHASDT SVVLSMDNNR.C	Keratin, type II cytoskeletal 2 oral	0.088		0.284		0.128		0.269		0.808		0.808		0.147		0.111		0.011	up
3103.398308	R.TLYEMELSQMQSHASDT SVVLSMDNNR.C	Keratin, type II cytoskeletal 2 oral	0.035	up	0.147		0.139		0.283		0.808		0.683		0.053		0.053		0.03	up
3104.406133	R.TLYEMELSQMQSHASDT SVVLSMDNNR.C	Keratin, type II cytoskeletal 2 oral	0.034	up	0.218		0.098		0.269		0.933		0.683		0.037	up	0.1		0.011	up
3105.405245	R.TLYEMELSQMQSHASDT SVVLSMDNNR.C	Keratin, type II cytoskeletal 2 oral	0.066		0.197		0.098		0.349		0.799		0.57		0.222		0.099		0.011	up
3184.6149	R.TTGIVMDSGDGVTHTVPI YEGYALPHAILR.L	Actin, cytoplasmic 1	0.077		0.075		0.045	up	0.142		0.142		0.368		0.419		0.385		nd	
3185.612433	R.TTGIVMDSGDGVTHTVPI YEGYALPHAILR.L	Actin, cytoplasmic 1	0.068		0.023	up	0.041	up	0.1		0.047	up	0.202	up	0.419		0.385		nd	

x- stands for peaks unidentified to peptides/proteins
Abbreviations: post-LVC ectasia – post-laser vision correction ectasia; *1st TR – central topographic region*, *2nd TR – middle topographic region*, *3rd TR – peripheral topographic*; log2FC - log2 transformed fold change.

Table S15. The discriminative proteins between post-LVC ectasia and KTCN.

Identified proteins classified as discriminative for particular *TRs* of the CE of patients with post-LVC ectasia,in comparison to corresponding *TRs* of the CE of patients with KTCN. Enrichment analysis was performed in two settings depending on the sex of the individuals included and presented together with m/z values, fragment sequence, protein name, p-values of Mann-Whitney test, and direction of change.

			post-LVC ectasia females&males						post-LVC ectasia males					
			1st TR		*2nd TR*		*3rd TR*		*1st TR*		*2nd TR*		*3rd TR*	
m/z values	Sequence	Protein name	p-value	change	p-value	change	p-value	change	p-value	change	p-value	change	p-value	change
821.4095891	R.LAAEDFR.M	keratin, type i cytoskeletal 12	0.072		0.05	down	0.678		0.59		0.476		0.62	
822.413646	R.LAAEDFR.M	keratin, type i cytoskeletal 12	0.029	down	0.042	down	0.175		0.232		0.288		0.947	
843.4859953	K.LAAMPALR.S	leucine-rich repeat-containing protein 20	0.242		0.606		0.032	down	0.982		0.074		0.234	
865.3901057	K.DGFNNTR.K	zinc finger protein 106 homolog	0.03	down	0.173		0.19		0.301		0.659		0.965	
961.4794575	K.NVDTNQDR.L	nucleobindin-1	0.888		0.509		0.151		0.506		0.04	up	0.411	
975.5197322	K.DKILESIR.Q	ATP-binding cassette sub-family A member 13	0.122		0.027	down	0.522		0.775		0.201		0.612	
987.5730292		PMS2L_HUMAN	0.422		0.19		0.65		0.039	up	0.492		0.117	
1097.510592	K.DLSKDQHGR.N	Dedicator of cytokinesis protein 8	0.034	down	0.226		0.098		0.355		0.74		0.791	
1540.778881	K.VVIGMDVAASEFFR.S	Alpha-enolase	0.063		0.109		0.45		0.156		0.043	up	0.249	
1549.800607	R.AGREPQGVSQQHVR.E	Fibroblast growth factor 8	0.28		0.025	down	0.9		0.808		0.2		0.251	
1598.743449	R.AHGSVFYQIMTMR.R	Nephrocystin-1	0.272		0.612		0.564		0.03	up	0.199		0.165	
1651.800455	R.SLLFGVYLDSNEPR.L		0.14		0.346		0.72		0.024	up	0.352		0.415	
1763.82384	K.LISWYDNEFGYSNR.V	Glyceraldehyde-3-phosphate dehydrogenase	0.102		0.044	up	0.564		0.308		0.026	up	0.809	
1764.81506	K.LISWYDNEFGYSNR.V	Glyceraldehyde-3-phosphate dehydrogenase	0.067		0.03	up	0.864		0.199		0.029	up	0.612	
1765.817217	K.EGEAVVLPEVEPGLTAR.E	Syndecan-1	0.055		0.022	up	0.815		0.165		0.04	up	0.912	
1795.844542	x	x	0.488		0.419		0.05	down	0.424		0.158		0.207	
1860.904692	x	x	0.095		0.01	up	0.057		0.415		0.159		0.185	
1861.916282	x	x	0.65		0.628		0.03	up	0.612		0.343		0.278	
1875.946715	R.REPWLLPSQHNDIIR.D	Neutral alpha-glucosidase AB	0.301		0.041	up	0.673		0.949		0.045	up	0.843	
1903.909237	x	x	0.407		0.044	up	0.988		0.843		0.051		0.301	
2030.029306	R.LLQQLAMTGSEEGDPRTK.S	GTPase-activating protein and VPS9 domain-containing protein 1	0.607		0.296		0.017	up	0.651		0.14		0.023	up
2031.030984	R.LLQQLAMTGSEEGDPRTK.S	GTPase-activating protein and VPS9 domain-containing protein 1	0.696		0.444		0.022	up	0.813		0.194		0.023	up
2047.053947	R.QNLEPLFEQYINNLRR.Q	Keratin, type II cytoskeletal 5	0.414		0.633		0.017	up	0.352		0.949		0.055	
2048.062733	R.QNLEPLFEQYINNLRR.Q	Keratin, type II cytoskeletal 5	0.414		0.607		0.011	up	0.476		0.912		0.023	up
2049.065553	R.QNLEPLFEQYINNLRR.Q	Keratin, type II cytoskeletal 5	0.612		0.522		0.032	up	0.503		0.947		0.05	up

2265.108064	x	x	0.008	up	0.137		0.731		0.021	up	0.085		0.676	
2266.106452	x	x	0.018	up	0.033	up	0.399		0.081		0.067		0.377	
2331.054414	x	x	0.195		0.031	up	0.164		1		0.214		0.982	
2392.169511	x	x	0.294		0.216		0.027	up	0.139		0.28		0.025	up
2403.23436	K.LYLQDNAISHIPYNTLAK MR.E	Leucine-rich repeat transmembrane protein FLRT1	0.938		0.473		0.013	up	0.367		0.311		0.008	up
2404.25209	K.LYLQDNAISHIPYNTLAK MR.E	Leucine-rich repeat transmembrane protein FLRT1	0.65		0.673		0.013	up	0.612		0.612		0.01	up
2432.132664	R.KQYRPDMMSLQIQMYQ LSR.L	TBC1 domain family member 4	0.016	up	0.026	up	0.134		0.055		0.029		0.454	
2433.138151	R.KQYRPDMMSLQIQMYQ LSR.L	TBC1 domain family member 4	0.013	up	0.009	up	0.206		0.03	up	0.014	up	0.613	
2434.139292	R.KQYRPDMMSLQIQMYQ LSR.L	TBC1 domain family member 4	0.006	up	0.01	up	0.31		0.016	up	0.018	up	0.708	
2435.141433	R.KQYRPDMMSLQIQMYQ LSR.L	TBC1 domain family member 4	0.008	up	0.006	up	0.325		0.011	up	0.016	up	0.741	
2436.147519	R.KQYRPDMMSLQIQMYQ LSR.L	TBC1 domain family member 4	<0.001	up	0.013	up	0.397		0.006	up	0.102		0.803	
2530.200277	K.EDVDPHNGADDVFSSSG SLGKASEK.S	MRG-binding protein	0.561		0.463		0.212		0.643		0.552		0.048	up
2541.33711	R.QDNSILKVLISMQLMSGD PCFK.T	Protein FAM102B	1		0.246		0.036	up	0.563		0.193		0.055	
2587.070371	x	x	0.551		0.628		0.017	up	0.439		0.676		0.044	up
2798.338657	x	x	0.512		0.259		0.036	up	0.946		0.577		0.253	
2799.340626	x	x	0.399		0.285		0.043	up	0.91		0.64		0.241	
2800.348426	x	x	0.544		0.277		0.02	up	0.874		0.636		0.128	
2801.351193	x	x	0.605		0.183		0.015	up	0.783		0.66		0.117	
2996.420953	x	x	0.237		0.048	up	0.316		0.517		0.166		0.768	
3012.551991	R.HIADLAGNSEVILPVPAF NVINGGSHAGNK.L	Alpha-enolase	0.696		0.103		0.21		0.708		0.036	up	0.463	
3013.549824	R.HIADLAGNSEVILPVPAF NVINGGSHAGNK.L	Alpha-enolase	0.408		0.059		0.209		0.612		0.014	up	0.666	
3102.399455	R.TLYEMELSQMQSHASDT SVVLSMDNNR.C	Keratin, type II cytoskeletal 2 oral	0.151		0.139		0.037	up	0.548		0.264		0.234	
3103.398308	R.TLYEMELSQMQSHASDT SVVLSMDNNR.C	Keratin, type II cytoskeletal 2 oral	0.077		0.044	up	0.021	up	0.44		0.107		0.15	
3104.406133	R.TLYEMELSQMQSHASDT SVVLSMDNNR.C	Keratin, type II cytoskeletal 2 oral	0.072		0.089		0.027	up	0.32		0.165		0.189	
3105.405245	R.TLYEMELSQMQSHASDT SVVLSMDNNR.C	Keratin, type II cytoskeletal 2 oral	0.099		0.148		0.034	UP	0.364		0.246		0.292	
3184.6149	R.TTGIVMDSGDGVTHTVPI YEGYALPHAILR.L	Actin, cytoplasmic 1	0.346		0.032	UP	0.058		0.946		0.06		0.266	
3185.612433	R.TTGIVMDSGDGVTHTVPI YEGYALPHAILR.L	Actin, cytoplasmic 1	0.141		0.011	UP	0.05	UP	0.982		0.035	UP	0.266	

x- stands for peaks unidentified to peptides/proteins

Abbreviations: post-LVC ectasia – post-laser vision correction ectasia; KTCN – keratoconus; *1^{st}TR – central topographic region*, *2^{nd}TR – middle topographic region*, *3^{rd}TR – peripheral topographic*; log2FC - log2 transformed fold change.

Table S16. The discriminative proteins in multigroup analysis.

Identified proteins classified as discriminative for particular *TRs* of CE in multigroup analysis embracing patients with post-LVC ectasia, patients with KTCN, and controls. The m/z values, fragment sequences, protein names, p-values of the Kruskal-Wallis test, and p-values of Dunn's post hoc test are presented. To see peak intensities for each protein fragment please check box plots shared in the Mendeley Data Repository (doi: 10.17632/p656wtzjv8.1).

				p-value of Kruskal-Wallis	p-value of Dunn's post-hoc test		
m/z values	Sequence	Protein name	Analyzed *TR*	*post-LVC ectasia vs control vs KTCN*	*post-LVC ectasia vs KTCN*	*post-LVC ectasia vs control*	*post-LVC ectasia vs control*
777.3853583	x	x	*1st TR*	0.027	0.215	0.059	0.009
			2nd TR	0.055	0.366	0.064	0.021
			3rd TR	0.059	0.944	0.025	0.060
810.4064156	x	x	*1st TR*	0.011	0.042	0.142	0.003
			2nd TR	0.053	0.224	0.104	0.017
			3rd TR	0.0409	0.079	0.184	0.396
821.1495891	R.LAAEDFR.M	Keratin, type I cytoskeletal 12	*1st TR*	< 0.001	0.078	0.004	< 0.001
			2nd TR	0.009	0.070	0.074	0.002
			3rd TR	0.027	0.734	0.015	0.023
822.413646	R.LAAEDFR.M	Keratin, type I cytoskeletal 12	*1st TR*	< 0.001	0.035	0.018	< 0.001
			2nd TR	0.004	0.051	0.048	< 0.001
			3rd TR	0.012	0.253	0.024	0.004
865.3901057	K.DGFNNTR.K	Zinc finger protein 106 homolog	*1st TR*	0.009	0.024	0.191	0.002
			2nd TR	0.076	0.177	0.185	0.023
			3rd TR	0.056	0.185	0.135	0.017
896.4314275	R.SSSHSPPR.H	Pre-mRNA-splicing factor CWC25 homolog	*1st TR*	0.001	0.100	0.010	< 0.001
			2nd TR	0.346	0.725	0.216	0.188
			3rd TR	0.007	0.808	0.002	0.023
945.5224892	K.RGNIHPPR.D	Galactose-3-O-sulfotransferase 4	*1st TR*	0.701	0.532	0.729	0.405
			2nd TR	0.278	0.409	0.292	0.113
			3rd TR	0.050	0.246	0.089	0.016
946.5231131	K.RGNIHPPR.D	Galactose-3-O-sulfotransferase 4	*1st TR*	0.380	0.832	0.169	0.353
			2nd TR	0.090	0.555	0.067	0.044
			3rd TR	0.017	0.216	0.038	0.005
961.4794575	K.NVDTNQDR.L	Nucleobindin-1	*1st TR*	0.528	0.896	0.267	0.432
			2nd TR	0.202	0.482	0.075	0.409
			3rd TR	0.032	0.185	0.079	0.009
973.5207475	K.DKILESIR.Q	ATP-binding cassette sub-family A member 13	*1st TR*	0.194	0.108	0.765	0.096
			2nd TR	0.151	0.412	0.154	0.060
			3rd TR	0.033	0.609	0.022	0.021
975.5197322	K.DKILESIR.Q	ATP-binding cassette sub-family A member 13	*1st TR*	0.003	0.101	0.019	< 0.001
			2nd TR	0.003	0.028	0.068	< 0.001

			3rd TR	0.033	0.609	0.022	0.021
976.473973	x	x	*1st TR*	0.045	0.073	0.270	0.013
			2nd TR	0.041	0.189	0.097	0.012
			3rd TR	0.019	0.941	0.007	0.034
1005.46904	x	x	*1st TR*	0.022	0.199	0.052	0.007
			2nd TR	0.024	0.134	0.085	0.006
			3rd TR	0.083	0.988	0.033	0.086
1007.506637	R.SYDEAILR.L	Butyrophilin subfamily 2 member A2	*1st TR*	0.207	0.390	0.224	0.081
			2nd TR	0.097	0.708	0.056	0.060
			3rd TR	0.007	0.333	0.010	0.003
1008.504567	R.SYDEAILR.L	Butyrophilin subfamily 2 member A2	*1st TR*	0.064	0.221	0.127	0.020
			2nd TR	0.044	0.444	0.042	0.020
			3rd TR	0.030	0.468	0.028	0.015
1009.508715	R.SYDEAILR.L	Butyrophilin subfamily 2 member A2	*1st TR*	0.089	0.255	0.149	0.029
			2nd TR	0.055	0.291	0.083	0.019
			3rd TR	0.012	0.365	0.016	0.006
1016.529016	R.REAPYGAPR.F	Latent-transforming growth factor beta-binding protein 4	*1st TR*	0.220	0.212	0.441	0.084
			2nd TR	0.096	0.195	0.201	0.030
			3rd TR	0.027	0.769	0.009	0.061
1045.560265	R.VLPDMVSLR.V	MHC class II transactivator	*1st TR*	0.124	0.125	0.414	0.044
			2nd TR	0.151	0.341	0.186	0.056
			3rd TR	0.060	0.120	0.218	0.018
1046.553857	R.VLPDMVSLR.V	MHC class II transactivator	*1st TR*	0.136	0.178	0.322	0.046
			2nd TR	0.173	0.258	0.283	0.061
			3rd TR	0.101	0.302	0.141	0.035
1059.598382	R.VLDELTLTR.T	Keratin, type I cytoskeletal 12	*1st TR*	0.012	0.127	0.049	0.003
			2nd TR	0.013	0.117	0.057	0.003
			3rd TR	0.033	0.447	0.032	0.016
1060.586979	R.VLDELTLTR.T	Keratin, type I cytoskeletal 12	*1st TR*	0.018	0.156	0.056	0.005
			2nd TR	0.010	0.190	0.029	0.003
			3rd TR	0.028	0.431	0.028	0.013
1061.581164	R.VLDELTLTR.T	Keratin, type I cytoskeletal 12	*1st TR*	0.015	0.124	0.061	0.004
			2nd TR	0.014	0.183	0.038	0.004
			3rd TR	0.012	0.530	0.010	0.008
1075.572561	R.RPHFFFPK.S	Clusterin	*1st TR*	0.011	0.422	0.012	0.006
			2nd TR	0.060	0.152	0.175	0.018
			3rd TR	0.001	0.805	< 0.001	0.003
1097.510592	K.DLSKDQHGR.N	Dedicator of cytokinesis protein 8	*1st TR*	0.022	0.031	0.331	0.007
			2nd TR	0.075	0.239	0.136	0.024
			3rd TR	0.031	0.129	0.112	0.008
1104.585083	R.QGLSMRQIR.F	Small ubiquitin-related modifier 2	*1st TR*	0.040	0.119	0.154	0.011
			2nd TR	0.112	0.465	0.101	0.048
			3rd TR	0.056	0.752	0.030	0.041
1108.556254	x	x	*1st TR*	0.088	0.131	0.288	0.028

			2nd TR	0.680	0.863	0.383	0.579
			3rd TR	0.020	0.065	0.150	0.005
1109.55798	x	x	*1st TR*	0.491	0.414	0.564	0.234
			2nd TR	0.453	0.684	0.208	0.507
			3rd TR	0.017	0.124	0.070	0.005
1112.56154	R.QDIAFAYQR.R	Annexin A2	*1st TR*	0.241	0.185	0.559	0.100
			2nd TR	0.147	0.379	0.163	0.056
			3rd TR	0.047	0.393	0.052	0.019
1133.560061	x	x	*1st TR*	0.101	0.154	0.279	0.032
			2nd TR	0.083	0.384	0.092	0.032
			3rd TR	0.026	0.528	0.021	0.015
1141.525265	K.EELDQQNKR.F	Golgin subfamily A member 4	*1st TR*	0.028	0.093	0.144	0.008
			2nd TR	0.042	0.107	0.177	0.012
			3rd TR	0.020	0.213	0.045	0.006
1152.574192	K.IREWYETR.G	Keratin, type I cytoskeletal 12	*1st TR*	0.745	0.987	0.462	0.560
			2nd TR	0.189	0.681	0.069	0.265
			3rd TR	0.023	0.507	0.020	0.013
1153.579693	K.IREWYETR.G	Keratin, type I cytoskeletal 12	*1st TR*	0.617	0.863	0.385	0.391
			2nd TR	0.310	0.833	0.131	0.299
			3rd TR	0.007	0.258	0.015	0.003
1154.577639	K.IREWYETR.G	Keratin, type I cytoskeletal 12	*1st TR*	0.499	0.591	0.404	0.249
			2nd TR	0.232	0.948	0.100	0.201
			3rd TR	0.021	0.348	0.028	0.009
1163.612106	R.LFDQAFGLPR.L	Heat shock protein beta-1	*1st TR*	0.954	0.758	0.932	0.839
			2nd TR	0.258	0.714	0.101	0.313
			3rd TR	0.013	0.259	0.024	0.005
1164.612131	R.LFDQAFGLPR.L	Heat shock protein beta-1	*1st TR*	0.930	0.717	0.821	0.892
			2nd TR	0.286	0.442	0.121	0.560
			3rd TR	0.033	0.330	0.044	0.013
1165.606688	R.LFDQAFGLPR.L	Heat shock protein beta-1	*1st TR*	0.736	0.058	0.087	0.221
			2nd TR	0.110	0.923	0.049	0.092
			3rd TR	0.008	0.324	0.012	0.004
1166.60228	R.LFDQAFGLPR.L	Heat shock protein beta-1	*1st TR*	0.342	0.339	0.445	0.143
			2nd TR	0.041	0.555	0.031	0.023
			3rd TR	0.005	0.074	0.039	0.001
1171.605614	K.APGSKGSCPLR.K	C-type lectin domain family 4 member F	*1st TR*	0.122	0.381	0.134	0.046
			2nd TR	0.063	0.527	0.050	0.031
			3rd TR	0.016	0.167	0.047	0.005
1221.639817	x	x	*1st TR*	0.080	0.093	0.362	0.026
			2nd TR	0.033	0.082	0.186	0.009
			3rd TR	0.140	0.139	0.421	0.050
1266.653945	R.AADVEPSSPKPK.R	Putative NPIP-like protein LOC613037	*1st TR*	0.014	0.667	0.009	0.012
			2nd TR	0.260	0.469	0.238	0.110
			3rd TR	0.038	0.978	0.015	0.046

1267.652091	R.AADVEPSSPKPK.R	Putative NPIP-like protein LOC613037	*1st TR*	0.011	0.743	0.006	0.012
			2nd TR	0.283	0.239	0.511	0.116
			3rd TR	0.027	0.936	0.011	0.034
1268.649403	R.AADVEPSSPKPK.R	Putative NPIP-like protein LOC613037	*1st TR*	0.032	0.710	0.018	0.025
			2nd TR	0.379	0.291	0.589	0.170
			3rd TR	0.033	0.778	0.011	0.068
1274.578974	R.QLLPGDEFSLR.E	Insulin gene enhancer protein ISL-2	*1st TR*	0.028	0.175	0.076	0.008
			2nd TR	0.135	0.319	0.178	0.048
			3rd TR	0.033	0.282	0.053	0.012
1275.581293	R.QLLPGDEFSLR.E	Insulin gene enhancer protein ISL-2	*1st TR*	0.037	0.152	0.110	0.010
			2nd TR	0.118	0.225	0.221	0.039
			3rd TR	0.045	0.320	0.062	0.017
1276.580218	R.QLLPGDEFSLR.E	Insulin gene enhancer protein ISL-2	*1st TR*	0.017	0.127	0.066	0.005
			2nd TR	0.170	0.338	0.212	0.063
			3rd TR	0.010	0.178	0.030	0.003
1322.655187	K.NRSQGNIISYK.R	Transmembrane protein 217	*1st TR*	0.267	0.337	0.340	0.105
			2nd TR	0.194	0.463	0.177	0.081
			3rd TR	0.03	0.436	0.034	0.016
1323.650852	K.NRSQGNIISYK.R	Transmembrane protein 217	*1st TR*	0.294	0.342	0.373	0.118
			2nd TR	0.175	0.644	0.114	0.091
			3rd TR	0.017	0.241	0.033	0.006
1327.704029	K.TPSKDSLDPDPR.C	WD repeat-containing protein 62	*1st TR*	0.016	0.206	0.038	0.005
			2nd TR	0.047	0.108	0.196	0.013
			3rd TR	0.151	0.589	0.107	0.074
1328.696177	K.TPSKDSLDPDPR.C	WD repeat-containing protein 62	*1st TR*	0.021	0.200	0.051	0.007
			2nd TR	0.101	0.267	0.160	0.034
			3rd TR	0.139	0.608	0.095	0.071
1329.691889	K.TPSKDSLDPDPR.C	WD repeat-containing protein 62	*1st TR*	0.019	0.143	0.067	0.005
			2nd TR	0.053	0.220	0.107	0.017
			3rd TR	0.078	0.337	0.099	0.029
1345.630926	R.DQLRGHPDIPR.L	Protein FAM186B	*1st TR*	0.402	0.370	0.493	0.177
			2nd TR	0.122	0.334	0.153	0.044
			3rd TR	0.037	0.251	0.066	0.012
1346.630597	R.DQLRGHPDIPR.L	Protein FAM186B	*1st TR*	0.492	0.442	0.528	0.233
			2nd TR	0.102	0.290	0.149	0.035
			3rd TR	.027	0.197	0.064	0.008
1355.775775	x	x	*1st TR*	0.023	0.588	0.017	0.015
			2nd TR	0.275	0.228	0.521	0.113
			3rd TR	0.526	0.978	0.285	0.372
1418.750943	K.VDALNDEINFLR.T	Keratin, type II cytoskeletal 7	*1st TR*	0.496	0.479	0.493	0.237
			2nd TR	0.039	0.388	0.043	0.016
			3rd TR	0.251	0.633	0.170	0.125
1419.752533	K.VDALNDEINFLR.T	Keratin, type II cytoskeletal 7	*1st TR*	0.582	0.445	0.661	0.303
			2nd TR	0.041	0.398	0.045	0.017

			3rd TR	0.234	0.626	0.160	0.116
1420.751756	K.VDALNDEINFLR.T	Keratin, type II cytoskeletal 7	*1st TR*	0.470	0.345	0.646	0.227
			2nd TR	0.042	0.301	0.061	0.015
			3rd TR	0.152	0.495	0.129	0.066
1450.740819	R.QFIMPVVSAISSR.I	Histone-lysine N-methyltransferase MLL	*1st TR*	0.032	0.285	0.050	0.011
			2nd TR	0.234	0.299	0.333	0.088
			3rd TR	0.177	0.685	0.109	0.097
1451.735498	R.QFIMPVVSAISSR.I	Histone-lysine N-methyltransferase MLL	*1st TR*	0.029	0.291	0.046	0.011
			2nd TR	0.122	0.159	0.323	0.041
			3rd TR	0.159	0.498	0.133	0.069
1452.73821	R.QFIMPVVSAISSR.I	Histone-lysine N-methyltransferase MLL	*1st TR*	0.041	0.231	0.081	0.013
			2nd TR	0.101	0.177	0.244	0.032
			3rd TR	0.097	0.418	0.097	0.039
1472.715506	K.SSQLDSGVPSSPGGR.Q	Ubiquitin carboxyl-terminal hydrolase 31	*1st TR*	0.026	0.186	0.066	0.008
			2nd TR	0.082	0.219	0.162	0.026
			3rd TR	0.045	0.492	0.039	0.022
1486.707677	x	x	*1st TR*	0.133	0.340	0.164	0.049
			2nd TR	0.332	0.392	0.373	0.139
			3rd TR	0.044	0.877	0.016	0.067
1540.778881	K.VVIGMDVAASEFFR.S	Alpha-enolase	*1st TR*	0.042	0.094	0.203	0.012
			2nd TR	0.090	0.121	0.316	0.029
			3rd TR	0.558	0.400	0.700	0.291
1763.82384	K.LISWYDNEFGYSNR.V	Glyceraldehyde-3-phosphate dehydrogenase	*1st TR*	0.004	0.151	0.016	0.001
			2nd TR	0.076	0.050	0.621	0.033
			3rd TR	0.813	0.574	0.638	0.909
1764.81506	K.LISWYDNEFGYSNR.V	Glyceraldehyde-3-phosphate dehydrogenase	*1st TR*	0.002	0.104	0.014	< 0.001
			2nd TR	0.039	0.037	0.458	0.014
			3rd TR	0.860	0.796	0.703	0.590
1765.817217	K.EGEAVVLPEVEPGLTAR.E	Syndecan-1	*1st TR*	0.030	0.078	0.183	0.008
			2nd TR	0.016	0.039	0.199	0.004
			3rd TR	0.869	0.871	0.596	0.774
1795.844542	x	x	*1st TR*	0.775	0.491	0.977	0.559
			2nd TR	0.417	0.479	0.200	0.678
			3rd TR	0.036	0.050	0.317	0.011
1859.901	x	x	*1st TR*	0.020	0.203	0.047	0.006
			2nd TR	0.221	0.362	0.259	0.085
			3rd TR	0.062	0.346	0.077	0.023
1860.904692	x	x	*1st TR*	0.017	0.077	0.112	0.004
			2nd TR	0.004	0.018	0.138	< 0.001
			3rd TR	0.025	0.069	0.172	0.007
1861.916282	x	x	*1st TR*	0.165	0.549	0.126	0.076
			2nd TR	0.174	0.683	0.107	0.095
			3rd TR	0.009	0.030	0.162	0.002
1863.96948	x	x	*1st TR*	0.466	0.495	0.441	0.219

			2nd TR	0.575	0.311	0.562	0.671
			3rd TR	0.017	0.795	0.005	0.042
1864.92974	x	x	*1st TR*	0.134	0.224	0.252	0.045
			2nd TR	0.934	0.721	0.837	0.882
			3rd TR	0.018	0.717	0.005	0.052
1902.909032	x	x	*1st TR*	0.018	0.194	0.046	0.006
			2nd TR	0.063	0.175	0.158	0.019
			3rd TR	0.068	0.974	0.026	0.076
1988.006537	x	x	*1st TR*	0.016	0.206	0.038	0.005
			2nd TR	0.034	0.079	0.198	0.009
			3rd TR	0.063	0.666	0.039	0.039
1989.006468	x	x	*1st TR*	0.007	0.239	0.015	0.003
			2nd TR	0.037	0.076	0.222	0.010
			3rd TR	0.065	0.594	0.045	0.036
1989.983248	M.VNPTVFFDIAVDGEPLGR.V	Peptidyl-prolyl cis-trans isomerase A	*1st TR*	0.007	0.180	0.022	0.002
			2nd TR	0.013	0.130	0.053	0.004
			3rd TR	0.050	0.300	0.073	0.018
1990.973795	M.VNPTVFFDIAVDGEPLGR.V	Peptidyl-prolyl cis-trans isomerase A	*1st TR*	0.008	0.151	0.029	0.002
			2nd TR	0.038	0.420	0.039	0.017
			3rd TR	0.161	0.573	0.118	0.077
2011.10915	K.QLVAGAVAGAVSRTGTAPLDR.L	Calcium-binding mitochondrial carrier protein SCaMC-3	*1st TR*	0.904	0.696	0.927	0.675
			2nd TR	0.241	0.779	0.095	0.268
			3rd TR	0.028	0.140	0.096	0.008
2012.10652	K.QLVAGAVAGAVSRTGTAPLDR.L	Calcium-binding mitochondrial carrier protein SCaMC-3	*1st TR*	0.970	0.804	0.955	0.863
			2nd TR	0.198	0.745	0.113	0.115
			3rd TR	0.035	0.152	0.107	0.010
2033.050567	R.LLQQLAMTGSEEGDPRTK.S	GTPase-activating protein and VPS9 domain-containing protein 1	*1st TR*	0.659	0.563	0.621	0.361
			2nd TR	0.400	0.406	0.445	0.176
			3rd TR	0.049	0.605	0.033	0.029
2048.062733	R.QNLEPLFEQYINNLRR.Q	Keratin, type II cytoskeletal 5	*1st TR*	0.656	0.382	0.591	0.737
			2nd TR	0.401	0.548	0.333	0.188
			3rd TR	0.038	0.015	0.140	0.341
2106.105774	R.LIQEQEQELVGALAADLHK.N	Aldehyde dehydrogenase, dimeric NADP-preferring	*1st TR*	0.048	0.837	0.023	0.043
			2nd TR	0.262	0.333	0.337	0.102
			3rd TR	0.809	0.705	0.536	0.866
2265.108064	x	x	*1st TR*	0.008	0.011	0.373	0.003
			2nd TR	0.225	0.097	0.332	0.499
			3rd TR	0.797	0.693	0.522	0.864
2266.106452	x	x	*1st TR*	0.024	0.018	0.563	0.011
			2nd TR	0.051	0.022	0.926	0.036
			3rd TR	0.489	0.378	0.618	0.236
2306.144385	R.TLQSNGIIMYTRANPCIILK.I	Protocadherin Fat 3	*1st TR*	0.087	0.172	0.218	0.027
			2nd TR	0.002	0.498	0.002	0.002
			3rd TR	0.069	0.274	0.109	0.023

2307.145093	R.TLQSNGIIMYTRANPCIILK.I	Protocadherin Fat 3	*1st TR*	0.116	0.168	0.291	0.038
			2nd TR	0.001	0.423	0.002	0.001
			3rd TR	0.074	0.305	0.104	0.026
2308.144862	R.TLQSNGIIMYTRANPCIILK.I	Protocadherin Fat 3	*1st TR*	0.090	0.117	0.328	0.029
			2nd TR	0.002	0.428	0.002	0.001
			3rd TR	0.088	0.290	0.129	0.030
2309.144491	R.TLQSNGIIMYTRANPCIILK.I	Protocadherin Fat 3	*1st TR*	0.094	0.102	0.389	0.032
			2nd TR	0.001	0.368	0.002	<. 001
			3rd TR	0.047	0.279	0.075	0.016
2310.144162	R.TLQSNGIIMYTRANPCIILK.I	Protocadherin Fat 3	*1st TR*	0.048	0.110	0.195	0.014
			2nd TR	0.002	0.450	0.002	0.002
			3rd TR	0.023	0.289	0.036	0.008
2311.146427	R.TLQSNGIIMYTRANPCIILK.I	Protocadherin Fat 3	*1st TR*	0.420	0.471	0.407	0.190
			2nd TR	0.002	0.512	0.002	0.002
			3rd TR	0.065	0.425	0.065	0.027
2330. 074755	x	x	*1st TR*	0.953	0.949	0.788	0.784
			2nd TR	0.046	0.180	0.017	0.447
			3rd TR	0.392	0.580	0.174	0.0537
2354.14968	x	x	*1st TR*	0.047	0.310	0.066	0.017
			2nd TR	0.208	0.427	0.206	0.084
			3rd TR	0.512	0.401	0.617	0.251
2381.175688	x	x	*1st TR*	0.279	0.633	0.192	0.138
			2nd TR	0.078	0.133	0.251	0.024
			3rd TR	0.048	0.204	0.105	0.015
2392.169511	x	x	*1st TR*	0.329	0.293	0.494	0.138
			2nd TR	0.142	0.240	0.249	0.048
			3rd TR	0.017	0.026	0.306	0.005
2403.23436	K.LYLQDNAISHIPYNTLAKMR.E	Leucine-rich repeat transmembrane protein FLRT1	*1st TR*	0.979	0.967	0.838	0.897
			2nd TR	0.456	0.442	0.239	0.781
			3rd TR	0.045	0.014	0.713	0.062
2432.132664	R.KQYRPDMMSLQIQMYQLSR.L	TBC1 domain family member 4	*1st TR*	0.006	0.013	0.255	0.002
			2nd TR	0.037	0.041	0.389	0.012
			3rd TR	0.007	0.127	0.032	0.002
2433.138151	R.KQYRPDMMSLQIQMYQLSR.L	TBC1 domain family member 4	*1st TR*	0.008	0.011	0.355	0.003
			2nd TR	0.023	0.013	0.708	0.013
			3rd TR	0.014	0.205	0.033	0.004
2434.139292	R.KQYRPDMMSLQIQMYQLSR.L	TBC1 domain family member 4	*1st TR*	0.006	0.008	0.373	0.002
			2nd TR	0.023	0.018	0.555	0.010
			3rd TR	0.076	0.265	0.123	0.025
2435.141433	R.KQYRPDMMSLQIQMYQLSR.L	TBC1 domain family member 4	*1st TR*	0.003	0.008	0.223	< 0.001
			2nd TR	0.017	0.006	0.961	0.018
			3rd TR	0.029	0.329	0.039	0.011
2436.147519	R.KQYRPDMMSLQIQMYQLSR.L	TBC1 domain family member 4	*1st TR*	< 0.001	< 0.001	0.079	< 0.001
			2nd TR	0.027	0.023	0.499	0.011

			3rd TR	0.206	0.377	0.232	0.080
2541.33711	R.QDNSILKVLISMQLMSGDPCFK.T	Protein FAM102B	*1st TR*	0.373	0.954	0.176	0.294
			2nd TR	0.464	0.218	0.621	0.492
			3rd TR	0.048	0.033	0.601	0.021
2772.332818	x	x	*1st TR*	0.014	0.070	0.103	0.003
			2nd TR	0.027	0.049	0.256	0.008
			3rd TR	0.112	0.151	0.314	0.037
2773.33583	x	x	*1st TR*	0.008	0.042	0.101	0.002
			2nd TR	0.008	0.080	0.055	0.002
			3rd TR	0.204	0.217	0.398	0.076
2800.348426	x	x	*1st TR*	0.325	0.485	0.294	0.142
			2nd TR	0.363	0.275	0.596	0.162
			3rd TR	0.024	0.020	0.502	0.009
2997.43113	x	x	*1st TR*	0.050	0.223	0.100	0.016
			2nd TR	0.402	0.187	0.893	0.291
			3rd TR	0.170	0.207	0.345	0.060
3185.612433	R.TTGIVMDSGDGVTHTVPIYEGYALPHAILR.L	Actin, cytoplasmic 1	*1st TR*	0.136	0.149	0.381	0.047
			2nd TR	0.021	0.011	0.804	0.014
			3rd TR	0.066	0.052	0.526	0.026

x- stands for peaks unidentified to peptides/proteins
Abbreviations: post-LVC ectasia – post-laser vision correction ectasia; KTCN – keratoconus; *1stTR – central topographic region*, *2ndTR – middle topographic region*, *3rdTR – peripheral topographic*; log2FC - log2 transformed fold change.

Table S17. The RT-qPCR data used for the original RNA-Seq data verification. The Ct values obtained towards verification of the relative expression of *CADPS*, *CPT1B*, *S100A14*, and *KLK5* genes in each of 72 originally CE samples collected in the study. The expression of *LDHA*, *RLP4*, and *UBC* genes was used for data normalization. Each reaction was performed in triplicates, and if one of the replicates deviated by ≥0.5 Ct it was removed (these replicates are marked below as 'NaN').

Sample	*LDHA*	*LDHA*	*LDHA*	*RPL4*	*RPL4*	*RPL4*	*UBC*	*UBC*	*UBC*	*CADPS*	*CADPS*	*CADPS*	*S100A14*	*S100A14*	*S100A14*	*CPT1B*	*CPT1B*	*CPT1B*	*KLK5*	*KLK5*	*KLK5*
1 OPT/M/OS/1	20.83	20.74	20.70	20.26	20.10	19.99	20.46	NaN	20.16	26.02	25.81	25.90	22.16	22.26	22.11						
1 OPT/M/OS/2	20.37	20.29	20.26	20.13	20.15	20.15	20.04	20.10	20.30	25.86	25.99	26.01				25.74	25.41	25.53			
1 OPT/M/OS/3	20.14	20.11	20.58	19.48	19.41	19.39	19.47	19.69	19.58	25.49	25.28	25.24	22.00	22.02	22.01				28.19	28.33	28.26
1 OPT/M/OD/1	20.97	20.70	20.57	20.24	20.24	20.15	20.05	20.12	20.06	25.87	25.94	26.02	22.11	22.09	21.45						
1 OPT/M/OD/2	20.70	20.81	20.37	20.58	20.50	21.05	20.15	20.15	20.55	26.54	26.72	27.40				25.37	25.82	25.74			
1 OPT/M/OD/3	20.25	20.53	20.62	19.62	19.68	19.54	19.63	19.96	19.87	25.59	25.43	25.66	22.07	22.14	22.10				28.21	28.16	28.17
10 OPT/M/OS/1	23.73	24.28	23.64	23.72	23.76	23.69	22.14	22.43	22.08	29.71	29.72	29.97	25.27	25.17	25.19						
10 OPT/M/OS/2	24.47	24.58	24.16	24.15	24.13	24.36	22.95	22.57	22.94	30.04	29.82	30.12				27.56	27.70	27.96			
10 OPT/M/OS/3	21.32	21.30	21.24	20.77	20.36	20.02	19.79	19.93	20.00	26.06	26.03	26.08	22.53	22.29	22.39				28.27	28.39	28.31
10 OPT/M/OD/1	27.80	27.89	27.76	27.03	NaN	27.12	26.12	26.04	26.00	33.28	34.10	34.22	29.51	29.22	29.74						
10 OPT/M/OD/2	25.57	25.74	25.39	25.41	25.22	25.11	23.97	23.95	23.82	30.75	30.79	30.75				28.48	28.13	28.20			
10 OPT/M/OD/3	21.21	21.00	21.17	20.33	19.31	19.94	19.62	20.05	20.09	26.08	26.17	26.23	22.32	22.32	22.71				29.24	29.07	28.81
11 OPT/M/OS/1	25.76	25.89	25.55	25.35	25.34	25.36	23.30	23.53	23.32	33.30	33.32	32.64	27.23	26.81	26.97						
11 OPT/M/OS/2	25.16	25.05	26.01	24.63	24.24	24.04	23.07	22.79	22.85	32.07	32.29	32.08				27.50	27.72	28.07			
11 OPT/M/OS/3	20.84	20.94	20.58	20.12	NaN	19.60	18.66	18.98	19.05	27.21	27.19	26.78	21.53	21.81	22.07						
11 OPT/M/OD/1	26.26	26.28	26.15	25.81	26.23	26.05	24.15	24.00	23.81	34.09	34.03	33.71	27.39	27.59	27.81						
11 OPT/M/OD/2	23.43	22.63	22.79	22.78	22.57	22.63	20.85	21.27	21.28	30.37	30.48	30.41				25.99	26.56	NaN			
11 OPT/M/OD/3	21.26	21.29	21.25	21.17	20.23	20.16	19.62	19.70	19.73	28.57	28.45	28.21	22.38	22.31	22.24				34.00	34.44	NaN
128 OPT/E/OD/1	20.43	20.05	20.29	21.07	20.44	20.56	19.95	19.75	20.03	29.74	29.43	29.93	22.16	22.56	22.73						
128 OPT/E/OD/2	23.37	23.53	23.28	23.41	23.75	23.32	22.59	22.40	22.51	33.30	33.49	33.84				31.07	31.03	30.84			
128 OPT/E/OD/3	20.20	20.45	20.46	20.69	20.45	20.39	20.32	19.98	20.25	29.29	29.50	29.61	22.76	22.78	22.86				25.56	25.69	25.92
136 OPT/E/OD/1	22.15	21.81	21.82	21.87	22.04	21.91	20.98	21.28	21.25	31.59	32.14	32.05	24.19	24.02	24.60						
136 OPT/E/OD/2	20.50	20.58	19.76	20.41	20.45	20.31	20.39	20.23	20.37	30.56	30.01	30.38				28.78	28.65	28.70			
136 OPT/E/OD/3	19.77	20.09	20.43	19.68	19.56	19.87	20.22	20.23	20.07	30.37	30.93	30.11	22.14	22.37	22.22				25.35	25.58	25.61
17 OPT/E/OS/1	20.60	20.25	20.32	20.79	20.58	20.83	20.41	20.24	21.29	31.05	31.22	31.19	22.32	22.26	22.17						
17 OPT/E/OS/2	20.75	20.12	20.19	20.41	20.36	20.41	20.18	20.46	20.75	31.46	31.01	31.11				27.02	26.93	26.95			
17 OPT/E/OS/3	20.08	19.73	19.91	19.87	19.97	20.14	20.53	20.55	20.09	31.34	31.00	31.31	21.52	21.65	21.74				24.91	24.67	24.72
26 OPT/E/OD/1	20.31	20.64	20.28	20.37	20.19	20.38	20.56	19.76	19.89	31.38	31.71	31.68	22.18	22.40	22.05						
26 OPT/E/OD/2	25.80	25.31	25.23	25.02	25.03	25.23	24.19	24.38	24.27	35.72	34.70	35.29				31.16	31.06	31.24			
26 OPT/E/OD/3	20.03	20.44	20.51	20.09	20.01	20.08	20.08	20.02	20.04	29.77	29.73	30.24	22.45	22.89	22.77				26.34	26.42	26.34
27 OPT/E/OS/1	20.27	20.68	20.55	20.42	20.35	20.45	20.20	20.06	20.25	29.00	29.08	28.97	22.37	22.44	22.26						
27 OPT/E/OS/2	20.68	20.22	20.55	19.91	20.18	20.06	20.20	20.45	20.27	28.92	28.60	28.70				28.08	28.10	28.04			
27 OPT/E/OS/3	20.37	20.45	20.30	19.93	19.77	19.91	20.13	20.11	20.17	29.69	29.64	30.03	22.28	22.39	22.38				25.86	26.07	25.98
31 OPT/E/OD/1	21.75	21.94	21.48	21.83	21.64	NaN	20.94	21.13	21.17	29.66	29.89	29.71	23.73	23.56	23.45						
31 OPT/E/OD/2	23.71	23.31	23.36	23.31	23.45	23.36	22.51	22.64	22.27	30.38	30.45	30.47				29.78	29.53	29.91			
31 OPT/E/OD/3	20.11	20.23	20.49	19.87	19.69	19.81	19.70	19.54	19.69	28.18	28.21	28.20	23.14	22.99	23.24				25.35	25.13	NaN
4 OPT/M/OS/1	20.73	20.79	20.64	20.46	20.26	20.09	NaN	20.42	19.92	27.86	NaN	27.80	22.60	22.55	22.51						

4 OPT/M/OS/2	20.10	20.01	20.01	20.13	20.05	20.25	19.86	19.82	19.61	27.64	27.57	27.85				25.53	25.22	25.67			
4 OPT/M/OS/3	19.84	20.03	20.11	19.73	19.67	19.78	19.21	19.19	19.27	26.68	26.31	26.41	22.13	22.08	22.16				27.97	27.96	28.10
4 OPT/M/OD/1	20.55	20.22	19.99	20.00	20.10	19.75	19.46	19.50	19.20	27.30	27.41	27.48	22.27	22.20	22.22						
4 OPT/M/OD/2	19.95	20.15	20.07	20.23	20.00	20.22	19.70	19.31	19.72	27.44	27.62	27.52				25.70	25.28	25.65			
4 OPT/M/OD/3	19.57	20.07	20.10	19.86	19.53	19.83	19.03	19.22	19.09	27.11	26.89	27.02	22.08	22.13	22.10				28.08	27.90	27.88
5 OPT/M/OS/1	21.15	21.16	20.58	20.53	20.67	20.25	21.08	20.42	20.49	27.46	27.52	27.44	22.68	22.66	22.61						
5 OPT/M/OS/2	20.48	20.26	19.93	20.23	20.14	20.19	21.08	21.08	21.08	27.00	27.12	27.30				24.94	24.37	24.08			
5 OPT/M/OS/3	20.37	20.93	20.67	19.50	19.43	19.78	20.06	20.12	20.02	26.47	26.65	26.43	22.52	22.42	22.42				24.99	24.79	24.82
5 OPT/M/OD/1	21.51	21.53	21.34	21.09	21.08	20.77	21.74	21.02	20.96	27.97	28.39	27.95	22.91	23.07	23.23						
5 OPT/M/OD/2	20.43	20.40	20.37	20.41	20.39	20.45	20.35	20.51	20.38	27.37	27.34	27.36				24.84	25.03	24.80			
5 OPT/M/OD/3	20.27	20.18	20.40	20.29	19.89	19.97	19.74	20.11	19.91	26.81	26.63	26.47	22.25	22.33	22.46				24.86	25.00	24.84
51 OPT/E/OS/1	20.70	20.56	20.31	20.95	20.88	20.80	20.33	20.31	20.39	28.80	29.13	29.00	21.96	21.87	21.93						
51 OPT/E/OS/2	20.66	20.11	20.14	20.16	20.21	20.02	20.63	20.69	20.55	28.52	28.32	28.57				28.42	28.23	27.96			
51 OPT/E/OS/3	20.22	20.40	20.25	20.19	20.05	20.20	20.64	20.46	20.25	28.70	28.74	28.75	21.58	21.65	21.82				26.01	26.00	26.00
6 OPT/M/OS/1	19.83	20.25	19.75	19.96	19.73	19.82	19.78	19.38	19.77	27.67	27.37	27.78		21.46	21.40						
6 OPT/M/OS/2	19.58	20.14	19.31	19.94	20.03	19.63	20.17	19.53	19.87	27.47	27.67	27.65				25.46	25.43	25.95			
6 OPT/M/OS/3	19.06	18.90	18.85	18.46	18.47	18.03	18.49	18.51	18.74	26.18	25.99	26.20	20.51	20.78	21.09				22.91	23.10	22.77
6 OPT/M/OD/1	26.68	26.47	26.18	26.22	26.07	25.73	25.34	25.03	25.30	32.78	NaN	32.50	28.25	28.42	28.12						
6 OPT/M/OD/2	20.37	20.45	20.23	20.39	20.41	20.44	19.76	19.47	19.70	28.25	28.51	28.56				25.67	25.27	25.18			
6 OPT/M/OD/3	19.47	19.94	19.80	19.35	19.12	19.25	18.59	18.64	18.54	27.10	27.05	26.29	21.40	21.28	21.28						
62 OPT/E/OS/1	25.26	25.45	25.54	25.50	25.30	25.36	24.33	24.28	24.20	NaN	37.42	36.13	27.02	27.18	27.08						
62 OPT/E/OS/2	23.17	22.96	22.90	23.13	23.20	23.34	22.03	22.10	22.03	34.84	34.57	35.75				30.09	30.07	30.33			
62 OPT/E/OS/3	19.62	19.96	20.31	20.44	20.18	20.13	20.12	20.00	20.25	31.74	32.04	31.99	21.29	21.62	21.56				24.76	24.83	24.83
7 OPT/M/OD/1	20.82	20.96	20.43	20.23	20.46	20.24	NaN	19.69	19.52	27.72	NaN	27.76	22.32	22.30	22.65						
7 OPT/M/OD/2	20.36	20.57	19.91	20.20	20.21	20.18	19.76	19.34	19.41	27.95	27.75	28.07				24.92	24.36	NaN			
7 OPT/M/OD/3	19.73	20.09	20.17	19.71	19.57	19.82	19.01	18.87	18.88	27.49	26.94	27.16	22.32	22.31	22.24				24.14	24.06	23.98
72 OPT/E/OD/1	23.93	23.92	24.55	23.88	23.78	23.55	22.21	22.49	22.24	31.52	31.50	31.39	26.09	26.26	26.11						
72 OPT/E/OD/2	21.02	20.28	20.65	20.26	20.50	20.43	20.27	19.97	20.05	29.43	29.38	29.85				28.77	28.58	28.47			
72 OPT/E/OD/3	20.20	20.29	20.55	20.13	20.10	20.08	19.86	19.74	20.04	29.12	29.07	29.21	22.17	22.20	22.04				26.01	26.03	26.11
8 OPT/M/OD/1	20.75	20.74	20.52	20.67	20.87	20.72	19.69	19.83	19.76	28.35	28.91	28.41	22.44	22.38	22.32						
8 OPT/M/OD/2	20.38	20.65	20.66	20.45	20.24	20.29	19.67	19.66	19.62	27.70	27.78	27.89				23.89	23.67	NaN			
8 OPT/M/OD/3	20.16	19.76	20.19	NaN	18.51	18.58	19.25	19.32	19.44	27.20	27.01	27.23	21.88	21.93	22.00				25.57	25.34	25.44
95 OPT/E/OS/1	20.27	20.08	20.29	20.04	20.10	20.22	19.81	20.08	20.06	30.63	30.91	30.96	22.19	22.17	22.19						
95 OPT/E/OS/2	22.40	22.22	22.05	21.63	21.73	21.62	21.17	21.04	20.97	32.56	32.59	32.78				29.10	29.02	28.69			
95 OPT/E/OS/3	20.42	20.41	20.40	20.23	20.18	20.28	20.03	20.10	19.97	31.09	30.97	31.03	22.65	22.68	22.61				26.12	26.13	26.29

Table S18. The results of RNA-Seq data verification with the RT-qPCR. Presented log2FC values were calculated based on means of 2^-ΔΔCT values of the CE samples (patients with post-LVC ectasia vs controls) obtained for particular genes and *TRs* (as indicated in the first column).

gene, *TR*	log2FC, RNAseq	log2FC, RTqPCR
CADPS, TR1	-2.3578	-2.2601
CADPS, TR2	-2.0253	-2.1624
CADPS, TR3	-2.0206	-2.8974
S100A14, TR1	0.8008	0.0031
S100A14, TR3	0.6517	0.1889
CPT1B, TR2	-0.8508	-2.8602
KLK5, TR3	1.8456	0.4802

Abbreviations: post-LVC ectasia – post-laser vision correction ectasia; *TR1 – central topographic region*; *TR2 – middle topographic region*; *TR3 – peripheral topographic region*; log2FC - log2 transformed fold change.

Table S19. The RT-qPCR data used for the validation of study results. The Ct values obtained towards validation of the relative expression of *CADPS* and *CPT1B* genes in each of 21 additional CE samples and three CE samples included in MALDI-TOF/TOF MS but not RNA-Seq experiments (samples: 146 OPT/E/OS/1, 146/OPT/E/OS/2, and 146 OPT/E/OS/3). The expression of *LDHA*, *RLP4*, and *UBC* genes was used for data normalization. Each reaction was performed in triplicates, and if one of the replicates deviated by ≥0.5 Ct it was removed (these replicates are marked below as 'NaN').

Sample	*LDHA*	*LDHA*	*LDHA*	*RPL4*	*RPL4*	*RPL4*	*UBC*	*UBC*	*UBC*	*CADPS*	*CADPS*	*CADPS*	*CPT1B*	*CPT1B*	*CPT1B*
146 OPT/E/OS/1	20.64	20.53	20.33	20.20	20.33	20.29	19.84	19.76	19.82	27.02	27.25	27.16			
146 OPT/E/OS/2	20.70	20.63	20.37	20.41	21.30	20.53	20.36	20.41	20.21	27.07	26.84	27.03	28.87	29.28	28.77
146 OPT/E/OS/3	20.11	20.08	20.11	19.55	19.60	19.61	19.92	19.90	19.82	27.63	27.93	27.61			
176 OPT/E/OD/1	20.40	20.59	20.31	19.99	20.06	20.27	19.00	19.80	19.47	26.78	27.07	26.94			
176 OPT/E/OD/2	20.81	20.65	20.50	20.57	20.61	20.51	20.51	20.37	20.21	27.16	27.12	27.26	28.10	28.04	28.27
176 OPT/E/OD/3	20.52	20.73	20.48	20.53	20.42	21.04	20.29	20.45	20.40	27.45	27.89	27.37			
178 OPT/E/OD/1	20.52	20.48	20.23	21.03	20.42	20.52	19.36	19.74	19.73	27.68	27.66	27.75			
178 OPT/E/OD/2	21.09	21.13	NaN	20.89	20.93	21.04	20.81	20.66	20.25	28.17	28.21	27.88	27.95	27.64	27.46
178 OPT/E/OD/3	20.28	20.82	20.34	20.31	20.20	20.93	20.39	20.24	20.50	28.26	28.10	28.27			
187 OPT/E/OS/1	20.17	20.47	20.38	20.54	20.38	20.40	19.47	19.84	19.66	29.72	29.94	29.92			
187 OPT/E/OS/2	20.79	20.54	20.40	20.89	20.74	20.79	21.08	21.10	20.88	30.43	30.37	30.56	28.28	28.12	28.68
187 OPT/E/OS/3	19.96	20.39	19.91	20.27	20.33	20.32	20.10	20.27	20.26	29.46	29.67	29.39			
188 OPT/E/OS/1	21.20	21.14	21.28	21.22	21.35	21.25	20.21	20.88	20.23	29.34	29.46	29.40			
188 OPT/E/OS/2	23.05	23.02	23.08	23.08	23.23	22.86	22.71	22.53	22.46	32.19	30.54	30.45	30.14	29.58	30.02
188 OPT/E/OS/3	21.12	21.37	21.19	21.44	21.40	21.47	20.81	21.07	20.83	29.36	29.43	29.30			
188 OPT/E/OD/1	21.11	21.16	21.15	21.12	21.08	21.19	20.65	20.56	20.67	29.40	29.13	29.31			
188 OPT/E/OD/2	20.93	20.92	21.06	20.76	21.04	20.84	20.83	20.70	20.66	29.57	29.43	29.36	28.33	28.09	28.11
188 OPT/E/OD/3	20.42	20.31	21.12	20.55	20.57	20.50	20.09	19.93	20.08	29.54	29.67	29.59			
195 OPT/E/OS/1	20.15	20.54	19.87	20.40	21.75	20.91	20.11	20.30	20.34	30.65	30.73	30.80			
195 OPT/E/OS/2	20.45	20.72	20.35	20.42	20.17	20.29	20.14	20.41	20.19	31.26	30.79	30.69	28.10	28.21	27.65
195 OPT/E/OS/3	20.04	19.97	20.12	20.08	20.04	20.12	19.63	19.84	19.53	30.38	30.08	30.19			
195 OPT/E/OD/1	20.81	20.37	20.34	20.98	20.39	20.67	19.59	19.64	19.62	30.85	30.85	30.68			
195 OPT/E/OD/2	20.55	20.79	20.73	20.40	20.45	20.56	20.00	20.23	20.31	30.99	31.00	30.88	28.02	27.28	27.58
195 OPT/E/OD/3	20.78	20.81	21.01	20.68	20.50	21.00	20.63	20.04	20.27	30.32	30.42	30.58			

Table S20. Quantification of fluorescence intensity of TBC1D4 and CIITA in CE samples.

Fluorescence intensities of TBC1D4 (*blue* channel) and CIITA (*green* channel) were quantified using Fiji (ImageJ) within selected regions of interest (ROIs) from 3D confocal images of CE samples obtained from a patient with post-LVC ectasia (#195 OPT/E/OD) and a patient with keratoconus (#207 OPT/KTCN/OS). For each marker, both raw (non-normalized) and normalized fluorescence values are presented. Normalization was performed relative to the nuclear signal from propidium iodide (PI, *red* channel). The table includes ROI area, as well as mean, median, minimum, and maximum intensity values for each condition.

ID	Channel	ROI area	Mean intenisty	Minimal intensity	Maximum intensity	Median intensity	Mean intenisty normalized	Minimal intensity normalized	Maximum intensity normalized	Median intensity normalized
195 OPT/E/OD	red_PI	21.037	52.053	1	85	57	-	-	-	-
207 OPT/KTCN/OS	red_PI	21.037	45.097	0	85	44	-	-	-	-
195 OPT/E/OD	blue_TBC1D4	21.037	15.008	5	37	15	0.2883	5	0.4353	0.2632
207 OPT/KTCN/OS	blue_TBC1D4	21.037	12.213	3	28	12	0.2708	-	0.3294	0.2727
195 OPT/E/OD	green_CIITA	21.037	5.805	2	30	6	0.1115	2	0.3529	0.1053
207 OPT/KTCN/OS	green_CIITA	21.037	9.561	2	82	9	0.7829	0.6667	2.9286	0.75

3. Supplementary Figures

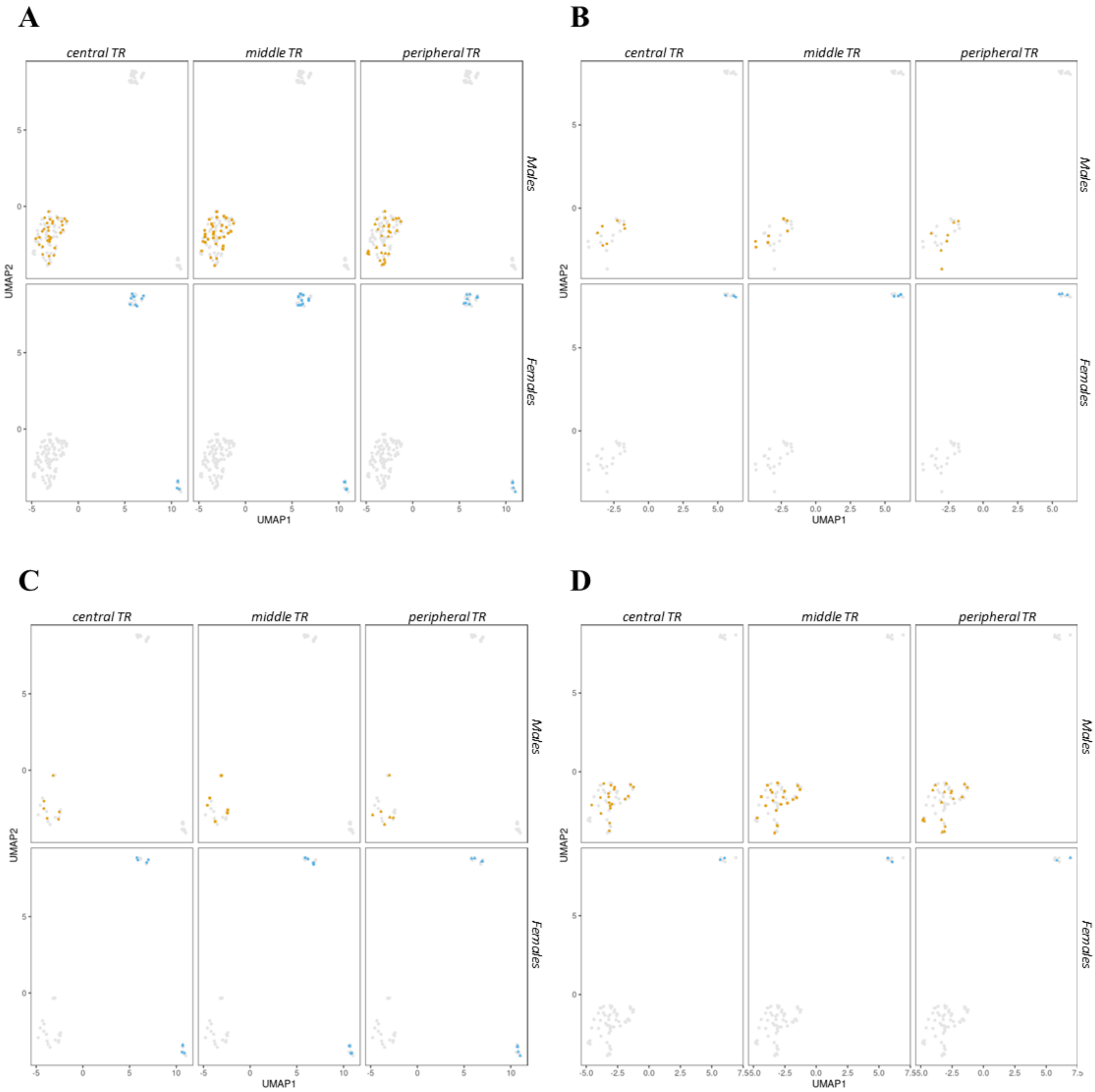

Figure S1. UMAP (Uniform Manifold Approximation and Projection) plots of RNA-Seq transcriptome data illustrating sex of patients (orange for male patients and blue for female patients) with *topographic regions* (*TRs*) visualized in separate panels. (**A**) UMAP plot displaying samples all study subgroups, (**B**) UMAP plot showing samples from patients with post-LVC ectasia, (**C**) UMAP plot showing samples from controls, and (**D**) UMAP plot showing samples from patients with KTCN. Notably, sex, rather than disease status, emerges as the primary covariate influencing clustering in the RNA-Seq data.

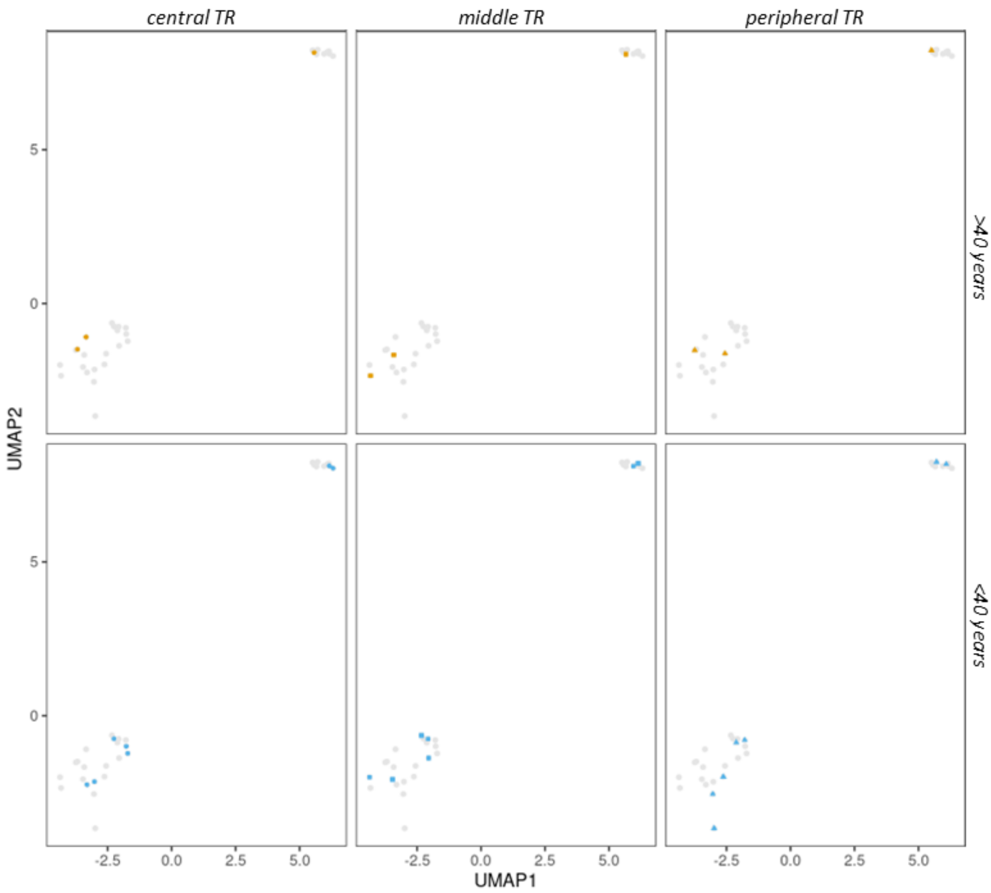

Figure S2. UMAP plot of RNA-Seq transcriptome data from patients with post-LVC ectasia, illustrating patient age (orange: patients >40 years; blue: patients <40 years), with *topographic regions* (*TRs*) visualized in separate panels. Age did not emerge as a covariate influencing clustering in the RNA-Seq data.

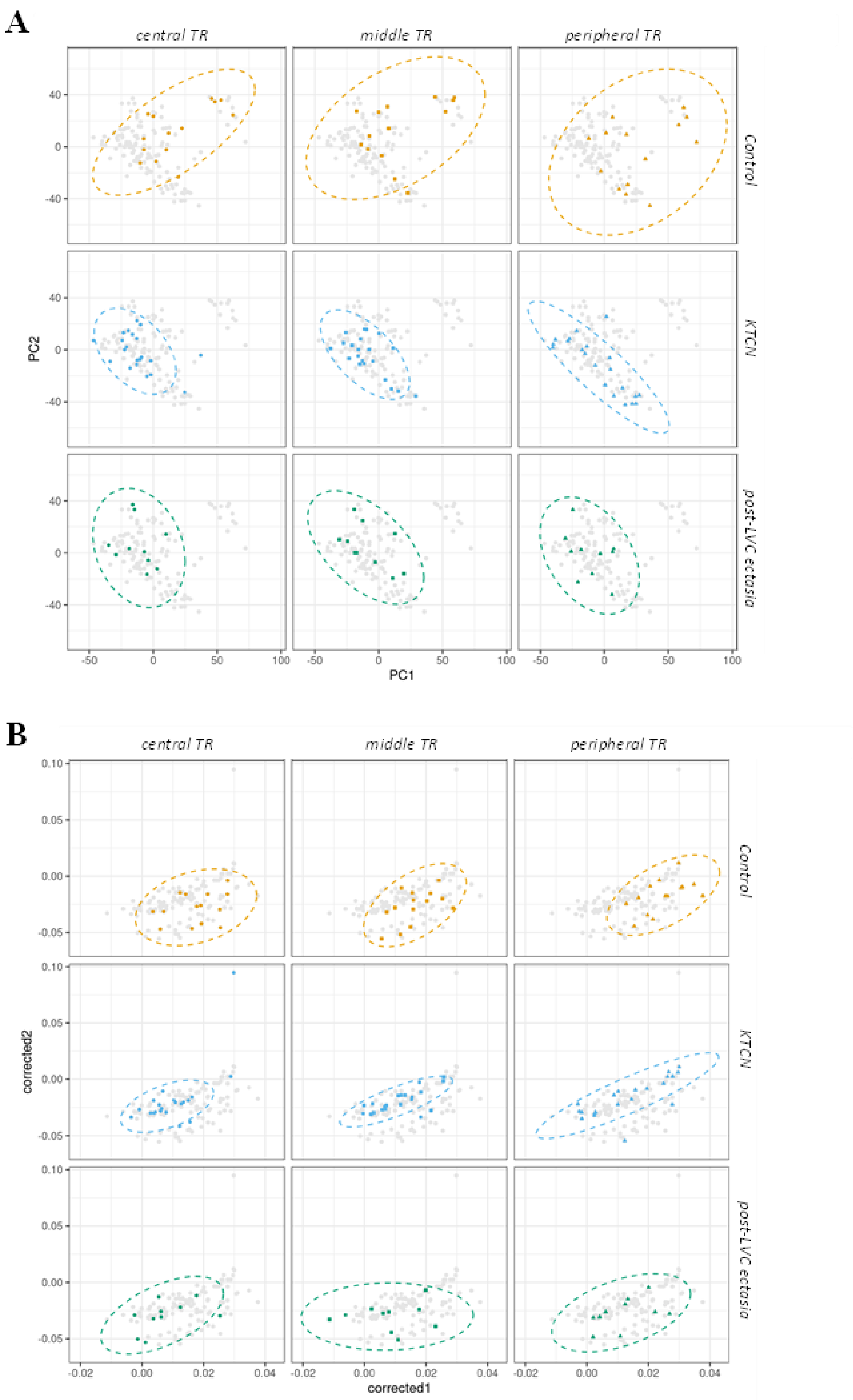

Figure S3. PCA plots of RNA-Seq transcriptome data illustrating experimental batch effects. Patient subgroups are color-coded: orange for control individuals, blue for patients with keratoconus (KTCN), and green for patients with post-LVC ectasia. *TRs* are shown in separate panels. **(A)** PCA plot before batch correction; **(B)** PCA plot after batch correction using the fastMNN() function, accounting for the covariate of NGS library preparation date (six levels). No substantial changes in sample distribution were observed before and after batch correction, indicating minimal batch effect in this dataset.

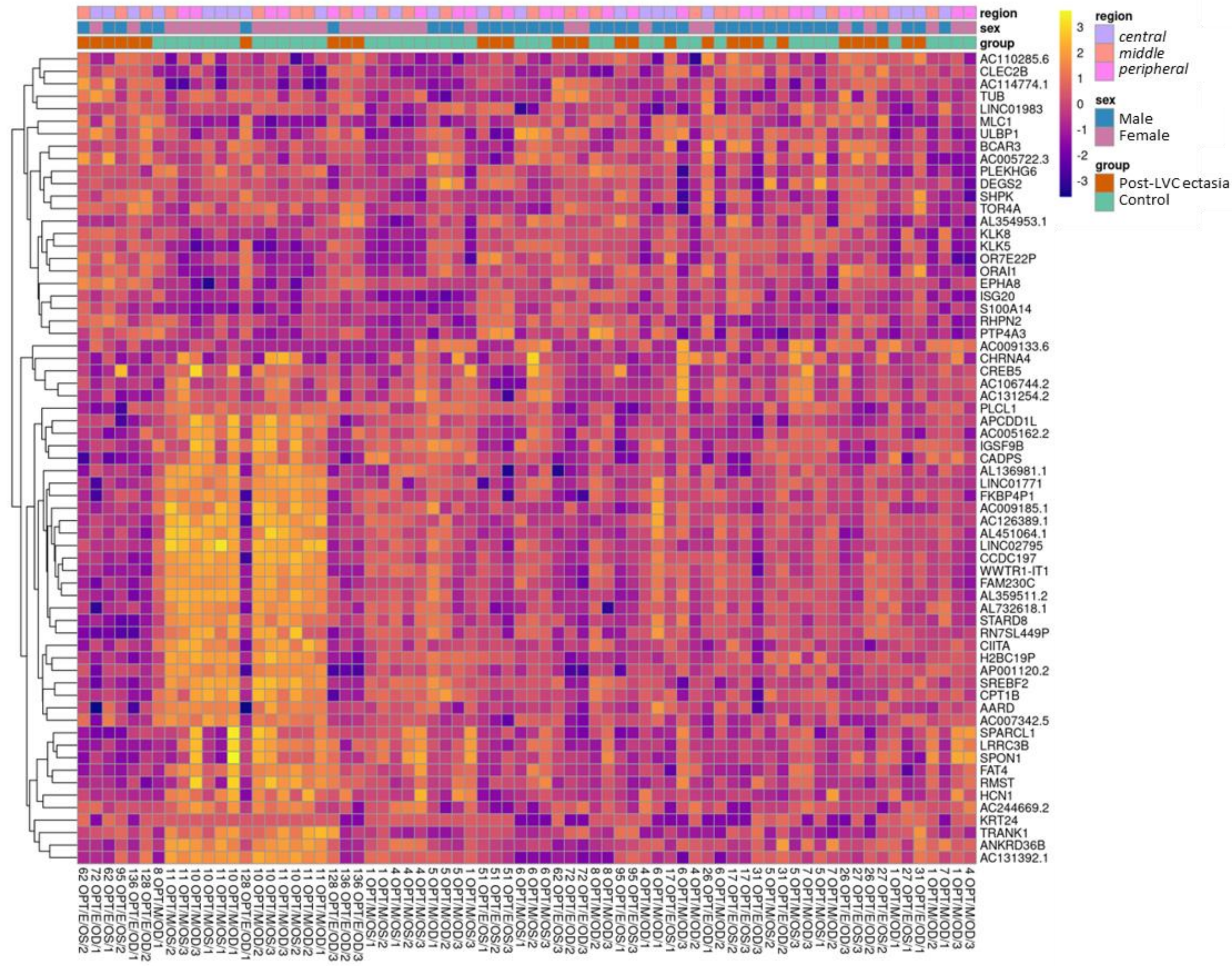

Figure S4. Heatmap of RNA-Seq transcriptome analysis for 65 selected genes from differential expression analysis in post-LVC ectasia. Colors correspond to data normalized using library size factors, log-transformed, and scaled (each gene had a mean of zero and a standard deviation of one), dark violet to bright yellow color gradient denotes lower to higher expression. Genes (in rows, *n*=65) and samples (in columns, *n*=72) were hierarchically clustered based on Euclidean distances. Presented genes correspond to the top differentially expressed genes (DEGs) in post-LVC ectasia (see Table S7) with our gene of interest (*CIITA*). Presented samples correspond to the sample list from Table S2 (no. 1-72). On top of the heatmap are control samples in green squares and post-LVC ectasia samples in red squares, whereas the sex of patients indicated by blue color for males and pink color for females, and the central TR is annotated with mauve, middle-peach, and peripheral-fuchsia colors.

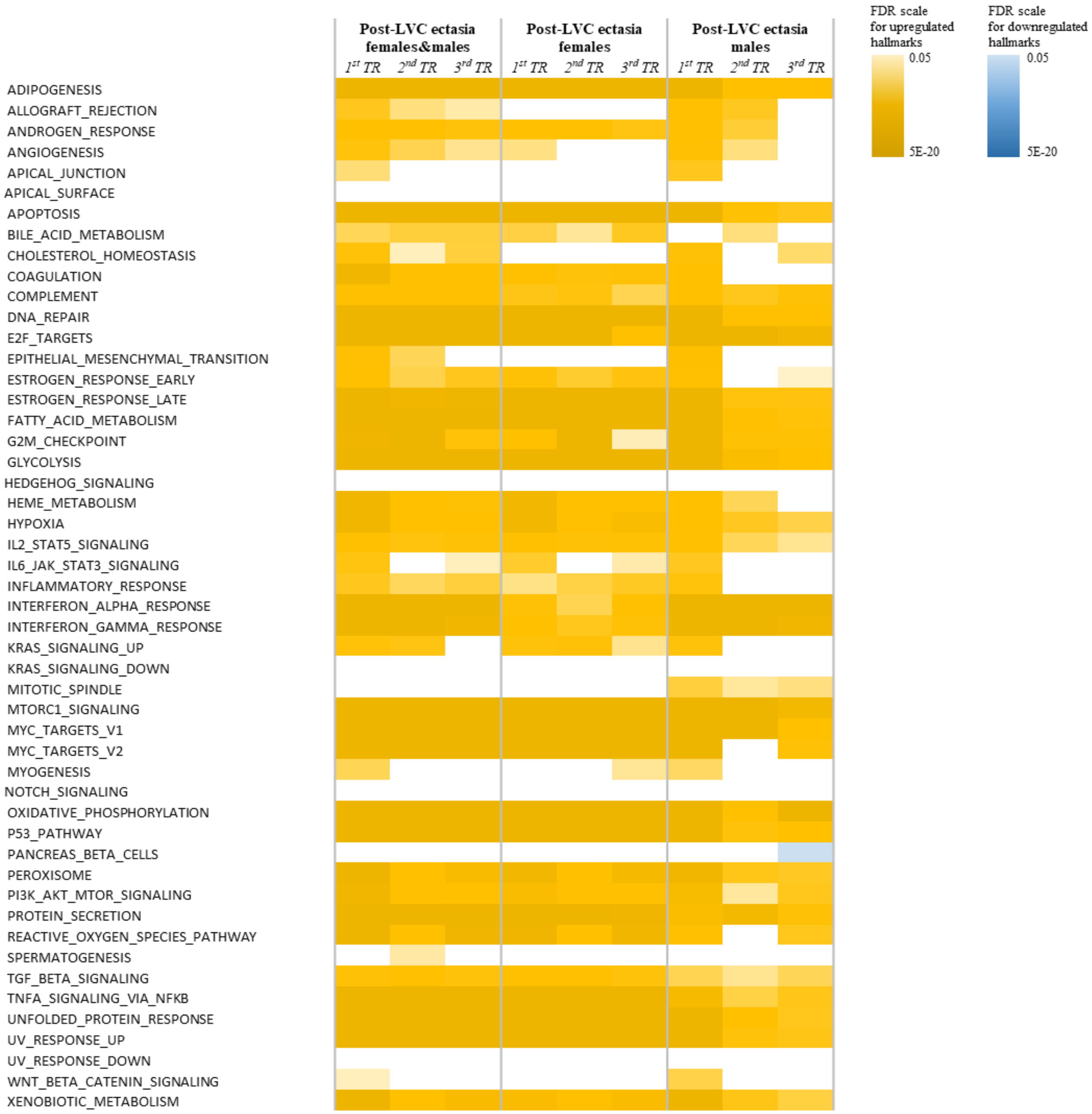

Figure S5. The hallmark pathways differentially enriched in particular CE *TRs regions* of patients with post-LVC ectasia, in comparison to corresponding *TRs* of CE of control individuals. Enrichment analysis was performed in three settings depending on the sex of the individuals included. The color scales represent false discovery rate (FDR) values for each upregulated and downregulated hallmark. For detailed information on values see Table S8.

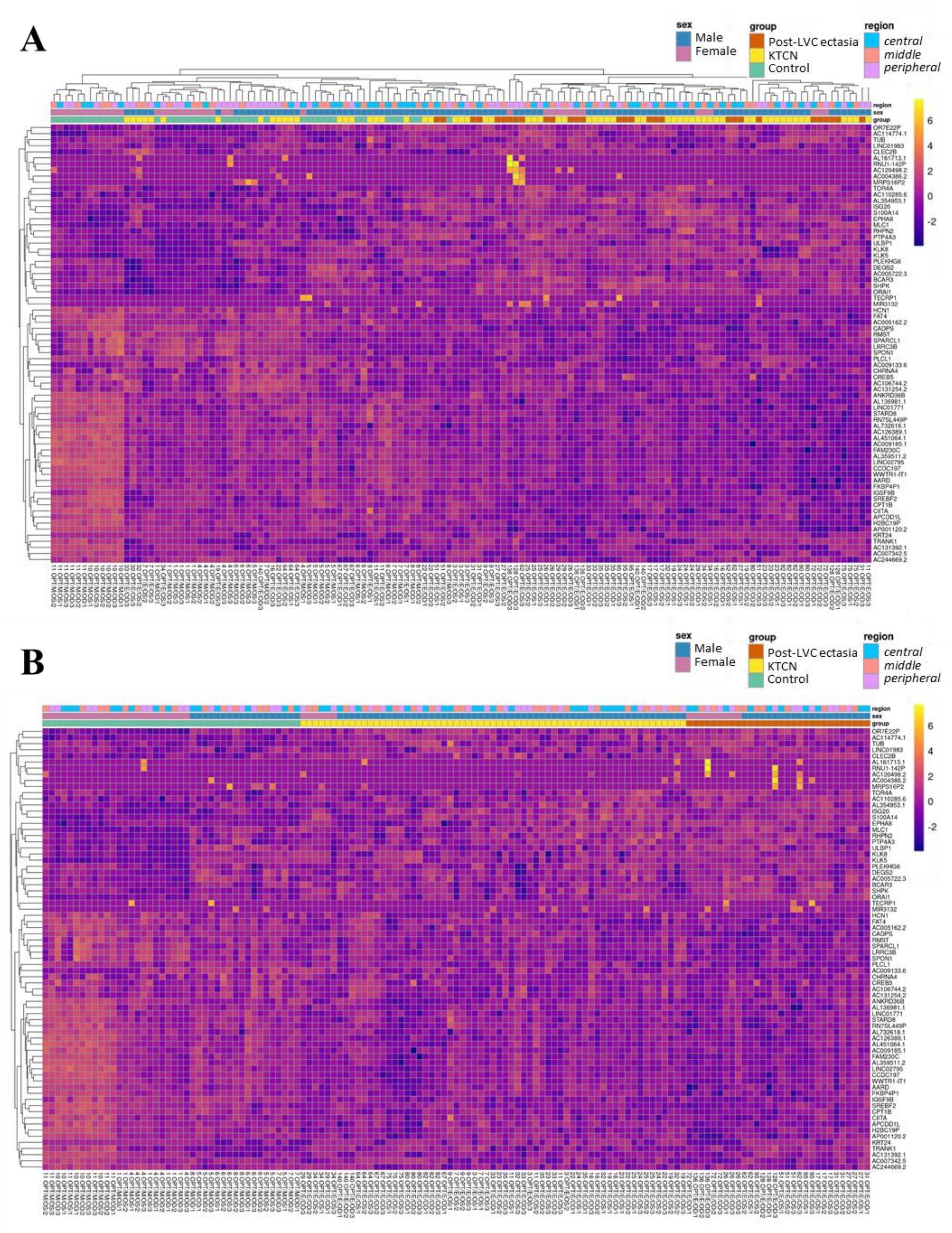

Figure S6. Heatmap of RNA-Seq transcriptome analysis for 72 selected genes from differential expression analysis in post-LVC ectasia, KTCN and control individuals. (**A**) Genes (in rows, *n*=72) and samples (in columns, *n*=135) were hierarchically clustered based on Euclidean distances. (**B**) Genes (in rows, *n*=72) were hierarchically clustered based on Euclidean distances. Colors correspond to data normalized using library size factors, log-transformed, and

scaled (each gene had a mean of zero and a standard deviation of one). Presented genes correspond to the top differentially expressed genes (DEGs) in post-LVC ectasia and KTCN (see Table S7 and Table S10) with our gene of interest (*CIITA*). Presented samples correspond to the sample list from Table S4 (no. 1-135). On top of the heatmap are post-LVC samples samples in red squares, KTCN samples in yellow squares, and control samples in green squares, whereas the sex of patients is indicated by blue color for males and pink color for females, and the central TR is annotated with mauve, middle-peach, and peripheral-fuchsia colors.

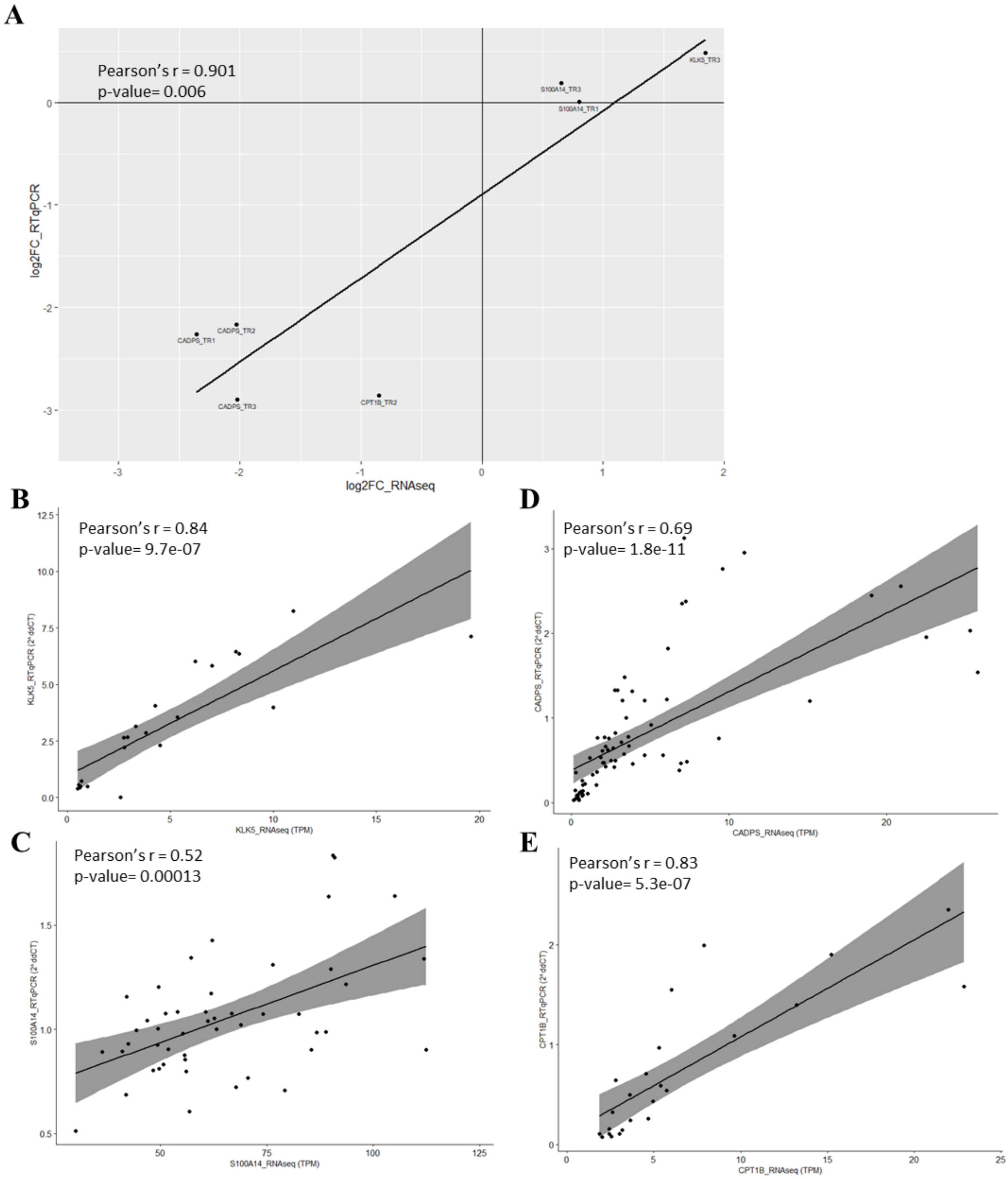

Figure S7. Correlation plots validating transcriptomic findings. **(A)** Summary plot showing the relationship between RT-qPCR results (log_2FC, Y-axis) for selected genes differentially expressed in CE samples across particular *TRs* in patients with post-LVC ectasia. Presented log2FC values were calculated based on means of 2^-ΔΔCT values of the CE samples (patients with post-LVC ectasia vs controls). The text labels indicate the gene and particular *TR* (*TR1* as *central TR*, *TR2* as *middle TR*, and *TR3* as *peripheral TR*). For details on log2FC values please see Table S18. **(B–E)** Correlation plots comparing gene expression levels obtained by RNA-seq (X-axis, TPM) and RT-qPCR (Y-axis, 2^–ΔΔCT) for: **(B)** KLK5, **(C)** S100A4, (**D**) CADPS, and (**E**) CPT1B.

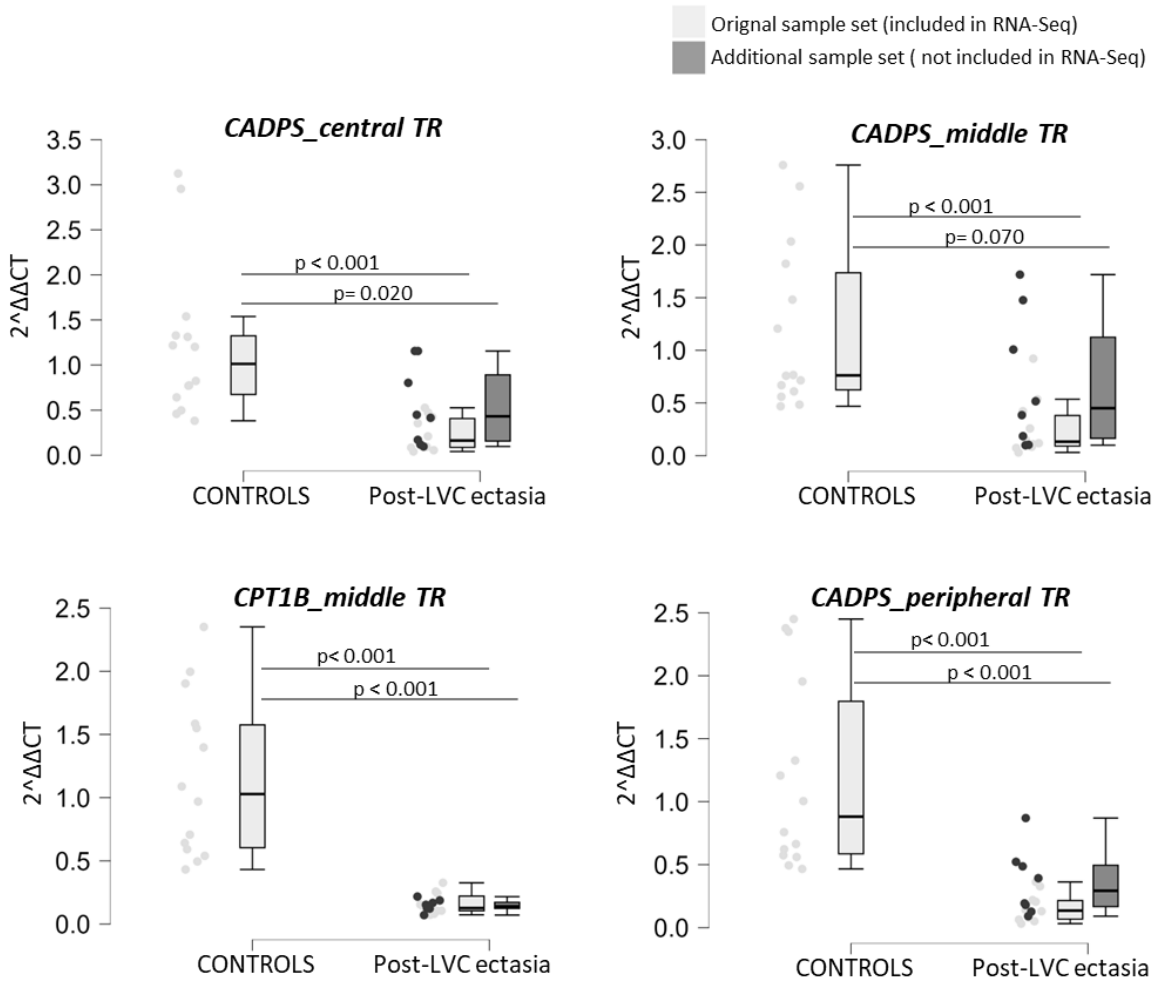

Figure S8. Box plots showing relative expression level of two selected genes, *CADPS* and *CPT1B*, specific for patients with post-LVC ectasia in original and extended sample set. Relative quantification of the gene expression was normalized to the level of the *UBC*, *LDHA*, and *RPL4* genes with the comparative CT method. The differences in expression levels of selected genes in particular *TRs* were verified Mann-Whitney U test. Symbols of gene name and particular *TR* (*TR1* as *central TR*, *TR2* as *middle TR*, and *TR3* as *peripheral TR*) are showed above each plot, on y-axis 2^ΔΔCT values are presented, with bright gray color original experimental samples are showed and with dark grey samples from additional post-LVC ectasia patients. For detailed information on CT values of each sample and gene please see Table S17 and Table S19.

Supplementary References

1. Jaskiewicz K, Maleszka-Kurpiel M, Matuszewska E, Kabza M, Rydzanicz M, Malinowski R, Ploski R, Matysiak J, Gajecka M. The Impaired Wound Healing Process Is a Major Factor in Remodeling of the Corneal Epithelium in Adult and Adolescent Patients With Keratoconus. *Invest Ophthalmol Vis Sci* (2023) 64:22. doi: 10.1167/iovs.64.2.22

2. Czugala M, Karolak JA, Nowak DM, Polakowski P, Pitarque J, Molinari A, Rydzanicz M, Bejjani BA, Yue BYJT, Szaflik JP, et al. Novel mutation and three other sequence variants segregating with phenotype at keratoconus 13q32 susceptibility locus. *Eur J Hum Genet EJHG* (2012) 20:389–397. doi: 10.1038/ejhg.2011.203

3. Kabza M, Karolak JA, Rydzanicz M, Szcześniak MW, Nowak DM, Ginter-Matuszewska B, Polakowski P, Ploski R, Szaflik JP, Gajecka M. Collagen synthesis disruption and downregulation of core elements of TGF-β, Hippo, and Wnt pathways in keratoconus corneas. *Eur J Hum Genet EJHG* (2017) 25:582–590. doi: 10.1038/ejhg.2017.4

4. JASP Team 2022. JASP Team (2022). JASP (Version 0.16.3)[Computer software]. (2022) https://jasp-stats.org/

5. Law CW, Alhamdoosh M, Su S, Dong X, Tian L, Smyth GK, Ritchie ME. RNA-seq analysis is easy as 1-2-3 with limma, Glimma and edgeR. *F1000Research* (2016) 5:ISCB Comm J-1408. doi: 10.12688/f1000research.9005.3

6. Ritchie ME, Phipson B, Wu D, Hu Y, Law CW, Shi W, Smyth GK. limma powers differential expression analyses for RNA-sequencing and microarray studies. *Nucleic Acids Res* (2015) 43:e47. doi: 10.1093/nar/gkv007

7. Liberzon A, Birger C, Thorvaldsdóttir H, Ghandi M, Mesirov JP, Tamayo P. The Molecular Signatures Database (MSigDB) hallmark gene set collection. *Cell Syst* (2015) 1:417–425. doi: 10.1016/j.cels.2015.12.004

8. Gillespie M, Jassal B, Stephan R, Milacic M, Rothfels K, Senff-Ribeiro A, Griss J, Sevilla C, Matthews L, Gong C, et al. The reactome pathway knowledgebase 2022. *Nucleic Acids Res* (2022) 50:D687–D692. doi: 10.1093/nar/gkab1028

9. Stein LD. Using the Reactome Database. *Curr Protoc Bioinforma* (2004) 7: doi: 10.1002/0471250953.bi0807s7

10. Wu D, Smyth GK. Camera: a competitive gene set test accounting for inter-gene correlation. *Nucleic Acids Res* (2012) 40:e133. doi: 10.1093/nar/gks461

11. McCarthy DJ, Campbell KR, Lun ATL, Wills QF. Scater: pre-processing, quality control, normalization and visualization of single-cell RNA-seq data in R. *Bioinforma Oxf Engl* (2017) 33:1179–1186. doi: 10.1093/bioinformatics/btw777

12. R Core Team. R Core Team (2024). R: A language and environment for statistical computing. R Foundation for Statistical Computing, Vienna, Austria. https://www.R-project.org. Accessed 17 July 2024. (2024) https://www.R-project.org/

13. Kolde R. pheatmap: Pretty Heatmaps. R package version 1.0.12. https://cran.r-project.org/web/packages/pheatmap/index.html. Accessed 16 July 2024. (2019)

14. Ren S, Zhang F, Li C, Jia C, Li S, Xi H, Zhang H, Yang L, Wang Y. Selection of housekeeping genes for use in quantitative reverse transcription PCR assays on the murine cornea. *Mol Vis* (2010) 16:1076–1086.

15. Karolak JA, Ginter-Matuszewska B, Tomela K, Kabza M, Nowak-Malczewska DM, Rydzanicz M, Polakowski P, Szaflik JP, Gajecka M. Further evaluation of differential expression of keratoconus candidate genes in human corneas. *PeerJ* (2020) 8:e9793. doi: 10.7717/peerj.9793

16. Matuszewska E, Matysiak J, Rosiński G, Kędzia E, Ząbek W, Zawadziński J, Matysiak J. Mining the Royal Jelly Proteins: Combinatorial Hexapeptide Ligand Library Significantly Improves the MS-Based Proteomic Identification in Complex Biological Samples. *Molecules* (2021) 26:2762. doi: 10.3390/molecules26092762
